# Supplementary material for: It’s not black and white: Perspectives of Western Canadian beef farmers on dairy-beef production
Source: PLoS One. 2025 Sep 10;20(9):e0330697. doi: 10.1371/journal.pone.0330697 (PMC12422455; doi:10.1371/journal.pone.0330697)
Supplement: S1 File — Interview guide used to explore beef farmers’ views on dairy-beef production, including open-ended questions and follow-up prompts. (PDF) [file pone.0330697.s001.pdf]

Consent for Participating in Interviews: Dairy supply to Beef

Canadian beef stakeholders' views on the dairy supply to the beef industry

I. STUDY TEAM

Principal Investigator:

Dr. Marina von Keyserlingk, Professor, The University of British Columbia, Faculty of Land and Food Systems, Animal Welfare Program, 604-306-5721, marina.vonkeyserlingk@ubc.ca

Co-Investigator(s):

1. Dr. Daniel Weary, Professor, The University of British Columbia, Faculty of Land and Food Systems, Animal Welfare Program, 604-822-3954, dan.weary@ubc.ca
2. Bianca Vandresen, PhD Student, The University of British Columbia, Faculty of Land and Food Systems, Animal Welfare Program, 778-539-8675, bvandres@mail.ubc.ca

II. SPONSOR

Who is funding this study?

This research is funded a Social Sciences and Humanities Research Council Insight Grant awarded to MvK and DMW.

III. INVITATION AND STUDY PURPOSE

Why are we doing this study?

We are conducting this study to explore the views of professionals involved in the Canadian beef production system about the dairy supply to the beef industry, including cull dairy cows, veal calves, and dairy-beef calves. Findings will form part of Bianca Vandresen's PhD thesis.

Why you have been asked to take part in this study?

You have been invited to take part in this study because you are a professional in the Canadian beef industry, and are of legal consenting age of 19 years.

IV. STUDY PROCEDURES

What happens if you consent to participate in this study?

The interviewer will give a brief introduction on the goal of the study, and will review expectations. You will then be asked a series of questions pertaining to the dairy supply to the beef industry in Canada and prompted to discuss your views with the interviewer. The interview is expected to take no longer than 60 min and the session will be audio-recorded for transcription purposes.

V. STUDY RESULTS

How will results from this study be shared?

The results of this study may be published in academic journals, books and presented at academic and industry conferences. Data from the interview will be collected and made publicly available at the time of publishing. However, only answers to questions will be

collected as data and made available. No data on your personal information or identity will be collected (i.e., all data collected will be de-identified).

Would you like to receive the results of the final study?
We will ask you to indicate if you would like to receive information on the final results of this study during the interview.

VI. POTENTIAL RISKS OF THE STUDY
Is there any way being in this study could be bad for you?
We do not think there is anything in this study that could harm you. Please let the interviewer know if you have any concerns. You do not have to answer any question if you do not want to and you can stop the interview at any time.

VII. POTENTIAL BENEFITS OF THE STUDY
What are the benefits of participating?
You may benefit by having an opportunity to share and express your views on the topic and ask questions about the dairy supply to the beef industry. We also believe the beef industry will benefit from the findings of this study.

VIII. CONFIDENTIALITY
How will your privacy be maintained?
You will be only asked to provide basic demographic information (e.g. province or state), the type of beef operation and the number of head of cattle on your farm or on the farms you operate on. Information that discloses your identity will not be released without your consent unless required by law.

The sessions will be audio-recorded. Recordings will be transferred immediately after the interview onto 2 password-protected encrypted hard-drives after which the audio-recorders will be formatted to erase all the data. All identifying information will be removed in transcription of the audio files, and you will be assigned a participant ID to insure anonymity.

All documents and data will be identified only by participant ID and kept in a locked filing cabinet, or if kept on a computer, in a password protected and encrypted file on a secure UBC server. These files will only be accessible to the research team. You will not be identified by name in any reports of the completed study.

If your interview is online, it will be held over Zoom. UBC licensed Zoom servers are located within Canada and Zoom stores your name and information. We ask that you log in using only a nickname or a substitute name.

IX. PAYMENT
Will you be paid for your time/ taking part in this research study?
You will not be paid for the time you take to be in this study. You will be offered a $10 gift card to a coffee shop in your area.

X. CONTACT FOR INFORMATION ABOUT THE STUDY
Who can you contact if you have questions about the study?
If you have any questions or concerns about what we are asking of you, please contact the study leader or one of the study staff. The names and telephone numbers are listed at the top of the first page of this form.

XI. CONTACT FOR COMPLAINTS
Who can you contact if you have complaints or concerns about the study?
If you have any concerns or complaints about your rights as a research participant and/or your experiences while participating in this study, contact the Research Participant Complaint Line in the UBC Office of Research Ethics at 604-822-8598 or if long distance e-mail RSIL@ors.ubc.ca or call toll free 1-877-822-8598.

XII. PARTICIPANT CONSENT
Taking part in this study is entirely up to you. You have the right to refuse to participate in this study. If you decide to take part, you may choose to pull out of the study at any time without giving a reason.

- We will ask you to indicate that you have received a copy of this consent form for your own records at the start of the interview.

- We will ask you to indicate that you consent to participate in this study at the start of the interview.

It’s not black and white: Perspectives of Western Canadian beef farmers on dairy-beef production

Bianca Vandresen1, Daniel M. Weary1, Marina A. G. von Keyserlingk[1]*

[1]Animal Welfare Program, Faculty of Land and Food Systems, The University of British Columbia, Vancouver, BC V6T 1Z6 Canada

*Corresponding author
Email: nina@mail.ubc.ca (MvK)

Supplementary Material 1. Semi-structured interview guide used to guide the interviews with Canadian beef farmers (n=20) about dairy-beef production.

House-keeping

- Do you have any questions about the consent form?
    - The form states that participation is anonymous and voluntary and that the conversation will be recorded. This is to ensure that I don’t miss any information and that I only write down exactly what you say, not my interpretation of what you say. The audio will then be transcribed, anonymized, and sent back to you for approval. Does that sound okay to you?
- Are you ok if I start recording the conversation?
- **START RECORDING**
- The last thing we need to do is confirm your consent to participate.
- Can you confirm that you agree to participate in the study?

Participant’s information

- Now that all the housekeeping is out of the way, can you start by sharing a little bit about yourself and your experience with beef production? For example:
    - Years of experience with beef production
    - Type of beef operation you have/work with
    - Where it is located (province)
    - Average number of animals on-farm / number of animals produced per year

Beef-dairy relationship in Canada

1) How would you describe the relationship between the beef and the dairy industry in Canada?
    - What would you say these two industries have most in common?
    - And on what do you think they differ the most?

2) We started thinking about the dairy-beef relationship because, inevitably, animals from the dairy industry enter the beef supply chain. According to the Canadian Cattle Association, about 20% of the meat production in Canada comes from the dairy industry.
    - What do you think about this number?
        - Is it higher or lower than you expected? Why?

[redacted]

Those are mainly animals that are not fit for dairy production for some reason, and the main group of dairy animals that go to beef are the surplus dairy calves.

You may already know this, but dairy farmers produce more calves than they need to replace in the milking herds, so they have many surplus (or non-replacement) dairy calves. Historically in Canada, these calves have usually been of pure dairy breeds and sold to the veal supply. However, some dairy farmers have started to use beef genetics in a portion of their dairy cows and have started producing dairy-beef calves. These dairy beef calves can be sold to the veal supply, but some may enter the beef supply chain and be raised by beef farmers. This change will possibly result in more supply coming from dairy farms into the markets that beef farmers target. And animals coming from the dairy and beef systems would reach the market in a similar way.

3) Have you heard about using beef genetics in dairy herds before?
- What have you heard?

4) What's your opinion on the use of beef genetics in dairy cows? Do you see any challenges or opportunities for the beef industry because of it?
- And to the dairy industry?
- Do you have any questions or concerns about this? What would you like to know more regarding this practice?
- What's your experience with dairy-beef calves?

If the participant has experience:
- Can you share more about how you manage those animals?
- Do you notice differences between pure beef, pure dairy, and dairy-beef calves? What are the main differences?
- What motivates you to purchase dairy-beef calves? (compared to pure beef and pure dairy)
- What are the main challenges you face with dairy-beef calves?

If the participant has no experience:
- Do you think beef farmers would be interested in purchasing dairy beef calves? Why/why not?
- Would you be interested in purchasing these animals in the future? Why/why not?

One of the things we consider about dairy beef calves is that these calves are raised as dairy calves for the first few days of life, under dairy farming conditions. They undergo dairy farming practices that differ from those used in cow-calf beef operations. For example, dairy calves are housed indoors and individually during their earliest days, which is similar to veal production, but not to beef.

5) What are your thoughts on the differing calf-rearing practices in dairy versus beef farming?
- Do you believe these different practices can impact the performance and success of calves in the beef supply chain? In what ways?
- What do you consider the main challenges of raising dairy-beef calves?

6) Imagine a close friend of yours is a dairy farmer, and they are interested in selling dairy-beef calves to the beef supply chain. What recommendations would you give What are the "must haves" of dairy-beef calf rearing while they are on dairy farms?

[redacted]

Consumers' views

In the dairy industry, there are numerous discussions about consumers' opinions on certain dairy farming practices that the public may not approve of, such as individual housing and cow-calf separation. These conversations often examine the potential influence of the media on public perception. I'm curious if similar discussions take place in the beef industry, considering that many of these practices are not common in beef operations. For instance, in cow-calf operations, the calf typically remains with the cow on pasture for several months.

7) Does the beef industry also have similar discussions?

8) Do you think the public thinks differently about dairy and beef production systems?

Cow-calf separation

As I briefly mentioned before, cow-calf separation is one of these practices that is not approved by the public. However, it is a common practice on almost all dairy farms to separate the calf from the cow right after birth or within the following hours. A lot of research has happened on this practice but we can still not reach an agreement on whether it is better or not for the animals. This practice does not happen in beef farms, but is the case for dairy surplus calves that enter the beef supply chain. So I'm curious on your thoughts about this.

9) What are your views about the cow-calf separation practice?

10) How do you think it can have an influence for the calf health and welfare?

Embryo transfer

Another practice that people are researching about, but it is not yet being adopted on many farms, is embryo transfer. In this case, dairy farmers would transfer a calf embryo that has 100% beef genetics into a dairy cow. This would result in a dairy cow producing a pure beef calf.

11) Have you heard about this before?

12) What is your opinion about this practice?

- Do you think it is a good idea? Why?
- Do you have any concerns about it?

Final questions

13) Considering everything we discussed about the dairy-beef calves, how do you see the future of this practice in Canada?

[redacted]

14) Who do you think should lead and take responsibility for the discussions about this practice?

- Is it something the dairy industry should take the lead, or the beef industry, or both?

15) Who else should be at the table for these discussions?

- E.g. genetic companies, nutritionists, etc.

16) For the last question, imagine we have a time machine and travel 30 years into the future. What do you think beef production will look like in Canada then?

Final thoughts

17) Is there anything else you have thought about this topic that I didn't ask you about but that you would like to share?

THANK YOU AND STOP RECORDING

Follow up actions

- Now, I will transcribe our conversation, and you are welcome to have a **copy of this transcript.** Would you like to send it to you? Note the email address to send it.
- This interview will collaborate with the publication of a research paper. Would like to **receive the results** of the study once published?
- Please feel free to invite others to participate. You can share my email and phone number.
- $10 Tim Hortons voucher via email.
