# Supplementary material for: It’s not black and white: Perspectives of Western Canadian beef farmers on dairy-beef production
Source: PLoS One. 2025 Sep 10;20(9):e0330697. doi: 10.1371/journal.pone.0330697 (PMC12422455; doi:10.1371/journal.pone.0330697)
Supplement: S2 Table — List and definitions of themes and sub-themes developed through thematic analysis of the interview transcripts. (PDF) [file pone.0330697.s002.pdf]

It’s not black and white: Perspectives of Western Canadian beef farmers on dairy-beef production

Bianca Vandresen1, Daniel M. Weary1, Marina A. G. von Keyserlingk[1]*

[1]Animal Welfare Program, Faculty of Land and Food Systems, The University of British Columbia, Vancouver, BC V6T 1Z6 Canada

*Corresponding author
Email: nina@mail.ubc.ca (MvK)

Supplementary Material 2. Codebook with detailed description of the themes and codes developed in the thematic analysis of interview transcripts with Canadian beef farmers about dairy-beef production.

Themes and codes	Definition
Dairy & beef relationship	Participants' perspectives on the relationship between the dairy and beef industries in Canada. It includes insights into the similarities and differences in how these industries operate, their respective markets, and public perceptions. Additionally, it examines how the two sectors communicate with each other and the sentiments that participants believe exist between them.
Dairy & beef markets	Participants' views and experiences with the beef market and its differences from the dairy market, as well as the implications these two markets have on each other and their interrelationships. Also includes comments on the dairy input to the beef supply chain.
Farming operations	Participants' views on how the beef and dairy industries operate. It includes their opinions on each industry's different farm characteristics and animal management practices, as well as their views about the intensification of each operational approach.
Public Views	This section includes participants' perspectives on how the public perceives the beef and dairy industries and the reasons for these perceptions. It also includes participants' own opinions about the two industries.

Attitudes to beef-on-dairy animals	Participants' attitudes toward dairy-beef production, including positive, neutral, and negative views. The reasoning behind participants' attitudes covers various aspects, such as the perceived impact of dairy-beef production on the beef industry, perspectives on the genetics of dairy-beef calves, and the different management practices used to raise these animals.
Management Practices	Participants' views on the management practices used to rear dairy-beef calves in dairy farms. It includes comments on the implications of raising calves under dairy farming conditions, the animals' resiliency and fitness for entering the beef supply chain and how these practices affect the calves' success in the beef supply chain. It also includes participants' recommendations for preparing dairy-beef calves at dairy farms for beef production.
Impact on beef business	Participants' views about the impact of dairy-beef production on the beef market. This includes opinions on opportunities for collaboration and concerns regarding market competition and public perception.
The role of genetics	Participants' opinions and experiences with dairy-beef animals, focusing on their genetic traits for beef production. It includes views on meat quality and production, and the beef market acceptance of dairy-beef products.
A shared future	Participants' perceptions on the future of dairy-beef production. It includes their views on how discussions about dairy-beef production should be conducted, and who should be involved in these discussions, as well as the roles of the dairy and beef industries. Additionally, it covers comments on the potential impacts of dairy-beef production on the future of the beef industry.
