# Supplementary material for: It’s not black and white: Perspectives of Western Canadian beef farmers on dairy-beef production
Source: PLoS One. 2025 Sep 10;20(9):e0330697. doi: 10.1371/journal.pone.0330697 (PMC12422455; doi:10.1371/journal.pone.0330697)
Supplement: S3 File — Anonymized transcripts of the interviews conducted with 20 beef farmers, used for thematic analysis. (PDF) [file pone.0330697.s003.pdf]

INTERVIEW P4

[Verbal consent, introduction, and demographics were removed from the transcript to guarantee participants' anonymity]

Researcher
If you had to describe the relationship between the beef and dairy industries in Canada, how would you describe it?

Participant
Well, I don't know. I guess there's not too much of a crossover. Like it definitely seems like two different worlds. From my perspective, in the last few years, I've kind of made some friends with more dairy farmers, so talking with them and kind of making those connections you definitely see. Like we're both cattle producers. So we can relate on a lot of levels. But I feel like the beef world is almost in its own little bubble and then the dairy worlds in their own little bubble. And from my perspective, they don't seem to crossover a whole lot.

Researcher
And why do you say they are on their own bubbles? Why do you think they are like that?

Participant
I think just because it's a different it's a much different, like world, like it's just different production. Like beef producers, we produce our beef in a much different way like cow calf producers, let's say for example, like what I'm doing is quite a bit different than my friend who has a dairy farm. And just production is different and ways of doing things is different because you're producing something totally different.

Researcher
And what would you say are the things that the two industries differ the most on, and also have the most in common?

Participant
Um, I think one thing I've noticed a lot is almost numbers. And like for a cow calf producer, like a hundred, two hundred is a decently sizable production for most people. Versus a dairy production, they'll have like 400 cows or 500. And that's like, average. So I think number size is very different. But again, that just comes down to our different production, like everything in beef is usually in a cow calf on pasture and you need a lot more land versus in the dairy. They're in there in the barn, and they need less land coverage to house all those animals. And then so yeah, I would say that's kind of the difference I see. And then similarities. Like, we have a common goal we understand. I mean, at the end of the day, they're all cows, right. So we understand the same health issues in the same calving issues. And we definitely can relate on feed too. Like, they do get fed differently. But there's lots of nutritional things that are the same and that we share the same struggles, like getting hay and getting feed when there's a drought. And there's a lot of things we can relate on because we're both raising cows.

Researcher
Yeah, those similar access to resources in this sense?

Participant
Mm hmm.

Researcher
Oh, nice. I was interested in learning more about how much cross-over is there, between the dairy and in the beef, as you mentioned, and because inevitably, the dairy cows, they will go to the beef supply chain when they're not producing milk anymore. So those are the cull cows from the dairy, but also some of the calves would go to beef production. And I wanted to know how much of meat is actually coming from dairy. And according to the Canadian Cattle Association, there is already around 20% of meat production in Canada that comes from dairy. So I wanted to know, what is your opinion about this number, if it's higher than you expected? or lower?

Participant
Yeah, that's interesting. I've never heard that. I would say, I don't know if it's... Now that I think about it a little more. I don't think it's maybe higher than I expected. Like, I'm surprised to hear that it's 20%. Because I've never really thought of that. But just knowing that like, of course, that makes sense. When the cows are done, and they cull into the beef area, then. Yeah, I guess 20% does make sense for sure.

Researcher
Yeah, it also makes sense when we start seeing the amount of cows of dairy cows that need to be replaced every year. Because that dairy has a very short use of those animals, let's say. They replaced the cows very fast. So, I believe a big portion of this 20% are those cows, but also a main group of dairy animals that go to beef are the surplus dairy calves. You probably already know this but most of the calves produced on dairy farms cannot be used as replacement in the herd. So all the male calves and some of the females. And historically in Canada, most of those calves have been sold to the veal supply. But the veal consumption is declining, which means that those cows are having lower economic value in some regions specifically. And then to overcome this, some dairy farmers have started to use beef genetics in a portion of their dairy cows as a way to increase the economic value of those animals. So they are more genetically prepared to produce meat. So this change will possibly result in more supply coming from dairy to beef. So this 20% may be higher in the future. And then I was just wondering if you've heard about this practice before and what is your opinion about it?

Participant
Yeah, so I have heard a little bit about that. Um, about this practice, and I mean, honestly, like, it makes sense. And if you can still get good quality calves from it, like if you can mix a beef bull with a dairy cow, and you can get calves that are marketable and okay to go into the beef market, then I mean, I don't really see why not. I see why a dairy producer would want to do that.

Researcher
And do you see any reason why a beef producer would be interested in those animals?

Participant
Um, I mean, I guess if the animals are proving to be successful in production, like if they're performing the same way that just the regular beef animal would, then I don't see why a beef producer or a feedlot owner would have an issue with that. And as long as it's not like a big price difference or anything, which I don't see why it would be.

Researcher
And considering the genetics of the animals, and also how they are raised in dairy farms, do you see any challenges or opportunities of this practice to the beef producers?

Participant
Um, I mean, again, like I said, if the cross between dairy cows and beef cows are showing good and that if these animals are performing well, then I could see how this would be probably a positive thing for beef producers. And I mean, I know that some dairy cows have quite a large frame. So maybe mixing this large frame with the beef characteristics could end up beneficial. So yeah, I guess it just depends how these animals are performing when you mix them. And if they are performing good, then that could be a definite benefit to beef producers.

Researcher
Okay. Nice. And any challenges that come to mind?

Participant
Um, I mean, some things like that, like, with most farmers, change is kind of hard to adopt. So some beef producers might see this and go oh, that's not what we do, that's not what we like. So there might be a little reluctance to adopt this and to bring these animals into the beef stream. But yeah, I don't know, I guess. Yeah. Just kind of depends on what people... Yeah, I guess results and how things go and if people are willing to adopt this or not.

Researcher
Yeah, that makes sense. And one of the things that we think of about those dairy beef calves is that they're going to be sold in the market as beef, but they're dairy animals. And they are going to be raised under dairy farming conditions as at least in the first days of life, before they go to a beef operation. And the dairy practices are very different from beef on how they raise the animals, as you mentioned, in the beginning, it's almost two different worlds. So what do you think are possible implications of the different practices that happened in the two industries to those animals that come from dairy but are sold as beef?

Participant
Yeah. So I think that there could be a lot of health issues run into there. I know that lots of the time, feedlot owners usually have issues even when they're buying calves from the market and bringing them into the feedlot. There's usually a period of time where these calves are adjusting to the feedlot. And they're really vulnerable to get sick and to have issues in this period of adjustment. And that's just coming from, well, that's coming off a pasture into a feedlot setting. So I guess, coming from a dairy

operation into a beef operation that would be a concern, I would think is health issues because just adjusting an animal to a completely different environment, you're always going to run into challenges there.

Researcher
Yeah, yeah, exactly, especially when we move those animals from indoors to outdoors. Because in dairy, what usually happens is that they removed the calf from the cows soon after birth, which also doesn't happen in beef. And then they are housed individually and indoors until they go out of the farm. So they experience definitely a different life than if they were born in a beef farm.

Participant
Yeah.

Researcher
Okay, and then, let's say you have a friend that is a dairy farmer, and they are interested to sell their surplus calves to the beef farm. They're doing the beef on dairy genetics. But they want to know what practices they should do with those animals while they are in a dairy farm. What will be your recommendations to these friends? What should they do to guarantee that those calves will succeed in the beef supply chain?

Participant
I would make sure that you have a good nutritional plan and a good health plan. So look what is being... I noticed even going back to... just listening from a feedlot producer versus a cow calf producer and how he was finding a lot of challenges with health, bringing calves on to his feedlot, and how he would appreciate certain vaccines and certain medications being given to these animals. So I would say to that dairy producer to really look into what's a good health plan, what should I be vaccinating these calves with? That's going to set them up for the most success as they go on into the beef industry. And then just making sure that they're growing at a steady rate, seeing what people are buying calves at what weight they're buying out what they look at, and try to replicate that almost or make them look better if you can.

Researcher
So focus on the nutrition and health of those animals at the early stages of life.

Participant
Yeah.

Researcher 17:23
Okay. And my next two questions are about consumers and their perception about everything. So in general, how you think the consumers perceive beef production? And do you think this is different from the dairy production?

Participant

I think they both get a bad rep for sure. But in a different way. Like I see a lot of criticism in the dairy industry about taking the calves away from the parents and saying, oh, these dairy cows don't have any room to move. And kind of all those sorts of deals versus the beef industry. I guess it depends where you're looking in the beef industry. Like, if you're looking at more feedlot, people are saying, oh, they're crammed in, oh, they're eating this horrible food. There's just a lot of misinformation. And then in the cow calf industry, people like to see that they're on pasture and that they're in wide open spaces, you know, and kind of have all that room to move, the free-range idea. But then they don't like, oh, they're getting antibiotics. Oh, they're getting this. They're getting that. So I think I think there's kind of different criticisms based on where the animal is in the industry, for sure. But there's a lot of criticism everywhere based on the misinformation that goes around.

Researcher
Yeah, that's that makes a lot of sense. Doesn't matter what type of production there is always something that there's some criticism about? Yeah, I asked about that, because we have a lot of discussions on the dairy side about the public opinion about some practices. So for example, cow calf separation and individual housing, which are things that do not happening in beef farms. But the public has shown to disapprove those, and do you think that moving forward if this dairy beef system advances and there's a little less gap between the two industries if those practices could have any consequence to consumers views about beef production overall?

Participant
Um, I think potentially, there could be, if there is quite a group that's really critical towards the dairy industry, and then they see this gap, maybe they will potentially move their focus on to the beef industry and start to put more views there. I guess it just depends on how invested the critics are in the dairy industry. And yeah, I guess how far they want to follow it.

Researcher
Yeah. And how the information is kind of shown right too? Because as you mentioned before, there's a lot of miscommunication out there anyways.

Participant
Yes.

Researcher
Yeah. And then, I wanted to ask you specifically about cow calf separation that we talked a little bit before. Because I'm curious on your thoughts about this practice, because it's very common on dairy. And we do a lot of research to see if it is good or bad for the animals and their health overall, when there's still inconclusive results on this. And it's something that we don't do in the beef side. So I was wondering, what do you think about the practice of separating the calves from the cows soon after birth? And how do you think that influences calf health, for example?

Participant
Based on what I see in the cow calf side of things, I think that calves do better and that their health is better when they stay with their moms for longer. I mean, I see the need for it in the dairy industry.

There's a reason why it's done. If you want to get the production levels that we require in Canada, and that's demanded. But on the cow calf side, like if we have an orphan calf or something that we have to feed ourselves, you can see the difference, you know which one's drinking off the bottle and which one's drinking from mom. There's definitely further development and the calf just overall looks healthier and looks better when it's with its mom versus when it's been taken away or orphaned. So I would be interested actually, to see what dairy calves would look like and what the difference would be if they were to stay with their mom. But for sure, based on what I see just on my operation, calves do better when they are able to stay with their mom instead of going on a bottle.

Researcher
Yeah, we don't even know, as you mentioned, we all would like to know how the dairy cows would perform, because just a few farms are doing it, at least in North America. And we definitely need to do more research to see how they perform, and then how this contact should happen. But yeah, it's good to know that in the beef side, it's a very positive thing for the animals.

Participant
Yeah.

Researcher
And we talked about the genetics of those animals. So overall, the dairy beef calf will perform better than a pure dairy calf in meat production. But it's some people say it's worse than a pure beef calf on that sense. So one of the things that people are investigating now, and it's not a common practice, but it's something that they are proposing is to use embryo transfer, which would mean you could have 100% beef embryo in a dairy cow. And then this calf would be born as a beef calf and the cow would produce milk for dairy. So that animal would have the same genetics as a calf from your farm, for example. What is your opinion about this practice? And do you think there are any possible concerns about it?

Participant
Well, yeah, that's really very interesting. That's pretty cool. I mean, it sounds like a great idea really, like if that's the goal, to move your calves into the beef into the beef production and then be able to keep your dairy cows calving. I could see that being a great method for sure. I could see that being a costly method. Absolutely. But I mean, people do embryo transfers in the beef industry, too. If they have really great genetics, especially in the purebred side of things. They'll do embryo transfers, just to get siblings of the ones that they know have really performed well. And if they are top breeders, there's a lot of embryo going on. So yeah, I mean, I can see how that would work. But it's definitely a time consuming and can be expensive for sure. So, yeah, I guess there's kind of positives and negatives, but it almost would be more of a producer level positive and negative kind of effect.

Researcher
Yeah, it's definitely a costly practice, at least for now. And labor costs as well, because we need to collect those embryos and do all these procedures. So it's not something that we're going to see happening in large scale, but it's something that people are considering bringing to the table, because the issue of having those surplus dairy calves is a big one for dairy. And mainly because those are

animals that don't have any economic value or practical value for farmers. And they don't have the resources to keep them on farm either. So they're kind of in a corner. And it has happened in the past that those calves will be killed soon after birth, because they could not keep them. And this is a big ethical problem for dairy, especially if it ever reaches the media, for example, which has happened in some other countries. So that's why people are investing a lot on those dairy beef calves, because it's a way of profiting from those animals, but also using those animals in the food supply chain in some way, and not just a waste. So we're just trying to identify ways of making this practice sustainable in the future. But one of the concerns that I've heard in other interviews is if this could ever be a competition for a cow calf beef operator. For example, will people prefer to buy the calves that are coming from dairy or the calves coming from cow calf operations? I don't know if that crossed your mind, or what is your opinion about that?

Participant
Yeah. That's a good point for sure. Yeah, I mean, I guess that would make for more more calves in the market for the beef producers. So yeah, I could see how that would be a concern of raising, just adding more to the market and making it more competition for just regular cow calf producers and beef producers. I mean, at this point, as you said, it's not on a large scale, so I don't really see it being too much of a competition. But I can see how that would become a threat or a concern for beef producers. But again, it also could become a business opportunity for a few producers anyways, that are selling these embryos. So I guess some people would, some people would benefit and some people would maybe find some issues there.

Researcher
Yeah, yeah. And not only for the embryo, but also with the dairy beef calves already. They are cheaper than pure beef, even though some say that they don't have as much quality. So we see more and more of those dairy beef calves showing up in feedlots, for example. And yeah, you mentioned that may not be an issue or is not a concern for now. But let's say we have a time machine and we traveled 30 years into the future. How do you think beef production will be looking like in Canada?

Participant
Yeah, I think that it would definitely, we would see a big difference. I think there would be a lot more beef in the market, which might push beef producers out. It might kind of drown the prices a little bit. It might drive prices down if the markets too oversaturated. Which, yeah, it's hard. It's hard for a lot of beef producers to make a go of it for some time. So it might push them out, you might see less and less actual beef production as a result of the beef and dairy. So yeah, could be interesting.

Researcher
Yeah, maybe less far less beef producers but bigger in this sense if they could source those animals. Because one thing that people have also mentioned is the struggle to find animals to fill the feedlots, for example. Especially because of the drought and all of that, in Canada.

Participant

Yeah. Yeah. So I mean, in that case, that would be a positive thing. And we would be able to fill those needs with dairy and beef. But as long as it doesn't oversaturate the market, and then be a detriment to just primary beef producers.

Researcher
Yeah, definitely. And then when we talk about moving forward on this, how do you think this practice should be coordinated? For example, who should take the lead on the discussions of dairy beef production systems? Should it be the dairy? Or should it be beef, or any other organization to coordinate this conversation?

Participant
I would say it definitely shouldn't be a one-sided thing. I would say the beef and dairy need to coordinate together and talk about it and collaborate. Decide what's going to work best for both industries.

Researcher
Yeah, that makes total sense. And any other organization or industry, you think that should be at the table for these discussions?

Participant
Definitely, provincial beef organizations as well as federal. We want to see, yeah, I would like to see everybody's perspectives heard and viewed and be able to contribute to the decisions. So yeah, for sure. beef and dairy from all parts of Canada.

Researcher
Yeah, to have the local input as well in the different scenarios.

Participant 31:44
Yeah.

Researcher
Okay, nice. I covered all my questions. Thank you so much for sharing. And is there anything that you have thought about on this topic that I didn't ask you about? And you would like to share?

Participant
I don't think anything else has come to mind. Okay. This is definitely the most I've thought about this topic.

Researcher
Well, that's great. Yeah, it's already, that's information for me too. Like, how much do you think you heard about it before we met? Or? In other words, do you think that's something that is on the beef producers minds or not?

Participant

Um, it was definitely like, I've definitely heard things about it. I heard a little bit at school actually, in just a livestock production class. And then just from having friends in the dairy industry, I've heard kind of rumblings about it. But it's definitely not, not on anybody's mind right now. Nobody, none of the beef producers are really chatting about it. And nobody seems to have any concerns. You don't really hear about it, so.

Researcher
Yeah, that's a big important result for me, because as we talked, it's something that is coming, and I think it's going to only increase. And if the beef farmers are not at the table for those discussions. I don't think that's a good thing. So that's why I'm doing this research. I'm trying to put the beef farmers voices out there, and what are the concerns that they have so that it is also taken into account.

Participant
Yeah, yeah, for sure. No, that's good. I'm glad that you're doing that. I'm glad we could chat about this. Because I feel like I know more about the situation and kind of more aware of what's going on now.

Researcher
I'm glad you're happy to participate. And if anything else comes to your mind after or if there's any questions that if you like, do more research on it, and you're like, Oh, I didn't know about that. I wonder what NAME knows. And also, feel free to message me or email me anytime. And I'm happy to continue this conversation.

Participant
Yeah, absolutely.

INTERVIEW P13

[Verbal consent, introduction, and demographics were removed from the transcript to guarantee participants' anonymity]

Researcher
And my first question to you on that sense would be if you had to describe the relationship between the beef and the dairy industries in Canada, how would you describe it?

Participant
Well, okay, that's interesting. If you are talking to commercial operations, both have very large commercial operations. Dairy focuses on removing cows at birth. And beef focuses on keeping calves with the cows at birth. Really, you know, indoor versus outdoor too, because dairy is very indoor. You know, 20 years ago, if you had a bull calf, in a dairy, you had problems getting rid of it. A lot of times they couldn't find someone to buy it not even for $5 a calf, and they went ahead and killed them if it was a bull calf. Or they would sell it to the auction and nobody wanted it at the auction. It's actually pretty sad. So that's changed over the years as more people eat Holsteins. And oh, boy, that's it. That's interesting, that relationship, like, you know, there aren't a lot of people who actually eat Holsteins, but the ones who do they appreciate the fine grain. So that's a market that's changed too because even now a Holstein calf is expensive. Where it used to wasn't expensive. Nobody wanted them. And so that's changed with the price of meat, especially, it's like any cow's a good cow. That's really changed. I have cousins who have a dairy operation. And they have jerseys, because jerseys give nice cream. And they do some cross breeding with Angus, or Shorthorns, depending and sometimes with Dexters, but usually with Angus cows. And for the meat, I don't think, but they do it with heifers so that they will use a bull from another species. Just, not sure why they do it. That's a good question why? They usually sell those ones off. They aren't. You know, they're not purebred jerseys. But then a cow was ready to go, then the cows ready to join the milking line, right?

Researcher
Yes, that's exactly linked to my next question, actually. Because, as you mentioned, we hear a lot about the challenges of what to do with the male calves in the dairy industry. Because as you mentioned, they historically haven't had good meat production or value to produce beef. So one of the practices that dairy farmers are using to overcome this is to use the beef genetics in part of their cows. Because ultimately they only want the milk production out of that cow and some of the cows in their herd, they will use it for replacement, but not all of them. So what has been happening is that in the good genetic cows, they use sexed semen to make sure that they get a female calf, a good female dairy calf, out of those cows. And then in the remaining cows, they use beef semen. So they're going to have those dairy beef calves that can be Holstein-Angus, for example, is the one that we mostly see now. And then these calves have better genetics for meat production, and then consequently an increased value on the market. So they can sell those animals more easily than a pure dairy calf. And then this practice may result in more dairy supply to beef in the coming years. As you mentioned, we need more calves in the beef side as well. So there's this interest on the beef to buy more calves and the dairy side has those calves as leftovers, let's say. So how much have you heard about this before? What is your opinion about the practice of using beef genetics in dairy cows?

Participant
I don't think it makes a difference. Right now, nobody's complaining about it. They're not particularly picky whether or not your cow has a frozen ear or whether it's Simmental-Holstein cross or all beef. All cows are selling really expensive. It's ridiculous, like the prices are out of this world. They are trying to make it so people won't be able to afford to eat beef. And that's what they'll question. Right now, you can sell a bred heifer for like $3,500. These prices are crazy, right? Like people are paying $9 a pound for hamburger? That's a lot of money. And so I'm not sure that it will actually have a negative influence on the market in any way, shape, or form. And with an influx of those animals, I personally don't think it's going to affect the price of beef. Especially if it's a cross, you know, like a lot of people don't have purebred herds. Like there are lots of people who do have purebred herds, but there's still lots of raggle-taggle herds of everything and they sell just as well as a purebreds. That's what I think.

Researcher
Yes, I think one of the things that I heard before is that those calves are more affordable for beef producers to buy than pure beef calves if you compare their prices, but it's a fair point, you mentioned that all of them are already very expensive.

Participant
Yeah, they are. And well, I mean, I guess, too, you know, like that. So when you're looking at, for instance, a Jersey is, you know, it doesn't have lots and lots of meat on it. But it doesn't have a huge bone, lots of bone to it. So you know, when you mix it with something else, it's it's a very reasonable animal, whether you want to milk it or eat it, right. But if you're breeding Holstein, to other animals, like Holsteins have two qualities, which one of them is the bones are big. So as far as meat goes, I think there's less meat on a carcass on a Holstein carcass or Holstein cross carcass than there is on different breeds of cattle. Other breeds of cattle. Sorry. And if you're dealing with a bull, Holstein bulls are really mean. So they would have to be, you know, you'd have to elastrate them good and early, you know, obviously, they were not going to want to keep having them as bulls. They want them as steers, but that's another element, just that the Holstein bull is mean. So you want to have that elastrated, right?

Researcher
Yeah, I never heard that before, because we don't see many male Holsteins at all. I only see the females. I never heard of their behavior being meaner than other breeds.

Participant
Holstein females tend to be really docile. And the males are really aggressive.

Researcher
Yeah, that's good to know.

Participant
And honestly, like not many people do keep the bulls. Like if you're if you're, you know, sometimes a dairy will keep a bull. But most of the time, they'll bring in a semen. It's just easier.

Researcher
Yes, that's what I've seen mostly, I don't think I ever saw a male Holstein out here in Canada yet. I've only seen the dairy farms with the dairy cows. And maybe some of the things that you mentioned about the behavior, but also the quality of the carcass can be linked with my next question, which is if you see any challenges or open opportunities with this practice, for the beef industry?

Participant
Well, okay, so when you have Holstein, in the beef, first of all, you're gonna get more bone in the carcass, that's all there is to it. The meat itself is really, really fine grain, it's very nice meat. So you know, for somebody who's just judging it based on the meat, if they like a fine grain, it's really nice. It's tender. However, it has very little fat on it, like a Holstein does not attain fat easily. So getting the marbling, the double A, the triple A, is very difficult. So that's a challenge, you know, it affects the market that is that wants to eat it. If somebody knows what it is that they are buying, like if you want to make lean hamburger, perfect. But if you're looking at steak and whatnot, and someone wants a marbled steak, they're not going to buy anything that's got Holstein in it, if they have that option, or if they have the knowledge, the advanced knowledge of what it's what its makeup is what its genetics are.

Researcher
I see. So the genetics is a big challenge on those animals?

Participant 14:22
If you want fat in the meat, yeah, but if you want to fine grain, it's really nice. If you want more meat. It's a challenge. You know, like if you have lots of dogs and you want lots of bones, it's perfect.

Researcher
Yes, yes. Depends on what you're looking for. I see and then any opportunities that come to mind with this?

Participant
No, I think personally, I think it's like a dog. And I'll tell you what I mean by that is like, if you have a dog that's good for herding sheep, they usually aren't good dogs for retrieving birds, if you're going hunting for ducks. They're not retrievers for ducks, but they're great for herding sheep. If you have a dog, that's a guard dog for something, they're not usually great for herding sheep. Or they might be if they're a German Shepherd, you know, like, the different animals have... The reason they've been bred in a certain way is because they shine in that area. So I don't see any unique or novel opportunities, but then again, I'm not doing it. And we've had Holsteins over the years, lots of Holsteins. I've got lots of bulls, but like a bunch of calves over the years that we've gotten, you know, a couple of days old and one of the challenges with them is they often get sick and die. But, so, I mean, I like Holstein meat, but, and I've had crosses like Holstein Simmental, Angus crosses, both of those. But unique opportunities or opportunities for it? No, not really.

Researcher

I see. And you mentioned that they frequently get sick and maybe die, those animals. Do you have any suspicions of why they you see more of that in the dairy calves or dairy beef crosses than in the pure beef?

Participant
Well, for one thing, they take the calves off the mother at birth. Right? They don't even they never once get to nurse. So they get given colostrum from a bottle or a pail. It's just, you know, it's stressful. There's no nurturing. There's, you know, a lot. I personally think it's inhumane. I think it's an inhumane practice. I disagree with it. It's one of the one of the reasons that I feel reluctant to consume dairy and I love dairy. Like, I don't drink milk unless... I'm lactose intolerant, but it's something that turns me off. Because I have friends with dairy. So when I see it, it makes me sick. Like, I think I'm a mother, this is wrong. There's something wrong with this picture. You know? I think there's, you know, when you've got someone who's like, and it's way different if you've got someone who's doing. It's like, okay. I don't know how to put this. Let me formulate my thought here for one moment. So the comparison I would make to someone who is running a small, you know, milking operation with three or four cows, and producing, you know, three or four babies a year of either gender. And I would compare that to like the kind of beef production that we have, where we produce just enough beef for ourselves. When those people, they're milking their cows, they're letting the calf have, you know, half a day of milk like they're taking from the cow, but they're not taking everything. And, and it's a different kind of, like the old farmers, they milked but the cow was with the calf. And you still milk the cow, but you didn't take all the milk that calf got from every day. Because I mean, cows will produce a lot if you milk them, and the calf is sucking, they'll produce a lot of milk, they'll produce what they're asked for. And it's so it's a different kind of way of thinking. But those people, they really have an alternative mindset, just like the people like we are where we have a really small production. We know exactly who our market is, and the number one people on our market are ourselves. But same with the people who are doing the milking and they're selling or sharing milk, raw milk, homemade butters or cheeses, you know, with a very select few number of people and it's generally a very covert operation because the Dairy Council disagrees with it. The government makes it simple possible to do legally, so they do what they want to do. And under the radar.

Researcher
Yeah, it's a very challenging situation. And we also talk a lot about the cow calf separation in the dairy side, which is a practice that, in other countries like in Europe, they are already discussing how to change this. Because the consumer also doesn't like it, as you are a consumer and you don't like it, the same with me. And this is also linked with something else that I wanted to ask you because although this practice is still new, of using beef on dairy, many, many farmers are doing it and many beef farmers are buying those animals and then selling them in the market as beef, even though it's an animal that came from dairy. So one of the things that we think about is that those animals, they are sold as beef, but they are raised as dairy under dairy farming conditions. As you mentioned, the cow calf separation, but also the indoor housing and individual housing of those calves, which is very different from what beef cow calf operations do. So I was wondering, what do you think about these differences in how the animals are reared in the dairy farm and in the beef farm? And if they can influence the calf, maybe health or their success in the beef supply chain in the future?

Participant
That's a hard question, you know that? I mean, like the whole system needs an overhaul. And you know, even it's a tough conversation, even when you look at, you know, a moderate sized cow calf operation versus your feedlot type of operation. Right? So, again, you know, taking it from farming to commercial or industrial farming, that's, again, you know, that's the next level. Same with the dairy. There's different levels of dairy. I've lost my train of thought on the question. What was the question exactly as you asked it?

Researcher
Yes. So the question is what do you think about those different practices between dairy and beef in how they can influence the calf health and success in the beef supply chain in the future?

Participant
Well, I think that I personally think that they should let the calf stay with the cow for at least two weeks. And, you know, and then socialize the calves together. I mean, I realized that they create veal too, out of the dairy calves in the little igloos, right? They feed them just the milk and whatnot. And you know what? Personally, if there was a way to do the half a day, half a day, you know, where you do the half the milking for the calf and half the milking for the cow. Even to keep the calves with the cows if they were to have? Yeah, I know, I don't know what the solution is, honestly, I don't have an innovative answer. I mean, I have lots of criticisms, but I don't have an innovative answer. The ideal thing would be as if you had your own cow and you milked your own three liters a day and your cow produce the other whatever liters that the calf had, and everybody was happy. I mean, we've done that we've had a milk cow where we milked her by hand, and we had a calf on her so.

Researcher
Yeah, it's not easy and there's probably not a right... Or there's not even an answer. What we're looking with those interviews is also to see what are the beef farmers recommendation to the dairy farmers who want to sell those calves to the beef supply. So let's say if you had a friend that is a dairy farmer, and they want to start selling their surplus calves to beef and those calves are dairy beef crosses, what do you do tell your friend that they should do in their farm? To those animals that are going to the beef supply chain?

Participant
Well, you know, I would say keep... because one dairy cow can feed about three calves. Yeah, keep your old cows. Keep your old cows, you know, at least some of them who could nurse, who could actually mother a couple of calves because you'll have healthier, better growing calves. The whole bet, you know like your survival rate will be higher, your growth rate will be better You know, and maybe that's something that they could look at is keeping the old cows and using them as nurse maids.

Researcher
Yeah, that's definitely an option. And also, you have anything on the housing of those animals? Because we see that the dairy, they house animals indoors, and in beef, they go outdoors, do you think there is any challenge with that transition?

Participant
Well, if they're born in the winter, obviously is gonna be real hard for those babies to go outside, especially if they're not getting easy access to milk, right? And I think that they're thinner skinned, but what do I know? Because they don't have any fat on them. You know, a lot of dairies do have their cows outside? In the... You know, they're going in and out a lot of times. They're not going into nice fields, they're going into disgusting, shitty pens. I don't know, I don't, I don't. I mean, I think, obviously, they need to be able to be acclimatized to the outdoors, because you can't have them inside and then stick them outside and expect them to do well, with this stress. I mean, it's a very stressful transition for a calf. And there's, you know, my experience, and I mean, I've got a barn, we've got lots of straw, we have an attentive crew, because I always have lots of kids. But in my experiences, those calves arrive, and they are fragile. That's my experience. They separate them way too young and get rid of them, you know, at a day old or whatever, they're getting rid of them. They're transporting them, like they're just way too fragile.

Researcher
And that compromises when they get... Sorry?

Participant
Yeah, just that they need to not do that.

Researcher
I see. Yes. Yeah. You were just mentioning how fragile they are when they left a dairy farm compromises their health and their performance, but also survival when they get to the beef operation, right?

Participant
Oh, yeah. Pneumonia. It's terrible. Those poor things, I feel sorry for them.

Researcher
So making sure that those calves have enough maternal contact, but also nutrition and exposure, let's say to some resilience to outdoors may help them get more resilient to when they move to a beef operation, let's say.

Participant
And they need to be moved later. Not at day one, day two or day three. Yeah, like, you know, they need at least a couple of weeks to get established, you know, to have a fighting chance. That's what I think.

Researcher
Okay, yes, that that makes total sense. My next couple of questions are about consumers. So I was wondering, how do you think the consumers perceive beef production, if that differs from how they perceive dairy?

Participant

I think most consumers don't know. I think a majority of consumers are people in the city. They haven't seen a feedlot. They don't know whether the meat that they're getting has been... You know, if it had an ear tag in it with... not an ear tag, like just an identification tag, but you know, the hormone tag they don't know that. They don't know if their feed has been sprayed with antibiotics. They don't know what kind of conditions it's been raised in. They don't know about the different breeds. What they do know is that if they go to an expensive steak house, they might see that it's AAA Angus, or they may find out that they're eating Waygu but besides that, I don't think they know anything. Like honestly, I think that their most consumers are kind of I don't know if the word is naive, but they're in the dark.

Researcher
Yeah.

Participant
It's not something, like neither the beef.... You know, anything that goes through a feedlot or through the market, nobody there is ever going to say, oh hey, come and look at my feedlot, this is the greatest operation, do you want to eat your beef here? Once you look at that you're turned off. Once you see what happens inside a market like the you know the auction mart, your turn off? It's like, I don't want to be part of this. If you go through the normal kill floor, if you go through like a regular abattoir. It turns you off. Like you don't want to consume that product. That's why they don't. They are not like, hey, come for a tour of our abattoir. No. People don't, you know, people are repulsed. And that's, I mean, I guess that's partially why we do our beef the way that we do it. I'm picky. It's like the butcher comes to our house, it's an immediate kill. It's like, it's no stress, there's like, it's really, I just can't be. And don't tell anyone that you talk to after me. I can't be part of that system. And I don't want to eat that meat. Like even when I go to a restaurant, when I go to the store, I don't buy meat at the store. We raise our own chickens, we raise our own cattle. I get cheap lamb and pig from producers who are like us that raise them in the same manner. And the only thing is I don't have a milk producer. And so I do get my milk and my butter and whatnot from the store. But I'm well aware of what it goes through. And I don't like it. And you know, small meat producers. Like I love how the cows are raised out in the field and whatnot. And I'm opposed to hot branding. I'm good with freeze branding. But you know, I don't have a problem with how they're raised out in the field. My problem is, once they go to the auction mart, once you go to the abattoir, once they go to a feedlot, any of those three things, I don't like it.

Researcher
And then, that makes total sense. I'm very jealous of you. I wish I could have all those produces. Because when I was living in Brazil, we never bought meat, because we could get it from my partner's family farm. And ever since we moved to Canada is just so sad. It's not the same, even the quality of the products is not the same.

Participant
Not what you can get in the store. However, if you came to visit me, we would serve you very good beef.

Researcher

Well, I may go. With everything you're telling me, I'm tempted now. Oh, yeah, I asked about the consumers, because there's a lot of concern on the dairy side about how people would feel about some practices that are done, as you mentioned, the cow calf separation and also the early life killing of calves that cannot be used. So there's a lot of media that can be used about those practices, that would have a very negative consumer perception. And I was wondering if that could have any consequence to the beef production as well, if this dairy supply to beef increases in the future?

Participant
Well, actually it would be good advertising for beef. It's like, well, we didn't bonk this little calf on the head with the hammer. He gets to go through the other system. Right like? Yeah, that's just, it could be used as a positive. But again, you know, like there are so many legislations in place right now that stop whistleblowers from actually reporting on things that they see in factory farms. There is legislation that stops people from telling the truth of what's happening.

Researcher
Yeah. And then some consumers may not even want to know, we've seen in some research that people want to know where their food is coming from. But sometimes if they know that it's not that good, they may just close their eyes because they like the product so much. But I don't think that that's something that is going to be sustainable in the long term. I think the tendency is for people to be concerned of what happens to those animals and act on it. So that's why we do this research to see what are the alternatives and as you said, this dairy beef system is a good possible solution to the surplus dairy calves because they're not being killed soon after birth. They're contributing to the food supply chain in some way. The question is how to make it sustainable economically as well and feasible for farmers right?

Participant
Are they still being killed soon after birth, the calves? Because I mean, it's hard to find one for sale now. You used to buy them for five bucks and it was 20 bucks. Now it's 100 bucks. Now it's 250 bucks. Now it's even more than that. Like people do buy the dairy calves. To me, it's not so much... Well, they're harder to find now. People are buying them because they're a little bit cheaper, like you said, but also, I mean, it's for me, it's like, man, they just don't survive, you know, 50% or 60 or 70% survival rate isn't good enough.

Researcher
I see. Yes. That's a great point.

Participant
Well, compared to the import, right? Those little calves, they require a lot of work, and sometimes they require a lot of medicine. They get scours really badly, they get pneumonia, they get shipping fever, blah, blah, blah, blah, blah. It's a lot of work.

Researcher
Yes, there's definitely some discussion on that. Especially on the ethical side of what is worse to have the early life killing of those calves or them dying later on in life, but because of poor health conditions,

because of the standards of care that they were provided. And I don't have an answer for that. I don't know if you, what do you think about it?

Participant
I have an opinion on it, not an answer. I am not opposed to killing any animal at any time, if it's done humanely. You know, if it's done, if it's quick, and it's humane, they don't know what's coming, it's coming, it's done, they're dead. I don't have a problem with that. I have a problem with keeping an animal alive in inhumane conditions. Treating an animal inhumanely, I have a problem with that. And I have a problem with keeping animals alive, when they clearly need to be put out of their misery in a humane manner. And an owner has that obligation to do that. And I'm speaking mostly pets, right? Like, you know, people who get talked into prolonging the life of their cancer filled dog or cat. Doing this, that and the other and mortgaging their house to keep thesuffering animal life, I have an issue with that. I don't have an issue ever, with killing any animal as long as it's done humanely, quick and humanely. Because you just not going to keep every animal alive. It's just not going to happen. I mean, and there's many reasons why you put down an animal, whether it's to eat it, or it's because you can't take, you know, because it's got issues, whether it's health issues, or, you know, viciousness issues or, you know, whatever the issues are, just as long as the death is really humane. That's, I mean, I'm being pragmatic, right? Like, it's a practical thing. It's like, you're on a farm, you have 50 animals, every animal cannot live. You know, it's like, if you've got a cow that doesn't get pregnant, doesn't get pregnant, doesn't get pregnant. This is not your pet. You know, you have to look at the financial aspects of having all these animals. You're going to put that animal down. Just do it humanely, that's really what I care about. And, you know, obviously waste not, want not don't waste things, you know, use whatever you can.

Researcher
Yeah. I agree with you, I'm also a pragmatic person. So I think I have a similar view. And in the case of the surplus calves, sometimes the farmers are cornered, if they don't have anyone to buy those animals, and if they cannot have enough resources to provide for them or keep them in the farm. It's better to have the humane euthanasia than to keep those calves in poor conditions for a long time until it's too late, right?

Participant
Yeah, absolutely.

Researcher
It's just something that is hard to justify to people who are not involved right? Like for the consumers for example.

Participant
Well, you know, this is another leg of your research that could go the other way as well. What about in chicken production? They do the same thing with all those little male chicks. And you know, depending on the operations, some of them, they just grind them up live.

Researcher

Yeah, exactly.

Participant
It's disgusting. And people aren't aware of that and they buy chicken like crazy, you know, like they don't have any clue whatsoever that that's going on. I mean, some of the places they gas them, which is way more humane than, I think, than grinding them up live. But you know, it's the parallel.

Researcher
Exactly. Yeah, it's not an easy problem to tackle. But there's definitely a lot of similarities in other areas and so many things to take into account, not only the financial, but then the animal welfare and then the public opinion. So that's, that's basically my whole thesis right there.

Participant
Yeah, yeah, it's a really tough balancing act. And then, there's one other element that factors into the whole thing. And I don't know, probably not going to say this nicely. And you see it a lot with the Hutterites. You see it with people who really don't have compassion for animals. Some people just are more compassionate, how they handle animals and other people, they don't see them as living sentient beings at all. For them, they are just a financial tool, or they are a means to an end. And they don't really care. They don't care if they're mean, they don't care if they're rough, they don't care if they hurt them, they don't care. That doesn't, that doesn't matter to them. It's about money, and getting it done. And that's all they care about. You know, you've got a vast spectrum of people who are involved, you know, people who actually love their animals. And it doesn't matter what size of an operation they have, they can have hundreds of cows, they love their animals, and they'll treat them really well. And you have other people who just should never have access to an anima. Period.

Researcher
Yes. And we can see that difference, right? It's just in the big operation. Sometimes it's hard to identify the people and how they feel about the animals.

Participant
Yeah, no, that's true. And you get that in dairy, you get that in pigs, and chickens, you get that in beef, you get that in everything. Pork, like you name it. You're going to find it and you're going to find it across every religion. Every you know, every subsect of people, you're going to get nice ones, and you're going to get dirty rotters. So.

Researcher
It's not about the animals. It's about the people. That's the thing.

Participant
Yeah.

Researcher
Okay. Thank you. Yes, we're reaching our 40, past our 40 minutes mark. So in we have a meeting at noon, my noon or 1pm. So I just gonna go over the final questions. But thank you so much, just this has

been great. And I was wondering if you ever heard about embryo transfer? This is a practice that is not happening right now. But it's something that people are talking about. Because of one of the things that we talked is the genetics of dairy beef calves, they're not as good as a pure beef calf, for example. So people have been talking about using a fully beef embryo in a dairy cow. So that would mean that the calf would be pure beef, for example, a pure beef Angus would be born from a Holstein cow, the cow would produce the milk for the dairy farmer and the calf could go to the beef supply chain. Any thoughts on this practice?

Participant
Well, you know, I think those calves might be sturdier. But I really don't think I have a problem itself with the practice itself. Not having a lot of information on it. But again, you know, the calves. I don't think the calf should be separated at birth. I just think that that doesn't make for good anything. You know what, I don't know the answer. But it makes sense. It makes sense. You want to get a a Holstein on the production line. It makes sense that if you don't want to risk having... If you want to produce meat from it, that's a good way to do it. I don't know if that makes financial sense. I don't know enough about the topic to actually give you an educated answer on it.

Researcher
Yeah, that's something that I heard before about the cost of this practice not being feasible, at least not at a bigger scale. That's why it's too exploratory. But one thing I heard before as well is that it may fix the genetics issue, but those animals are still going through dairy practices. So, as you mentioned, the cow calf separation. Yeah, any comments on this?

Participant
You know, a dairy farmer does everything they can to get that calf away as soon as they can. A beef farmer will do everything they can to get the cow calf pair to work together. And they will do everything that they can, if they have... And I just had a thought about who I'm going to have you phone also. They will do everything that they can, if they have an orphaned calf, to put it with a mother who doesn't have a calf. I mean, you will phone around the countryside to put your calf with a cow who will take care of it because you know, that the very best chance that they have for survival, and for learning to be a cow is by having a good mother. Like that's the key to having a good healthy, well growing beef cow. Yeah, you know, obviously it would be the key to having a well growing healthy other calf too. But, you know, really, the efforts that you go to trying to pair up a calf that has lost its mum or, and a cow who has lost its calf. I mean, you'll take a dead calf skin, you'll cover them in, you know, all kinds of things in order to get that cow to take a calf that's not its own.

Researcher
Because it is important. And you know, it is important for that calf.

Participant
It is a primary... It is how it's going to survive.

Researcher

Definitely, definitely makes total sense. And, okay, I wish we could have all day to talk. But I feel like I need to move on to my officially last question, which is if we had a time machine, and we could travel 30 years from now in the future. How do you think that beef production will be looking like in Canada then?

Participant
Depends who, what happens in the governments around the world.

Researcher
In which sense?

Participant
If we keep going in this globalist manner that we're going now, with the focus on having people eat crickets, and being vegetarians and having fewer calories, and making the cost of everything unaffordable, I think it will be a rarity to ever get beef. And I think only the very rich people will get it, of meat of any sort. I think it will be like Cuba, where it's very hard for your regular person to get meat on a regular basis. If the globalists, you know, if the Trudeaus of the world keep controlling the world, beef will be almost non existent. You know, like, in comparison to steak would be 100 bucks. Like, you know, like if this was 30 years in the future with the same dollars, if you wanted a steak, you'd have to pay 100 bucks for it. So if that trend keeps going, which I'm hoping that we're going to have a huge rebellion around the world concerning this trend. That keeps going, you can say goodbye to beef, you can say goodbye to a lot of protein. People will become vegetarians, not because they want to but because they'll be forced to. If there's a turnaround in the politics, which is what I'm hoping and which I believe is going to happen. I mean, as the world is is revolting, right? And I hope they continue to revolt. If that's the case, then I think that we will see a different kind of consumer and a different kind of producer where our producers will maybe not feel the pressure to mass produce the same way and maybe less of a focus towards the enormous operations where... We're having a realization in people, that there's a necessity to be more self reliant. There's a necessity for farming to return to family operations. And you know, people are starting to produce more of their own food now, just in the past three years, right? Like the light bulbs going on. And so I'm hoping. And I think that if this trend continues on, you'll actually see a growth in number of people producing maybe smaller amounts. But their mind will be, I want to have more control over the way my food is produced. And I will pay for the things that I think are important.

Researcher
I do like that future. I prefer that option.

Participant
I like it too.

Researcher
But that's important to mention as well, it's not only about animal health, or all the science on that. But there's also the economic and political side of why things happen that needs to be taken into account.

Participant
Oh, yeah. In fact, a few years ago, or maybe 20 years ago? There was a real push. They were making it. The federal government here. So this actually links to Brazil, the federal government here with Pierre Trudeau. So Justin Trudeau's dad, they were actually trying to sink the Canadian beef industry. And the globalist push was that we will bring in cattle from Brazil. And their whole thought was, we're going to wean these Canadians of eating beef.

Researcher
I see.

Participant
And you know, it's gone... There are fewer and fewer people producing beef in Canada. And the ones who get to keep doing it, their operations keep getting bigger and bigger because the small producers sell out. But like I said, right about now you're starting to see the opposite, where people are saying, you know, starting to reclaim that, I better get some cows. You know, let's move back to the country. Let's get out of the city. Let's get some cows that start raising our own chickens. Let's do this, you know?

Researcher
Yeah, well, I have a lot of friends that that's their dream now. And it's also my dream, to be able to do produce my own food with my own animals. But then you try to buy a piece of land in Canada, and it's impossible.

Participant
There are ways. There are ways, you know,

Researcher
But yes, that's the mentality right now. I can see that. Okay, yes, thank you. We have two minutes. And I just want to make sure if there are anything else that I didn't ask you that you would like to share on this topic?

Participant
I think I probably shared more than you asked me.

Researcher
No, that's not even an option. There is never too much. It was a great conversation. I really appreciate it and I appreciate your time as well. What I'm going to do now I'm going to stop the recording.

INTERVIEW P29

[Verbal consent, introduction, and demographics were removed from the transcript to guarantee participants' anonymity]

Researcher
How would you describe the relationship between the beef and the dairy industry in Canada? And I think is interesting that you have experienced with both. So, I'm curious on what you have to share about that.

Participant
Yeah. How do I answer this, I guess. I mean, I guess the biggest difference in dairy farming to beef farming is like dairy farm, you have a steady income, right? So, you have a milk truck every two weeks. And whereas the farming, if you're a commercial breeder, you get like one cheque per year, if that kind of makes sense. You have kind of one shot at shipping or sending your calves to market or to, I guess, be further developed and stuff. But you basically one check. So, I know a lot of beef farmers kind of I wouldn't say they look down on dairy farming, but almost like they kind of have an unfair advantage to a certain degree. If you know what I'm saying. That'd be kind of one thing. Yeah, I don't know. Kind of a tough question to a certain degree. I know because I've been on both sides of it. So yeah, it's a tough one.

Researcher
Yeah, and there is no right or wrong answer for sure. It's like how we perceive those industries and maybe how they relate to each other or don't.

Participant
They definitely do, I guess. like they're both for food production essentially. So, I mean, they're related there. But in terms of like, they're kind of two, two completely different operations, if you know what I'm saying.

Researcher
What do you think they differ the most on and maybe what they have most in common?

Participant
Most in common? I guess we're both trying to produce a product that is at the end kind of thing. Right? And there being milk and beef production being beef, right? What we have most common would be that, I think. Yeah, I don't know. It's something to think about, that's for sure.

Researcher
Yeah, they have a similar goal. And what you mentioned before about the supply, management of dairy, this is something I heard before, too, from beef farmers, and there are different views about that. How do you think that can influence this relationship with beef? Or what you have heard about from other beef producers?

Participant
A lot of other beef producers to kind of look at the supply management or the quota system being kind of in a negative in regards to... I mean, obviously, it increases your milk prices, like compared to say, the United States where they don't have a quota system? In my opinion, though, I actually would prefer the quota system management. The reason why is it kind of... I get that it helps ensure that the product that being milked that is being produced is... It's not becoming like a factory, if you know what I'm saying? So, you're not having these massive dairy farms, like in the states where they're milking 8000 cows or whatever, right? Like, you just you don't see that up here. Right? And it kind of helps, I think, just basically create a better product.

Researcher
Yeah, it's a way of protecting the farmers in the first place, right?

Participant
Yeah.

Researcher
But I can also understand why some beef farmers are not so happy about it, because it doesn't exist in the beef side.

Participant
And I mean, I don't know if that's a good or a bad thing. It's a lot of cost too, like if you want to, for instance, myself, I'm, I guess, a younger individual in the beef industry, and I'm trying to grow so I'm trying to get more and more numbers. If you had a quota system that might make it a little bit tougher, because you would be having to buy quota, right? In order to actually gain more numbers. So that would be kind of...

Researcher
Yeah, it has, you know, ups and downs, right? There are two sides to the coin.

Participant
There are positives and negatives, definitely.

Researcher
Yeah. And then I was doing some research on how much dairy and beef have in common and how much dairy has a role in the beef production. And I found some data that about 20% of the meat production in Canada already comes from dairy in Canada and I was wondering if you think this number is true or it's higher or lower than what you would expect.

Participant
It's actually higher than I would have expected, but then to be honest with you, I know there's a lot of dairy farms starting now or their background, like they don't get paid I guess for a Holstein bull calf like we would an Angus bull calf, right? So, for them there's a lot of dairies now that I've seen, they're

starting to background and actually raised their own steers if you will, from calf to finish not actually maybe too surprised by that like I could see that, definitely. Like I could see that number being realistic.

Researcher
Yeah. And your comment about the dairy beef calves is exactly what I'm going to ask you about after this. As you already know, dairy produces more calves that they need for replacement. So, there are those surplus calves and then for many years, they were pure dairy animals that were sold for veal. But even with those calves going into production, it's not a profitable animal for the dairy farmers. So, one of the solutions that people have started using is the beef genetics on dairy. So then, the dairy cow still will produce milk, but this calf will have part of their genetics being beef, so it's a higher economic value to that animal. So yeah, just wanted to know how much about this you heard before, and what is your overall opinion about that practice?

Participant
So, I guess. Actually. my girlfriend, she's actually studying at [university’s name] right now. She's actually on their [identifiable information] team. She is one of four individuals that are chosen from [university’s name] to compete in this, they call it a [identifiable information]. So, she actually has gone to, I guess, three states now, and seen three different dairy operations down there. And then the Americans came up here to a dairy operation in [city in Canada]. So, it’s kind of a neat, like [identifiable information], you know, like those some of those bigger universities down there, too. And so, long story short, she was actually just in California here, just actually a few days ago, she just got back. A 4000-cow dairy, and they are implementing beef genetics in their dairy herd. So basically, and I have heard of that before, it was actually starting, even when I was like, when my family are still dairy farming, it was starting. One reason why was, I guess, due to Holstein bulls, essentially, were quite dangerous. So, guys would just do one cycle of AI, with sexed semen, and they just throw him in with an Angus bull. That then took the value of that, if they got a bull calf, took the value that calf from 100 bucks to maybe they got 500 for it, kind of thing. So, I mean, I can see from the dairy standpoint, why they would want to be doing that, like it was saying that this dairy in California, they really increased their, you know, how much money they're getting from their calves like doubled. I mean, it totally makes sense from their standpoint. On a beef standpoint, I don't necessarily disagree with it. Like, I think there's a couple of positives to it, I guess is that, for instance, myself being in the purebred industry, we can sell bulls to these guys that or even sell semen to some of these dairy farmers as we would get an economic benefit from it as well. Definitely, I can see where it could have an impact on. Like, there's only so many feedlots, say, in the world. And if dairy cattle are now going to be part of that, I don't know what it'll do to the beef market. It could drop them a little bit, right?

Researcher
Oh, nice. Yeah. There is any fear or I don't know, or views of it becoming a competition in some way?

Participant 12:54
Yes. Yeah. Yeah, I could definitely see that.

Researcher

Yeah, that's what I was wondering, because many of those conversations that happen with dairy farmers and on the dairy industry side, but we never asked how the beef farmers feel about it. And what is their input about that. So that's why I'm curious about your views on how you feel and maybe other beef farmers feel about the increased maybe supply from dairy to the beef in Canada?

Participant
Yeah, like, I mean, kind of, like I said, kind of mixed emotions on it. Like, I totally get it from their point of view. Looking at it from I guess a beef point of view. I mean, yeah, I could definitely have an impact, I think for sure. I don't know. It's tough to say, because in a feedlot situation, how well are those animals going to perform as well? Right, like, if they're staying in Canada, and they're going to grow them in Canada, are they going to be able to do as well in minus 35 weather in a feedlot situation as a beef animal? I'm not too sure, right? Yeah. There'd have to be a little bit more research done on it to see if it would work and then if it were to then affect the beef industry.

Researcher
Yeah, that's my exactly my next question on. Especially being someone with dairy background but working with beef now, what do you think are the main challenges and or opportunities with this practice for beef producers?

Participant
Definitely kind of like I said before, like for purebred breeders and stuff, it opens up another market for bulls. Whether that be them buying a bull for cleanup or even buying more Angus or whatever breed they want to use right. These breed to implement onto their dairy farm. It definitely opens up another market to market, you know, semen and bulls, I guess you could say right? I know there's actually dairy farms so far away. They milk, I think they're around 900 cows or so. They're actually they're all Fleckvieh Holstein cross cows. So, they've got Fleckvieh and all.

Researcher
Yes, definitely. It's a mix of maybe challenges and opportunities for the beef industry in that sense.

Participant
Yeah.

Researcher
And you mentioned before that there's probably more research that needs to be done to clarify how those animals are going to perform. And then I was wondering, what are the questions that you would like the researchers to answer maybe on that sense? What do we want to know about it?

Participant
That’s a good question.

Researcher
That's what I do. It's okay, if you need some time.

Participant
I'm trying to think how to work stuff so it kind of sounds right. I mean, I guess we need to know, from a beef standpoint, for research purposes, how are they going to perform in a feedlot scenario? Or are they going to be profitable for a feedlot scenario? Like if you're going to have to put in double the amount of feed into one of these animals to get them to say, whatever weight they need to be or finished they need to be right? They are not as profitable. Yeah, that's, that's a tough one. I would have to think about that one a little bit. For sure.

Researcher
Yeah, and I'm happy for you to text me anytime, if anything comes up the next day or the next week?

Participant
Yeah, I'll think about that one.

Researcher
Sure. Yeah. And then you mentioned you work with your beef animals. You never worked with dairy beef calves before in any way?

Participant
No, I don't. Like when in my, when we were dairy farming, we just didn't. Well, we basically did straight AI. We did a bull for cleanup. But yeah, it was just straight dairy cattle. And then we actually do have on our beef farm a couple of Holstein cows. So, we can implement them was basically nurse cows, if you will. So, have any twins or maybe a cow that's maybe struggling to, you know maybe a first-time heifer, maybe she doesn't have as much milk as what she should have or something like that. We'll use these, we have a couple of Holstein cows and then they'll we'll just put the calves on them. There is nothing wrong with them. Basically, what happened was this farm went to all robotic milking. And just the others weren't quite, the placement wasn't right for the udders. So, then we implement them, which is kind of handy. Right? So.

Researcher
Yeah, I'm curious how is their maternal behavior because well, sometimes dairy farmers say that the Holsteins are not good mothers, but they also don't have much chance of feeding their calves. So, I wonder how they do with your calves.

Participant
They seem to do good. We actually to these Holsteins now, this is actually their second year, so we actually bred them to beef bulls, okay. And they had completely black calves actually quite muscled up and stuff too. Like you would never, they're going to sell this bull and nobody's going to know that they've got Holstein in them. They look quite beef-like and both of them mothered those calves really good. And then for twins and stuff, they just normally they'll just kind of take them they just kind of stand there. Everybody goes sucks.

Researcher

They don't mind. That's good. Yeah. And it's funny you mentioned that they don't look like they are dairy beef at all because I was going to ask because some of the farmers are already raising those dairy beef calves and my question is what is the difference that they see in those animals? So, I know you only have a few but you already could not see much difference between those.

Participant
Yeah, like I if you just walked out there and you looked at him you would have you would not know that they have Holstein in them. Well, I guess these cows are half Fleckvieh, the half Holstein so they would be like a quarter Holstein, I guess.

Researcher
Yeah, I see.

Participant
No marking like they're just straight black like you would not you would have no idea.

Researcher
Do you think it's also not going to be something that shows up much in their meat production?

Participant
On these ones, I don't think but then they would essentially be a three-quarter beef animal. So maybe half beef. I know actually out of these two cows. So actually thinking about that, these two cows are actually half beef as well. Like Holstein cross. The one cow is, she's more Holstein. Like she's more not like she's built more like Holstein. The other, she definitely carries a little bit more weight. But still, she's still quite Holstein. So that's something else, like a researcher maybe should answer is maybe it's two generations away from a Holstein, right. Or a dairy animal. Until you get that ideal beef animal?

Researcher
Yeah, the proportions of.

Participant
Yeah, yeah, exactly.

Researcher
Yeah. And then, as I mentioned, some beef farmers are already buying those animals, even though I know you're not on your interest. What do you think motivates the beef farmers to purchase dairy beef calf, if you compare it to maybe a pure beef, or a pure dairy?

Participant
So I guess the beef industry you get paid by the pound. It comes down to that. Basically, why guys would be interested in buying maybe some something with a dairy influence on it, definitely as a female standpoint, would be to get maybe that added milk. A little bit extra milk, milk turns into pounds, pounds turned into money.

Researcher
Yeah, that makes sense. So in the end, it's an economic decision. What makes sense?

Participant
Yeah. 100%.

Researcher
Yeah. And one thing that I've been hearing is that the prices of calves are very high right now.

Participant
Calves, yeah. They're crazy high right now. It's like, I haven't seen them as high before.

Researcher
Yeah. So how do you think those prices can influence maybe the motivation of people to buy the dairy beef or pure beef?

Participant
It's, I mean, I don't know. It depends, I guess, on the individual. But I do know, there's one guy that I know he's up near Buderhein area. And he, he has some dairy influenced females in his herd. They maybe at the most half Holstein. But he runs them. Like I said, he does it for the added milk, the added pounds. I don't know necessarily, if the increase in beef prices would motivate a guy to then go and, you know, buy something that's half Holstein or half dairy. Right? Like, you know what I mean? Like, beef farmers are a little bit more. I guess, compared to a dairy farmer, I find the beef industry is more traditional, if you know what I'm saying. Like, they're not as motivated to change. Whereas dairy industry, the individuals that are, you know, they're on top of technology, and they're like. Whereas beef industry, it's more like, well, that's how my grandpa did it. You know what I mean.

Researcher
Yeah, I can definitely see that because we see so many new technologies being adopted on dairy. That is crazy.

Participant
Out of what gets adopted on dairy, it's like 15 years, and then the beef guys start to use it.

Researcher
Yeah. And why do you think that happens? I don't know if there's an answer, but any suspicions and why it is different in this way?

Participant
I would say, well, a lot of the dairy so there's a little bit more money in dairy farming, I guess you can say, from the outside looking in, there is, right? With that means that the younger generation probably is more willing to want to take over the farm. So maybe there's a younger group of dairy farmers, if you know what I'm saying. Beef farming where it's probably more of an older group of farmers.

Researcher
Yeah, I can definitely see that.

Participant
The younger guys, they kind of want to, they're more willing to change and they want to, you know, do better. And whereas the older generation, it's kind of like, well, that's what dad did it and we'll just keep doing it that like kind of thing. Right? It worked the, so why wouldn't it now.

Researcher
That makes sense. And maybe this is me also, not sure, but maybe having the first generation being more open to change helps things.

Participant
Exactly. Yeah. 100%.

Researcher
Yeah, and then on that note of that beef farm being more tradition based. I also see the different ways that the animals are raised in those two different industries. So, for example, in the dairy farm, the calves are separated from the mom they are in housed individually indoors while in the cow-calf beef operations, cows and calves stay together and usually on pasture. When we talk about the dairy beef calves, this is an animal that was born on dairy and will have the first early life experiences of a dairy calf. But its future life is going to be in a beef farm. So, I wonder if you think those different practices at early life can have an impact on their performance in the future?

Participant
It definitely could. If you look at, I guess, a dairy calf, just in general, probably an animal that may have a lower immunity. If you know I'm saying than a beef animal. The reason why, like you know a beef animal they're out on they're out in the world if you know what I'm saying, right? Whereas your dairy calf is kind of more. It's kind of in a little. Yeah, it's more. It's not out and about as much right? Sees a lot less kind of thing, right? The beef calf might have a little bit better immunity better, you know, just a better overall health, if you know what I'm saying.

Researcher
And why do you think so mainly for this housing outdoors or anything specific? What are the main practices that you think can influence that? Maybe the immunity that you were mentioning?

Participant
Yeah. So maybe like, I mean, that's tough, because dairy you I mean, you have to take the calf away from the cow. It's just the way it is because you're after the milk. So how you would increase maybe that immunity in a dairy calf? That's a really tough one to do.

Researcher
Yeah, I'm not sure we have a solution at all.

Participant
They're really tough.

Researcher
Yeah, that's why I'm trying to see like, based on the experiences of beef farmers, what do you identify as important? So I'm assuming that you were saying that the calf that stays with the cow has a better immunity you think?

Participant
I would just think in the beef industry, in general, I think we have a stronger immune system. Could be an example one thing just because they are, you know, they're about they're out on pastures, they're, you know, like they're seeing so many different things. So many different attributes for us. Whereas a dairy calf is very, I guess, confined, if you will, like compared to beef cows, right, like they are. They're usually in a building or you know what I mean? Like, they're, they're more, they don't get that immunity as much if you know what I mean. Yeah, so and again, how a dairy calf is raised could play and this is where the research aspect comes in too is how well they will perform in like, in a feedlot scenario.

Researcher
Yes, I can definitely see that. There are so many different things that happen in beef that don't happen in dairy that it's hard to identify which ones are the main influencers on the immunity, for example?

Participant
Yeah, yeah. No, it's, it'd be tough. It'd be, that would be probably a challenge I could see. But then again, there's dairy farms that are they've got basically feedlot scenarios, right? They're, they're feeding out their own calves and stuff. Seems to be working pretty good for them. so.

Researcher
So there is always a way to find things to work.

Participant
Exactly. Yeah.

Researcher
Okay. Yes. And then. Well, I had a question. I have a question that is a little bit more focused on possible solutions. But if it is too hard, it's okay too. Imagine you have a friend that is a dairy farmer. And they want to sell their dairy beef calves to a beef farmer. They come to you and ask what do you think I should do with those animals while they are under my care? What are the practices that dairy farmers should do?

Participant
Yeah. So big thing would be, obviously making sure that the calf that you are going to be selling to the beef producers is healthy, right? Would be free of any, you know, anything like scours or anything like that, right? That's biggest one practice that I would ask them to do. Another would be to try maybe grouping them, making sure that they can, you know, be in a group, if you know what I'm saying.

They've been kind of more individual kids or whatever, make sure that they can be grouped. But I do know, there are beef producers actually having a conversation the other day with a with a customer of ours. And he was actually kind of interested in buying, buying those kinds of calves. Holstein cross calves. So, there's definitely I think the beef market would I think they would adopt to it, personally?

Researcher
They would be interested in those animals?

Participant
Yeah, I think so.

Researcher
Okay, so guaranteeing that they're healthy. Maybe doing that by grouping them to helping their immunity, and anything else that would be important to help with keeping them healthy that you can think of?

Participant
I mean, even if they're exposed to maybe some different environmental factors too. Like beef cattle, or, I mean, they're exposed to everything from plus 30 to minus 35. So maybe if you can expose some of those calves to some of those different climate changes and stuff like that, too. Right. Like, or if you can, instead of them being undercover the whole time. And more or less, no, not babied. But you know what I mean? Like they're a little more, you know, they're, they're undercover and everything. And you can maybe expose them more of some of the environmental factors that happen that can also help, right?

Researcher
Yeah, maybe some outdoor access or something to help with the resilience.

Participant
Yeah, yeah.

Researcher
Well, my next couple of questions that are about consumers, because we have a lot of conversation on the dairy side, about public opinion about some practices. This is mainly because we live in a world with media and all of that. So, I was wondering if those conversations also happening the beef side? And how frequent they are? And whether the public perceives the dairy and the beef in the same way or differently? What are your views about that?

Participant
Yeah, I would, I would definitely say that dairy industry is probably under more scrutiny than the beef industry. A little bit more on the on that side of things. The reason being is like, the dairy industry, those cattle are handled, very, like, people are involved with those cattle every day of their life. Right? You don't see dairy cattle out on big open pastures. And you know what I mean? Like, if you're, if you're coming from, like, from, say, a city or whatever, and you're not, you know, you don't know how a beef

industry or dairy industry works. I can see how that you would view the dairy cows' lives being very poor, if you will. I'm saying that, I mean, that's, that's, that's not the case. Like, I mean, those cows are there. They've got a very strict health protocols and programs involved in and I mean, they have everything that they would ever need, right? Now, I guess implementing dairy into a beef scenario? Personally, I wouldn't really want the public to know that, to be honest with you.

Researcher
And why so?

Participant
Reason being would be I think if the public found out that dairy cattle are then being used for beef production, I just think it would I just, you're just opening up a whole other can or worms. Yeah, that's what I think personally.

Researcher
Yeah. because then you're maybe merging the problems from one side to the other, possibly?

Participant
Yeah. Yeah. And I know like, there's actually my father-in-law, he always kind of said, so there's programs like Aggie Days that run at the Calgary Stampede. Calgary Stampede themselves they have little petting zoos and stuff like that. Sometimes it's almost how much you want the public to know. Now, like there's generations where… Well, there's people who are generations removed from agriculture. It's not where my grandfather owned a farm now. There are people who think their food comes from a grocery store. And so, when they have these little events, for instance, I'll use Aggie Days, as an example. What do they bring into that event, they bring the cute little Jersey cow with the big eyes, and they bring the little piglets and the little baby chicks and everything and, and then people are like, Well, how could I eat that? You know what I mean? And they don't know the other side of it, where, you know, the animals that you're eating are not quite necessarily that friendly, if you will. Can be quite... yeah.

Researcher
Yeah. It's hard to navigate that, right?

Participant
It's tough because you want to educate the public. But then I think there's a line that needs to be drawn through on how much you educate the public, because sometimes maybe less is more, right?

Researcher
Yeah, it definitely hard, because you don't know how people are going to interpret things. Not everything's in your control. So, it's definitely hard. But I'm hearing that it's probably a problem that is more frequent on the dairy, but it's a common issue, maybe or a common concern for both industries.

Participant

And I mean, the worst case, like social media has been, I guess, something you could say, to blame for some of this, you know, people that go onto farms, and what does everyone hold in their hand? Well, they have a cell phone that's got video capabilities. And so, they'll put up a video of a cow that's maybe sick, or something, right? Then that gets up on social media. And it's like, well, this farmer is not looking after this animal. Well, you didn't know that that farmers maybe lifted that cow 10 times that day and drenched, and he's doing absolutely everything he can to save that animal. But they don't. You don't get to see that part of it. Because there's a video put up.

Researcher
Taking things out of context. Yeah.

Participant
And I mean, there's an old saying, we always go by and, you know, I mean, I love animals. That's why I do this. Like I don't, I never like to see animals that suffer or anything like that. And there's always an old saying that we always go by, and it's, you know, if you have livestock, you're going to have deadstock. And it's just the way it is. But it's reality, right? Definitely.

Researcher
Yeah. Okay, yeah. And then I mentioned, many practices that happen on dairy that don't happen on beef. One of them being the cow calf separation that we talked a little bit about. And there's a lot of research on that. There are some countries that are trying to push farmers to keep the cows and calves together. Because some research says that it's better for the animals, but it's too inconclusive. So now that I'm talking with beef producers that do keep the cows and calves together, I'm curious on their views about this practice, and how much they think it influences the animal health and welfare overall.

Participant
Yeah, so the way I look at it, I guess, growing up doing both sides of it. Beef farming, you're trying to keep your costs as low as you possibly can. So that could be one reason why you're going to be keeping the calf on the cow, right? You're going to let the calf grow on the cow. And then when that calf is of age or a weight, then you separate them, and so on and so forth. The dairy industry, the product that they're after is milk. If you have a calf that's going to be nursing on that cow and you're still going to be trying to take the milk from the cow. I don't see how that would be the most sanitary, you know, like, to me, basically dairy farm, they milk the cows, they are taking place of the calf. So maybe a dairy farm implements milking cows instead of two times a day, maybe they milk cows three times a day. Maybe kind of more, more as if a calf is nursing on that cow kind of thing, right? A calf will nurse on a cow, you know, three, four or five times a day kind of thing, right? So maybe that's something you do with the dairy industry, maybe you milk cows three times a day. And there's dairies that do that. Not necessarily, because they're thinking of it as the cow is necessarily better health wise, but maybe they get a higher amount of milk. Cow, that's about three times a day versus two times a day, right?

Researcher
Yes, their focus is on the product, which is milk.

Participant

That's what they're selling. And that's their, you know. And I have nothing against them taking the calf away from the cow, the cow doesn't know. I mean, really, the cows are so used to that they don't, they don't know any better. I remember when we were on the dairy and, and we take the calf away and the cow, she wouldn't even lick the calf off. She would just walk away. She didn't have any interest in it.

Researcher
Yeah, that's why I was curious about your experience with your cows, but they are a mix. So maybe that's what brings them some more-

Participant
I guess you could kind of say like, over natural, well not natural selection, I guess would be artificial selection. But over time, the dairy cows have kind of gotten where that mothering ability maybe isn't there as much as say in a beef cow kind of thing? For a beef cow, that calf could be born out on a 200-acre field. There's predators and you know, so she's got to have that mothering ability to keep that animal alive.

Researcher
Yeah, and it's something that beef farmers have been looking for. Right? You want a cow that is good mother that will take care of that calf, which is the opposite maybe for the dairy.

Participant
In the beef industry, if you have a heifer or a cow, who has a calf and doesn't mother it, well, that's pretty much for her sentence to become a consumable product.

Researcher
Yeah, it's not going to help.

Participant
Her job done, right?

Researcher
Yes, totally.

Participant
-against those traits, whereas in the dairy industry, it's not that they are selecting for traits of, you know, but it's just their focus is more on milk production. And, unfortunately, milk production, I guess, you could say, is a part of the calf. Like, you know, part of a calf growing is milk, right. So that selection has been, hasn't been bred out of them. It's just, they have no need to raise a calf. So, it gets essentially bred out of them, I guess you could say, right?

Researcher
It's not the priority.

Participant

Yeah.

Researcher
Okay, yeah. And then I'm curious about your views on a new practice that people are doing research on. It's not happening in many farms, but it's being discussed as a possibility. And you mentioned you have experience with embryo transfer. So, in this case, dairy farmers would use embryo transfer to put up 100% beef embryo into a dairy cow. So, this cow would get pregnant and the milk from that pregnancy would go to dairy, and the calf from that pregnancy would be a pure beef calf, that could go to the beef industry. And this is our way of overcoming the challenge of having the dairy genetics on those calves, so you have a pure beef calf. What are your thoughts about this practice?

Participant
And if they were to be doing that now, like to say all of a sudden, right now, dairy farmers trying to put in an embryo or a beef embryo into their cows, it'd be quite expensive right now for them to do that. That would be huge, huge thing. Because a lot of the guys that are doing embryo work are purebred. It's all purebred cattle, right. So those cattle are, you know, some of these embryos are worth, you know, anywhere from $1,000 to $10,000 per embryo, right? So, they're not necessarily, the embryos in the beef industry as of now are not, they're there for breeding stock, I guess you could say. They are not there for consume, like for beef production. I guess, like they're not going to enter the food chain right away.

Researcher
Yes, yes. That's why it's something that they're still doing research on because it's so expensive, that we're not even sure if it's doable on a large scale to overcome the specific issue that they want to overcome. But, yes.

Participant
It definitely, I think it could be doable. Like if a guy were just to go and pick some of your better commercial cows and dairy farmer approached you and say, hey, I'm going to pay you, I don't know, $100 per embryo or something like that, right? I could see it then being profitable for both sides of it. You flush that cow as a beef producer, and you get. So, you flush her three or four times in the year and you get 20 eggs per flush. At $100, you've turned a cow that maybe would give you $2,000 calf into giving you $4,000 worth of embryos essentially, right? So, I could see it being profitable for both and then the dairy producer and then implant those embryos. Now, that's a whole other thing too is would it work on the dairy standpoint? Like dairy cattle are under, they are under a high stress load? So, would you be able to get dairy cattle set up to accept an embryo essentially, right? Like would they be? Yeah, like it's like even some of their AI rates or AI success rates are not even that high right? Or with embryos or even that's the next step even, more than that, right? So, would they even? I don't know, it'd be tough, maybe there would have to definitely be more research done on that for sure.

Researcher
Yeah, so it's something that would be interesting if it was economically viable, and also could be done in practice on farms.

Participant
Again, like from, from my point of view, as a beef producer, where we are in the purebred industry, and we're already doing embryo work. And we're already doing all that. I actually could see an economic benefit. On my point of view, I can take up just one of my recent cows, that gives me nice calves and stuff every year, and I can flush her and like I said, turn her from maybe a $2,000 calf and maybe $4,000 for the embryos.

Researcher
I see. Yeah, definitely an opportunity there.

Participant
Yeah. Now, would the rest of the beef industry adapt to that? I'm not sure. That would be. I could definitely see a lot of a lot of the current beef producers like just, I mean, they don't even do AI, let alone embryo work. Right. So.

Researcher
You mean more based on the traditional view about?

Participant
Yeah, kind of a little bit more of a traditional viewpoint? Yeah.

Researcher
I see that. Okay. Yeah. Okay, we're on the last questions. Thank you. This has been a great conversation. We've been on the phone for a while now. I really appreciate your time. So this practice of using the beef genetics on dairy herds is something that we believe has come to stay, and it may increase in the near future. Do you think that the dairy should take the lead on those discussions about this practice? Or is it something that the beef industry could take more responsibility? How do you think those conversations would go from now on?

Participant
Dairy industry would have to take the lead on it. I think they would have to suggest the idea to the beef producers, and see how the beef producers would, I guess, convey the option of doing that kind of thing.

Researcher
And why do you think the dairy should take the lead and talk to the beef about it?

Participant
I just I don't think the beef industry as a whole would be necessarily all for it. I think there's definitely going to be those select individuals that would be, but I could see a lot of individuals getting like they just wouldn't be for the decision for doing that. Right?

Researcher
Yeah, I can see that.

Participant
If the dairy industry is pushing for it more. Yeah, maybe it would, you would get maybe some beef producers that would want to go ahead and do that. They can see the economic benefit of doing it. And then maybe if your neighbor starts doing it, then maybe you start doing it kind of thing, right?

Researcher
So, definitely the beef producers should be at a table when those discussions?

Participant
I think so. Yeah. And then and you would have to have all aspects of beef production too. You wouldn't just have a feedlot you need, you know, cow calf producers, feedlot producers, packing plants, heck, even grocery, you know, the whole food chain of beef production, I think should be there, right? Yeah. And why do you think it's important to have the different groups of beef production involved too? Every group of beef production has, I guess, a different, they would have a different viewpoint on it right. So calf producer might not be for it because it's going to affect their market. They already have kind of a closed market, right? For beef production, so I could see a lot of cow calf producers not being really that happy with it. Feedlots, I mean, they get paid to feed cattle, you know, by the pound kind of thing, right? So, or by the day, they get paid by the day. So, for them, if they're feeding, it would have to be I guess, like I said, there'd have to be the research aspect to show that, hey, dairy cattle can do as well as beef cattle in a feedlot scenario, right? Oh, if there was, then feedlot guys would probably be all for it. Right? They’re going to make the same amount of money feeding one of these animals as their beef animal. Why wouldn't they? Maybe they can buy these half dairy cattle for less than a purebred right? So, there's that and then there's the packing plants. As far as I know, actually, dairy cattle grade very well. They will grade, like a triple A and stuff like that. That's just from what I've heard. I don't know. That's what I've heard. So, I mean, then get their viewpoint and then what they think. And then the grocery store, like how if, if the public then finds out that they're being fed dairy cattle? Are they going to be all for that? Or is the public going to be like, Well, I'm not paying to eat a Holstein, I'm paying Angus beef or something like that?

Researcher
Yeah, I can definitely see why all the different pieces come with different views right to the table. Nice. Okay. And then my last question is also maybe a hard one. I'm sorry about that. There are no right or wrong answers. And then let's say we have a time machine. And would travel 30 years in the future? How do you think beef production is going to be looking like in Canada, then?

Participant
That's a tough one, for sure. I think there is going to be more adoption of technology. I know even on our operation, we're looking at doing like, for instance, in the dairy operation, I think it's [company’s name], like a tech company. They make tags where they essentially can track that cow, what that cow does every single day, how many from the amount of breaths she takes to the amount of steps she takes to a location, right? I think that actually, like, honestly, we've been looking at that for our own operation, where we kind of could see a, you know, something to benefit us would be, then we can, you know, work on our phone, and we could see if there was maybe an animal that was sick or not doing as

well. But I think definitely, like an adoption of technology will be a big thing in the next 30 years. I think there's going to be less beef producers in 30 years. Just because I mean, farming in general is in the next 30 years might change a lot, too. But I think it's just older generation, and then the younger generation not wanting to, you know, proceed anymore with agriculture. So, definitely less beef production, I would say. Bigger beef operations, compared to some small I can see all the smaller guys being essentially just selling out kind of thing. I feel like right now, price of posture and stuff is getting crazy, because guys see the calves are worth money. And so, they want to take their cut out of it. And there's got a neighbor down the road, and they're thinking of selling cows as of now because they're just getting to be too expensive. Yeah, I mean, the next 30 years could be definitely some big changes for sure.

Researcher
Yeah, there are many things that can influence that. Right. So, it's a little bit unpredictable.

Participant
The market will too. Like if the market crashes and it crashes for a few years. I mean, that's going to have a huge impact, right? If the market stays strong. Well, there's going to be more and more people getting back into cows.

Researcher
Yeah, yeah. Yeah. So, we may not know exactly how it's going to look like but we do know what can influence how, how it will be right, like, technology, the market and everything. Yeah. Okay. Perfect. Thank you. So, my those were all my questions. I don't know if there's anything that you have thought about this topic that I didn't ask you about, but you would like to share?

Participant
I don't think so. I think you covered it pretty good.

Researcher
Yeah, I did many questions. That's true.

Participant 54:52
No, I was glad to do that. Yeah, definitely. Definitely some good questions out there for sure that you brought to the table, so thank you.

INTERVIEW P31

[Verbal consent, introduction, and demographics were removed from the transcript to guarantee participants' anonymity]

Researcher
My first question for you would be, how would you describe the relationship between the beef in the dairy industry in Canada?

Participant(s)
It can be prickly at times because of the quota system and the marketing boards. And for me, personally, I don't really have any, because I've been on both sides of it. So I don't have any bad things to say about it. But I know personally, I think I've heard beef producers say that they think and maybe can hamstrung or our trade a little bit because they feel the dairy industry is a little more protectionist. Whereas us in the beef industry, we have to survive on an open market. So that's what I would say about that. So it can't like the relationship can be Prickly, but as a footballer I don't think I don't think it's bad, I don't think there's bad blood or anything but you know, there's definitely there's definitely that

Researcher
Yeah, I definitely heard about the aspect of the trade market before which is a very delicate situation because of the quota system. And to be honest, I still don't fully understand how it works. But Canada is very special on that case. I feel it is one of the last few countries that has a quota system in place. But another aspect that I've been hearing is how the animals are raised in the two systems if you have any comments on that since you already have experience with both as well.

Participant(s)
Well, we were finding more that the you know more people coming from the hall and walk the Dutch people are kind of taken over the dairies here and this in Alberta and whatnot and I think in a lot of places because and they're definitely closed housing systems like you drive by a dairy farm but you wouldn't even though it's a dairy farm. You don't see nothing outside. And I don't particularly like that I don't when we milked cows our cows when outside everyday even in the wintertime for exercise. So, and there is still people like that don't get me wrong, a lot of it are a lot of the dairies now are very confined that. I mean, that's their business and I don't think the cattle are healthy. I'm not saying that they're very well cared for very healthy, but I would rather see them I'll get a chance to go outside a little bit more. So that's my only thing I have to settle back

Researcher
And since you are two, this is the first time that I'm interviewing two people at the same time, feel free to both of you talk. I cannot see if I'm cutting someone, so feel free to stop me if I'm not hearing any cues.

Participant(s)
Yeah. Yes.

Researcher
And about the animals, it was also new to me when I came to Canada because in Brazil, most of our animals are outdoors, both beef and dairy. So it was definitely a little shock of.. I totally agree with you. They're healthy. They're having all their nutritional needs met. But it's just a different way of seeing those animals because I feel it's especially for

Participant(s)
Yeah, totally different environments from Brazil. So I understand they're asking, like, there's 30 below days where they cannot go outside I realized that, like here the other day it was 15 degrees. And when we milked cows our cows would have been outside that day. That's just how that's how we did it. But they, everything is so controlled now and their robotic milkers and everything is very regimented. And any, I think they feel any little glitch in the cows routine that affects their production. So they just they have they keep everything the same. Whether it's right or wrong, I am not arguing that. It's not how I would do it. And so that's yeah, that's what it is.

Researcher
Yeah, yeah. Yeah. And when I started to do some more research on these, what those two industries may have in common or not, I found a number of how much the dairy supplies to the beef. And it's around 20% of meat production in Canada comes from dairy. And I don't know if you've heard this number before, if you think it's a big number, or you were expecting this amount.

Participant(s)
That's kind of what I expected. Yeah. Yeah.

Researcher
Yeah. And do you think any influence what does that amount of animals that come from dairy to beef mean? Do you think to the beef industry?

Participant(s)
I don't think it means.. I don't think it's changed or will change or means a whole lot because there's always going to be bull calves that that turn into steers that are going to go into the into the veal into beef. And then they cull cows, cows that they get, they don't milk anymore. They'll they'll go into the into the hamburger chain pretty much it is what happens with them. And I don't have a problem with it, because I know they're healthy. And I know, to be perfectly honest with you, the dairy guys have a stricter standard to follow with antibiotics than what the beef, you know, they're more I shouldn't say they're stricter, but they have more. There's more supervision there because they have they have more inspectors and stuff like that. So.

Researcher
Yeah, I also heard about that before, like they those animals usually don't go to high quality cuts of meat, they're usually going to because many are cull cows, for example. So they already old animals, the meats probably..

Participant(s)

When you're buying, if you're buying an extra lean hamburger, chances are that's coming from a dairy cow because there's not much fat on them. So I would say 90% of your cull dairy cows would be lean hamburger. That would be my view on that.

Researcher
Yeah. And then you also mentioned about the male calf. So usually I have a text explaining what the surplus dairy cows are. But basically, in brief, you probably already know that the dairy herd produces more calves that they need every year. So all the male calves that won't produce milk and some of the female even maybe consider

Participant(s)
I would guess, yeah, around 20% of the females. Yeah.

Researcher
So there's a lot of surplus calves that, that's how we call them, and historically all those animals have been sent to the veal supply. But with time we are seeing a lower consumption of veal in Canada, which consequently reduced the value of those animals being sold to to veal supply. So this is going into the topic that I'm more interested about that is the dairy beef calves that are starting to show now. So basically, since the veal is not a good economic output for those animals, and consequently sometimes the surplus calves will receive less milk or colostrum in the first days of life. Or even sometimes some farmers have reported that they killed those animals soon after birth, because they cannot pay the cost of raising them. It's not worth it.

Participant(s)
Yeah

Researcher
So to try to solve this, they are starting to use beef genetics in their calves. I don't know if you've heard about this before.

Participant(s)
For sure. And you know, even when we did it, we used a very small amount of beef genetics on our on some of our Holsteins. So but there is definitely more of a going on now. Yeah, so that gives you more that's more supply of a beef dairy cross into the into the system.

Researcher
Yeah. Any thoughts on that on that practice? What is your opinion about that practice now more on based on your experience?

Participant(s)
I don't, I don't think it's wrong or anything. I mean, our numbers show that we are short or short beef. So there's guys in our business that will buy them and fatten them and it's I think it's good all around for the industry. I don't have any, any problem with it at all, actually. Because if they're if they weren't beef cross, they would be straight Holstein or straight Jersey, which are less economical for us for guys like

us to feed and convert into beef. So I'd rather I'm fine with having more beef dairy cross animals out there. It doesn't it doesn't really affect. Really, I don't think it affects our industry.

Researcher
So you mentioned you have experience with like, buying those animals in the rearing them?

Participant(s)
Oh, yeah, like we've fed beef dairy cross cattle before. They've got a little of beef on then and it just makes them just a little more hardier and, and they can withstand the elements a little better, like Holsteins, straight Holsteins and Jerseys are the two dairy breeds that are most prominent there. They're tougher to keep alive. And they will... They don't have as much try in them, let's say as a beef cross.

Researcher
And now, I think you're the first person that I've talked to that has experience with both dairy and beef, but also raising those dairy beef calves, and now asking more specifically about that. What does motivate you to purchase those animals, anything in particular?

Participant(s)
Oh, anybody that would go into that business of purchasing them would, because they know, you can still see that they're dairy. In the animals, you're buying them at a cheap, you're buying them cheaper. So your output to purchase them is less dollars. So that would be the only reason that you wouldn't want to, like less, less, less risk less upfront cost.

Researcher
I see, economically.

Participant(s)
Yeah, yeah. And having said that they won't convert and feed as good as the straight beef either, but they are, you know, that that would be the thing.

Researcher
Yeah, I was going to ask about that. Like, you probably have raised both dairy-beef calves. So animals that were raised their early days in a dairy farm, and also beef calves from a cow calf operation. What do you say, or what are your perspectives on the main differences between those animals?

Participant(s)
Between beef and dairy or?

Researcher
Yes, yes. The dairy and the dairy-beef cross.

Participant(s)

It's just more vigor, more hardiness, like, like, than anything that has dairy in them like that, you know. That was what that would be what I would say.

Researcher
Okay. Yeah, and not only the genetics are different, but also how these dairy beef calves are raised in a dairy farms. So you've probably know that they are separated soon after birth from the dam and probably raised individually or for those first days. Do you think that those practices can influence anything on the animal or maybe to the beef industry that will purchase those animals?

Participant(s)
I don't think so. Because they like I've been into these new operations and they get they get tremendous care. So I Yeah, the only thing that would, then when we get them, the only difference would be they're coming from an indoor environment to an outdoor environment. So that would be the only difference. So, you know, the catalog, the wrong fan here, when you get them that they're slick hair, they don't grow the hair, because you've been inside. So then they get they get chilled, easy. So that would be the only difference like as far as the care they get from the time they're removed from their dams. It's 100% like it's the best care sold out that park and I know they use up to date vaccinations and all that it's the only thing I would say just moving from the indoor environment to an outdoor environment

Researcher
It is adaptation period?

Participant(s)
Yeah, that's the word for it. It's adaptation right?

Researcher
Yeah, some people believe that keeping the cows and calves together may also help with the immune system of the calf. In comparison to being the bottle fed colostrum or sometimes in the dairy industry they use those powders colostrum I forgot the name in English now.

Participant(s)
Yeah, I would I would agree with you on that I like we always left our babies on the cow for 24 hours. Make sure they got the mamas colostrum and then then pull them but I do agree with you I think the mothers do nothing better than the mothers colostrum so you would get I would I would agree with that.

Researcher
Yeah, because like I'm thinking now about maybe the future of those dairy beef calves or this dairy beef system that may be starting to get in place in Canada where beef farmers like you will buy those calves from dairy and then move them up into slaughter. How do you see them, the future of this, do you think there is a place for dairy beef calves in the beef supply in the long term?

Participant(s)

Yeah, I do. I think it's perfectly fine because like I said before the numbers of the animals is changing. So either you're gonna have straight bred dairy cows or you're gonna have a handful more of crossbreds and beef dairy and I don't have any I think there's a future there. Yeah, for sure.

Researcher
And then any, any recommendations let's say if you could recommend something to a dairy farmer that wants to invest on selling their dairy-beef calves to the beef supply. What would you recommend them to do with those animals in the moment that they are under their care?

Participant(s)
They just start to maybe get them converted to being outdoors like. Once they are, you know, if the weather is especially in the summer and fall and get them outside get outside a bit before they send them to a background replace like myself or whatever, that would be the only recommendation. I know they are, as long as you're vaccinated vaccinations are up to date and all that stuff, they'll be fine.

Researcher
Yeah, and some practices that are concerns for the dairy side now are some of the cow-calf separation that I mentioned and also not allowing them outdoors or housing the calves individually which happens in most of the farms now the dairy industry is concerned about the public image of that. Do you think that it could become a concern to the beef side as well if this dairy start supplying to the beef supply?

Participant(s)
I'm not sure if it wouldn't be or not. I don't think it would be. Like we have to worry about our public perception as well. So yeah, I don't think I don't see a big concern or no.

Researcher
Yeah, I asked that because the beef way of rearing the animals is way more aligned with the public expectations so..

Participant(s)
Absolutely.

Researcher
Yeah, the animals are outside they are doing their natural behaviors and pasture staying with cow and calf together.

Participant(s)
People drive by our farm all the time in the spring when we're calving and they just love to pull over and watching the little calves out there with their moms right. And whereas, I mean they're trying hard the dairy industry like I said before you drive by dairy if you don't even know if it's a dairy farm a chicken farmer a pig farm because nothing, you don't see anything. That's one thing I do not like, I don't like that. I think almost makes it look like you're trying to hide something and I know they're not. But let's see I think people from the city like to drive around and see. See that they like to know where their food

is coming from. So like we say we've got nothing to hide. You want to drive by and have a look, go for it. We are not hiding anything. So.

Researcher
Yeah, and sometimes I question myself if the public or the general consumer when they go buy milk and beef if they even know the difference between an animal that is raised for dairy and for meat?

Participant(s)
I don't think they do.

Researcher
Yeah, that's what I was wondering if you think there could be any influence to the image of the beef industry being more well, having this a smaller line between the dairy and the beef, if the concerns of the dairy could somehow impact the beef as well.

Participant(s)
Maybe, to a certain extent, but I'm not really overly concerned about it. Because I know we have just different things like I don't know, I don't I personally, I don't think it will.

Researcher
Yeah, and especially as you said, you're open and anyone who wants to come and see your animals or old or so I feel like you're pretty open about your image on that side.

Participant(s)
Very open, like we have friends from [city name] they want to bring their kids over again, like we hoop in the truck and we go for a tour, and we show them where they are. And they like that. It's nice to see that kids need to see a calf being born and all that stuff. Like it's it's just it's it's good. It's healthy. There's there's nothing to hide. I'm sure that I know the dairy industry does that as well. And they're, they're doing it. You know, they they have open open days for the public.

Researcher
Yeah, and I definitely missed that part of me seeing the cows outside. I live now in Vancouver, the Fraser Valley. And most of the farms are dairy, so they're all inside. So I definitely miss seeing some cows outdoors.

Participant(s)
And I remember the kid going through the Fraser Valley, and it wasn't like that. Because we lost the cows outside. It's just changed so much. For the good or for the bad. I don't know. But yeah, we there's so much stuff on social media. That's not true and stuff, we really have to be vigilant and careful to make sure the wrong stuff isn't getting portrayed.

Researcher
For everyone. Both dairy and beef.

Participant(s)
Exactly. You bet for both sides. Yeah.

Researcher
I have one more or two more questions. So I can let you go soon. Another practice that is, is not happening on farm but is being developed and studied, to maybe be more widely used is embryo transfer. So in that practice, the dairy farmers would buy embryos, so 100% beef genetics, and then implant them on the dairy cows. So the dairy cow would generate a pure breed beef calf, and then go to the milk system to be milked to produce for the dairy. Any opinions on that practice?

Participant(s)
No, not really. Yeah, I've heard of it happening. But I don't really have an opinion one way or the other on it? I guess. Yeah. I don't.

Researcher
Yeah. In this in this situation, it would be mainly to overcome the issue of the dairy genetics not being as good for beef production.

Participant(s)
Yeah, but I mean, I don't see it as being a real threat. I don't see it as being anything we can be too concerned about, because it costs quite a bit of money to transfer those embryos, like we've done before. We've done embryo transfers before in the past, and that conception rate is like 40%. So if you put 100 embryos in, you're only going to get 40 calves. Yeah, and it costs it costs a lot of money. I don't think economically it's something that we ought to be concerned about.

Researcher
Yeah, I also don't see that even practically, in practice happening because there are so many challenges. And one other thing that I've already heard as is, even though you overcome the genetic side of the animal, so it's going to be a pure breed. They may still need some rearing conditions, as you mentioned before maybe going outdoors and getting enough colostrum. So maybe, I don't know, what do you think about what is more important the genetics of the animal or how the animal is raised in those early days?

Participant(s)
I think it's, I think it can be. I think it's 50/50. On both. Yeah. You're just because they have the genetics. And but if they're reared in the same environment as a dairy cow, fine. I don't I don't like that. No. So you're, basically what you said I'm in total agreement with.

Researcher
Okay. And yeah, that were the those are the questions that I have for you. And I don't know if you have anything else that I didn't ask that we didn't cover.

Participant(s)
We covered everything. And I appreciate to being able to help, you out.

INTERVIEW P33

[Verbal consent, introduction, and demographics were removed from the transcript to guarantee participants' anonymity]

Researcher
My first question to you is, how would you describe the relationship between the beef and the dairy industry in Canada?

Participant
[…] what I have found beef and dairy are very different in in Canada and BC and the main reason is I believe supply management. So, there dairy has supply management and a quota system and beef does not. And guess the other big difference between the two is that dairy is generally you sort of confined or an intensive livestock operation that the cattle are in barns. And the farms are smaller acres is, or at least the livestock area is has a smaller footprint on the land because the animals are are more in barns. I'm not sure how much I need to go into this. You're probably familiar with it. But dairy has really moved that way since I was in university with NAME. When we started, dairy cows were out on pastures and would go into barns in the evening. And now dairy cows spend most of their time in the barn and during this drier months, or I guess, the summer months, they they can get out and do a loafing area, but most of the time they're in a barn in Western Canada, these cattle spend all their time outside. And they might spend some of the time in a confined area like a feedlot if they're if they're young animals that are being prepared. were fed for slaughter. But if their child the things like the factory, the equivalent of the dairy cow on the beef side is going to be a cow that has calf and she's gonna be out in pasture or out on Crown range, graze throughout the spring, summer and fall. And then in the winter, she might spend some time in a more confined area, but it would be a feed ground like a large area with some shelter, usually trees or windbreaks. But not in a barn. When you when you get to eastern Canada, it's so the beef becomes a little more like a dairy site because in Ontario, and I think some of Quebec in the Maritimes, smaller farms. And it's more like what I would call the European model where the beef cattle are raised in barns with maybe some lounging areas or some limited pasture access during the day. more similar to the dairy but Western Canada, totally different production systems. And the two big things are the the supply management and the the intensity. I'll call it intensive versus extensive for the beef cattle, meaning barns versus large pastures.

Researcher
Yes, definitely. Those are two points that I've heard before from other interviews that I did. And I definitely have more questions about the confinement of the animals. But I'm now curious about the supply management, because many people have also discussed about that. And I'm curious on how do you perceive the role of the supply management in the dairy in this relationship with the beef industry? How do you see this influence?

Participant
Well actually, that kind of makes me think about a third thing. dairy production goes year round. And so I'm not a dairy producer, but I do have friends that are and so my understanding is that they have dairy cows that are producing calves. Is your round in our calves that the year round? freshening milk

supply? So that's another big difference. I think I should have said third point, we will. And the last question is that seasonality, I think I think you're getting dairy calves pretty much round the calendar, where beef calves again, in Western Canada come mainly in the, in the spring, April, March, April May period is like when 90% of them are going to hit the ground. And so you have this very this kind of cluster of production, where dairy is going round the calendar. And then when you get to the supply management, what my thinking is that the supply management is focused on the milk production. But it means that, again, I guess my impression, because I know dairy farms have struggled with pricing and profitability in recent times. But they have kind of a, almost a guaranteed income is how beef cattle producers perceive it. Like they have a milk board that says you produce this much milk and we'll pay you this much money as long as it doesn't have, you know, as long as it's not contaminated or some other problem, if you over produce, you get penalized if you on your produce, you might lose quota. But you know, if you can manage your productions, you're producing the, the amount of your quota and the quality is there, you're kind of guaranteed a paycheck. And in BC, we don't have that we're just printing pretty much reliant on market forces you can buy or take advantage of insurance programs if you want, like you can buy pricing insurance. But that becomes a business expense. You know, may or may not. It's all subject to market forces. And so if something happens in the market goes sideways, you are it's gonna hurt you sort of directly. And personally, I don't know that that same thing happens with dairy. It does if you have against what I gather, and like if you have contaminated milk, or if there's antibiotics or not sure what's gonna happen with the avian influenza in the milk, but you know, if something like that happens, I think you still it doesn't have the dramatic effect that it might on a beef producer where the market just suddenly disappears like that, like it did for BSE 20 years ago. And so I think that's a common understanding that beef producers have about dairy is they think oh, those guys have a guaranteed monthly or weekly paycheck, which makes it much easier to get access to capital to finance the operating that's the main thing if we go to a bank and want to borrow money to buy land or equipment or cattle, you have to have the business plan and you're saying well I'm going to produce this many this many calves and I'm going to sell them for this price. And that's really you don't have a lot to base that on. If you have a track record as a producer you've been in business for years and you can show that you know here are my here's my revenues here's here's what I get from my calves in the fall so I can prove that I know what I'm doing I'm, you know, my production practices are sound and I can raise cattle that are good quality and command a good price at the auction market or whatever. If you're able to prove that then of course that helps. But if you're new producers starting out, you don't have that track record, it's very hard to say to the bank, well, this is what I believe I can generate an income because you don't have any guarantees. So I think it makes access to capital, quite a bit more difficult for beef. Dairy, dairy has this a much more regular cash flow, and I think it's much more guaranteed probably isn't quite the right word, but it's more

Researcher
Stability maybe?

Participant
Yeah.

Researcher

Yeah. So I what I hear is that the supply manage provides the dairy more stability financially, but there's not something that happens in the beef side, which then can compromise the access to capital or as you were mentioning.

Participant
Yeah, and, and all the things that go along with that. So, yeah. Both businesses, I think, are very capital intensive, dairy has the, you know, the land values, usually dairy is located closer to urban centers. So the land values are going to be considerably higher. So if you're, you have to account for the value of your land one way or another. You know, if you're buying it, it's right there in your face. But if you own it, you have to look at your opportunity cost or maybe there's other family members involved that, you know, don't want to be in the business. So, you know, you're well aware of that your land might be worth 100,000, an acre in a dairy farm might be worth 1000, 2, or 3000, maybe 10,000 In a beef farm, just because they're usually located away from the urban centers, certainly, the Lower Mainland. So in general, beef farms are going to have a much lower land cost. Beef doesn't require the facilities ie the barns and the milking parlors. So that's a big difference. So you've got kind of a lower cost of production there because you don't have the infrastructure. Dairy has to pay for all those barns and milking parlors. Labor, I'm not sure. Labor's probably higher in dairy because you've got the daily, twice daily or three times daily task of milking, or if you're using automatic milkers, you still got to be checking all the equipment and in monitoring everything. So probably higher labor.

Researcher
Yeah, sorry.

Participant
Okay. No, I should stop for a minute, make sure I'm addressing your questions.

Researcher
Yes, you are. And I think the more we think about the two industries, more things come up in how they differ from each other. And I asked about that, because I'm interested on the aspect where they kind of overlap or merge at some point, which is the dairy supply to the beef industry. And I was doing some research on that. And I found that in Canada, about 20% of the meat production comes from dairy already. And I don't know if you How do you feel about that number if you think it's higher or lower than what you were expecting?

Participant
Well, I think the actual number is higher. Right? I think it's probably closer to 40% and that is because there is cull cows from dairy that go into the into the beef side. Hamburger. But the thing that is really starting to change the picture is the calves coming out of dairy that are going into the beef supply chain. And that is that's sort of a new and evolving thing. I know it's not brand new. I know it started it's it's been going on for a long time, but it's becoming much more refined and that the numbers are increasing. And so what I understand is happening is that because dairy producers are looking to just like any business or, you know, they want to maximize their profit they're looking for, for ways to generate revenue from the investment they have. And so these cows are producing calves all the time, but they're getting more and more efficient production is is always increasing, you rarely hear a

production decreasing. So they moved to artificial insemination long time ago, but then to semen selection and sexed semen I guess is the big thing where a dairy producer now can take his top cows, and he can breed them to you know, top of the line milking genetics and get replacement females from them and he can take his lower level, lower producing cows, and he can breed them too. Usually they are using a Black Angus, I think that's a predominant one. And but they can breed to a Black Angus, bull that and select semen for males, so they get bull calves. And on the Holsteins, those bull calves are going to come out largely black, they're going to look very much like an Angus little different frame little different years. So, you know, a knowledgeable person could say, Okay, that's a Holstein Angus cross, but lots of people can't tell the difference. But those half Angus male animals can go straight into the beef supply side. And that is why I think it's gone from the 20% to the 40% Because there's an awful lot more and more of those animals coming on every year.

So it used to be back when I started you know, 30 years ago, the competition from dairy on the beef side, nobody worried about it because it was a dairy beef was considered to be either veal calves, which were kind of a select market and called cows which would go into hamburger. So not a big deal. But where dairy can now produce these black bull or steer calves that go right into the beef side. It has a lot more impact on beef production. And I think we look at it as they're able to do things that we on the beef side can't do. I don't want to sound negative about this. I'm not saying it's a negative thing. But it's definitely a new thing and it's definitely making us think so I can really only share my thoughts I guess. So what I think is the dairy guys have an advantage because they've got the facilities, these barns and that the livestock are already in these barns where they're really easy to you know monitor and handle and everything the funds paid for on the milk side and now they can say hey, let's AI these two male beef sires, that's not the right way to put it to beef sire that can produce male tabs and that's like an extra it's a bonus for them. And in addition to the milk producing hay now because we've got all these facilities in that and the ability to handle the animals so easily, why don't we produce a bull calves for the beef side? And they can they can do that way more easily than I can because for me to go out and AI my cows. I don't have the barns I don't have the facilities to monitor them and to AI, and, you know, bring them in and out every day and synchronize these and all that, it's much more difficult for me, I just don't have the facility.

So I guess I could, if I wanted to, I could become more intensive, but then my operation would, would start to look more and more like a dairy farm. Because I would get more confined and more intensive. And it would be harder to justify because I don't have that, that that stable income from the milk checks, I'd still be in the beef market where I'm taking these animals off the auction and hoping I get a good price. Maybe I could start to do more forward contracting, or price insurance or whatever. But all of those things kind of push you from the traditional beef industry towards a production model that looks more and more like dairy, more intensive, more confined, and a more high cost. So what I really liked about the beef site, and I think in BC, we have this advantage in terms of beef production. And in fact, Western Canada, I'm not going to talk about Ontario on the Maritimes, because I'm just not as familiar with them. But I am pretty confident in my understanding of Western Canada beef production, one of the big selling points that we have is that our animals aren't raised in a confined situation, they're not raised in barns, we don't have the disease and the animal welfare issues that come with confinement. Our animals are largely raised outdoors on pasture in kind of a natural environment almost more like wildlife. You know, we feep them in the winter. And we we vaccinate them and we monitor their health.

But they're basically raised for food production out in a natural environment. They're, they're not they can't really be criticized for like factory farm animals, not all that kind of negative thing. And that has been a major selling point a marketing dynamics that I think western Canadian beef has. So our young male animals, or, or females that are going for meat production, rather than reproduction. They're going to go into a feedlot at some time, and they're going to get finished, you know, they're going to get that a high concentrate ration and, and, you know, go to slaughter. But that's going to be a period of, you know, maybe four months, one quarter to two, maybe half of their life at most more like more like the last four months on a lifespan of 20 or some 20 months, you know, so it's maybe 20% of their life they're going to spend in a feedlot the rest of time they're going to be out in a natural environment romping around and having it pretty good so I think that's a good thing. I liked that production model. The dairy animals the dairy beef, their production model is very different. They're going to they're going to those calves are going to get weaned somewhere between a day and a week of age. And they're going to go on to milk supplement for a while and then they're gonna go on to pretty good quality feed ration and they're basically feedlot animals from day one. They're gonna be in a little pen and they'll they'll never leave that sort of intensive type setting. They're there their whole life. So they can probably be more efficient at putting on the pounds. And, you know, getting them from birth to slaughter weight. I'm not sure of this, but I'm assuming that they can. Maybe take advantage of some efficiencies there because they've got more control over the animal. Our animals that are weight gain is going to be subject to a year like this very dry, winter, relatively easy winter, in terms of not much snow. So they probably gained a little more, but right now we're very worried about drought. And that's going to affect how much for it to have in front of them through the summer.

Researcher
So many things to consider

Participant
More variables and variables that you can't control. Yeah.

Researcher
Yeah, yeah. So this is great. I was actually, many of the beef producers that I've talked to don't even know about the dairy, beef calves. So you already know a lot of what is going on. And this is great, because first, not many people, not many beef producers knowing about it, I can see that as a risk. And this is why I'm doing my research to kind of give you the voice to be on the table about the future of this practice. And I think my next question on this that considering everything that you mentioned, how do you see the benefits and risks balance for the beef industry with that practice? Do you think it's more risks than benefits? And what would the benefits be?

Participant
Okay, good question. So what my friend, NAME also knows fairly well, we've we've actually done some, a little bit of experimenting. One I take, I usually take an Angus bull or two from him for the winter. And we have just sort of a trade deal. My young bulls get fed very well and live a life of luxury at the dairy farm. And they do a clean up breeding on his heifers usually. And so that's partly why I'm familiar with what's going on there. And then we've tried taking some of those beef calves, the Angus cross calves that he gets from either his AI bulls or from my bull as clean up, and I brought them back

up here to the we live in the Williams Lake area in caribou. So I brought some of those calves back up here and put them on pasture to to see how they do with with our yearlings. And it's been very, very interesting. They, they have just no understanding of life outside the pen. It's like when you put them out in the field, and they're like, Wow, where are we, you know, and the fences are new to them, the watering systems are new to them. We use a lot of electric fence, and you know, so these are all things that they've never seen, never experienced before. So it's almost comical watching them, you have to put them in a setting where they're safe and can learn what a fence is. And you know that and learn what electric fence does and how to drink out of a different water system or maybe a creek or a pond or something. So it's very funny watching them and it takes a while, maybe about a week and they kind of get things figured out. And then they kind of settle in and do okay, so you can take those dairy animals or the dairy beef, we'll call it and you can put those dairy beef calves out with the regular beef cattle like my yearlings, and they can transition and they'll do okay, but we're not sure that it's not sure that it pencils out basically. Because once you've got those animals in that intensive system, where you can control all the variables and can just put the feed in front of them that most efficiently gets them from a young animal to animal ready for slaughter. Why would you sort of derail that process and spend a bunch of extra money giving that animal a different kind of quality of life? That's really what it comes down to like to take the animal out of the, from the dairy farm down there, and Agassiz, Chilliwack, bring it up to the interior, you've got your transportation and labor costs, and then you've got those, that period of adjustment, and then probably the animal walking around on pasture, selecting its food and grazing, it's going to live a more natural life. And maybe it's and this is where NAME comes in. Probably it's welfare is going to be better. It's you know, state of mind, its happiness, when I'm not, I have my views on how you measure that I would think that animal was happier in that environment. But the games aren't going to be as efficient. So the animals is going to grow slower, you might, you might have better quality meat. From slower growing. I think these are perhaps some weird ideas. But this is kind of my experience as a as a meat inspector, that are in a confined situation, and fed a high concentrate ration for rapid growth. And let's face it, if you've got an animal in a pen, and in a confined situation, in a very aware real estate is at a premium, you're not going to want that animal to take 24 months to grow, to slaughter weight, if you can do it in 12, because your cost of housing that animal, their infrastructures were so much you just can't afford to do it. So you're going to push that animal to gain weight and get through the system as fast as you can, you can't afford to do it slowly. And when you push the animals, you end up with other issues like there's a high concentrate rations damage their organs, you know, you get liver abscesses, you get adhesions and I think if you look at a tree, a tree that grows slowly and incrementally the growth rings are tight. The wood is better quality than a tree that grows really, really rapidly. And has these really wide growth rings, I think there's some parallels there with with beef, an animal that grows too fast is going to have health issues, very likely going to have health issues. Of course, an animal that goes too slowly, this also could have health issues, they can be stunted, they there can be malnutrition issues. So I guess what I would be really interested in knowing a bit more is what is the balance between animal health and welfare? And sort of the production? If you take an animal from from birth to slaughter weighed in, in 12 monthS how does that compare to, you know, 18 months or 24 months? I don't honestly know the answer to that I have my views or my assumptions. I don't know so I'm thinking that if you asked me what one would, or what would I want to eat: an animal that has been a born out of a pasture situation and had a year of its life where it kind of wandered around with mom and lived out on pasture and romped with its fellow calves and then maybe went it got weaned and went into a feedlot for for a stretch or pastured as a yearling

weaned and pasture does yearling or backgrounded out on pasture which is what we do with a lot of the animals in BC. I'm not sure if you're familiar with this part, but kind of what happens is in the in the fall because the calves are generally born in those concentrated spring months March April May. So then in the fall, the calves are weaned October November kind of thing. And then they go into sort of a winter feed situation. If the big calves really big growthy calves, they might go straight to the feedlot and those are the ones who I say they might spend half their life outside and then half their life in the feedlot. But if they're lighter, they usually get backgrounded. And then they get turned out on pasture again in the spring. So they graze as yearlings are in New Zealand and Australia, they call them stockings I guess. And then at that 18 month period, they would go into the feedlot. So by 24 months, there, they're probably hitting the slaughter floor. That's kind of the production system that I follow. And I haven't talked to NAME about this but this is a change that my wife and I made in our operation after NAME spoke at a producer meeting here in Williams Lake. And we were, well, she was showing us some of the research that she's done with with calves and their ability to experience pain and distress and all that. And so when we sold our calves that fall, we thought, you know, this is, this is not nuts, but this is just, it's wrong. It's not what we want to do, we're just taking these babies off her mother's that six, seven months of age, and, and then just loading them on the truck, and they go to this auction market, and then they're hauled away and like what traumatic experience and we wouldn't we wouldn't treat our own kids like that why do we do that with our animals? And you know, you just go to one of the sales and you'll listen to the calves balling and the stress they are. And we thought, you know, we don't need to do this. This is just the way it's always been done. But it doesn't have to be. At the same time. We had fires and droughts in 2017 and there was a lot of extension education information out there about you know, you got to balance your, your herd size with your pasture resources and you got to identify if you have a drought or fire which animals which get rid of first and have a plan for destocking. So we came up with this idea, not at all originally unique. But if we raised less cows, and instead of selling their calves kept them over and ran them as yearlings, then that would give us the flexibility to sell those yearlings sooner or later depending on how much pasture we had available. And we wouldn't have to sell, you know, not even weaned calves when we went out to sell calves right off the cow. We could we could leave those calves on the cow. And then pasture them for a while. And you know, when we ran out of grass, we just sell.

Researcher
Yeah, that's great. And those are some practices that are very different from what happens on dairy. Right. So the way the calf is separated from the mom, soon after birth, and raised individually, and they barely see pasture as we were talking before. So on the dairy side, we have a lot of conversations that dairy farmers are concerned about the public concern about some practices like cow calf separation, and indoor housing. Those are things that don't happen on the beef industry. But if the dairy start supplying those calves to beef, this may be something that could affect the beef industry in some way. So I was just wondering your views about that.

Participant
Okay, I gotcha. I guess my bias is we give a lot of thought to animal welfare. And I think that's a good thing. I think it's the right thing to do. I also think that it has some value for marketing. And so our practice now is, we don't actually wean our calves until they're about between 10 and 11 months old, in general. And we have found that by leaving the calf with the cow, we have no health issues over the

winter. But the cow looks after her calf, they don't get sick, they don't get pneumonia, they we vaccinate. So we're not like I'm not saying we're organic or something. But, you know, we vaccinate for that we follow our vets recommendations there. We don't use everything, things that we can avoid, if we can avoid disease through improving management practices, we'll you know, we'll try and do that. But for the BVD, and BRSV, and those things we vaccinated, and so vaccinate the calves at birth and branding at weaning. So for say that the BRSV by the time they are actually weaned, they've had three vaccinations, and for most of the other diseases, they've had two, when we just leave them on the cow. They're, they're still getting milk. So the cow is teaching them how to eat the hay that we have. But they're also supplemented with milk. So we don't need to use grain or anything. And the cows teaching them how to find water, whether it's a watering system, or whether they're using natural water, which they can tell freezes. And the cow teaches them how to find shelter. So our cows are pretty happy and healthy all through the winter. And it's much less work for us. We've improved our animal health, and we've reduced our workload by not weaning those calves, which is totally contrary to what everything about beef production tells you. It says, Well, you gotta we wean your calves and feed your different age groups separately. We don't do that just feed everybody together. And we feed them really well. And then in February, we fanceline weaning, which means one day we open the gate, and we don't go through with the tractor in the feed wagon. And the cows come through the gate, because they're waiting for the feed. And the calves are a little reluctant to go through gate because it's a little more cautious and they're weak. And close the gate, cats are on one side of the electric fence cows are on the other for a day or two, the cows are kind of, you know, like I want my mom. But the mother can stand right there on the other side of the fence, and calf gets hungry. And then we put out a trough not only do they have their own hay, but then we start to give them some pellets. And we do this partly for training partly for nutrition. But by hand feeding them pellets, they, we make sure they're getting the nutrition, they need vitamins, minerals, so on. And we also train them to respond to us almost come when they're called. So then, then they can go out on pasture on their own after they're weaned. And we can sell them as they're totally preconditioning train calves ready to face the world. And, you know, they're, they're good to go. I feel much better about that system. So there's my bias. So, you know, I'm a little different than the average beef producer who's just selling calves, fresh off the cow. And I'm a lot different than the dairy producer who's got these calves taken away from their mom then raised in these little pens and understandably, they got up, they got to push them, they got to get them through the system as quick as they can because they can't afford to have them around taking 24 months to get finishing weight. Yeah, so I wouldn't say I don't approve of it. It's not my preferred system. And I guess I would, I would if I'm marketing my animals directly, which we used to do a lot more of. Now I don't, partly because it's a conflict with my ORGANIZATION job. But if I was marketing my animals directly, I'd say, hey, what would you rather eat this animal that's kind of raised naturally out on pasture, or something that's kind of locked in a jail cell and hardly sees the light of day and is force fed? You know, I could put it like that. And I would be exaggerating, but I wouldn't be totally wrong. So I guess my preference would, would be to not go for that, that the dairy beef calf. Now. I also think that if you took that dairy, beef calf, and if we could find a way to take them out of that confinement situation, and put them out on pasture and raise them in a more natural way for at least part of their life, I think that would be an improvement. But we haven't figured out the cost side of that. Yeah. What would that look like? That would mean, the dairy industry in the beef industry would have to work a lot more closely together? Because the dairy people would use would need to use the beef side to you know, background or pasture those animals? Yeah.

Researcher
Yeah, that's exactly linked with my next question. And I promise we're almost in the end. It's been almost an hour.

Participant
I'm just having fun. Don't worry.

Researcher
Okay, great. Amazing. I was concerned because I promise you 30 minutes, but it's been such a good conversation that I,

Participant
Yeah, I can't do it in 30 minutes.

Researcher
Okay, perfect. Yeah. Because my next question is that, probably those dairy, beef calves, they came to stay. Because it's something that benefits the dairy farmers as a way of finding a revenue, as you mentioned, for those calves that they have extra. So what would be your advice as a beef farmer, to a dairy farmer who wants to sell those calves to the beef supply chain? What do you think they should focus on when raising those calves?

Participant
Well, one of the things that is happening right now is a lot of dairy farms are buying ranches in the interior of the province. I think it's happening in other provinces as well. But they're moving out of the populated areas where dairy is traditionally found into the more rural areas where land values are a lot lower. And they're, they're doing that so that they can when take their replacement heifers and raised them on land that is in a less expensive facility, basically, like the ones I've seen, they bought the land out in sort of rural interior BC, much cheaper than the land in that Fraser Valley. But then they still built a barn and raise the animals in barns. And I thought, well, that's interesting, because you could you could raise that animal in a more natural setting and, and maybe that would have some overall health benefits for the animal but it just appears that they just stick with they know, we know how to raise animals and barns. So that's what we'll do even if we buy, build that land out i Sorry, if we build that barn out on cheaper land, and in the rural interior of BC, they still stick with their same production system. So they're not really they're not really cooperating or working with the beef cattle producers. They're just taking over the beef cattle producers. They're buying the buying the beef farms or the beef ranches. And they're just turning them into dairy farms and are using the dairy production model and maybe they're just using it to grow their replacements, some are using it to feed their their dairy beef calves and lower their costs. So as a beef producer I am concerned that the dairy beef is just going to wipe us out, take us over, I think it's going to happen slowly. But I think that's what's going to happen. And, and I tell you that I say that for three or four reasons, one, the province has changed and not in a particular order, but the provincial government has new water licensing requirements, and we've got obvious effects of climate change is going on. So it's getting harder and harder to run, kind of a traditional beef farm, even in the interior of British Columbia. Land values have gone up dramatically,

and the government hasn't done anything about foreign ownership. And hasn't done anything about protecting the ALR land from non farm uses. People just buying it for recreation, and they buy 160 acre farm land in the interior, for they sold their house in the Lower Mainland. And they can, they can buy a whole ranch in the interior for what they sold their house for. But they don't have any intention to rent, they just, you know, want to have a rural retreat, and they don't know anything about controlling weeds, building fences, and so they just don't, and it makes it hard for the neighbor who is trying to be a bona fide rancher. Now, like I got, I live in an area where there's weeds instead of pasture and where nobody fixes their fences, and they can't keep the cattle where they're supposed to be. And they get on a highway where these people are driving 120 kilometers an hour. And, you know, they don't know to watch for cows on the road, and they have an accident and they get sued. And so the pressure, I would say there's increasing pressure on our traditional beef farming methods, which, which make it tougher and tougher to continue. Even though I think what we're doing has a ton of environmental and animal health benefits, it's getting tougher and tougher to do it. So those dairy guys with their guaranteed paycheck, and they're huge. The equity they've got in their capital, you know, investment, they can buy a ranch and they don't have to kind of work with me, they can just take over and impose the dairy production system. And the thing is that dairy system, you might not like it, but it's efficient. They they have they figured out a really efficient system for producing beef. And I don't think it is, I think it scores very low on the animal welfare side. And I think it scores lower than our traditional beef on the animals health side. But the reality is, they can probably put out a pound of beef cheaper than I can. And I am I'm concerned that, you know, in a few years, probably be more than a few years, but in another 30 years, all the beef farms will probably look like dairy farms.

Researcher
Well, that's exactly my next question. And it's funny, you mentioned 30 years, because my next question that I have written down is 30 years from now, how do you think the beef production is gonna look like in Canada?

Participant
Okay, well, yeah. You know, I have so I have a daughter ranching in Saskatchewan and another one in Manitoba. And that it's interesting as you move from, from west to east, the Manitoba daughter and her husband, they have much bigger cattle than we do larger frame size animals. And they kind of produce big calves and sell calves. I'm not sure whether that'll change whether that kind of experience I'm having will influence them. I know it influences my daughter. Her husband has a pretty conventional approach to things but in their area, you know Large stream breeds with high production are pretty common. And there's a lot of farming and, and the cattle are kind of getting less and less. And the farming is kind of where it's at. And that's where the big money is. And so you see fences coming down or not being rebuilt and larger and larger fields. That's also happening in southern Saskatchewan, northern Saskatchewan where my vet daughter is there still more kind of cattle country because they have more of the marginal land. But they have many, many of it's amazing how many places they just don't have livestock anymore you don't see fences, because they they're not mixed farms anymore. They're large grain crop farms, or, or maybe they're a ranch, but the two don't seem to mix as much as they used to. And I think if that trend continues, you see our kind of crop farmers get larger and more intensive. And you see the the dairy farm model, which got the potential to be really efficient, beef producers, even if it's not animal welfare, Animal Health ideal. I can see that expanding, and the

traditional kind of beef production that the cattle ranch, I see that as is really kind of under threat. I am concerned I'm thinking that we're not going to be the winners, because people are going to choose.. it's a luxury to say I want to eat be from an animal that's that is kind of certified animal welfare. And I should mention that, have you heard of the verified beef program? Verified beef production Plus? Yes, I heard in other interviews. Other people mentioned that too. Okay. Have you looked into it? Like, are you familiar with that?

Researcher
No, I didn't go into more details.

Participant
Yeah. Okay, I would encourage you to look at that, because it ties to what we're talking about. I've known about the program for a long time. But one of the things that we did this winter was we went through the, the audit process. And so we pass the audit and we are now certified, as verified beef production plus producers, and there is a very large animal welfare component and animal health component in there. And so I'd encourage you to look at that. Because I can say that our animals are certified, not organic, but as verified beef production plus, and if you look at what those criteria include, it's got some good stuff in there. I don't believe that the dairy beef can pass that audit. At least right now. But I think that audit does have value at Cargill, is the big of the big plant. But there they are paying a premium for verified beef, and so is like McDonald's through the Canadian table and sustainable beef. So this is an advantage that I as a beef producer have based on our production practices is that I can say well, we're certified verify beef. I don't know that a dairy producer, a dairy beef calf producer could do that. That's now that I mentioned that to you. That's something I should look into. I would be interested to know. Got asked my auditor about that. But it comes down to what's the public going to want to pay for? Who's gonna care? Are people going to care about whether you're verified, defer or not? I hope I hope they do. And I hope that that puts some value in my production system. But just given when I look at the world, and I see that I'm very aware of the pressures that we have on our operation. And I look at what they're doing in dairy and how they're able to kind of take advantage of of some situations. And I don't begrudge them that at all. I mean, good on them. But it is there might be more opportunity for them than then there is for my beef production model, given the way things are going in this world. That's kind of how I see it. I would. Yeah.

Researcher
No, I can definitely see that. And with what you mentioned about the verified beef production, maybe there's like a niche market that the ranchers would be able to sell on. But it's definitely scary to have this kind of competition on their market. Because in the end of the day, maybe a choice between the quality and the efficiency or productivity of the system, I guess?

Participant
Yeah and not all dairy farmers. I mean, farming, ranching, raising livestock is such an individual thing. Like, I strongly feel there's science to it, but there's also an art, like, like, and this is just what I believe. But I mean, I think some people have an affinity for working with animals with livestock, and some don't. And that's a concern that I have is, is technology and automation around livestock production. The province has a BC as a big focus on technology right now. And I think, I think that's wrongheaded.

If I was the Minister of Agriculture, I would be saying, Hey, we don't need more people that can, can go around with, with iPhones and sensors, and, you know, monitors and say, Oh, this cow's temperature is high, let's stick an antibiotic into her. I think we need people that can look at animals and say, you know, this is, this is why her temperature is high. She's in a bad situation, I think we need more focus on the art part of raising livestock. And we're really not getting that we're just getting more and more focused on the science. Stick more technology at them. I don't think technology is going to be the way to technology's taking over everything. So it's going to happen anyway. I think we need somebody teaching people about the art of raising livestock and the feeling side of things. I don't think we're getting that.

Researcher
Yeah, it goes back to the tradition and how it is being threatened, and basically about the introduction of those technologies. Yeah. And NAME, I think NAME just wrote a paper on that on the automation in animal production. If you're interested, I can send that to you.

Participant
I would definitely be interested. Yeah.

Researcher
I can definitely send that.

Participant
I'd love to hear what you think. But I mean, I think that automation, and animals just pushes to factory farms. You can't have automation, when you're out there with the with the animals living in a natural environment where like wildlife, very hard to cost, effectively apply that automation. In order to apply that automation, you've got up, bring them together and confine them. And, you know, kind of stick it to them. And so it's just, I have a hard time seeing how that technology and automation takes us in in the in the direction that I feel comfortable with raising livestock.

Researcher
And then now towards my last question, I promise. And you mentioned before about the the risk of this dairy supply to beef increasing and how maybe the dairy and the beef should work more together on that aspect. So I'm wondering what would be the ideal scenario moving forward? Who should be involved in those discussions? And how should this practice move forward into the future in a way that would make you comfortable

Participant
Well, I guess I would like to do more of what I would like to try doing what my friend and I were doing where I took those calves at a young age and passed pastored them. And then in a sort of a more natural environment, and then they went into the more the, I guess, the feedlot finishing sector. So kind of work together to get a bit of the, the efficiencies and of the dairy side, and some of the sort of, let's call it the welfare health benefits of the of the beef side, at least the way my production practices go. And I mentioned that the well, the challenge there was, there's the transportation and the cost of moving the animals almost more than twice because you moved them out of the Lower Mainland, and

then they end up going back to, eventually they gotta go back to where our plant is. So there's, there's something there, that has to be worked out, it's probably economies of scale would have to do with that, because we do have.. see I think the thing could work, and maybe we just need to spend more time on it. But the obstacles are that the cost of moving the animals around. So if you're getting some economies of scale going, then maybe that could help, if the benefits of slowing their growth and giving them that more natural environment for at least part of their lives. If that had some economic value, recognize them. That would help when I go to sell those calves, they get discounted in the beef market. Because they look different, they have a little, they have a little different frame, it's usually a larger frame size, and then not as flashy. And they've got these larger years. So when I take them to sell in my market, they have, they're going to get discounted, because they're not the same as all the other beef calves. They're the odd ducklings. Now, if I could take them further, and get them closer to being finished, where the next buyer was just looking at him as a beef animal, and like, not not the best way to put it, where, where the buyer is looking at him, not for it's kinda like, How pretty is this animal or how conformation wise, you know, which is what a lot of the background is like, they're looking for an animal that is sort of their traditional beef steer or beef heifer, you know, and the dairy ones look different with the bigger frame, the years so, so they discount them, they don't want them because they're, they're not looking at them strictly as beef they're looking at these are not the kind of animals that I usually put out on pasture and I don't know if it'll do as well. And they probably know that it'll have a transition period where it learns how to live in a more natural environment, not just in its little crate. So I guess those are the reasons they get discounted, but you could remove that discount if you kept the animal long enough that it was now viewed as as just how many pounds of beef are in that animal when it's ready to go to market. It takes away all those kinds of cosmetic variables and you're just looking at the beef value.

Researcher
I see. Yes, definitely.

Participant
Yeah. So there's some things like that, that, you know, some of that I could do if, like I said, Okay, well, I guess I'm gonna give up my and these are the things by the way that I'm, my wife and I are very actively thinking. She's, she's not home this morning. So it's me doing all the talking. But you know, we're thinking, well, we're getting older. Maybe we don't want to feed cows all through the winter. But Maybe we want to go to Mexico. So maybe we should just graze animals during the grazing season, we'll take them in April and and we'll sell them in October, November, maybe even December something. And we'll just raise the stalkers and forget about the cow calf side make our life simpler. We could, we could have more work on more of the finishing side, getting those animals to finishing weight. So we would consider doing that if going back to what I said about the pressures on our, our kind of beef cattle production system just seemed to be increasing to the point where is it even worthwhile trying to raise cows here? Maybe we should give up on raising cows here in more remote places […] out in Alberta do that. And maybe we should try and sort of find a middle ground between the dairy beef and, and the finishing? And so that's, yeah, we're we're kind of thinking about that. And so if we had, like economies of scale, and we had the animals long enough, so that we could get past that bit about, oh, they, they don't look the same as all the other animals in the market. You know, maybe, maybe that would make it work. But then, on the other hand, how do I stop? I could maybe talk to my friend and

and say, Hey, let's, let's take another, let's try this again, I'm going to do it on a bigger scale, and maybe it'll be more worthwhile. But what's to stop him or his neighbor from just saying, Well, you know, forget about that. I'm just gonna buy you out. And I'll just put a big barn up there. And I'll do it myself. So I don't know, I think the only thing that could, if there was some pressure from it would have to be a societal pressure. That basically says, you know, dairy farmers, you don't have the social licence to raise those animals in box or whole life, you got to do a different. You know, unless there was something like that I I'm just not sure that over time, that that production model isn't going to take over. Yeah,

Researcher
Yeah, it is the way the production system how the production system works, and how it is kind of taking over the way beef is being produced in some way, I guess. And yeah, I was just wondering on those conversations, or basically, who is responsible for leading these discussions? Do you think it's something that the dairy industry is responsible for? Or is the does the beef industry also should take the lead in those discussions of what should be changed or not?

Participant
That's a really tough one. Because that the the dairy and the beef people that are presently in charge are you know, it's the old guard. And that's just the way the system works. It's, it's generally the old guard even if they're not the front runners, they're the ones with the power and the money behind the scenes. And I think they're more I don't know how much I don't know there's a lot of willingness or interest in cooperating. Here, I gotta tell you something that you may or may not know about, but a few years ago, the BC cattle men's association had an initiative to build a beef brand in British Columbia, it was called BC beef. And what they did was, we had an expert on the the abattoir side come in and work with us we secured an existing plant least that built the the kind of the brand did all the market research. And then we the next step was to sell shares to producers and get the sort of producer buy in and a commitment to supply and it was basically a focused on the cull cows and trying to increase the value of cows and so we started on beef and several beef producers brought it bought in but not enough to make the thing viable. So then we turned to dairy. And quite a few dairy producers also bought in, they thought, hey, this is good because you know, we don't get much for cull cows, we we know that sometimes they're all just skin and bone and nothing much to do but render them but you know some of them are, are, are better than that and have some value. And so a lot of dairy people bought in, but not enough to get the thing off the ground so that this business or brand BC beef is just sort of sitting there on the shelf all ready to go, but not quite enough buy in from producers on both sides to actually do it. So that is a I raise that because it's a positive example of where beef and dairy have worked together in the past. And it's, it's worth digging into a little bit. I'm, I mean, I have some shares in it. So and I'm still a member, NAME, I guess, the BC cattlemen association is probably the guy who could get you more info. For example, I don't know exactly how many dairy producers or how many dairy cows were committed, versus beef, but he might. But that's a positive example of where they work together. Many of the others that I'm familiar with are with where the dairy farms have bought out the beef farms. And generally, the the neighbors, the beef farmers are a little bit bitter about it, because the dairy farmers come in with all this money. And they totally changed the the nature of the production system and it leaves some some bitterness, resentment, some envy, I suppose. So I'm, I'm kind of familiar with that. And that is not a good example for moving forward. The other thing that I know

is, I'm on the NAME OF BOARD. It's called the NAME OF ORGANIZATION, [definition or organization]. When this was getting going, dairy and beef were working together, but dairy went its own way. And dairy has its own dairy traceability. And beef has its own. So right now, the beef is the Canadian cattle identification agency and dairy is what dairy trace, but when a beef animal, or sorry, when a dairy beef animal leaves the dairy side and goes into the beef side, then they have to change their tags and go into this other system. So that's kind of an example where it's an unfortunate example of where dairy and beef again couldn't work together. And this is the national level instead of having one traceability system, like we're probably going to for every other livestock, dairy has been its own way. And so I guess my experience like locally looking at real estate provincially working on the beef plant and nationally on that traceability. It's tough. dairy and beef are two very different industries, two very different cultures. And I just don't know how much potential there is. You've got to get you got to get the right people together. And, and some willingness to pay let's try kind of combining or doing things different and I'm just not, I'm not. Not exactly sure. That's a really good question. I I would actually, I would like to talk to my my friend NAME in the dairy side. And we could do some brainstorming and see but I don't have a real good answer to that right now.

Researcher
No, that's fair. And I don't even know if there is a right or like wrong answer to it either. You just what I'm hearing is that there is probably some benefits and maybe even a need of those conversations between the two industries but it's just hard to go around all the obstacles that are there between them. Yeah, and then that's That's why I'm doing this kind of research as well to see what are the opportunities? And what are these obstacles to possibly try to identify a way forward. Because, as I mentioned before, the dairy, beef calves, it's something that probably came to stay. But we don't want this to happen at the cost of any of the two industries, so trying to find a sustainable way to move forward. But yes, thank you so much for your one hour and a half. We went over all my questions. But I want to open to if there is any other thoughts that you have about this topic that I didn't ask you about?

Participant
Oh, well, I did want to say one thing. Or, in the last five years, we've gone, my wife and I have gone to both Switzerland and New Zealand. And we've kind of done agricultural tours and, and looked at this. Not not with the purpose of looking at this, but kind of came away with quite a few observations. But in Switzerland, the beef and the dairy are much more together, like you have a beef farm. And they're basically raising their beef cows the same way the dairy farm next door is raising their dairy, and they're much more integrated, and New Zealand, that Zealand a dairy is taking over everything. I mean, it's taking over. I think it's more of the takeover model. Like I was saying, I'm a bit I'm concerned that the dairy will will take over beef production and BC and New Zealand, it seems like that's what's happening. I don't know if you're familiar with New Zealand. So you may or may not have seen the same, but my observation was that the dairy farms are getting bigger, and they're they're able to do more pasture dairy. So they've quite easily been able to just sort of somebody who's just producing beef is at a disadvantage because the dairy guys can produce beef the same way he does. And they also got milk. So they kind of got two revenue streams. And he's only got one. So the the beef guy just kind of yields to the dairy. And so they're they're rotationally grazing, large dairy herds under pivot irrigation. And you know, some of them are producing milk, and some of them are going off for beef and, and even the

sheep guys look at this and say, I can't compete with just raising sheep. I'm better off, you know, selling out to the dairy guys are trying to do dairy myself. That's what we observed in a lot in New Zealand.

Researcher
Yeah, no, I never been to New Zealand. But that's very interesting. I've been to Ireland around a year ago. And they're they also have a way closer relationship between the beef and dairy. And I visited the farm from a friend that her parents did exactly what you and your wife are thinking of doing. So they had a full year fullcycle beef farm. And then they made an agreement with their neighbor who is a dairy producer. And now they just do the backgrounding and finishing off their dairy, beef calves.

Participant
You've actually seen what I was talking about.

Researcher
Yeah, as I went there, and it's just such a great system, all the calves are pasture based. They have to milk feed them for a while, because they come still before weaning. They do not have their mother cows with them. But the way that they raised those animals is just so caring and it just beautiful to see. So that's maybe definitely something that could happen in the future in Canada, depending on how things go forward. But it's possible. That's what that was nice to see.

Participant
Wow, okay, I see I'd be really interested to if you if you have pictures or, about that, I'd love to talk to you about I've not talked to you, I'd love to listen to you talk to me about that. Because that that does sound kinda like what we're thinking. And I don't, I don't know, we're trying to just kind of start it from scratch because I don't know of anybody else doing it. But you're saying in Ireland, they have at least one place doing it. Yeah. And New Zealand was very interesting because I think some of these new regulations that we Have in BC around water in that I think they're from somebody who went to New Zealand and saw the dairy farms just taking everything over. And it's really interesting talking to, I'm sure we did get a complete accurate view of things, but we get stopped and talked to some sheep farmers and they're like, ah, yeah, you know, we can still do sheep in this area, because dairy doesn't want it, but you go down there and you know, you just give up they those dairy guys, they just take over and they got so much money and all this. And then and that and we talked to some beef guys. Yeah, we just we used to just raise beef but you know, we can't compete because the dairy they got the milk and that and they can get they can get milk year round and and sell the beef and we just can't compete. So you know, we just sold to the dairy and then you get the farms, neighbors that are upset with that they're so intensive, they use all the water where they pollute all the water, and you smell manure all the time. So that dairy kind of taking over everything. Definitely seem to have had a few critics in in New Zealand. It just kind of looked around the world. And and it makes you think that what we're doing here is just going to become under more and more pressure. And I would be very interested to learn more about what what you saw in Ireland.

INTERVIEW P40

[Verbal consent, introduction, and demographics were removed from the transcript to guarantee participants' anonymity]

Researcher
So, my first question that I have for you is how would you describe the relationship between the beef and the dairy industry in Canada, and it can be both historically and maybe any changes that you may have noticed recently and why?

Participant
So, in Canada, the dairy industry itself is you know, very focused on fluid milk production. So, all activities of the industry tend to focus only around that product. But ultimately, every dairy animal becomes a beef product. So that relationship gets rather interesting. Interesting when one is very highly supply managed and the other is not. Industry wise, I think in terms of production, dairy animals, and beef animals are fundamentally the same, but have, you know, very lots of differences as well, but fundamentally, they're all beef. They're all cows. So there's lots of similarity there and, you know, health management, those kinds of things. It's the separating of the different products from the animal, but that is where it gets a little bit tricky.

Researcher
Yes. And how would you say the communication between those two industries is? Are they closely related or not at all?

Participant
I think there could be more communication and acknowledgement of the final outcome of dairy animals. It tends to be more? Well, and because of the supply management, they tend to lobby against each other's products when ultimately, they are the same product. That's kind of how I view it.

Researcher
I see. Yeah, as you mentioned, can be tricky, sometimes.

Participant
It can be very tricky, sometimes. Yes.

Researcher
And, as you mentioned, ultimately, all dairy animals will go to the beef supply in some way. And do you think this dairy supply can have an influence to the beef industry? What does that supply mean?

Participant
When it comes to cull cattle, I don't think so. It tends to be a fairly specific market in terms of grind, that kind of thing. When it comes to beef on dairy, youthful cattle. I think that there is opportunity with utilizing what traditionally was considered a cull product, so male dairy calves to fill a gap, say when the

beef cow herd declines, and we need to keep our feedlot systems and our packing systems, you know, at capacity for the system to work. I think there is an opportunity there. But I think that's where both industries need to acknowledge the other and communicate better.

Researcher
Yes, and I was looking more into data about this dairy supply to beef that already exists as, you mentioned cull cows, and maybe some of the dairy calves that go to beef. And according to the Canadian Cattle Association, about 20%, of meat production in Canada comes from the dairy industry. So you probably already knew about this number. But I've been asking people how they feel about it. And if it's a big number, is it a small number? And how maybe this number may change over the years?

Participant
Well, I mean, ultimately, beef and dairy cross will feed differently than straight beef production animal. But, you know, like I said, the feedlots need to operate at capacity. That's how their business model is built. And if there isn't enough supply of beef calves, they ultimately might need to be managed a little bit differently. But if we need the numbers, we need the numbers. And I don't think that there's a problem with that. I think it's better than having, you know, a cull product, potentially with male calves that don't go into the fluid milk industry.

Researcher
Yeah, and I think your answer is related to my next question. Thinking about this, the cull cows, but now mainly talking about the dairy beef calves. These are animals raised under dairy farming conditions and go through dairy farming practices different from how animals are raised for beef. So I'm interested in your thoughts about how these animals are raised on dairy farms and whether there are some concerns that the beef industry could have about how these animals are raised.

Participant
I think the fact that... That's a very good question to be included in your research. Because they are managed differently, at a very youthful age, they're entering potentially a feeding situation where there is a gap where they can fall out. So, the dairy industry will have, you know, its code of practice. Animals that enter the veal market will have a code of practice, but any animals that enter directly into a feeding situation, there is a gap there where the beef codes or best management practices for antimicrobials, those kinds of things. There's a gap because originally, it wasn't intended for animals that young to be entering the feeding situation. So, I do believe there is a gap. They have covered it in the States under the BQA model. And I think we need to acknowledge that in Canada and look at a standard of self-assessment, best management practices, specifically for those animals, whether they're coming up from the States or they're coming from a dairy or veal umbrella in Canada into a feeding situation or feedlot situation as youthful animals.

Researcher
Yes, and as we commented a little bit before about the communication between those two industries, and maybe we could have better communication. Regarding addressing that gap, who do you think could be the lead on this initiative and maybe who's responsible for starting those conversations?

Participant
I think it needs to be the beef industry, because you know, the beef industry is going to have to deal with a negative outcome from those cattle entering a beef feeding system. So I think it's the beef industry that should be addressing it.

Researcher
I see. And you mentioned the gap when those animals are entering the beef. I'm also interested on your views about what happens before they go to beef. So their first days of life on the dairy farm. So for example, historically, those calves are surplus. So as you mentioned before, the dairy industry is very focused on their main product, which is milk. And many dairy farmers don't see those animals as of interest to them, but also, they don't have economic value. So sometimes they don't receive the best care or the same care as the dairy calves that will stay as replacement in their herds. However, now with dairy beef, those animals are getting more economical interest. So as a beef producer, if you had a friend that is a dairy farmer and is interested in starting a dairy beef business, selling those calves to beef, what are your recommendations for that dairy farmer? What could they do with their calves to guarantee that they will have success in the beef supply chain?

Participant
I don't know necessarily. I mean, it's kind of separated into two parts because there's a little bit to unpack in that question. I don't necessarily agree with the statement that they don't get the best care, or they get different care. Knowing producers that I know, neighbors or otherwise, I don't know that they would be less cared for. I don't necessarily as a beef producer know enough about it to be able to give a recommendation. I would say, knowing cattle that going into a feeding system, the strongest, the healthiest they are, the more success they have. So practices that really are going to promote the strongest, healthiest animals going into a feeding system are going to make them successful.

Researcher
Yeah, and I mentioned the different care because we have a survey that was done a few years ago, where some dairy farmers reported giving, for example, less amount of colostrum and milk to male dairy calves on farm compared to the females. Although it's not a common practice for everyone, economically, sometimes, it's not worth it for dairy farmers to invest in animals that they cannot keep on farm. So, it also happens sometimes that those calves have been killed soon after birth. And that's why we're talking more and more about dairy beef cows, because it's a way to provide those animals with better care because they will have a better profit out of them. So, this is why I'm having those conversations, because I feel like the dairy farmers could learn a lot from the beef farmers on how we can start those conversations and prepare those animals for the beef supply. So now in general, about this practice of using beef genetics on the dairy herd, what are your thoughts about it? And how do you think the beef industry, or you personally feel about this, if this is probably an opportunity? Or does it come with some challenges to the beef industry?

Participant
I think it's an opportunity. I think that if they're going to be coming into the beef industry anyways, which if there's a gap, it's going to get filled. That's the way it works. You might as well take advantage of the feeding capacity of the beef genetics versus straight dairy genetics, which are very focused on the fluid

milk production. So I think that if that's going to happen, that the beef on dairy is absolutely an opportunity.

Researcher
Are there any challenges that you think we should be aware of? Maybe a neighbor beef farmer may not see that as positively? Or is there anything that we should be aware of in this process of starting to invest in this dairy beef system?

Participant
Well, I think there are a lot of people that believe that there are enough numbers of beef on dairy, especially coming up from the States, that they're going to depress or replace the Canadian beef cattle opportunity. I don't think that we're there yet. I think we have capacity to grow the feeding industry in Canada. But there are people that believe that their calves are going to be worth less if they're competing against a lower value, you know, cull product like beef on dairy.

Researcher
And you mentioned before about the communication to address the gap when those animals transfer from the dairy to the feeding in the beef. And maybe the beef industry should be the one starting those conversations. Do you also think that the general conversation about how dairy on beef will work in the future should also be led by beef, or dairy should take the lead or what.. My question here is how can we facilitate these conversations? Do you communicate between these two industries on this topic?

Participant
I'm not sure that it... I think the conversation is going to happen no matter who leads it. And I think that the cow calf producer on the ground, if they continue to see that their calves are bringing what they should be and there's still capacity and they're not being penalized in a competition with beef on dairy. That's going to speak louder than any industry telling them that it's not. Being honest, I think that you know, if either industry was going to say, this is a good thing, then they need to come out with some numbers to support that, whether it's the feeding industry or the beef industry in general. Say okay, you were looking at 20 percent dairy coming into our feed yards. You know, producers can see, especially if they're feedings straight Holsteins, number of Holsteins being fed. But here's what would happen if we didn't do that, we lose feedlot capacity, we lose packing capacity. That's the way I would approach that conversation. So that people, you know, it'll allay their fears without... People don't like being told things. Like, I'm telling you this, even though you don't believe it, so give them the information to believe it.

Researcher
Yes, to have the basically the research to back up on the decisions. I guess I'm on the right track then, with my research. It's good to know. And yeah, as you mentioned, the cow calf farmers, I feel like they are the ones where their system is more different from the dairy industry in Canada, at least. So for example, they keep cows and calves together for a long period of time, and many of them are grazing on pasture. While those things do not happen on dairy, and we have a lot of conversation in dairy side about the impact of those practices of separating the calves from the cow soon after birth, keeping them indoors, how they may affect animal welfare and how the public may feel about it. Do you think

that supplying those dairy animals to the beef industry, some of those concerns can also influence the beef industry or not?

Participant
Well, it would influence in a way that the beef industry is going to wear the results of that. So it's going to be the beef industry... And it's sometimes a concern on the beef side of things, that practices that dairy producers do that are solely focused on fluid milk that don't... But we wear the impacts as a cattle producer. If things are perceived negatively in the public sphere. So I do think there is a concern about that. I'm not sure what the solution is. I think, you know, if there is a you know, a set of standards or best management practices that could be verified, that tells a story or is able to say, well, I understand your perception, but here's what we're doing to allay health issues, issues of antimicrobial use standards of care. That standard of care is there. That covers the gap for those cattle.

Researcher
So […] as you mentioned, some practices that are done on dairy farms, they're very focused on milk production. But we already are seeing some push to change them. Could this supply from dairy to beef be another motivation for dairy farmers to do some practice change on their farms? One example may be being keeping cows and calves together.

Participant
I don't see that being an economic value at the dairy level. When you're focused on milk production, keeping cows and cows together. I could see a downside to that for them. I'm not sure how they would handle that. I firmly believe in the producer's right to choose what's right for their operation. You might see some of them try and do that and see what the impact is on production. But I don't know enough about it to really make a comment on it.

Researcher
Yes, yes. That makes sense, too. And as we're approaching the end of the 30 minutes that we had scheduled. I have one question about embryo transferring. I believe you've worked with that. So I'm curious on your thoughts about using embryo transfer to produce a pure beef calf in a dairy cow. So all our conversation has been about a mixed breed, between dairy and beef calves. So genetically, they're not fully made for beef production. But some people are starting to talk about using embryo transfer. I don't know what your views about this practice are, or is it another opportunity? Or does it bring concerns?

Participant
Well, I think it depends on the price point. I was at a conference a month ago, where they were talking about collecting oocytes at the slaughter plant level and producing embryos that could be implanted in dairy animals. When I was participating in IVF, the price point was definitely not there. But you know, I think that as technology evolves, and when the price point is there, the opportunity is there. That's really, the only thing I see for the future of that is what the cost is going to be. And then it's a decision the producer makes.

Researcher

Yes. And a final general question. Considering everything that we discussed today. How do you see the future of dairy and beef production in Canada, let's say 30 years from now, how do you think it is going to look like?

Participant
I think it's always going to exist, because those animals are always going to be... They're either going to be euthanized at the dairy level, if there's no value, and we keep both industries totally separate. I don't think that's very efficient or not good for anybody's public trust. I think you're going to always have a stream of animals from the dairy side coming to the beef side. And I think that the feeding industry will adjust for that. And I think that if the two industries can manage to get over or agree to disagree on differences in supply and non-supply managed industries. That it could be very copacetic relationship that benefits all of us, or if they can't, it might not. But I think there is opportunity there and acknowledging that, you know, ultimately, dairy becomes a beef issue, then, it’s the first place to start.

Researcher
Yes, yes. That makes a lot of sense. Thank you so much. And if there's anything that I didn't ask you about this topic that you would like to share, we have a few minutes for that. I don't know if there's anything that you would like to mention?

Participant
I think you've covered everything. I thought your questions were very well thought out. Made me think, that's always a good thing.

INTERVIEW P40

[Verbal consent, introduction, and demographics were removed from the transcript to guarantee participants' anonymity]

Researcher
Okay, perfect. Thank you. So now a few questions about the beef-dairy relationship in Canada. Overall, how would you describe this relationship of these two industries?

Participant
Well, I would I would describe it as a symbiotic relationship. Like I'm not sure if you're familiar with the statistics of [audio glitch] 28% [audio glitch] that are in British Columbia traditionally have come from like dairy herds into the beef cow business. Right, So about 14% of the whole cow [audio glitch]

[audio glitches and phone call ends]

Participant
[call returned] What I think what you're asking what was my understanding of dairy and beef process. So, my description was that they are, they both work together to achieve a goal of filling the requirements of the slaughter business in British Columbia.

Researcher
Okay. Yeah. And you were mentioning about the retired cow. And while the call was off, I did some research. And that's totally new to me.

Participant
Yeah, well, I think it's a better term to use. And the reason is, because a cull cow would be like you cull something out? Because they're not good. But the reality of it is a lot of cows are removed from the food chain, because they're no longer present. Right? They're not. They're not. I mean, they are. Our industry calls them a cull, but for the purposes of beef processing, they're perfectly good beep animal. Yes. Right. Okay. So that's my, my, but anyhow, that's not just something that's got really. That's really not that important of a comment. So.

Researcher
I think it's very relevant. And I think it talks about how can we do a best use of that animal on its lifetime?

Participant
Right. Yeah.

Researcher
And then going back to this, these two different dynamics of the beef and dairy production in Canada, there are some differences on how the animals are raised, for example, some practices on each side are very different. I don't know if you have any thoughts on that?

Participant
Well, you know, I mean, the reality of it is, the majority of dairy operations now are, what the term is they use a basically, they're, they're confined, they're in a confined feeding operation. So they're in barns, where they never go out. And they walk up to the milking parlor, and then they go back to their beds. And they eat and they sleep, and they never, never seen much outside time. So, you know, from a perspective of the consumer now, that would be considered a factory farm. And so when we promote beef to consumers, you know, the question becomes, do we want to be associated with factory farms? Even though we have, we have our own variety of that, but our cow herd our mother cows are not factory cows, they go out and they graze for a large percentage of the year.

Researcher
So there is a difference?

Participant
Yeah.

Researcher
And do you think this could have any influence for the beef side, in the case of this contribution of dairy to the beef supply?

Participant
I think it will have a profound impact. Because if you ever look at [identifiable information] animal welfare. But if you if you look at the cases that, where there's any kind of media coverage on cruelty to animals or whatever, then basically, almost always, it's a black and white dairy animal that's involved. Right? And so I think the point is, I guess my point is this, that if that's, you know, if you're a consumer, and you're sort of not completely familiar, well, not at all familiar with ranching, or the beef production system. You know, what, what you would take away from that, as all cattle are treated the same. And that fact is not the case, our cattle graze out and they have a much better life than other cows.

Researcher
This could be a concern, or maybe a risk in the long term.

Participant
Well, it's a marketing risk, your know. For sure. For sure it is.

Researcher
Yeah. In talking about the market now, and we mentioned that a little bit when we talked first, these two industries are also managed in different ways. Any comments?

Participant
Sorry, the market risk? The comment with a confined feeding operation as opposed to open concept? Is that what you're talking about?

Researcher
Yeah, I mean, more on the way, the sense that the dairy supply managed, while in the beef side, this is not the case, if there is any different dynamics for the marketing of those products?

Participant
No, not really. I mean, they're both marketed through the same people that use them you know, the slaughter them. You know, the dairy cattle are not as they do, they don't have as much meat on them. So there are lower yielding cattle. And so lower yielding cattle are less sought after, by, you know, meatpacking plants, because they're less efficient. But, but, you know, I don't think the average consumer understands difference between the two. Right? I mean, most people a cow is a cow is a cow. So that would be my understanding of it. You know, I don't exactly know what you're asking. But my, my comment would be, do people buy? People buy beef? Because it's supply managed? Or do they not buy beef? Because supply manage? I don't really think it enters into most people's knowledge of that.

Researcher
I also believe most people don't even know about it, I think they mostly notice the price of products on the shelf. When they go do groceries, and then they see that Canada pays a lot for dairy products, for example.

Participant
Yes. So you know, we are because we, because dairy cows are part of the beef chain. We are somewhat associated with the public's perception of subsidized cattle. Because the supply chain is a is a subsidized system, you're not allowed to buy cattle anywhere else. You know, you can't, you can't buy milk from any other country. Without without a very large tariff. You can't buy cheese from another country, you can't do any of that. So, you know, unless you're a pretty informed consumer, you may not understand the difference between the beef sector and dairy sector. Most people are not going to talk about it in the cattle business. Because you know, there's a certain there's a certain part of the industry that depends on getting dairy cattle to rebuild their requirements, slaughter cows. So you don't want to, you know, in large part, I don't think the cattle people are strongly advocate for a non use of dairy cattle in the beef sector. I don't think that's the case at all.

Researcher
I see.

Participant
I think what, what the only thing that really, at the end of the day, there's two things that probably affect the image of beef cattle. One of them is one of them is the idea that they are confined or supply manage. And the other one would be you know, the trade issues that created by having a closed market for built products.

Researcher
Yeah, yeah, I don't know if you want to expand more on the trade market aspect.

Participant
That would be my main emphasis, actually, if I was going to comment about for your studies. This is the biggest implication for the expansion of dairy cattle in this beef industry. So right now, if you're a dairy guy, you can apply the milk marketing, the Dairy farmers in Canada can apply to the marketing board for a increase in their production costs. And they do that pretty much every year. So, regardless of what happens, then the price of the commodity which is milk has to increase somewhat to cover the cost of the farmer. So that is a good thing for the supply managed people because it allows them to ask their costs on to the consumer in the beef cattle business, that is not the case, we do not have the opportunity to go to any board and say, You know what we want to 8% increase this year. So, what happens is we have traditionally had to trade cattle out of country to be able to find higher value for some of the carcasses or some of the parts of the carcass. So, what, internationally, Canada is not well respected for having a closed market system for milk products. And if you do any research at all, you'll find that the vast majority of all our trading partners do not agree with Canada's supply management system. Because they see it as a restriction. Right, they see it as restriction for them to be able to freely trade in our country. We are affected by that, because when we go to the international negotiating table, things get taken up because of the supply management system. And so we we are shut out of some trade agreements with some countries, or we have restrictions on our ability to trade because of an industry that won't allow access to the Canadian system. So it's a it's a big problem.

Researcher
That's a big consequence for your for the beef side, right?

Participant
Yes, it's a big conflict. And the reality of it is, is that while the dairy industry is important, for the beef industry in Canada, we probably have lost millions of dollars in trade globally. Because we are part of the supply managed system. You know, because our country allows supply management. So there are many free trade agreements that we are that we have taken a second, a lower position or have not got as good a deal. Because supply management is restricting factor in the trade agreement.

Researcher
Yeah.

Participant
If you make all your money selling stokes, for cooking, and you export them around the world. But you don't allow anybody to, you won't allow anybody else to sell a stove in your country that's not made in Canada. That would not work in this day and age at all. But it's very political issue. Because Quebec is a very strong political partner in Canada. They own you know, they collectively make up 25% of the vote in Parliament. And so the dairy industry is very, very focused in Quebec on keeping supply management. So those treaties, trade agreements are kind of a, for lack of a better term, they're called a sacred cow, which means they can't be touched, because otherwise you'll lose votes in Quebec. Right. So that's the problem.

Researcher

Yes, yes. And I don't know if there is an answer to this. But what love would be an alternative? Or do you see any any change in the future on this?

Participant
Well, I guess you know, what, what happens in the dairy industry, is that if, like in the case of making a free trade agreement with Europe, that the producers of the dairy industry ended up giving up 2% of their products. I think it was changed or something like that. But then the government, but then the government paid them, like a loss of revenue because of that. So the government came right out and subsidize their loss through the free trade agreement. So I'm not advocating that they government start subsidizing the beef industry, but it's a bit of a it's a bit of a contradiction that you would allow one industry to get paid because you had to make concessions on trade. But another industry, you give no consideration to on that. Right. So my, my solution would be, I guess, I don't really have a good solution. But the real ultimate solution is to do what they've done in New Zealand. And other countries like Brazil, where they just allow milk products to be sold into Canada. And the dairy guys have to compete on the basis of that.

Researcher
Yeah. Yeah. Look like, yeah. It's a little bit hard then. For them to make that happen, though, right.

Participant
Well, it's politically very unpopular. Yeah. For the dairy guys, and for some of the population in, in Canada, but, you know, I'm not too sure I wouldn't be unpopular with consumers, if they knew they were gonna be able to buy their milk for 30% less.

Researcher
Yeah, my suspicion personally, is that most people don't even know that dairy is supply managed, or what does that mean? They just know that it's expensive to buy cheese. But no one ever told them why?

Participant
Yeah, well, it's because you know, we have a we have, we have what would be considered by the rest of the world, but not a monopoly that dairy cattle have a monopoly on the market in Canada. So that is the that is the place you want to be if you're want to make money, you want to have a monopoly. So you don't have to compete with anybody except for your fellow dairy farmers.

Researcher
Yeah. o you think that has any influence on how beef and dairy farmers interact in some way? Or is totally unrelated?

Participant
No, I would say there's an interaction there, I would say there's kind of like, having difficult conversations at the Christmas dinner table. You know, like the unspoken thing is that the dairy guys understand that. The dairy guys understand that. You know, they're getting a pretty good deal. And then so they justify what they're getting and, we understand that we're not getting that good a deal. But

we also understand that they're part of our industry, and that they have more political ability to not change then we have.

Researcher
I see. Yeah, and then talking about this change. And this is something that I may be jumping ahead on my questions that I think is related to this is that what I mentioned the increased use of beef genetics in the dairy herds, and how the dairy contribution to the beef supply may be increasing over time and then how does that dynamic apply then?

Participant
Well, I mean, the dynamic applies that we will be competing against producers that are funded indirectly by government policy. So, we will be as the producer straight reproducers we will enjoy no significant benefits from that. But dairy producers who are already have a monopoly on the milk industry will be allowed to take some of our market share with their own subsidize operations. So that that will create at some point, if it goes like they're using sex semen now, and so they can produce way more bull calves, like it's no longer a 50% ratio. 50% heifers 50% bull you can, you can do 75% bull calves and 25% heifers now. And so, what what it allows you to do is use your advantage your economic advantage to do to be able to sell into a different commodities, space. Right, increase your market share, even though you are operating on a government subsidy. That would be a problem.

Researcher
And you mentioned before are a little bit on the dairy cows look black and white. But now with the beef genetics on the herd, they are more and more looking all black for example, people using Angus. So how? Yeah, how does that? How does the beef farmers feel about it and how they image of these may be influenced by those practices?

Participant
Well, I think that these guys probably believe that it is. That it's not, it's not gonna go to have a good outcome for them. I mean, how could it really? I mean, how could you? If I said to you, Researcher name], I'm gonna, I'm going to pick up black and white cows, I'm going to give you a subsidy every year of 30. Or, like, say, maybe 50% more than your beef cow neighbors. Right? And then, and I'm okay with you producing beef cattle, with your, with your subsidy so that you can compete against them? Well, you know, it's, it's just it's an unfair advantage to the dairy guys. It's no, it's not a free market system. So how did the beef guys feel about it? Well, if any of them are paying attention, they will understand that it's, it's a system that is not going to be you know, there will be little to no advantage to the cow calf or feeder producers such as myself.

Researcher
Yes, yeah.

Participant
It will actually create more competition with no compensation. You can quote me on that. [...] Well, I think that's important. catchphrase, you know, more competition with less compensation. Or no compensation.

Researcher
And yeah, my question was on any thoughts on also the quality of care of those animals, or just the way they are raised differently? So there is the aspect of the competition, but also the way the animals would be raised during their lives is different, right. As we were mentioning before.

Participant
Yeah well, they will spend their entire lives, you know, from the time they're born, until the time they are slaughtered in a confined factory operation. So I don't know about you, but most people I know, that are, have a dog or cat or like animals or have sympathy for animals. They don't view factory farms as progressive. They, they they view factory farms as something that's wrong with agriculture, not something that's right with agriculture.

Researcher
So this could be a consequence for the image of the beef industry as well, right?

Participant
Correct. It will have a negative image. Yeah.

Researcher
Now one of the main things that I think of is the cow calf separation that happens in a dairy. So in the beef, the cow and the calf stay together and graze together on the cow calf operation, but on dairy as soon as the calf is born, or a few hours after the calf is removed from the mother because she needs to be milked to produce milk for the dairy, and then the calf is raised alone with no maternal contact. And this is something that is increasingly a concern for the dairy side, and is solely the opposite of what happens in the beef. I don't know if you've heard about this practice before.

Participant
Oh, yeah, no, I mean, it's it's a known practice. Until the price of beef went up, they used to just kill the bull cows with a hammer, like, dispatch them, because they were had very little value. So they would just get rid of them, or sell them for $10 and haul them all the way to Alberta. If they did make it out alive. You know, where they would be. They made a new law a few years ago that they had to be seven days old before they can be hauled 15 hours to a feedlot. Well, you know, in terms of human things, that that would be a very good consequence. You know, increasingly, humans view animals as an extension of their own lives. You know, they feel animals should be treated similar to what humans are treated. And so the short version is that it should have, it will have and continues to have a negative image for people that are grazers that and the cow has a great life for as long as she's producing a calf. But anyhow, I'm just now we're kind of going over the same stuff. But I think that trade is an issue. The image of factory farming is an issue. You know, the animal welfare considerations are an issue. So, you know, I think those are all things that have a consequence for.

Researcher
Yeah, and I think my last topic that I would like to discuss is if there's any positive future for this. So one thing that we hear from some dairy farmers is that they don't want to raise those calves. They just want

to get at least some income out of them, but they don't want to be responsible for raising, for example, the dairy, beef cross calves. Is there any space for collaborations that you think with the farmers on this?

Participant
Well, I think there is. And you know, that's the part that we haven't talked about, because there's different sectors in the beef industry. So we're in the cow calf sector, which is, you know, basically, it is a sector where we do that we raise the cows have the calves and then raise the cows after they're weaned from the calves and all that, but if you're a feedlot person, while you're doing is another confined thing, what you're doing is you're raising in grain, and then you are selling your grain to whatever will eat it and make you the most amount of money. So the dairy cross cattle will be able to be purchased cheaper. And so as a result of that, there will be more of there will be a demand for dairy cross cattle, because they will be cheaper than purchasing beef cattle. And so for some people that just want to process the grain and they grow or the feed that they grow, it's, it's a way to process that grain. And to be able to be able to make money from the feed that they grow with having to put out less dollars per head. On average, a dairy cross animal will will probably trade for about $500 less per head. And so you know, if you beat 1000, head of cattle that's $500,000 If beat $100,000. It's a lot. It's much, much less money you need to borrow.

Researcher
Yeah. But then what would that mean for the cow calf operations then?

Participant
Well, I mean, I guess from the point of view of keeping all parts of our industry healthy. You know, we recognize that slaughter facilities have to have cattle to slaughter if they if we if the industry doesn't provide enough cattle for the slaughterhouse facilities to operate? Or the feedlots to stay full, that they have, they have to put something in them. You know, so that's, I talked about it and very beginning that there's this sort of symbiotic relationship, because we can't have we don't want more slaughter capacity leaving the country. We don't want to export our cattle to the United States to hace it slaughtered. But having said that, you know, what would if we were enjoying the same subsidies that the supply managed system has, we would probably we would out compete them without a doubt in the in the growing of beef cattle. Right, but then we would have problems exporting to the United States at that point. Because then we would get stuck into trade disputes. So I guess, circling right back to your first, your last question in the last part, what are some of the advantages of having more beef cattle in the system? I guess what it does is allows industry capacity to stay consistent. Right, so it allows the industry to be able to have enough cattle to fill up feed yards and slaughterhouses. Right? So, without those extra cattle, there would probably be less capacity for the industry in total. And that would be a negative thing for cow calf producers.

Researcher
Yeah. It's hard thing to balance all those parts, though, right?

Participant

Yeah, it's pretty hard. And I think, you know, we've got to compete with other countries in the world to like, we have to compete with Brazil, New Zealand, European beef. So we have to compete with quite a number of other countries in the beef business where the dairy guys do not compete with any other country. So it's hard to square that. But at the end of the day, here's a couple of things that are take home messages if I was going to draw a conclusion to our conversation. So the animal welfare thing is a big deal, because the consumer may or may not be well informed about which animals come from factory farms and which don't. So that's one. Second thing is the trade agreements that we have create a better, more robust pricing system for foods. So if you don't have good, free trade, the price of food is higher. And that that is in fact, what is happening in Canada with milk, the price of food in milk is higher. And so, the consumer, at the end of the day, the taxpayer, who subsidizes dairy cattle is paying more. So in this day of inflation, where people are paying more for their food, and they have higher interest rates, everything else, the losses really are to the consumer. So, you know, I think people need to think about that. And so as a consumer, if you are a consumer, you know, of beef a consumer of dairy or consumer of any of these things. You know, what is? What's the upside for you? There isn't one really. So I, I think political change would reduce the cost of food. But I'm not sure we're gonna get any political change. You know, for the purposes of your study. It's probably, it's probably negative to have more beef cattle coming through the system, from the dairy producers.

Researcher
Yeah, yeah.

Participant
Right. I don't I don't see any distinct advantages. I see. Yeah. I think there are some, but I don't think they outweigh the negative implications.

Researcher
Yeah, this is what I'm getting to conclude. From what I'm learning: there are so many different aspects of these big political and economical and practical problem. That in the dairy side is basically the excess of the animals that they have with those surplus dairy cows turning into their beef crosses. But then

Participant
I'll give you another example of an impact that is being felt in the area we have where we live from the dairy industry. So the dairy industry, basically, if you're a good dairy producer, you will grow your gross receipts will be $10,000 per cow per year. That's your total income not counting the calf. And then maybe the calf will bring you another 10% from the beef chain. So $11,000 dollars. If you are a beef producers, you're producing where's your gross receipts are probably $2,000 a cow. So it does cost the dairy guys more because they need more infrastructure to milk all these cows. But [audio glitch] they are buying up land and driving the price of feed higher because in our area now they've got over 1000 acres of hay fields. So those hay fields are no longer available to produce, produce forage for beef cattle. Those acres now produce forage for dairy cattle. They're already subsidized. So you might say that the stuff that they're getting through the supply management system is now impacting the actual land purchasing ability of farmers. Yeah.

Researcher
Yeah. The more the more we think about it, more things come up that make these even even more complicated.

Participant
Well, maybe even a bit more unfair to think about it that way. It's not fair to the consumers, it's probably not, it's not a great deal for the beef industry. And it's probably not a very good deal for even the government in terms of how much money you have to spend, you get to get bills, which costs way more? Yeah. Yeah. So it's a bad, it's a bad business practice, is what it is.

Researcher
I see. And how do you see the future of everything? No changes expected or?

Participant
No, I see a change. But I don't think it's one that most people will want to speak about. But I think you have already experienced it in South America. And the change that I see is that if you have enough bad policies that cost the citizens of a country, too many dollars, eventually, what happens is the country gets into financial difficulty. And once it's in financial difficulty, they just start hacking away all the all the different programs. That's what they had to do in New Zealand. They had a supply management system in New Zealand, exactly the same as Canada, they ran into financial difficulty as a country. So they've completely eliminated it. So you know, we will we will in Canada, we will run into more economic hard times. Because we are not a well, not a particularly well managed country. And because our debt, our debt and our productivity are out of balance.

Researcher
I see.

Participant
So, you know, the solution will be an economic one event.

Researcher
And driven by the population, do you think because of this economic aspect? […]

Participant
Yeah, I believe it will. It'll also be driven by the taxpayer, not just the consumer. So you may say, Well, this is I am voting for us, I am going to vote for a party in Canada, that eliminates a system we have in place where it costs me twice as much to buy milk and cheese. So I'm gonna vote for those guys, because they're gonna make my cost of living more affordable.

Researcher
Yeah, I see that.

Participant

Right. Yeah. So that's what that's eventually what will happen. I don't know how long it will take. But you know, it will eventually take place. unless Canada prospers more than it's prospering right now. And right now it's not prospeting, you know, it's not we're not in a good fiscal position right now.

Researcher
Yeah.

Participant
Well, that's my take on it. But anyhow,

Researcher 44:05
Yeah.

Participant #2 44:07
maybe I'll be wrong.

Researcher 44:10
I don't know if anyone is right. If anyone is wrong, the more I learn more questions I have. But I feel like we talked about many, many topics. Thank you so much for your time. I'll definitely will have a lot to think about for the rest of the day. And I don't know if you have any final thoughts that we didn't cover?

Participant #2 44:33
No, I think we've pretty well covered it all I know what I would be interested to know and maybe we can have a conversation in in a month or two from now.

[identifiable information towards the end of the interview]

INTERVIEW P51

[Verbal consent, introduction, and demographics were removed from the transcript to guarantee participants' anonymity]

Researcher
Oh, that's very cool. Well, as I mentioned, I'm curious about this relationship between the beef and dairy production. So my first question for you would be if you had to describe this relationship between the beef and dairy industry in Canada, how would you describe it? And why?

Participant
Um, I guess the word that come to mind is symbiotic. Right? It makes sense. We're gaining efficiencies from both sectors. And the other word I would probably use would be growing or an opportunity, it's really an emerging sector and a lot of opportunity to write our future as to how we're going to do this as a country and create that opportunity.

Researcher
Okay, yes. And why do you mean on that opportunity, or how one industry can influence the other, let's say.

Participant
Right, you know, I look at dairy and the, the ability to take those calves which previously had very low value, if no value and turn them into a viable food product with really a minimal footprint, given the fact that you the footprint of the cow is offset by her milk production. You know, we have a real opportunity to produce more food with less resources, which I don't care what sector of agriculture, and that's always been the vision. So I, that's why I see it as an opportunity. I also see it as an opportunity in that we really haven't invented a livestock system in a long time, I would say the last one was when the hog industry went from farrow-to-finish to three site production in the 80s and 90s. And so we have an opportunity to think about how this can be done in a way that it's best not only for animal welfare, but also for animal health, food safety, lots of different aspects.

Researcher
Yes, I can see that. In one of the data that I found when I was doing my research is that already 20% of the meat production in Canada comes from dairy, which is all those cull cows or the calves that are actually their surplus calves in the dairy industry. Do you think that 20% is a big number or you're expecting it? How do you think this number may change in the future?

Participant
I think it jives with what I've heard, what I'm hearing is that we have probably in the United States started to taper off the growth of those dairy, beef cross calves. So that sort of a lot of the opportunities been captured. But there's probably still some room for growth in the Canadian market.

Researcher
And when you mean growth is related to those dairy, beef calves in that sense, or?

Participant
That's right. So, so increasing the number of calves or shifting the production of calves that are straight Holstein, to a dairy beef cross or potentially shifting even to a full beef cross, you know, through an embryo going into that dairy cow So I, you know, I think that 20% number doesn't surprise me. And there is an upper limit, because the dairy industry will always need to retain a certain percentage of purebred Holsteins. But beyond that I, you know, I think we've still got some room for growth in Canada.

Researcher
And one of the things that come in discussions when you're talking about those dairy beef calves is the differences of raising those animals in dairy farms and beef farms. So I'm interested on your thoughts about these different practices that happen on dairy and beef and how you think it can influence as you mentioned, animal welfare and health, but the overall production of animals.

Participant
Yeah, so I'm far from being the expert in this area, and mostly repeating things others have shown me or taught me. But what I've really come to understand is that those calves are an entirely different production system in of themselves, and they need to be managed that way, right through up until, at least, the feedlot, and even some of the feedlot operators would argue right through to slaughter. They even require unique rations to optimize their performance. So I think the mistake is made by trying to slot them into an existing system, as opposed to just defining them as what they are, and writing the optimal approach.

Researcher
I can see that and let's say we would create our adaptive system to those animals, how do you think it would look like?

Participant
Um, you know, I've toured some of the big calf ranches down in the southern states, I think there's, you know, a lot of opportunity for that intensive, intensive livestock model that still create room to manage the individual calf at, you know, the very young level. So, certainly, this is far beyond my area of expertise. But I think we've got lots of opportunity to manage that individual animal when they're really young. And then really uniquely, both nutritional and housing and groups, etc. Manage, you know, at the very young, I mean, think vulnerable animal we're managing. So to set that up in a way that we can really optimize their health, their welfare, their performance, all of those things. We have it right in Canada, honestly, I don't know. Do we have it right in the States? I don't know. I think it's evolving. And we're still learning.

Researcher
Yes, definitely. And some of the practices that come to mind at least that are different in dairy and beef is for example, as you mentioned, individual housing. The calves are removed from the cows soon after birth, and then in the beef side, they are on grass, on pasture, usually with their cows and calves together. So if you were to give a suggestion or a recommendation to a dairy farmer, who is willing to

start selling their calves to beef, what do you think they should do on their farms to guarantee a success for that animal in the beef chain?

Participant
Oh, boy. Okay, so I probably speaking outside of my area of expertise, I think a very close communication between the dairy operation and the operation that will be receiving those calves. It's close communication on how is colostrum going to be managed? How are potentially vaccines going to be managed? We have a lot of vaccines that can be given now, you know, at days of age, how is castration and dehorning going to be managed? How is the nutritional transition going to be managed and then of course, housing and transportation, I think it's all about the communication so that none of those pieces fall between the cracks more than about saying this is the right way and this is the wrong way. And of course then the other thing would be just communication across the chain on genetics, which is another huge opportunity we have, you know to it because we've got traceability in the system. So those would be the things but I think it really comes down to relationships and communication.

Researcher
Yes, that's not the first time I hear about having this communication mainly because I feel at least my impression is that the dairy in the beef are still very separated industries. I don't I don't know how you feel about it if there is much conversation between farm, dairy farmers and beef farmers, or how do you think this communication could start getting shaped or how it can be lead?

Participant
Yeah, I you know, to me it's it's a business to business relationship. It's not at the industry level. And so it's really knowing your buyer and or from the other perspective, knowing your supplier. And the consistency of having the same buyer supplier relationship is what creates opportunity to drive that. As opposed to, you know, treating the calves like they're just a commodity, in which case, then you lose that communication piece.

Researcher
Yes, yes. And one of the challenges we see with dairy is that for years historically, historically, the surplus dairy calves have been going to veal for example, because it's, it's not their main product, they produce and sell milk, the calves almost like a consequence. So this kind of reduces the value of those animals. And consequently, some circles like the male calves receive less colostrum or less milk overall. So how do you feel that these value of the animal can be improved? And how can we start working with the beef to kind of change those practices in dairy?

Participant
Well, I think that value of the calf has improved, I had heard, you know, values right up to $600 for a day-old calf. So boy, at those kinds of values, it is worth your while to put some money in and effort in to ensure the health of that animal that's, you know, is now a valuable byproduct coming off the farm. And I really think as operations get bigger, both on the beef and the dairy side, that's when we tend to get more of these business to business relationships, and then the consistency of the processes. So often in just, you know, the way the industry is structured takes care of that.

Researcher
I see. Yeah

Participant
How I see it.

Researcher
I think I think we cover many opportunities. And I do see this as a more positive attitude to what we can do with dairy and beef. But there's any challenges that we should be aware of, or something to keep in mind as we move forward on this?

Participant
Well, I think a challenge is simply perception, there is some negative perception in the beef industry. Certainly, people, there's a certain segment that would see these calves as competition. So we need to have communication that probably these calves have been entering the food chain always. They've just been coming in as Holsteins to your point. I think there is challenges, undoubtedly, that will arise with welfare and health. As we start to manage these calves intensely. I've, I'm a firm believer in intense intensive agriculture, but it's it's not not cost free. So we need to be we need to be intentional about health protocols, we need to be intentional about welfare. But all in all, I guess I see it, you know, if it was, I see it is predominantly an opportunity. And the threat, sort of the risks. They are foreseeable largely, and I think if we're proactive, we can we can get ahead of them.

Researcher
What would be being proactive, then?

Participant
I think being proactive would be investing in research. They are a unique animal. And so we do need to very much understand them. I think it would be proactive to consider how existing and future regulations could affect this industry and affect how it develops or doesn't develop. I think it would be proactive to be maybe including them in curriculums that that schools and agricultural colleges so that we've got, you know, our emerging consultants are aware. Um, yeah, I think those would be the big things I see. Yeah, maybe the communication maybe we could proactively talk about the moral, oh boy, they are top of mind for a lot of people. I was at four conferences in January, and they were on the agenda, every single conference. So they're, they're top of mind right now.

Researcher
Do you mean at beef events?

Participant
Thats right, beef events, and they're talking about, you know, one was on nutrition one was on genetic opportunities. One was just on the structure of the industry and the economics, but people are interested.

Researcher

Oh, okay. Yeah, this is this is nice to hear because we also see the same on the dairy side. I'm going to conference this week, and we're also talking about dairy beef and how to implement beef genetics on the dairy herd. So, but it's something that I don't think we saw, like, a year and a half ago. So...

Participant
Yeah, I would agree. I think it I think in the States, they've been talking about it for a few more years than we have. But the interest is really picking up in Canada.

Researcher
And, on your experience with other beef farmers, and maybe on those conferences or other events. What do you think is their main interest? Why are we talking about it among the beef farmers?

Participant
Some of it is curiosity. And just, you know, what's new out there? I think some of it is probably producers looking for an opportunity, is there a way to either expand or modify their operations? Um, I'd say those are probably the two drivers.

Researcher
Okay. And maybe one question more to myself now. And you mentioned research. As a researcher, what do you think we need to answer now? What kind of questions the farmers could be asking that we could help them answer?

Participant
So again, I'm not intimately involved in this type of production. So I would defer that to somebody working in this field day in and day out. But you know, the obvious ones would come to mind is, how is health different in these calves than a veal calf or dairy calf or a beef calf? What protocols work best? What are the biggest risk factors? Welfare, right? What is the best housing? What is the best monitoring? Where are risks for welfare? How do we measure welfare in these calves will be one. And you know, feeding protocols seem like a pretty obvious one, because they're not a veal calf. They're clearly not a beef calf. They're not, you know, on the cow. So what are those optimal protocols to set them up for for repro-, for success? Those would be my my sort of obvious ones. But again, I don't work in this space day to day. So.

Researcher
Yes, yes. The practical answers is for the farmers, let's say, so they don't need to take the risk. We take the risk first as researchers and then we inform them what it's less risky to do on farms.

Participant
Yeah.

Researcher
Some of the talks that we have, mainly on the dairy side, in general, are about public opinion. And mainly some practices that happen on dairy. So as we mentioned, the cow calf separation, no access to pasture in most of the farms, those are not well received by consumers. And those are things that do

not happen in beef. Do you think it can be any concern for the beef on that, like, merging the gap between the two industries for the public perception on that sense?

Participant
That's a good question. Answering carefully, I don't know that public perception is always right. Public perception is a thing. It doesn't necessarily need to drive. It doesn't necessarily drive the best outcomes for those cattle. And to meet that, the production of food and the best outcomes for the cattle need to be the driver. And so sometimes that communication, is there a risk? Yeah, probably there is right? Is there a risk in all types of things we do in agriculture, for public perception? Yeah, I think we need to constantly be telling our story, and the rationale behind our choices. But at the end of the day, it's a pretty good news story, from an from an environmental footprint point of view, from a food production point of view. Even from a welfare point of view, if we are proactive in how we design and and build this industry. So again, I guess I see more opportunities than threats.

Researcher
Yes, and some things that I heard before are also the public doesn't even know the difference between a dairy and a beef cow probably, so it's sometimes it's also hard to see how to imagine how people will interpretate the different practices maybe they already don't see much difference between cows. So we never know.

Participant
Yeah, I struggle with that. I struggle with public perception. I know it's something we need to remain engaged with what our consumers want. But we also need to be careful to still do the right thing. And then communicate the right thing back.

Researcher
Yes, yes. And you mentioned you ran a cow calf operation. And I think that's the main portion of the beef that differs from dairy. How you think the farmers perspectives of their rearing of those animals may be?

Participant
Yeah, you know, I don't know what farmer perspectives are, we haven't sort of done any sort of survey work or anything to my knowledge. And just anecdotally, I hear everything from this is really neat. This is really cool. This is a great opportunity to this is a threat to our industry, and those calves will compete with ours so the whole gamut is out there. What's the common perception? I don't know.

Researcher
I also don't feel like I see the same all the points of views. But I also see one leading institution, group, I don't know who should be responsible for trying to guide those conversations or if it is, maybe as you mentioned, in a supplier and seller level, or is it the responsibility of the beef industry or of the dairy industry to take the lead? Or maybe the genetics companies? Like I don't know if you have any thoughts on who should be the one guiding those conversations?

Participant

It's, it's an it's a great question. Certainly, we're leaning into it a little bit in the beef industry. So I had connected you with both NAME with ORGANIZATION. They're doing some work. ORGANIZATION and NAME, they're they're funding some research. We're talking about how these calves may or may not fit into the upcoming beef code. Because we weren't the codes of practice. So leadership is or you're right, there's a bit of a vacuum there. And I think that's still being sorted out. I think everybody's a little careful not to be stepping on toes. And so we're feeling that out.

Researcher
Yes, yes. And I see we have a few minutes left, I only have a few questions to you. Also, you already mentioned about it before. And I'm curious on your views about the production of a fully beef calf. So with the embryo transfer from a dairy cow, what is your opinion about this practice in general? Do you see any concerns or opportunities with that?

Participant
Um, well, at this point, I don't believe they find it economically viable. And I do understand that there are certain aspects of the cross-bred carcass that are actually a very desirable purpose. So we need to make sure that we're spending money for a more desirable product. But I think that as Theriogenology continues to evolve, I see it happening. And I see it creating quite a lot of opportunity for dairy producers, because now you have three choices when you go to inseminate that cow, and so you can sort of slot your market. And so I suspect we'll at least a certain percentage of the industry, we'll move that way.

Researcher
Yes. And maybe now also talking about the future. My last question is, after everything we talked in overall, how do you see the future of dairy and beef production in Canada? Let's say 30 years from now, how do you think is going to look like?

Participant
Um, well, I think that we'll reach that market saturation point as far as the number of new cow, you know, calves switching from Holstein, to crossbred. I think that we'll probably have a much more established supply chain. And to your point, some of these leadership questions, who's investing in the research who's doing the communication, those will be solved? And I would hope that we've got a thriving industry that's creating more opportunity in agriculture.

Researcher
Yeah, that's great. That's a very optimistic future. I hope I hope that becomes true. Yeah, thank you so much for your time. I don't know if you wanted to mention something that I didn't ask or that we didn't discuss about this topic?

Participant
No, I think your questions are thorough. And yeah, I've just wished you the best of luck with your research and glad that you're sort of delving into this new space.

INTERVIEW P56

[Verbal consent, introduction, and demographics were removed from the transcript to guarantee participants' anonymity]

Researcher
So, my first question for you would be very broadly, how would you describe the relationship between the beef and dairy industries in Canada?

Participant
There's a lot of people that would be have a lot of a lot better understanding of this. I think one of the big difference between the two industries is that the dairy industry is supply managed, and the beef isn't. And the beef industry traditionally has been quite adamant that they not be supply managed. And maybe that's a bit of a badge of honor in the beef industry. But on the other hand, we look at the dairy industry and see that consistently, dairy has been, I think, I would be able to say more profitable than beef in the beef industry in in general, or it has a more even it has a more even market than we do ours are in the beef industry, we have a much more volatile market than the dairy industry does.

Researcher
Yeah, and what do you think that that means? How, what influence do you think this has for you as beef farmers?

Participant
Well, I guess, as an individual, we're as farmers, we're all part of a big, a big scheme, right? We don't, none of us operate on individual basis, even if we'd like to think that. We're, we're, we have, we're influenced by international markets, and not by just what we do on our own farms. And I think that's what that's what makes the industry so, so complicated. And, of course, dairy. They, they're there. I think, if I'm my inter-, my feeling of the dairy industry, how it impacts what we think of the commercial beef industry is the ground the ground beef meat market. That's my interpretation of how dairy influences most the beef industry, the beef industry, because a lot of those dairy cows are getting you know the culls are getting ground and that's part of our, the whole the whole beef industry.

Researcher
I can hear the dogs in the back

Participant
I have barking dogs here. Sorry about that.

[...]

Researcher
Oh, no worries, yeah, I was going to, I think you already addressed this. But I was going to ask what the two industries have most in common and the most different. But I also want to ask you about how the

animals are raised in each of those systems. And what do you see the big are the biggest differences between the two?

Participant
Well, I think you don't understand I'm looking at as an individual producer, and not as someone that operates a feedlot or a background in operation. And I think in the, you know, as far as the, and I think in Canada, we're a little ways away from what, from the US where they have much bigger farms, and probably in Europe, too. I think in, in our beef industry, we're on a larger land base. And our animals aren't raised in the same confined, confined situation that the dairy animals are. I think that's, I think that's the biggest difference for a producer, like me, and someone that's a dairy producer, is that we're on a much bigger land base, the animals aren't in the same confined situation. And that's just the nature of the difference between the two, two industries.

Researcher
I see. Yeah. And I started researching about the dairy beef relationship, because inevitably, some animals from the dairy end up going to the beef supply. For example, the cows that cannot produce milk anymore, they will end up getting into the beef supply as like after they are cull cows for the milk production. And based on data of the Canadian Cattle Association, about 20% of meat production in Canada, comes from the dairy industry. And these are mainly the animals that, as I said, are not fit for dairy production anymore. So, I don't know if you ever heard about this, or you ever heard about this number? If 20% sounds too much, or it sounds okay.

Participant
You know, I'm not familiar with that I probably should be, and I think most cattle producers may have some idea of that but wouldn't be able to quote that 20%. It does surprise me a little bit. But it's it's a fact of life. We know that there's dairy cows in there. And when they're when they're finished producing milk, they go into the, into the food chain to the beef, the beef chain. So yeah, that doesn't that doesn't surprise me. I think it is really, really interesting. That shows a huge impact of dairy in the beef industry. Because for most of us, the cattle we're producing, we're hoping that aside from our cull cows, we're hoping that what we're doing is going into like, the the more expensive part like going away hoping that it's gonna be great. We really hope we all grade AAA, but I don't know if that's, that's a realistic objective or not.

Researcher
Yeah, yeah, I was just going to ask you. What does this dairy supply mean, to the beef industry? And I think you've already touched a little bit on that. I don't know if you want to expand on it.

Participant
You know, I don't really know if I can comment on the exact economic impact of that to the beef industry. But everyone knows that, that those dairy cows come into the, into that part of our market chain. So but I think we would have a hard time coming up with a 20%, which I find it's good to know that.

Researcher

Yeah, yeah. Yeah. So the animals that compose this 20%. They are raised as dairy and under dairy farming conditions. And they go through common dairy farming practices that are very different from how animals are raised for beef in beef farms. So I'm interested on your thoughts about how these animals are raised and about some concerns that we have on dairy and whether they could be concerns for the beef side as well. So the first group of animals that I would like to talk about are the cull cows and one definition that we can use for cull cows, and this applies to beef as well is an animal that cannot meet or maintain performance and economic criteria. In Canada, every year 30% of the dairy herd is replaced, which means that this amount of animals, almost 30%, will go to the beef supply chain. All those being animals that are not fit to produce milk anymore, basically. What do you think about those cool cows going to the beef supply any first thoughts?

Participant
Well, like I said, before, we I mean, we all know that is the same in the beef industry, when the animal is, is no longer, you know, producing the way you want it to, you have to make a decision to, well we say to send them down the road. And all I know is that in the when they're doing their job and producing a calf or in the dairy producing milk, they're valuable to your operation. So it's in the producers best interest to look after them as best they can. So I, my feeling is knowing what it's like in the in the beef industry, in our industry, we look after our cows the very best that we can, until they no longer are economically viable for us. And then we make the decision that we have to cull them or ship them. And my presumption is the same in the dairy industry, those until they're no longer producing milk, they're a valuable part of their, their production.

Researcher
Yeah, some of the things that dairy farmers have concerns regarding the cull cows are how they arrive at the slaughterhouses because sometimes those animals are not fit to produce milk anymore for health reasons, for example. So there's has been some findings on lameness and low body condition scores and how these animals are transported? Basically? Is that a concern for the beef side as well or not?

Participant
Well, I think any conscientious producer doesn't send a lame cow through the food chain, or, I mean, we're, our operation is part of the verified beef production. We have we have that we have verified beef production status on our farm. But even if we didn't, we would never ship a cow that was lame, or had was in poor condition, or had been treated with antibiotics or parasite controls. That's all part of our protocols, that none of those animals from our place, go into the food chain. And that should be for every producer. And I my assumption is that every dairy producer would follow that too. That's, that's only the right thing to do. So I don't have, I just I make a presumption that producers don't do that. And we have we have checks and balances in place, that we have withdrawal rates for all drugs, and anything that we use. And I think every producer, whether you're in dairy or or beef has, you know has an obligation to follow those. And that's, that's part of protecting, protecting our our industry and protecting the food chain, protecting our customers and our, our whoever's consuming our product. So I presume that dairy does that the same way that we do on our operation. So I can't I can't speculate or can't say that, that dairy operators do do it differently than beef operators. And maybe there are some,

but it's not the not the protocol that anything in either of our industries, anyone in either of the industry should be contravening.

Researcher
Yeah, I totally understand. And, yeah, the cull cows are a more stable part of those animals that go from dairy to beef. And the other group that I wanted to talk about are the surplus calves, which is my main interest. So a quick overview of the dairy cycle is that each dairy cow needs to produce one calf a year to maintain the milk production, which means that the dairy farms have more calves than they need as replacement in the herd and more than they can keep in their farms. So the male calves and some of the female calves they are considered surplus and they are usually designated to the beef supply. However, the value of the surplus calves does not sometimes justify the cost of raising them. So practice that happens in Canada and many other countries is that some of those animals may be killed soon after birth. When they are raised on farm, some dairy farmers may not consider those animals to be part of their core business, which is to produce milk. So as we talked before, it has been reported that some of those calves do not receive the same standard of care as the calves that will stay in the herd, for example, less colostrum or less milk in the first days of life. And that has resulted in more health, poor health conditions of those animals. So this is just an overview of the scenario on and the issue of those surplus dairy calves for dairy farmers. And as someone that also raises calves, but in a different way, what are your thoughts on the management of surplus dairy calves? And what do you think could be done to manage those animals?

Participant
Well, that's a really tough one, because like the calves are the cornerstone of our operation. We need every we need those calves and we need full like, everyone attains to have 100% calving rate with a calf that's going to be raised that we could eventually sell. And it doesn't work that way in dairy, although from some of the reading that I did. And I think this is where the where part of where your study is going is that it would be to the advantage of the dairy herd to not only have the cows pregnancy, produce the, I mean you know, given the renewed milk production, but to have the calves that that they use that they get from that to have value for them too. So I think that is a real problem for the dairy industry. As far as perception goes. You know, the public consumers don't like to hear that they're euthanizing calves that they don't need, or that they aren't getting the very best of care. And that's, you know, that's one of the whole things when we're when we're in food production using live with live animals, is that we are under a microscope for you for, for looking after the animals in a way that's acceptable to the consumers that are going to be the ultimate purchasers of our product. So I think I think dairy's got, they've got a big, big job to assure their consumers, that they aren't just using their, you know, their their cows as a commodity, which it's a little bit harder to do that in the beef industry, because every one of our calves is you know, we need it, that's where our money is coming from is from the calves not from the milk.

Researcher
Yeah. And that's how the dynamics are different right, from the dairy. Yeah, in so these surplus calves, they can enter the beef supply in two different ways, one as veal or as a dairy beef calf. So the most common historically in Canada, what has happened is for those surplus calves to be sold to the veal supply. And then the breed of those animals has been mostly Holstein and Jerseys. But veal

consumption is declining over the years and this resulted in the lower economic value of those surplus dairy calves, especially in some regions of Canada. So to overcome this, dairy farmers have started using beef genetics in part of their herd and as a way to increase the economic value of those animals. So the dairy beef calves can be paid more for, therefore, it is more sustainable in the long term for the dairy farmers. But then those animals they can enter the beef supply and be raised the same way as beef and be slaughtered the same way as the beef calves are raised by beef farmers. So I just I'm just curious on your thoughts about this basically new revenue of dairy beef calves and what is your opinion about the practice of using beef genetics in dairy calves?

Participant
Well, I don't think anyone would resent the dairy farmers from trying to get more value added off their surplus calves. Because we all understand that not all of them, they're not going to use those really expensive the expensive semen or embryos. They're gonna reserve those for their for it was the 30% that they need to re- that they need to replace. That's where they're that's where they're going to spend the money on that. So, but I think in the beef industry, we'll be pretty darn protective of how we raise our cattle because they, the dairy people can't raise them the same way we do. Those, those extra calves that they're giving the beef genetics that they're using the beef genetics on, will not be raised, like most of ours are in BC anyway, on open range in the, you know, on grass, using, where their manure is, is using to, to fertilize grasslands, and big, big areas. My perception, and I think most people in the beef industry's perception would be that those surplus calves are still being raised pretty much in confined quarters and in a different in a different situation in a more intense, more concentrated circumstances than, most, most beef cattle are. Most beef cattle until they're weaned anyway, they're on range with with their mothers. And those, those calves that, that they're using the beef genetics on wouldn't be raised with their mothers because they need the mothers for the milk production. So I think in the beef industry, we'll be pretty possessive of how ours are raised differently. Although when they get down into the food chain, it might be pretty hard, it might be pretty hard to distinguish. And I also really question whether the finished hybrid crossbred dairy or Holstein, and I think it's mostly Angus genetics they're using how it would be pretty hard for them to grade Prime or Triple A,

Researcher
The quality of the cuts?

Participant
I don't think the quality of the beef would be quite the same. I mean, I'm saying that from a prejudiced point of view, but one of the ways that you get good, good, you know, get get your top grades is from from muscle. And if the calves are raised from birth in fairly confined quarters, they won't have the same muscling that, that the animals that have been out on range do.

Researcher
Yeah. Yeah. My next question was about the influence of those, this new pathway, let's say the dairy beef system, what could that influence for beef producers in the long term?

Participant

Oh, I'm not really sure about that. I guess the influence, I mean, it will have an effect on maybe farther down the prep, you know, for finished cattle, it might have an effect on some of the price of a finished cattle, but I don't really think I'm quite qualified to say how they're gonna to influence the market in general, you know, and I'm prejudiced here, but I really do not think that they'll be influencing the quality. I don't think there'll be influencing that the quality of meat that's in that's in the chain. I mean, they'll still they won't be top grade. That's probably a beef production. lead producer talking about dairy cross cow, so I'm not sure if I'm gonna be quite qualified to comment too much on that.

Researcher
Oh, no, it's all good. I'm more curious about your opinion. […] I'm curious about your views as someone that raises the calves for beef, since they start, with also the future scenario of calves coming from dairy farms to the beef in compete with your animals, let's say, on the market.

Participant
Well, I mean, if there's more, I mean, there's so many factors at play in that, Bianca because the there will be more of not the not the ground, not the cows. It will be a different type of beef on the market. And it's, it's really hard to say how that will affect it, for me anyway, to project how. Because it all depends on how it projects, how it will affect the supply. And I'm just not sure how, how big that will be. I mean, as as it as it gets more in into the as more as more dairy producers embrace this, I think it will affect the supply because you know, everything supply and demand. On the other hand, I think about 50 to 60 percent of our beef production goes into the US. And right now the US has, the US herd is decreasing. So, but again, there is, it's going to be happening in the dairy herd in the States, which is much bigger than the Canadian dairy herd for sure. So, yeah, I don't really know how I can can project that I know, it all comes down to supply and demand. And if there's more beef, even if it is a lesser quality, like I say, not that not the top prime beef, it definitely it could affect our prices for sure. It could put downward pressure on, on our prices, for sure.

Researcher
Yeah, yeah. Yeah, it's very complicated. It's a complex scenario that we cannot clearly estimate what may happen. But yeah, so many things that play into it. Now, I'm curious specifically about the rearing of those animals. And you briefly touched on this. But do you have any thoughts or concern about how those dairy beef calves are raised? From the beef, from the dairy farm, but also maybe moving into the beef industry? Would they have a place on it?

Participant
Well, again, it's going to be very different because they're raised in a... First, first of all, they're not on their mothers. And they won't be getting the same colostrum and the same benefits of colostrum that that the beef calves get when they're raised with with their mothers. And I know because I buy replacement colostrum. How expensive that is. And I'm, I don't know how efficient the dairy the dairies are in saving the first milk and the colostrum and giving it giving it to the calves because I know that milk doesn't go into their their production chain, I think I'm not sure how long the dairy cows are kept out of the, their milk is kept kept out of the production because of the colostrum. But I'm pretty sure that it comes down to dollars and cents that those the beef cross dairy calves, won't be getting, in many cases, the colostrum that they that at the same rate that the beef cows, the beef calves would. So I

think everybody wants things raised naturally in the way nature intended intended to be and those calves will be getting the manufactured colostrum or the dried colostrum and the and milk replacer, which is very expensive. I know, because I've had to buy some for, you know, when we have calves we have to bottle feed. So I think I think again, that as a beef producer, my feeling is that those calves will be will be raised in a slightly compromised situation. And again, the likelihood of them having to having getting sick or having to have antibiotics is probably a lot greater than calves raised in the beef, the beef side of the production.

Researcher
Yeah, yeah. And I wanted to ask you specifically about the cow calf separation that you mentioned many times already. But I'm very curious on this specifically, because from the dairy side, this is something that we're aiming to change. We try to promote dairy farmers to keep their cows and calves together. So as a farmer, that that keep them together. I don't know your personal thoughts about the practice of cow calf separation? And yeah, what would be your recommendations, let's say for a dairy farmer when talking about keeping cows and calves together?

Participant
Well, I know it all comes down to dollars and cents. And that's one of the reasons that they haven't done it because they have to have a different facility and different. You have to have the facility to be able to do to do that. And it's probably it's probably better for the calf. I mean, then they're, they're, they have to go through the separation anxiety and everything. So I think it would be quite a difficult scenario, which would add to their bottom line which may make their product more expensive. I mean, I guess, again, that's where the beef people will say, well, we keep the calves you know, we keep the calves with with the cows. That's that's that's the way nature intended it to be. So if it was economically viable for dairies to do that they would be doing it.

Researcher
Yeah, I see. And do you think that their practices could have any influence on the image of beef? If like, the dairy and the beef merge more into the future? Could that have any consequences to what maybe people think about beef farming?

Participant
Definitely, yeah, for sure. And I think that's we're always trying to, I mean, anybody in any business is always trying to please their consumer and do what their, do what the consumer wants. And I think that would be, would be something that beef producers would be worried about the detrimental effect. You know, when it comes down in the store, looking in the meat counter, if you decide, again, from the prime cuts, you don't know if you're not going to know if that came from a beef animal or a dairy animal. And, and then, so if people can't distinguish that, then that may be something that would deter them from from consuming the beef, because they if they hear that the dairy ones aren't, you know, raised in a humane way or in a in a more, not humane, not not inhumane, but in a more undesirable way to consumers, then that may inhibit their consumption of our product, which is beef.

Researcher

Yeah, yeah. Yeah, now that now I know that you we started this conversation with you thinking you could not collaborate much, but you helped me a lot on reflecting about it. I don't know if you after hearing about this, about the possibility of dairy becoming a bigger supply to beef. And those animals like entering the market the same way as beef calves, if you have any other thoughts that people that are working like me, researching about these topics should take into account.

Participant
Well, you know, I'll be interested at my meeting on Wednesday to find to talk to other people to see if they're aware of it or under have an understanding of it. Because it, you know, maybe I'm not as well informed as some other other producers or other people that. So I don't I'll be interested to hear what other people have to say. I mean, it's, I think it is going to, it will affect us if, if, you know, and our, in our industry, we're so concerned about perception about what we're how our image is, as far as consumers go, and the general and the general public. And if people, you know, consumers think that animals raised in what they like to call factory conditions are getting into the regular food system when you're looking at the meat counter, that some of those animals have been raised in so called, although everyone likes to call feedlots and everything factory farm, so I think it's gonna be so much of a perception. And as I said earlier, that the beef producers are going to want going to want to hang on to the fact that their animals are, you know, out on the range and harvesting sunshine to make good beef, although most of our most of our BC beef goes most of our cattle in BC end up in the, you know, in the feedlots in Alberta, and in what a lot of people think is a factory situation, so, but I think we will hang on to the fact that our animals are, you know, have a good path as part of their life, have access to free range and that they are in, you know, what people what the consumers like to think the traditional settings are, no matter what, what industry you're in, if you're raising animals for consumption, it's really important to know that they're treated humanely and that it's a safe product.

Researcher
Yeah, I totally understand that. And I'm also curious to talk to some feedlotters on their views about it because I have this suspicion that maybe they have different views from cow calf, people who run cow calf operation.

Participant
They will because they're, they're just they're another sort of step in the in in the chain. And they don't have that they don't go through the birthing process and, and raising the calf and weaning the calf. But they do have, you know, they're huge. They're huge part of the industry and also they're very concerned about their the perception of what they're doing too. So they'll you'll definitely get a different perspective from that.

Researcher
Yeah. Oh, I almost jumped one question. But another practice that I was surprised to hear about is that some dairy farmers are interested to use embryo transfer to produce a pure beef calf. So they would transfer an embryo of only beef genetics into the dairy cow. And then a dairy cow would produce a purebred beef calf, she would produce the milk for them to sell and this calf would have the same value as a calf that is born in your farm, let's say, What are your thoughts about this?

Participant
Well, I think that probably, well, that's more, I'm not sure if that it's all gonna come down to economics, right? How much the embryos cost and where they'll get the embryos? And then again, I think it's still going to be the perception of the circumstances like how that beef calf is raised too. Because they will be raised in this in a similar situation as the other dairy calves, and also be subjected to the same stresses where, again, I'm not sure I'm, I'm not sure if this is a valid quote, but I did read in the one article, and that was one of the concerns of the dairy industry is that their calves in general, receive more antibiotics and have higher mortality, and a greater higher incidence of sickness than I think the beef herd. So because if they're born and embryos born into that situation, they would probably be subject to the same pressures.

Researcher
Yeah, yeah. Yeah, this is one, this process is definitely not common. It's something that is a starting as an idea. But there are all practices that aim to overcome this, what to do with the excess calves that end up being a surplus product of producing milk basically. So yeah, they, my mind, just personal view on this is that they are trying many technical solutions to this problem. But maybe there are other aspects that they should take into account, like what you mentioned the quality of care of those animals, before they start investing money, because it's very expensive, for example, to do an embryo transfer, to have these pure beef calves. But it's a very complicated thing from the dairy side that hopefully we'll be able to find a sustainable solution that works for the beef side as well. And this is why I'm doing those interviews to hear the beef opinion about it because no one ever asked your your side about it. And yeah, this for all the questions I had. And now I wanted to just give an open space, if you have anything else that I didn't cover that you think would be interesting to look more into, or anything that I didn't ask you about.

Participant
I can't actually think of anything right now. But I have to say that since I talked to you last week, it has given me a lot to think about and how it, how this this concept. There's just you know, there's so many pressures on our industry. So many things happen, you know, like the price of grain. But what's happening at the Grain Exchange in Omaha or something affects affects me ultimately, because that's, that's, you know, there's just so many pressures and so many aspects of the industry that we really don't have control over. And I think, actually in the beef industry, the biggest thing that for, and I mentioned this earlier, that for the beef industry, I think. Given this and see if it does, does amount, like dairy farmers, like every farmer has, often has a hard time changing, right? They like to do do things the way they always have done and yet, there's always the people on the leading edge that can see that this will help them help their bottom line. I read there's one farmer in Ontario and this was in In 2021, or something, it was another $50,000 on his added on his revenue, which you know, is for a lot of operations, that's, that's quite significant. So, but I think that I really honestly, I truly think that the beef side will really be very protective of our cow calf operations, which is dairy, dairy's cow calf, the cow calf operation is quite distinctive. And is in many ways, the beef that we're producing will be more wholesome. That's my personal feeling. And I think the beef industry will, will really hang on to that. And that's how we will probably try to counter this to be possessive of our aspect of it. And that's going to be the perception. But that isn't going to change the supply and demand and the how the how many of these, these new crossbred dairy, beef animals get into the market. And we won't really be able to

control that, but we're going to look at how we're perceived in the market. I think, I think that's, that's, I, that's my opinion, of how our side of the industry will, will try to control that.

[end of interview]

INTERVIEW P59

[Verbal consent, introduction, and demographics were removed from the transcript to guarantee participants' anonymity]

Researcher
So in your opinion, if you had to describe their relationship between beef and dairy in Canada, how would you describe it?

Participant
Well, I think that for the beef, it's more a lot more grains being fed, and, you know, more grains than for Holstein, for the milk production. They feed silage and grains and things like that. There's a different feeding program, for sure. But as far as the rest goes, I think that, you know, that they're similar, but not really similar. You know what I mean?

Researcher
What would you say is the main difference between the two industries in that case?

Participant
The one is producing milk and the other is producing the meat. And they do have the you know, they've been consistent in have been very good market price for their dairy products, whether they're converted into cheeses or whatever, but just being dairy products. And so they've always had a consistent, made a very good thing where, the cattle industry is up and down, and up and down. And at this very moment, the cattle industry is up. So yeah.

Researcher
Yeah, I can see that. And I was doing some research on this overlap of how many animals from dairy go to beef in Canada. And I found that around 20% of the meat production in Canada already comes from dairy. And I don't know your opinion about the number if you think that's a fair number or it is higher than you expected or lower?

Participant
Say that again. How many you thought?

Researcher
Yeah, so it's 20% of the meat production in Canada comes from dairy. And was wondering how do you feel about that number?

Participant
I don't really agree with that number. Actually.

Researcher
And why so?

Participant
I don't know. I'm not sure that I would agree with that number. I think that it is not that high.

Researcher
Oh, you suspect it's lower than that?

Participant
Yes, I do. Yeah.

Researcher
Yeah. And this number, I believe, from what I saw, it's mainly from cull cows. So for example, many dairy cows with at the end of their production life, they would ultimately go to the beef production. Definitely not a, high quality meat, as many of the beef producers would have in the end. But it contributes to beef production in some way. But what we're seeing now is this increase of the dairy calves going to beef. So as I briefly explained to you, the dairy farmers are using the beef genetics on their herds. And then they have those calves that they don't need, they don't have resources to keep on their farms anyways. So there is some interest from some beef farmers to buy those animals. So I was just wondering how much you heard about this before? If you haven't experienced about that before?

Participant
No, I haven't actually heard of any of this before. This is just all new to me. And I'm thinking okay, well, they have their own market. But why now, the beef is at a high market for beef cattle producers. Why they would want to jump in? And, you know, kind of capture that. It's just I don't know. How much productivity do they want? You know what I mean? Like, they're infringing on beef cattle, you know, they've already got a very good market. And now they're, you know, kind of trying to get into the beef with the, you know. I can see they're trying to utilize everything they possibly can.

Researcher
Yeah, I think that that's linked with my next question about that is if you see any challenges or opportunities with that practice to beef producers, do you think this is more of a good thing or more of a bad thing for the beef industry?

Participant
Well, like if they did cross and beef them up there? And they did that, you know, are they expecting to get the same high prices as the beef cattle producers? You know, this is my question, is that what their goal is? Is to, you know, just I'm not sure about that.

Researcher
Yes. At the moment, the main reason that we see for the dairy farmers to use the beef genetics is to increase the value of those calves that they already don't have any market. So it also making an animal more attractive to beef farmers to invest on and to buy from. So it's kind of looking for a way to have an output for those animals.

Participant

No, I don't really know. I just think that it would be great if they could get a better market for them. But I think there is a market for them. Like I said, a lot of people like them, because they have a different enzyme and breaks the meat down. It's more digestible coming from the Holstein. Like I'm just talking Holstein, I don't know about the rest. And I do know that it's very, very good beef once it's you know, grained and fattened up, like a beef is fattened up, like beef calf. I know. It's very, very good.

Researcher
Yeah. So there may be some opportunities for the crossbreeds maybe to invest in some research on that and see how we can influence maybe the meat quality on those genetics?

Participant
Well, it would certainly make a market. It would get a good market going if, it could influence that. Because they're so bony. They are tall and a lot of bones, and there's not a lot of meat on them. They are not meaty. You know what I'm saying?

Researcher
Yes, yes.

Participant
So it would be very beneficial if they could increase that beefiness, beef up those cattle, and they would be a very delicious […].

Researcher
Invest on making those animals profitable for the end goal of the product that they're going to be raised for because now they're raised for dairy. And they have been selected for many, many years for dairy. So now we're kind of changing that trend, right?

Participant
Yeah, they still have their dairy producing cows right? They are just trying to get that beef market. They are trying to get rid of those calves at a higher price and make them more valuable so they can re-enter into the beef industry.

Researcher
Yeah, so that's how they're aiming to get more profit from those animals, from the beef supply. Yeah. And they have been pairing that with sexed semen in the dairy. So basically, they can identify which cows, which dairy cows have a good genetics for dairy, invest more on sexed semen, which has higher chances of having a heifer in those cows, and then in the lower genetics dairy cows, they can use the beef semen and then get rid of those animals from their farm to the beef. That's how it is looking like now.

Participant
It sounds very viable. It sounds like it would be a very, very good thing.

Researcher

Yes, exactly. So that's why we suspect that this is a practice that came to stay. And it's probably going to increase in the future, if we see the dairy side of it. But now I'm talking to beef producers on what are their views about that practice? And if they think that's sustainable in the long term and how they think that would affect them?

Participant
Well, I don't think it's going to anybody, because I don't know exactly how many they have. The dairy cow has milk production for females. Right? Then they have the other ones, there's really no market of. A lot of them used to knock them over the head. Years ago, that was a practice, just to get rid of them. Nobody wanted a dairy cow, you know, and that took a bit for grass, just to eat up around the place. And that kind of thing, right?

Researcher
Yes, exactly.

Participant
There's really no market for them. And now they're trying to gain this market. And I think that it would be well, I think it would be okay. Like to me, it's alright for that to happen. But the other thing is too. There's also for cows that have those calves, they also have an advantage because they sell the colostrum. You know what I'm saying? They have a market there for colostrum. And that colostrum can be very, very expensive. They dry it, dry that cow's milk after they collect the colostrum, which is a large amount. They sell it, you know, so, I mean, they have a lot of advantages already. So they're just trying to get more I can understand that. But I would I would not be opposed to it.

Researcher
Yes, yes, definitely. And I know it is something that you heard just now and it's very new. And maybe because of that, what are your main maybe questions or doubts about this practice? Or why would you like to know more about it?

Participant
Well, I'd like to wonder about the market for it. There are still very bony, alot of weight for pricing, like a lot of that weight goes into bones. And so you know, there's a there's a little something to kind of worry about. Now, if they beef it up, does it really, what are the genetics going to look like? Is it going to get rid of that bone while they're trying to beef it up? Because, you know, I don't know what they plan on crossing with, or just the genetics just to get whatever. So?

Researcher
Yeah, so more and more research maybe on the genetics and how it's going to influence the performance of those animals.

Participant
That’s right. they have to have a trial. A trial to see if this is even viable. You know, if it is and of course it can't be genetically modified because of its genetically modified, they got no market because people aren't going for genetically modified anything in beef.

Researcher
Yeah, that's going to face some maybe consumer barriers right there.

Participant
14:06
Yep. Yep, that's right. There's going to be some backlash from that for sure.

Researcher
Yeah. And then in addition to the genetics and other things that we talk a lot about this topic is how the animals are raised. Because the dairy is very different from a cow calf beef operation, for example. So in dairy the calf will be removed from the cow soon after birth. They're going to be housed most of the times individually and indoors. And is basically the opposite that happens on a beef operation. How do you think those different practices can influence those calves and how they're going to succeed if they go to the beef supply chain?

Participant
Well, I don't know what they're feeding. Are they feeding them milk? They usually go with those leaflings, you know what I mean?

Researcher
Yes, yes.

Participant
And then keep them separate. And then they feed them out of there. But if they're feeding the milk powder or what they're feeding them from the dairy, I don't know.

Researcher
It will depend on what they do, what they provide for those animals in a sense,

Participant
Well they can't get milk, because that takes away from the milk production and their sale there. So, what are they going to do? How are they going to feed those calves because they need milk, they need that nutrition. So we'll have to have a supplement.

Researcher
Yeah, so some dairy farmers do give part of the milk they produce to the calves. Usually around eight liters a day would be a high amount. And then some other farmers do the powder. So it depends on a case by case scenario. And then some give it by bottle some by buckets. So it also depends on farms. But for sure all of them are not with the cow, for example. So yes, I was wondering, and this is something that I'm asking the beef farmers their opinion on. Let's say if a dairy farmer came to you, and said that they are interested to sell their surplus calves to the beef supply, they are dairy beef calves, let's say half Holstein, half Angus. They want to know what they should do with those animals in the

first week of life that they are in their care. What do you think the dairy farmers should be doing to those animals to guarantee they're going to succeed in the beef supply chain?

Participant
Well, they're going to have to do the first one, they're going to have to follow through as if it would on a beef cow, like you know, buy the milk, milk and everything else. And then just see actually what they actually produce, how they what the meat is, like, what the weights are, what's the how much it costs to to make that a beef cattle.

Researcher
Invest on that. And we've asked many, in the nutrition, would it be?

Participant
The nutrition and the investment, it could be pretty big. And it just doesn't seem viable to me, to get them to that without being on the cow or out you know. And eight liter is a lot a day. I mean, you start out with four liters twice a day, or a day, four liters a day. In the end, you would go when they start getting bigger. And when they're going to wean them and what weight do they wean them like getting the milk? From calving time. Okay, how long are they on milk for? Till they're like three months, four months old or three months old? And then they go to grass? When is their birth? Is that feasible? Like those kinds of things? There's lots of questions like when they're calving out, how long they have to be on the milk, if they make grass season.

Researcher
Yeah, those are all the practice that are very different on an in dairy compared to beef. Because as you said, they are calving year round. So all the time, they're going to have calves. And they will usually wean them around 70 to 90 days. So that's way shorter than what will happen in a beef operation.

Participant
Absolutely, they go wait five months, they go from January to Fall. And then the other one is there's the calves are on the cows. I mean, it's a lot longer.

Researcher
Yeah. So do you see any effects that those practices could have on those animals? How do you feel about that?

Participant
Well, they might be defeating their own purpose. You know what I'm saying? They might just be defeating their own purpose. I mean, because they have to make it viable, like how long are they actually going to feed? You know, what, how long before they can go to grass and do the time everything has to be changed? So they can go to grass? I don't know.

Researcher

Yes, yes, definitely. So there's that there's an maybe a need to invest in those animals to make sure that they're actually going to be successful in the beef and is not just sending the animals from dairy to beef. Maybe some changes need to happen on the dairy practices as well.

Participant
There definitely. There have to be done on that part. But I just like I said, You've got to make this viable. I mean you really got to watch what you're doing. I mean, you can't be feeding them milk as long as they. I can see three months of milk till they are three and a half, then they go on to the grass, they have to have a little bit of green supplement to make to beef them up. I don't know, I don't know exactly what the what the genetics? That's big story.

Researcher
Yeah. So they need the genetics to make them resilient to that ad associate the genetics with the good practices, let's say. Yeah, and then we talked a little bit about the consumer perspective before when we mentioned about the gene editing. But I also wanted to ask you about the consumer perception, because that's something that we talk a lot on the dairy side of things. Because there are some practices that happen on dairy that the public, the consumer, may not approve one of them being the cow calf separation, for example. I was wondering how much the beef industry talk about the consumer opinion. And if you think the consumer opinion about beef is different from dairy in any way?

Participant
Well, I don't think for myself, I have separation, that's just business. That's, you know, you got tree huggers, they might, they might have a few things to say about it. But separating them, they've been doing it all along. There hasn't been any protests anyways. So that's it what they do. And it's successful, they can do it cheaply and successfully. Well, better off to them.

Researcher
Yeah, yeah, it's just hard sometimes to explain that to consumers, because there's a lot of media out there. And it's always something that comes up on Facebook or things like that, that is out of farmer's control. And I was wondering if that's something that happens in the beef production as well.

Participant
No, in the beef, it's the hormones. I see. People who put hormones in the beef and you know, the needling and making sure that they don't have antibiotics in them. And this and that, you know, when they're in the beef production, there has to be, it's very much right now on the honor roll, you know, you treat something that, you know, you're going to sell, you have to wait 48 days, or whatever the duration of the medicine, leaving the meat. So it's basically on the honor roll, which has, farmers are pretty darn good about it, I always. It's really not been an issue. But it is an issue in the feedlot, they want the maximum for the least amount of input to get the maximum output. So they use hormones into the year like around grow, or for a heifer or for a steer, it's a growth hormone. And a lot of people are opposed to that they won't buy that. Top dollar goes to the organically grown one or whatever, as long as there's not hormone. So you got that it's a deep industry that's kind of a taboo. And in the dairy industry, I mean, what could be, unless it's genetically modified, they're genetically modifying these, then it won't work. Nobody wants. Nobody wants to GMOs in anything. That's the a big one. Yeah.

Researcher
So there's challenges with the consumer perception for both industries in a similar way. It's just different topics.

Participant
Yeah.

Researcher
Yes, I can see that. And yes, and then one more practice that people have been talking about. It's very new. And it's not happening on farms. But there's some research happening. So I'm curious on your thoughts, and it's doing embryo transfer of pure beef embryo into a dairy cow. And so with that, the dairy cow would produce a beef calf, and would get the milk from that gestation to the milk production, and the beef calf would go to the beef supply chain. What are your thoughts about that practice? And have you ever heard about that before?

Participant
Yeah, artificial insemination. We've done a whole herd. We wanted specific genetics. And so they were flushed. And then we did them and AI?

Researcher
Yes, yes.

Participant
So you could do the exact same thing with the proven beef and that would be fine. That's still natural. That's natural. It's artificial inseminating. It's not naturally ovulated, but but it is still an embryo and an egg, you know?

Researcher
Yes. Yes, you're not... Sorry?

Participant
There should be no trouble with that.

Researcher
You're not modifying the DNA, as in the case of the gene editing you're talking about exactly.

Participant
You're not modifying but the minute you modify, nobody wants. Yes. Here's another interesting fact. They've done it in pigs. But they haven't done it in beef cattle. These bunch of wackos these vaccinating people that have been murdering and killing all these people with these vaccinations. I got five neighbors all dropped dead have the third shot, all three neighbors dropped dead. The other two drop dead about a week after they got the shot. Oh yeah. Anybody that could be vaccinated, it was a big scam. So here's the new thing. They wanted to do that technology, the mRNA, in vaccinations for

beef cattle, for all cattle. Well they did it in the pigs, and let's just call it 100. Okay, 33% died right away. The other, but now we're up to 67% died shortly after. Lots was born with let's say defects. The other one was born with defect, poor [not audible], non-productive. It's a terrible situation. So only about 12%, out of all the 100 came out normal. So if they think that's going to change all the vaccinations in beef cattle, nobody will be vaccinating and nobody will purchase or buy mRNA beef.

Researcher
That's crazy. Yeah. That's, that's why we need to be super aware of the new technologies.

Participant
Our farmers, beef producers are producing and putting videos out and everything else. And the thing they're trying to hide is what happened with the pigs. They tried it in a group and the 1000 cattle are dead. The shot killed 1000 cattle down in Texas. Well, it's just not going to happen. But that's what they want. They want the meat infected with that mRNA.

Researcher
Yeah, so and then that's why I see people are suspicious about all that new gene editing all that technology. And the consumer is on the same page. No one wants to have. That's a different story from the embryo, as we were saying in the embryo, you don't? Yeah, it's a more natural process. And they have been using the embryo transfer for that specifically because you overcome the genetic issues. So as we were talking before, Holsteins, they have a lot of bones, they don't have much beef quality. So with the with the embryo transfer, you're gonna have, you are not going to have that problem. So that may be something that is interesting.

Participant
Yeah, that's, that would be very interesting to see how that goes.

Researcher
And you're open to that, you think?

Participant
Absolutely. Who would be the most genetically dominant? You know? Is it the beef or the dairy. You know, you got a 50-50 chance there.

Researcher
Exactly, yeah. Amazing. Yeah. I'm just going over all my questions, and I just have one left. So let's say we have a time machine, and we traveled 30 years in the future. How do you think the beef production is going to be looking like in Canada then?

Participant
Well, I believe in the Lord Jesus Christ? I can't answer that, because I know that we are at the end times, and we're going to be raptured out of here. There's seven years of tribulation and then 1000 years of living, I don't know.

Researcher
It's very uncertain.

Participant
God won't be allowing any such thing. Right? So I can't give you anything in the future. But I mean, it would be wonderful. If that was because there's such a shortage of beef right now. Worldwide disasters in Texas, the Mongolian cattle, they lost all their livestock. Texas, they burned them up. I mean, those are big producers, big big producers. We hear a lot of people are elderly. They're getting out of this. Their kids don't want to farm, you know, so we're in a real dilemma for as far as beef goes. So, the positive here is that in the future, I think that if they can successfully AI them into the dairy calf into a beef cattle, but then at the same time, they don't know what? So is that going to alter the milk production? […], well there's the risk.

Researcher
Yeah, sorry, you cut it off a little bit, but I think I got most of it.

Participant
If you're in the future, let's just say the dairy industry, they start trying to beef up all these babies. And they're getting females, which they need to reintroduce it to their milk production. And they are going to be beefed up well, then what's gonna happen? Are they going to I don't know, they have to keep a pure breed for? I don't know, it's a very defeating. Is it going to be a beefy heifer and then not good at milk production because it is more beef? Or is it going to be a beefy steer? That's going to be in the meat production? Like they could be cutting your nose off despite the face? That's the trouble. That's the trouble when you start, you know, AI for the beef […] and then you end up getting and they lose their Holstein attributes? [...] That's a risk for the future if they want to go this way.

Researcher
It's important to keep the goal. What is your main goal? First, right? If it's for milk or beef, and then focus on that?

Participant
Yeah.

Researcher
Amazing. Yes. Thank you so much for your time, we went over all my questions. Thank you for that. Is there anything that you thought about everything that we discussed that I didn't ask you, but you would like to share?

Participant
Well, I wish them luck. I wish them luck. And I need, everybody wants the maximum, you know, to diversify and make maximum production, you know what I mean? As far as it's all about, it's all about the investment they put in, and what they get back. That's, that's the bottom line. It's the cash. And that's what this is all about. Those calves just aren't doing it, right? And unless they get a market, you know, complete market that just deals with these cattle, I don't know and fatten them up. But beef, I will

tell you that the Holstein is one of the better meat when you put it on your table. For the disadvantage of the bone. That's a lot of waste, right? And the bone and, you know, a lot of people don't know, but the big producers, the 1000s of cattle like the Nielsen brothers, like Dennis Niles, the big big producers, when they want for on their own plate […], they use the Holstein in a feedlot fattened up. That's the meat they get. So there's a big advantage to have the them beefed up because they have that extra enzyme that breaks that beef down. That makes that beef more easily digested. And it is it's much easier to digest it with that enzyme breaking that meat down already. It's very, very helpful.

Researcher
There's definitely opportunity there and there's reason to invest on. It is just figuring out the best way to do it. Yeah.

INTERVIEW P70

[Verbal consent, introduction, and demographics were removed from the transcript to guarantee participants' anonymity]

Researcher
So one of the questions that I have is, how would you personally describe this relationship between the beef and dairy industries in Canada?

Participant
Well, in fact, I don't think there is much relationship between the ranchers and the dairies other than you know, in a sort of peripheral way. I was learning from NAME and then a little bit from you and reading about it, but there is more beef coming out of dairies than there used to be I mean, always knew that cull cows were turned into beef. And we have now more dairies up here that are buying out ranches to raise young animals or to [phone statics] that's one relationship I'm aware of, but that's more where the dairies are sort of just taking over the ranches. We also buy cull dairy cows as nurse cows, so I've done that and clients and [phone statics]

Researcher
oh, sorry, I think it cut off a little.

Participant
Okay. Well, I was saying that we buy cull dairy cows to be nurse cows. Beef ranchers.

Researcher
And what would you say the two systems have most in common? Or maybe how different they are in the way they operate, the beef and the dairy farmers?

Participant
I think it's radically different. The dairy is very intensive, you know, confined animals, not out on rain [phone statics] My cattle it's [phone statics] for the animals.

Researcher
Sorry, you're just cutting off again. I'm not sure if it's my side or your side?

Participant
Well, it's probably mine, because I'm up in a sort of country setting here. The reception is not that perfect. Okay, so what did you not? Or what did you hear last?

Researcher
I heard that the animals are raised differently. So in dairies more confined, what does not happen to beef.

Participant

Yes. So my animals are grazing most of their life. And then in the winter, that they are fed, but they're still fed, or in a big area where they also are grazing and are in the woods, if they want to be. I mean, that's the animals that live here all the time that the calves that are [phone statics] into a more intensive kind of situation in a feedlot, but that's not my operation.

Researcher
Yeah, and I heard already this before about how different the animals are raised, but also some comments on how the market operates for the two industries. Like the dairy is supply managed, what doesn't happen in the beef production. I don't know if you have any thoughts on that?

Participant
Well, I certainly know that the dairies have an enviable price that they know is coming to them. And that has some relationship logically to the costs of production. Whereas in beef, it's really a bit of a gamble, whether you are going to get enough money in the fall to pay for what you've done. You don't really have any certainty at all.

Researcher
Yeah. And do you think the supply management of the dairy industry has any influence on the beef production?

Participant
Has what to the beef?

Researcher
Has any influence on beef?

Participant
Well, I think because now dairies [phone statics] beef properties, because they have more money. And so some of these places, that were beef ranchers now are not, they are taken over by dairy. And I think that's probably because they have a bigger income. [phone statics] to buy the beef people out.

Researcher
Yeah, I started doing some research on this dairy beef relationship, because inevitably, some animals from the dairy industry will end up in the beef supply, as you mentioned, like the cull cows, for example. And then one data that I found is that based on the Canadian cattle Association, about 20% of the meat production in Canada, comes from the dairy industry. I don't know if you ever heard this number before or if you think this is a big number, or is it as much as you expected?

Participant
No, I think I've heard that before too. But that, and I think it probably is, or I assumed before it was cull cows getting mostly ground up into, you know, hamburger or not the most prime cuts of steaks and roasts that I think our beef animals are producing when they're slaughtered younger too compare it to the mature cow.

Researcher
The different quality of the product that comes from dairy, let's say.

Participant
Yeah, I would think that the dairy beef is going to be of a lesser quality. Not that I think hamburger is anything less it's just a different, cheaper product.

Researcher
Yeah, and this 20% is mainly of three groups of animals, one being the cull cows, but then also the veal calves. And then lastly, but increasingly, the dairy-beef calves. So I will go over each of those, and then make some questions about each of them, if that's okay. So the first one that I wanted to talk about are the cull cows. And one definition that we use for cows. And this is both applies to beef and dairy. But it's an animal that cannot meet or maintain the performance and economic criteria to be kept in the herd. And another number that I found is that in Canada, around 30% Of all the dairy herd is replaced every year. Which means that 30% of all dairy cows end up going into the beef supply each year, which may explain this high number for the overall dairy supply to beef as well. You already touched a little bit on that, but any thoughts on the supply of those cull dairy cows to beef, maybe in addition to maybe being a lower quality product?

Participant
I guess my concern is more animal welfare one. I feel badly for those young dairy cows, that can't, that don't live a very good life, and then don't even live a very long life. More that a life must be very hard on them to no longer be kept after they're four or five years old.

Researcher
Yeah, I also discovered quite late in my vet school studies that the lifespan of a cow in North America in the dairy farm is around two to three lactation years. So it's a very high replacement rate that they have where a cow would leave only this amount of time in a dairy herd. And this is a concern for the dairy farmers as well. There's a lot of research on how to improve the longevity of those animals.

Participant
I don't have a simple answer, I think [phone statics] they work so hard, metabolically. They're producing so much milk and it's hard to to do that and stay healthy and sound and yeah.

Researcher
And another concern that the dairy side has is, in addition to the welfare is like how those animals arrive at a slaughter houses. Because most of the times when they are not producing anymore, the reason why they are being culled is maybe health reasons or metabolic reasons, as you mentioned. So there are some research finding like lameness and low body condition scores and in those cull dairy cows when they arrived to those dollar houses. And in the end, those animals are not the priority of the dairy farmers because their main income comes from milk. So do you think that could be a concern for you as a beef farmer as well?

Participant

Well, yes, I do. But mainly, I think, because if cows are perceived to be an are being treated poorly, then that's going to be a problem for everyone that produces cattle or works with cattle. So the public will perceive that these skinny old cows are being put on trucks and getting banged around, and some of them are not even able to walk properly. And so then the whole beef industry, our meat eating industry gets blamed. And that's not the case for our strong beef animals.

Researcher
Yeah, this is one thing that I'm finding more, the more I research about the supply of dairy to beef is kind of the associated risk that it brings to beef producers. Because as you mentioned before, the way animals are raised is more natural, let's say in a more natural setting. So not confined, on pasture, which is not the case for dairy. And with time, maybe we won't be able to differentiate anymore where those animals are coming from. And these may reflect on beef farmers that in the end of the day don't have anything to do with some of the practices that happen in the dairy side right.

Participant
I don't think you can tell if you go to the store and buy beef, whether that was dairy or or ranch beef. It's not going to look any different and you maybe they aren't getting graded AAA, ribeye steaks and things those are probably beef reading of not dairy. But no I don't think the public can tell.

Researcher
Yeah. Yeah. I may have more questions about that later on. But now moving to […] can you hear me?

Participant
Yeah, I can there was a couple of seconds there.

Researcher
Oh, sorry. Did you say anything that I didn't hear? No,

Participant
I was listening but I didn't hear anything. Okay.

Researcher
Yeah, I was just going to say I may have other questions related to that when we talk about the dairy beef calves. But the second group of animals from dairy that goes to beef are the veal calves. And many people may not be aware of this, but a common practice on almost all dairy farms is to separate the calf from the cow right after birth or following within the following hours. So the cow goes to be milked and you probably know all this but the destination of this calf will depend on whether if it is a female that needs to stay in the herd as a replacement. But if it is a male or an axis female it will be considered a surplus and historically those surplus dairy calves have been sold immediately to the veal industry and is the breeds of those animals are mostly Holstein followed by Jersey so it's a dairy animal that will go to the veal supply and be sold as meat to basically. Any first thoughts on the supply of of dairy calves to the veal production?

Participant
I don't have anything to do with that so I don't really know veal farming. I don't much like the idea of animals in confined solitary confinement if they're in those little [phone statics]. Animal welfare thing. I think that that's I don't think that's their natural way to live. And I don't know anything firsthand about it. I'm afraid. I do not have have any clients who do that I'm not aware of anyone raising any significant number of calves like that around here.

Researcher
It is not it doesn't get to be linked or associated in any way of your with your business. Right.

Participant
No, we don't have any clients that raise animals.

Researcher
Yeah, yeah. And you mentioned the welfare aspect of raising those animals. And as I mentioned, for the dairy farmers, those surplus dairy calves, they are an excess. So they're not part of their core business, which is to produce milk. So they may end up being a problem that they need to solve, like what to do with those animals. So it has been reported in some studies from our group, even, that the surplus dairy calves, they don't receive the same standards of care as the females that will stay as replacement in their herds. One example is receiving half of colostrum or less milk in the first days of life while they are in a dairy farm. And as someone that also raises calves, but in a different way. What are your thoughts on this issue on the management of those calves and basically the overall problem of surplus dairy calves?

Participant
Well, I think if they cannot [phone statics] release this publicly I don't like the idea that there's [phone statics]

Researcher
Oh, sorry. You're cutting off?

Participant
Yeah, I've been missing some of what you say too. I think if if the public can't see what they're doing [phone statics]

Researcher
Sorry, I missed you again.

Participant
The public has to be able to see and accept it. And if they are raising calves individual hutches are raised that would not stand up to public scrutiny. [phone statics] Then well it would tarnish the reputation of beef for everybody.

Researcher

This would have a consequence to the beef as well?

Participant
I mean, by that, I mean, why don't mind if people are willing to come and see how ranchers raised their animals? We would be glad to see it. I would have to probably explain, you know that there's one day a year where it's not so fun when they get branded. But we're doing things to mitigate the pain of that. And it's for their mostly for their own good that they're getting vaccinated and treated in a few of the ways that are uncomfortable. But I don't think that I guess I don't know enough about how veal calves are raised to have a detailed opinion except that I know that the public would not like the idea that a calf is taken away from his mother on the first day and then put in individual Hutch and has no [phone statics] at all and has a short and unpleasant life.

Researcher
My next question is actually exactly about that, specifically about the cow calf separation, because that's an increasing, emerging, concern for the dairy production. It is a big thing in Europe already, but it's still in Canada is, is having a slow start. But separating, as you mentioned, separating the cat from the cows soon after birth is not something that is well approved by the public. And this practice does not happen in the beef production at all. It is far removed from the natural conditions for cattle. I think you already touched a little bit on that. But any other thoughts on this practice? And how do you feel about it? And how, what will be maybe your advice for dairy farmers on this issue?

Participant
Well, I, I guess if they can just produce the females that they need to repopulate their herd. That would seem like that would be a better way to do it. But they probably produce too many then eh, I don't know the sort of the economics or science of that. But they probably don't need as many replacements as they have. Like every year, the cow does not need to replace herself.

Researcher
Yeah, the average in a commercial dairy farm, they want a cow to produce one calf per year. So which means that many of these calves will not be needed to stay in the herd. So those are the surplus, but also even the females that they keep in the herd, they are still separated soon after birth. Even if they stay on farm.

Participant
Yeah, I realized that because the milk is too valuable. I don't know what an answer is. If if there was enough incentive to say that's unacceptable, then maybe they do like we do here with our nurse dairy cows. One cow has four or five calves on it. And then she raises them. But I can say they don't do well. They don't look like a healthy, great cow. Because there's so skinny.

Researcher
Yeah, the foster cow in this case. Yeah.

Participant
Even though they're getting as much to eat as, as they want.

Researcher
Yeah, it's it is a trick scenario that we do not have an answer yet. But yeah, maybe based on what you said, I feel like you will need to have an alternative sometime soon.

Participant
No, or maybe there doesn't have to be one cow for? No, obviously not one to one, but maybe not even one to four. Maybe you just have a few mother cows in with the calves and they can still get their other feeding, which I guess is done by some kind of automatic bottle feeding. I don't know the details of how they feed those. Those replacement heifers. Yeah, they would just be nursing.

Researcher
Yeah, there's a lot of research on the amount of labor because the these heifers, the calves they need to be either bottle fed, or the dairy farmer needs to purchase an automatic feeder that is not cheap. So there's also this dependence on technology for example, to to, to buy those products, those equipments. But yeah, in the third group of animals that I'm personally most interested on are the dairy beef calves. So we talked about how the veal industry has historically been the primary market for those surplus dairy calves, but the view consumption is declining over the years. And then this resulted in a lower economic value of those surplus dairy calves in especially in some regions of Canada.

Participant
The veal calves is not so desirable.

Researcher
Yeah. So like consumption of veal is decreasing with time and then consequently, the economic value of those animals to supply for veal production is decreasing as well. So to overcome this, dairy farmers, they started to use the beef genetics in part of their dairy cows, as a way to increase the economic value of those surplus calves. So in this, let's call it a dairy beef system, the calf don't enter the view market, but rather they are slaughter at the same age as the beef calves that are raised in beef farms, like yours, for example. So a possible pathway of these calves would be they are born in a dairy farm and they are removed from the dam soon after birth. And then they are transported out of the dairy farm to maybe a nursery farm, then potentially a grower, a backgrounder and then a feedlot. And then they will reach the slaughterhouse the same way as beef calves would do. So this would change how much supply the beef has from dairy, potentially increasing over the years. And we would also result in making it harder for everyone to differentiate basically, where those animals are coming from, because both dairy reared and be reared cows would reach the market in the same way. Any thoughts on all of that?

Participant
Well, I guess, selfishly I would be able to distinguish that my, my animals, the meat that comes from mine was not raised in that way. And so I think that there is a lot of marketing of labeled beef, know that you know where it's come from. So we have people who buy directly from ranches, but that's not a majority of people. And that's not going to be what you're going to get a big supermarket or or Costco. I think it would it be another force making ranch raised and sort of certified beef more desirable,

hopefully, within that good economic premium. I don't suppose the dairy people would like that, though. They're going to be by implication. A lesser kind of meat.

Researcher
Yeah, yeah, I guess one thing that we hear from the very side is that they don't want to be involved in the beef production, they just want to have an out of those calves that they cannot keep. But they also want to make it feasible economically. So for example, when the veal prices are not worth raising those animals for even a few days, common practice is to kill those calves soon after birth. Because the cost of rearing them is not worth the value that they get from them. So the use of the beef genetics is a way of finding a pathway. But I don't know your thoughts on this, this issue basically that they face.

Participant
Well, I don't know that it would necessarily be inhumane, I hope not. But I think it would be a public relations mess, you know, with the public. Got to know that okay. These many calves are are born and just hit on the heads right away. So I think there'd have to be some, I hope there'd be some better way to handle that.

Researcher
And in the in the aspect of the dairy farmers don't want to rear those animals? How do you see maybe a future with more and more dairy, beef calves coming from dairy farms? Do you think those animals have a place in the beef supply?

Participant
That I think there would probably be some trouble associated with that. I hadn't really thought about the fact that most people I think, would like to think the animals had a decent life. And if they knew that they were separated at birth and raised in a sort of factory kind of setting. That's not going to be good for the beef marketing. If that's the same beef as what comes off of a place like mine.

Researcher
For the public, the image of the beef production to the public, that could be negative.

Participant
Yeah, that is a negative, frankly. People already don't like the idea that, you know, the animals were in a feedlot. And so we sort of play up the fact that well, that's only the last part of their life. And most of the time, most of that, like, we hope it's a natural enough setting. And I, again, say it isn't a significant amount of my sales. But I do sell meat separately. And people liked the idea that they know where it came from.

Researcher
Yeah. And one thing that I've been talking about is, I'm mainly talking with people that run cow calf operations. But do you think that people who run feedlots would have a different opinion about this?

Participant

Yeah, I imagine they would. I mean, they probably are finding some way to make their own conscience content with what they're doing. And if they run the feedlots well, the animals are comfortable and well attended, and certainly well fed. And I guess kept in there for a minimum, then. I'm not sure how they would view this, they probably wouldn't ser it different from what they're doing intensive farming because I think intensive agriculture is similar whether pigs or chickens or, or I guess, dairy cattle, too. I mean, the animals are not living their natural life. They're, they're confined of concentrating [phone statics] to work as part of the machine.

Researcher
Yeah, one of my personal perceptions is that the feedlots are a little bit, well, way more similar to the dairy operations than the cow calf. And a fear that I have is that this supply of dairy to beef may cut it straight from like supplying from dairy to those feedlots. And then the animals will have a full life in intensive systems, if this ever becomes economically viable and practically viable. That's a fear that I have. But I'm still learning about this. I don't know. I don't know how the future will look like. But I don't know if that that is a reasonable fear or not.

Participant
Well, I think that does make sense. I cannot say that I know it for a fact. But [phone statics]

Researcher
Sorry, I think I lost you.

Participant
[…] to go to some feedlot somewhere.

Researcher
Oh, sorry. I didn't I didn't get any of what you just said.

Participant
I agreed. I think that [phone statics] do a feedlot. And that would be the only way that I think they could be handled.

Researcher
And yeah. Any thoughts on maybe opportunities that this could bring to be farmers on a more positive side?

Participant
Well, I think If we can distinguish our animal kindness. Sorry, I'm having to shoot a dog out of here.

[…]

Researcher
Oh, all good. I asked if there could be any positive side of this any opportunities to beef farmers?

Participant
Oh, yeah. Okay. Being able to distinguish ourselves as providing the beef that has some naturalness to its life would be appalled [phone statics] . But that's marketing. And then that's also sort of pointing the finger at other types of beef and saying, You better look where your beef is coming from and ask for label origin, which I know is a slightly contentious thing, because that's been used in the States to work against Canadian exports of beef, because they want to say that it's American raised, and if it came from Canada, that's not the country of origin that they want.

Researcher
So the positive will be maybe a premium for the animals that are raised on cow calf operations, let's say?

Participant
Yes.

Researcher
And in Yeah, there's another practice that I just recently learned off. And it's definitely new, it's not something that is happening. But maybe a possibility that to address the excess of surplus dairy calves. Some dairy farmers are interested in using embryo transfer, which would be having 100% Beef genetics embryo in a dairy cow. So the calf born would be 100% for beef, and the cow could go back to the dairy herd to produce the milk.

Participant
Yeah

Researcher
Any thoughts on this?

Participant
Well, that sounds like another sort of undermining of what us ranchers are producing, because you really couldn't tell them genetically or any other way. What that was, but it would still have to be raised in a more intensive way.

Researcher
Yeah that's the issue. So these approach has been developed to overcome the challenge of the dairy-beef genetics being of lower quality for meat than full beef genetics, but they still have the challenge that the milk of that cow would go to dairy. And then these calf doesn't have a cow to rear them and doesn't have maybe a structure to be raised in the natural or

Participant
Comes to my mind here, then [phone statics] I have worries in my mind that I was not even aware of. So.. or concerns. I hope that were not a problem for you, when you present your data that, you know, ranchers that you spoke to, weren't even aware of what the dairy people were up to. And now that they are they're worried and annoyed.

Researcher
Well, that's that's exactly what I'm looking to find in my interviews, because that's, I think what is happening. Beef farmers don't know of any of this. They don't even want to talk to me because they feel like they cannot contribute because they don't know anything about the topic that I'm researching. But that is exactly a result that I want to report that the dairy side is making all those decisions and developing all those technologies, but not even communicating with the beef people their views on that. So my goal with this research is to give voice in bring awareness for the farmers and then publish what do you all think about it and how you think you should be taken into account and all this.

Participant
Well, I think that's admirable. I hope it doesn't get you some less friendly reception from the dairy people.

Researcher
Yeah, I don't know, that's a good point. I personally advocate for a sustainable pathway for everyone. That also involves the animals. So if the best if the alternative that they are proposing is not the best for beef ranchers, but also not the best for the animals, I don't know if they should invest on it. And I think it's worth for us bringing some data and some facts for them to take that into account as well. With that in mind, any other words related to this that you think I should take into account in my research or that you were not thinking off before or conversation, but now you're, you're concerned about?

Participant
Well, I'm thinking about it, because you have brought things to my mind that I wasn't quite so aware of. I think the sort of transparency of what people do is what should drive things. So if farmers are doing something or other that they don't want seen. And they're trying to disguise it somehow or other I think that's going to be a problem when it when it is ultimately revealed. Now, some of the things that have been, you know, people breaking into farms and filming things that is not accurate, is taken out of context, or, you know, they were, I don't know the whole story of it. But I've heard that some of those animal rights people breaking in, we're filming things which were in the sick pen. So obviously, they were the worst cases. And so it's not really representative of the, the main healthy line of the animals.

Researcher
I think in in expanding in a bigger scale, any kind of unpopular practice from the dairy may reflect on the beef then. If this line between the two industry becomes more and more narrow.

Participant
Something that comes to my mind is from [NAME] farm where they believe that animals should be transparent and viewable if people want to see it.

Researcher
And you feel you've shared before, did you feel comfortable with that with people coming to your farm?

Participant

Yeah.

Researcher
And making questions about it, but maybe not. That's not the case for all dairy farmers?

Participant
No, I suspect not.

Researcher
Yeah, we went over all my questions were made mostly but just chatting now, I believe. Which is great, which is what I wanted to do and what I aim to do in all my interviews, I have some questions to go over. But it's mostly a conversation. And as we briefly talked about this before, I just like to bring awareness to beef farmers and basically explore your views that I think people should take into account. So this is basically an opportunity for you to share. And for me to put your voice out there basically.

Participant
No, I think that's a good thing to do. Is this going to be part of a degree for you? Are you working on a PhD? Or is this postdoctoral or?

[end of interview]

INTERVIEW P77

[Verbal consent, introduction, and demographics were removed from the transcript to guarantee participants' anonymity]

Researcher
So I'm still very new to Canada. I'm still learning how both industries operate. And my first question to you on that sense is, how would you describe the relationship between the beef and the dairy industry in Canada?

Participant
For the most part, they're almost two very, they're two very different kinds of industries, they operate in very different manners. The only place where I would say that there's a similarity is in what they call a confined feeding operation. In the dairy industry, the barns are generally what they call a free stall barn. And all the cows are contained within that barn. And that's kind of similar to in the beef industry. The majority of the animals are raised in a feedlot as opposed to being out on a pasture or anything like that. Other than that, you know, depending on whether they're a commercial beef operation or a commercial dairy operation, or whether there are purebred breeding programs, you know, selecting sires and that offer the best benefit for you know, return on investment, I guess would be the way to put it. Other than that, I mean, we, we put up our feed, they put feed, kind of more designed towards weight gain. We put up feed more designed towards milk production and longevity and that kind of thing. Housing environments are going to be different because we're asking dairy cattle to live on a basically a concrete within a barn on a concrete foundation. The beef industry, they're more outdoors on dirt and that kind of thing. So, they're very different in that regards as far as the relationship between the two, they both recognize that they are raising cattle. You know, when you break it down a cow is a cow is a cow kind of a thing. But our expectations like certain breeds are bred and designed for milk production more so than meat. And you know, the other breeds are designed more for meat production instead of milk. That's kind of how I look at that anyway.

Researcher
Yeah, definitely. So, there are some similarities, but also some very different aspects of each industry. When I started thinking about the dairy beef relationship in Canada, I looked into the numbers of how many animals from dairy go to beef, for example. And I found that around 20% of the meat production in Canada comes from dairy. And it makes sense after I saw the number because the dairy cows, when they are cool cows, they will they'll go for the beef supply. But have you ever heard about this 20% before? Or do you think that's a good number? Or were you expecting that to be lower or higher?

Participant
Probably, well, when I think about it, it probably doesn't really surprise me a whole lot. Mainly, mainly because here in Canada, we operate with a quota system for milk production. So, if you have if you're on, if you have a dairy, and you've got, you know, you have a certain amount of quota you have to fill and you have enough cows to do that, but you keep having heifer calves. And you end up you end up with more animals than you need to match your quota. So, you end up having to get rid of, you have to cull a bunch of those out. And sometimes you end up calling animals that are quite young, because

they're their pedigrees are just not showing that they're going to make what you need to have in your dairy herd. So, you end up keeping the best animals for milk production. And then you end up with more animals on the lower end of things that get culled out and sent for meat production or beef. Or if you're lucky, you might be able to sell some of those to other dairies. And they might work at the milk cow in their herd. But for the most part, a lot of them end up going into the food chain as meat animals just simply because they're not needed. If we had just an open market for the for the milk production, a person could milk as many cows as he wants and ship as much milk as he wants. And then those animals would be used more in milk production than instead of meat production.

Researcher
I see.

Participant
So the quota system that we have in the dairy industry plays into that, for sure, I would think anyway.

Researcher
So the quota system can influence this dairy beef relationship, do you think?

Participant
Yeah, I would have to think so. Because if I know, I still know an awful lot of dairy farmers, they're all faced with kind of the same thing, they've got more and got more animals than they need for their milk production. So, they, you know, they just any cow or anything that's going to be a bit of a problem, the animal gets shipped out quite quickly. Which, I mean, doesn't sound good. But that's the reality of the situation. And you know, and with advances in things like genetics, that kind of thing is going to happen even more as they develop the breed more, you know, you pick up a high-producing cow and you match that with a bull that's known for producing high-producing daughters, then you put those two together, and then they should get even more. So, instead of a cow that produces say, 50 liters of milk a day, you start working towards 75 or whatever. So then to match your quota you don't need as many cows. So, then you've got, you know, it's just kind of a circle that keeps going.

Researcher
Yes, definitely. And then this system is new to me because we don't have anything similar to that in Brazil, for example. And I also understand that is not the case for beef. Beef is not supply managed, right?

Participant
No beef is not. And I do know some beef producers that, some of them would like to see some kind of supply management system because they like the advantage for supply management. As some people figure, it's just an easy way to make money but it provides a predictable income for the farmer. So, as long as he meets him down to things, he knows how much money he's going to have to work with throughout the year and he doesn't have to try to take a guess on what prices might be or anything like that. And the beef industry, there are some people in the beef industry that would, they would like to have that bit of comfort and security in their operation as well. But there's a lot of people out there that would like to see the supply management system disassembled. And it's not a perfect system. I mean,

there are laws in it which I'm not going to get into. But it is a system, it works. And if you manage it, right, it can work very well.

Researcher
Yeah, yeah. Yeah. I think you covered already that there are some pros and cons that you can work with in the system.

Participant
Yeah.

Researcher
Nice. Yeah. And yeah, we're talking about this 20% in many of the animals that represent this number are the cull cows, but also an important group is the surplus dairy calves. So, you already know that, you already mentioned even that the dairies are producing more calves that they can keep. And then those calves would historically be your dairy genetics. They would go for veal production mainly in Canada. But as you already mentioned, the genetics is making the... Dairies are producing even more calves that they don't need. The veal consumption, there is no indication that it's increasing to meet that requirement. So, they have even more dairy calves that are not needed, not even for veal production. So, to overcome this, many dairy farmers have started using beef genetics in part of their dairy cows, as a way to increase the economic value of their surplus calves. So, this is kind of a dairy beef system, where the calf, it has half of beef genetics, but it's coming from a dairy environment. I don't know if you heard about this practice before or how much you heard about them.

Participant
Crossbreeding with certain beef breeds, actually, I have a neighbor that does that. And part of the reason for doing that is like you said, it's you know, relating to having a mix of beef and dairy on there. The other thing is some breeders they don't have a lot of faith in some of the purebreds, like the Holsteins are not as being strong enough animals and being able to take the environment that they're being asked to live in, so they try to cross them with other breeds that might make them a little more hearty a little more. They'll take a little bit of a hit in milk production in order to have animals that maybe don't require as much veterinary attention, I guess would be the best way to put it. The problem is, like I said earlier, cows, dairy cows are basically living on concrete. So, if you have, you know, concrete is hard on a cow, there's no way no way around it. And the reason they do that is because to be able to compete and to provide, you know, to provide milk to the consumer and that kind of thing. You got to have a fairly large herd and it's a lot harder to manage a large herd outside of a barn area. Now, with the crossbreeding, they're trying to establish animals that can take walking on concrete for a long time and that maybe don't get sick as easily there. They tried to cross them with breeds that are known to have good health traits, that kind of a thing, so... At least that's the way it looked like to me. I always operated with a purebred herd. I never really had any major problems with it. I had individual cows that would have issues, but on the whole, I didn't. I don't know that crossbreeding would have been my choice. But for some guys, they've done it and it's worked well for them. So, I guess it is an option.

Researcher

Yes, I think you cover a little bit what of my next question, which is, uh, what are the main challenges and opportunities that you see with this practice of the dairy beef crosses?

Participant
Yeah, that would be I guess, if you're, if you're producing enough animals that you're going to be selling some of them for meat. If they have some beef blood in them, then they'd be more marketable, I suppose.

Researcher
Yeah, higher economic value. So that's an opportunity there.

Participant
Yeah, yeah, I could see that. Yeah, I mean, they're probably not going to be as marketable as your as your prime beef breeds like Angus and some of the others. But they would certainly have some attraction anyway.

Researcher
Yeah. And then any challenges that come to mind about this practice?

Participant
Because I don't have any real experience in that end of it. Because I've never tried it, I really wouldn't know what problems they ever might have come up with or come across? Yeah, I don't know what kind of challenges would actually be there. As far as that goes.

Researcher
Yeah, that makes sense. And then maybe related to that. Since this is still a growing practice, but it's still fairly new. What do you think are the main questions that you have about it? Or would you like to know more about the dairy beef crosses?

Participant
Me personally? Yeah. I'd be, because I've never really looked into the statistics as far as levels of milk production or anything like that. I've seen very few studies, as far as the health traits, you know, whether the beef, whether the crossbreeds stay healthier, longer, or if they rebound from sickness faster or anything like that. I've never seen it. But I would be kind of curious as to especially if I was still in the industry, I'd be quite curious as to whether or not things are working the way that those breeders had hoped that they would.

Researcher
So their health resilience would be something to look into?

Participant
Yeah, yeah. Because that can certainly be. I mean, that's the same with just about any kind of livestock that you raise, whether it's dairy, beef, horses, pigs, sheep. You want the animals to be healthy because sick animals cost you money. And so yeah, if they're having success with the animals being

healthier, then money that they're not spending on the vet bills, is basically money, staying in the bank account from you know, what they might lose, and milk production they will gain and not having to spend at the vet, is what I would hope anyway.

Researcher
Yeah. So, and then also how they compare maybe with the pure dairy, and also the pure beef, in this sense, to justify the crossbreed.

Participant
Yeah, that's. Yeah, it'd be interesting to see what those numbers are actually. I'm sure that the research is out there somewhere. But I'm not sure. Because the crossbreeding thing has been talked about, I would say probably the better part of 20 odd years. There are I know of, well, like I said, there's one that's not that far away from me. And I know of another one in Saskatchewan, that I think is still doing it. But I don't know of any records or research that has been done. I'm sure it's out there somewhere. But I don't know. I don't know of any myself.

Researcher
Yes, it's definitely something that people are investing research on right now, especially in North America. But there are other countries that have been doing that for a long time like Ireland. Because also they have such a small country, and the farmers, they all know each other. So, it's a very close community. So, there's a lot of dairy beef collaboration there. And then he invested a lot on research, but they have a different scenario as well. So, we are we are doing more research in our reality in Canada and US mainly, to try to inform better the farmers about that. So that's why I asked what kind of questions do you have, so maybe I can inform other researchers on? This is what the farmers are looking to know more. Yeah. Amazing. So, one of the things we mentioned is the genetics of those animals. So, the goal with the beef genetics on there is to make those animals more resilient and also increase their economic value for the meat production. But outside of genetics, is there anything about the rearing practices that you think can influence the health of those animals?

Participant
I don't know what a guy would change that way. As far as raising them from calves on up to adults. I'm kind of thinking not a whole lot of that would really be different. Whether you're a purebred or crossbred, or whatever. Personally, I can't see where there would be a difference in there.

Researcher
Yeah, yeah, we asked that. Because if we compare a dairy beef calf that is coming from a dairy farm in a pure beef calf that is coming from a cow calf operation, the way that they're raised is different, as you mentioned, like in the dairy, they're going to be separated from the cow and be housed indoors. While in the cow calf operation, they stay with the cow for many months on pasture. So, we ask how you think those different practices can influence the calf's success in the future of the beef supply chain?

Participant
Well, the calves that are beef calves that are left on the mother for the for a few months and that. Yeah, in the dairy industry, I kind of think that they would still they would probably raise the calves the same

way they would take them off the mother, because they want to get that once that cow calves, they want to get her into the milking lineup as quickly as possible. And the calves will be raised, I would have to think that the calves would be raised the same way that dairy calves have pretty much always been raised because the end game is they're still wanting them to go into a milking lineup. So, you're going to raise them in a way that will allow them to do that. And quite honestly, like dairy animals get a lot more human interaction with them, than cow calf operations do. So, raising them in a dairy environment means they're human contact is every day, you know, multiple times per day. So, there'd be that because otherwise you put an animal into a barn that hasn't dealt with people much, they get scared and they slip they fall they hurt themselves and things like that. So, there would be that. And yeah, you'd have a lot more control over the feeding and all that instead of grazing, you'd have a lot more control over the feeding, the growth rates all that.

Researcher
It's a way more controlled environment for sure.

Participant
Yeah. So yeah, that to me. Yeah, but guys doing, you know, like, out here, the neighbor here is using Fleckvieh as the as a breed that he's crossing with. And I think the majority of us herds probably. Yeah, it's probably mostly Fleckvieh at this point. And but I think, you know, when I drive past this operation, it looks just like any other dairy barn out there. You wouldn't know what breed is in there. So, I think they're may be crossing with a beef breed, but they're still raising the animals the way they would dairy breeds?

Researcher
Yes, exactly. And that's, that's why we ask about this. Because even though the dairies know, providing some animals to beef, they still need to operate on their dairy conditions to make the milk production feasible. So that's what we're trying to identify how can the dairy farmers adapt to their practices? Or how much do they need to change their practices to provide a good dairy beef calf to the beef supply? So, do you think they need to change anything, basically your views about that?

Participant
I think if I was doing that, I would, basically I would set up pretty much two operations. One would be the dairy operation and the animals that I feel are going to be good enough would be steered that way and the ones that I don't, that I would think are not going to make the grade as a dairy animal, I would steer them towards like a feedlot kind of a setup, then those ones would get out when they would be out in that where they would be fed. They'd be fed the same way that beef cattle are fed in a feedlot kind of an environment. I don't think that there would be any cow calf kind of thing where the calf is left on the cow. If a guy was to go that route, they would probably end up establishing a completely separate beef operation from the dairy operation. And if you're trying to work the two of them together somehow. You'd probably the way that I would probably do it anyway would be to try to identify as early as possible calves that might grow up to be dairy animals, and then the remainder would be sent to a beef operation. With genomic testing and DNA and that kind of thing, you can sample the calves, you know, take a hair sample and send it off, and they can determine. With the result of that, you know, they can have a pretty good guess as to how well the animal is going to perform in a dairy environment.

Researcher
Yes, yes, that's a very effective way of identifying those animals. And so you would change the housing and change the nutrition, I would say with this system?

Participant
Yeah, I would have to think so anyway. I think if you're raising animals in a feedlot, like for beef, some of your lower quality feeds could be sent to those and your higher quality your higher quality dairy feeds would be specifically for the dairy operation. And then the feeds that you put up there, maybe, you know, a crop didn't come off like you wanted it to do. Well, you can use that in a in a beef operation, because you're looking for them to put the weight on words. Milk production is a little harder to accomplish with, with feed that isn't nutrient rich, I guess it'd be the best way to put it.

Researcher
Yeah, that makes total sense. And then you mentioned about the cow calf contact, which is something that is very hard to make in the dairy. And it happens on the beef. And then there are some people that think that it's better to keep the cow on the calf for the calf's health. Some people think it's better to separate. What is your opinion about that? And how do you think is the best practice on that system?

Participant
For beef cattle, I actually think that the calves do better if they're left on the cow for a while in that kind of environment. A lot of the times, like the animals that you see on the feedlots are ones that have already spent time with their mother, before they were weaned and then get sold to a feedlot. In the dairy, on the dairy end of things, I know there are guys that have tried to leave the cow and calf together for a little while before separating so that that calf gets a good dose of colostrum and that, you know, to get a good start. And there are some there are some also that believe it's better to take the calf away as quickly as possible and take care of all that yourself instead of leaving it up to the cow. Me personally, sometimes it's an awful lot of work. And I had limited, I didn't have a lot of people working for me or sometimes I was by myself. So sometimes I would leave the cow with a calf for a few hours. And sometimes I would take it away right away. But for the most part, I often thought that if the cow was getting at the calf and can give it a good start, then I could go and get something else done. So, in my situation, it was more a case of what was easier to manage from one calf to the next and it would kind of change sometimes.

Researcher
Yeah, that makes total sense, because there's a lot of labor involved on that too on that practice. And if the cow was a good mother, she's already knows what to do. So that makes sense to let her take care of the calf for the first hours. Yeah. Yeah, so we're talking about some new practices. Now changing to a very new practice that people are doing research about. It's not something that is happening on farm, but it's something that is being considered to address this the surplus dairy cows. That is embryo transfer. In this scenario, the dairy cow would receive a pure beef embryo. So she would generate a pure beef calf and the milk from that gestation would go to the milk production and the calf would go to the beef production. Have you heard about that before? What are your opinions about this practice?

Participant
Oh, embryo transfer has been around since the first one that I remember hearing about was in the late 70s. But not in that particular manner putting a beef embryo into a dairy animal. But actually, you know, I'm actually I'm kind of wrong on that because I know of quite a few dairy producers that have used beef cows and implanted dairy embryos into them. So there's yeah. And because they would have a top grade Holstein cow, and they would do an embryo flush honor. And they would have, you know, say you might get 12 eggs, but maybe they only had three other dairy animals to implant those eggs into. But if they had some beef animals there, they could set them up and implant them into some of the beef animals and get those calves on the ground a little bit faster. So I mean, it's not unheard of. And yeah, if you've got a cow in the barn, and you're not sure if she's going to be a good milk cow or not. And you know, if you're worried that she's not going to be a good milk cow, then you don't really want to have a calf from her. Then she could still carry a calf that can be raised as a beef animal. Then, she calves out, she goes into the milking lineup. And then you find out whether or not she's going to be a good milk cow or not. And so, and then she turns out, if she turns out not to be a good milk cow, then you don't have any calves from her that are going to be a possible problem later down the line. So.

Researcher
So you see some benefits about this practice?

Participant
Oh, yeah, definitely.

Researcher
And any, any challenges?

Participant
To doing embryo transfer? It's generally kind of an expensive thing, there's a risk. Because you never know, whether you're going to get, you know, one or two embryos, or whether you're going to get 20 embryos, or you might get. I remember, we actually did some embryo transfer ourselves here. And sometimes I would get 10 really good embryos that I would implant and I would get lots of pregnancies. And sometimes I would, you know, the vet would jump back in and say, well, we got nine embryos, but only one is fertilized only one viable ones. So I mean, there's a risk, there's a lot of work, because you have to, you know, the cows have to undergo a number of injections with, you know, the hormones that they need to do all that stuff. And there's a timing issue. So you got to be pretty dedicated to it to, you know, to get the good results out of it.

Researcher
So it's a lot of investment.

Participant
Yeah, both in time and money wise. And I mean, ultimately, you're trying to improve your herd. So you're going to try and get as many calves as you can out of your best cows. And so, yeah, there's always going to be a risk with it. But the thing is, if you got a good cow, and you can get 10 calves out of her instead of two calves that are very well, then you're and you're hopefully going to improve the

quality of your herd overall. And if the herd better, then it is more efficient, and then, you know, the whole idea is to spend as little money as possible and get the maximum results.

Researcher
Yeah, so a lot of opportunities. And maybe in the future, the more we advance with this technology, we will know better how to make it feasible for farmers.

Participant
Yeah. like I said, in my texts to you. Some of that stuff moves along actually at a fast rate. Like I've been out of it for 10 years. And I'm hearing about things that they're doing that are like in vitro and things like that, that I never even would have thought of, and it's starting to become more and more commonplace. So, Yeah, the advances and it's on both dairy and in beef. As far as I understand it, anyway. I haven't spoken to anybody about that in particular. Lately, but yeah, the advances they move along pretty fast. The information that I worked with 10 years ago, is bordering on obsolete now. So.

Researcher
Yeah, time changes very fast. We never know. Okay, and the last portion of my questions is about the consumers. So in the dairy side, we talk a lot about the public perception about some practices that happen, that consumers may not be aware of, but they may not approve. One of the examples is the cow calf separation. How do you think the consumers perceive beef production and dairy production? Do you think that's different?

Participant
Oh, that's a very hot topic these days. Farmers in general are faced with a huge kind of a problem where they're being asked to produce food for the entire population. And they have to do it under increased scrutiny because now with things like Facebook and all that, any kind of image gets out there and gets spread across the world in no time at all. And farmers receive a lot of flack, the problem is, the consumer, I think some of them don't care, some of them care an awful lot. A lot of them, they'll see, for example, they'll see a video of a cow that's in distress. And I mean, a cow is a very large, heavy animal, difficult to work with. So there's no gentle way of helping them out of a stressful situation. And somebody will video something like that, and they'll post it on Facebook. And then people start screaming that dairy farmers are cruel to their cows. And granted, not all dairy farmers are cruel, and not all dairy farmers are gentle, I mean, we are all individual in our own way. And you know, you have some good farmers and some bad farmers. But a video like that goes out there. And all of a sudden, you got a whole slew of people condemning the dairy industry, because they see this one video and dairy farmer in general work very hard to make sure that the cows are looked after and comfortable. And healthy and all that. So, the consumer perception is something that even when I was still milking was something that was always at the forefront, we always tried to make sure we did things in a way so that we can show the consumer that we do care about the animals. And we don't necessarily, like every farmer I know would love to have their cows just out on a pasture. But it's not feasible when you're asked to... I mean, here in Canada, we're dealing with what 30, 40 million people. And we have to feed those. And sometimes there's just not enough land base, and that kind of a thing to be able to do pastures and all that. So we try to do the best we can with what we've got. And I mean, that's the case worldwide. Really. Yeah. So yeah, the consumer aspect of things is, it's always going to be there. I've

always encouraged you like anybody that I talked to, that asked me about this kind of thing. And I've been asked a lot, because I have a lot of friends that live in the city. I've always told them if you ever get the chance to go and spend some time on a farm or work with some of the farmers out there, you would realize that they really are trying hard. And sometimes, it's just about impossible to handle a cow in a gentle manner. Sometimes you have to really literally use a tractor and a strap or something to pick up a cow that has fallen. And you have to use something some kind of mechanical means to get her out onto better footing. And that looks awfully cruel. But sometimes there's just no other way to do it. And cows can be extremely stubborn and hard to work with.

Researcher
Yeah, and in those situations can be taken out of context.

Participant
Oh yes, absolutely.

Researcher
And then, you would say it's a risk not only for the individual farmer but also the whole the whole group, right?

Participant
Yeah. Yeah. Yeah. One farmer doing things bad can definitely make it rough on the rest of them. And I know if you ever get the chance if you want to learn about the dairy industry an awful lot, in Red Deer in the spring, usually in March, I think it is, which would be would have been last month. In Red Deer. They have the Western Canada Dairy Seminar.

Researcher
Yeah, I was there. Were you there?

Participant
You went to that?

Researcher
Yes, I was there last month.

Participant
Yeah, that's a very informative seminar. And I mean, they cover everything from raising calves to nutrition, or housing environments, and handling practices and all that kind of stuff. It's just amazing. The technology that's out there a guy can take advantage of.

Researcher
Yeah. As in this year. They also talked about social media on that sense.

Participant
So I mean, Facebook, TikTok, Twitter, all that stuff is so prevalent now.

Researcher
Yeah. And it's good to have the farmers out there as well so they can show their reality and kind of changed the narrative a little bit on those things.

Participant
Yeah, it's an ongoing battle. And nowadays, like Facebook can be a wonderful thing. But I also think that Facebook is one of the worst things to ever happen to people because you know it shows us the best and the worst of everything.

Researcher
Exactly. And I don't know how much you talk with the beef producer, other beef producers, but do you think that those conversations are constant for them as well or is something that is higher in the dairy?

Participant
I think it's a little higher in the dairy because the just because of the buildings that the cows are kept in. For dairy producers, and in the beef aspect of things, so feedlot producers where you have, you know, a small plot of land with, you know, 10,000 beef animals on it, which is very similar to a dairy barn with, you know, 300 cows in it or something. But I think they have the same conversations that dairy people do. But they look at it from a different aspect, their conversations, I would imagine are probably centered more around what they're getting as price per pound on their beef compared to what they've put into them. Because, you know, like I said earlier, they're not dealing with or they're not using a quota system or supply management system like the dairy is. So I imagine their conversations as to how they have to deal with the with the public. There's going to be some similarities, but I think their dairy industry tends to come under a little bit more criticism, just because of the fact that their cows are raised in for the most part in a different, or they're housed in a different environment.

Researcher
Yeah, so it can be taken more even out of context or be framed in a in a more negative way, let's say for the public in that sense.

Participant
Yeah, I would have to think so. I mean, I haven't actually talked to, like, I know, a number of beef producers. But I mean, yeah, there's got to be fewer and fewer beef producers, because a lot of them are getting older. And so they did things in a different way. And their kids are, you know, they're looking at the farming industry thinking we don't want to do that. It's too much work.

Researcher
Yeah, we can see a lot of that, I think in both industries even.

Participant
Yes, absolutely.

Researcher

Yeah. And then that may be related with my next and final question. And you mentioned before about how things change fast with time. Let's say we have a time machine, and we traveled 30 years in the future? How do you think that beef production is going to be looking like in Canada, then?

Participant
That's an interesting kind of a question. Because right now, we're dealing with a kind of a volatile political situation with our current government and that kind of thing and what they want. We're also faced with an ever-growing population of people, I think it's going to be awfully hard to supply enough meat or beef to the people. And you know there's people are looking at different... well, what they want in a diet, you know, whether you're vegan, vegetarian, or whether you eat meat or not, and all that kind of a thing. Oh, I mean, there'll always be advances in crop production to, you know, raising the crops in a way to get, you know, faster growth. And there'll be, I know, a lot of guys, there's been a lot of criticism in both into both beef and dairy as to the use of growth hormones and stuff like that. And a lot of beef producers that I know, are trying to stay away from that, because of the criticism. And, you know, they don't know if it's really to their benefit or not. So they're going to be trying to look for ways to grow those animals out without having to use that kind of thing. But I mean, there's certainly going to be more challenges, it's going to be harder to meet. But, yeah, what's the political situation and we have some governments that want to limit what farmers can do. I mean, if you follow any of the stuff that's been happening in Europe, there's a lot of farmers that are protesting over there, because they're being asked to, basically they're being asked to not farm though that's their livelihood. So that's going to play into how all this stuff continues in the future as well. So I don't know that there's a real easy answer as to how I see that in the industry in the future.

Researcher
Yeah, it's hard to predict how it's going to look like.

Participant
Yeah, I think, you know, the industry. I'm pretty sure, you know, 30 years. Man, the way things advanced so much, I mean, oh, it could be such a different picture than what we're looking at now. I don't even know what a guy could expect.

Researcher
Yeah, but in we cannot see how it's going to look like exactly, but I think we can assume that is going to depend on the economics, the politics, as you mentioned, and then the technology, and all those different aspects will probably have a influence on that.

Participant
I don't see how they can't have an influence; I think there's no way that they would be able to, I mean, some things like every producer that comes along every young producers that comes along for those that are going to be brave enough to try and farm, they're going to try to be aware of the public perception of things. They're trying to do things on the most affordable scale that they can, they're going to probably try and do things on a very large scale. Because, you know, sometimes it's easier to do things on a large scale, because then it ends up being cheaper per animal and whatnot. Yeah, I think, like in the dairy industry, what we're seeing already is back, I would say, in about the early 2000s,

or late 90s, the average size of a dairy herd was probably around about maybe a little over 50 cows, I could be wrong on my dates, but there was a time when the average size of our herd was around about 50 or 75 cows, like milking cows. Now, now, if you ever heard of 100 milking cows, you're considered a small herd, So I mean, you're going to see, I think you're going to see fewer farms. But you're going to see bigger, like the farms that are still around are going to be bigger. That's happening now. And I think that'll continue to happen. I hope that we still get to see the family farms, I think a lot of them are going to be corporate. And I don't know, I couldn't possibly begin to guess any kind of a prediction that way. But I would have to, that would be my guess is that things are going to go that direction, technology wise, is going to make some of that easier. The internet has already served as I mean, you can go online, and you can find genetics like on the other side of the planet, and get them shipped in. And so I mean, it's made the world a lot smaller that way. So, I can see that continuing even more.

Researcher
That is a tendency that probably is going to be in the future as well.

Participant
Yeah, whether it's good or bad. I mean, that all depends on how a person uses it.

Researcher
Exactly. Well, that's great. And thank you so much for all your time. I think this is one of my longest interviews, which is a great sign. And then yeah, in those last moments, I don't know if there's anything else that you think about this topic that I didn't ask about, but you would like to share?

Participant
Not that I can think of I think we've got pretty much covered everything that I figured you might ask, so I wasn't really sure exactly what you were, what kind of information you were hoping to obtain. So and that's why I thought well, you probably wanted somebody a little more up to date on stuff. But if most of the people that you talk to were beef people, then hopefully my information, like most of it is just the way I feel about it personally, coming from a dairy and things, so.

Researcher
Yeah, but that's exactly what I'm looking for. So what I learned from the dairy and the beef in Canada is that they're still very separate in the communication aspect, that there's not much talk happening between the two and with the increase of the dairy beef calves, some communication is going to have to happen at some point. So that's what I want to do with my research in kind of starting those conversations. So you with experience both in raising those calves for meat, but also previous work with dairy, is a great person for me to talk to. So I think that's why we had a very good and long conversation. Thank you for that.

Participant
No problem.

INTERVIEW P79

[Verbal consent, introduction, and demographics were removed from the transcript to guarantee participants' anonymity]

Researcher
And I have been asking people, if you had to describe the relationship between the beef and the dairy industry in Canada, how would you describe that?

Participant
Well, I think the beef industry is much easier than the dairy. Dairy, I mean, you're married to your job, you've got to be there every day. Now with a robot milkers, that's an incredible invention. If I was young, I'm X years old now. but if I was young, I would love to be in the dairy. But get a partner that thinks like you, which is pretty well, impossible, so you have some time off. Dairy is a commitment. Whereas beef, yeah, the cows are on grass. Yes, you have to make hay and all that type of stuff. But you know what? Dairy? Oh, boy. Commitment. Commitment. Commitment

Researcher
Yeah, it's a different way of living even right?

Participant
Oh, yeah. Totally. You're married to your cows?

Researcher
Yeah. And, and I have heard from people that also mentioned that the two industries are not too close, or, I don't know, what is your impression of that? And how that does that work in Canada?

Participant
Well, you're comparing apples with oranges. The Holstein is milk. Yes, I can see that they would want to crossbreed to get a little bit beefier so they could slip your bull calves into that into that beef market a little better. In a Holstein, your feed conversion to pound gain, isn't worth a hoot. It takes so much green. You know, especially when they get bigger, they eat [not audible]. A Holstein can get as big as, you know, fifteen to seventeen hundred pounds to get it to finish. You can finish them. There's no doubt about it. But your feed conversion is way out of whack. If they could crossbreed them to a muscled bull and try to get them to finish at 1500 pounds, it would be much less. Eventually, you'd save, I'm just guessing off the top of my head, well over $200 a head.

Researcher
Yeah, those animals, they were for many years selected for different purposes, right? So now we're trying to mix that and then trying to figure out how to make it work.

Participant
Years ago. in the dairy world, they were even just killing off bull calves. There was no market for them.

Researcher
Exactly, yes, that's something even related to what I was going to ask you. We are seeing this increase in the use of beef genetics on dairy as a way to overcome that issue of having too many calves. So the dairy farmers, they are very interested in it, because it's a way of getting an outcome for those animals and of a higher value because if it is a pure dairy calf, it doesn't have much value. They would sell it to veal production, but there's not enough search for veal right now. So, they need to find another outcome.

Participant
Yeah, absolutely. Right now, we are paying $700 for a bull calf crossbred, and $600 for a heifer calf, that's big money. I know 30 years ago, like I said, they were killing them off, of getting maybe 10, 15 bucks a calf. It makes a huge difference. So even if you would say on an average year that you would get $500 straight through. You got 100 sows straight through just like that. That's 50 grand. That's a lot of money. That all helps the cash flow.

Researcher
Yeah. So, you think it's something that benefits beef producers as well, this practice?

Participant
Absolutely. Absolutely. But, I again, I repeat this, I would go try to go to a beefier breed than an Angus, I would go to a Charolais. But that's just my opinion.

Researcher
Yeah. And maybe that's linked with my next question about that. Regarding this practice, what are the main challenges that you see with the dairy beef calves?

Participant
I don't think there's any challenges there at all. The market is so hot, people would gladly take them. And I would be number one at it.

Researcher
You're very you're very motivated to seek those animals.

Participant
Oh, absolutely.

Researcher
And is there anything else other than the economic or is the economic reason the main one?

Participant
Well, you know, everything runs on money. Unfortunately, that's an unfortunate thing, but it's a fact of life. To feed a straight Holstein bull calf, boy, boy, boy that cost a lot of money. If you could get a crossbred, and get him a meatier muscled bull. Oh, yeah. You I know for a fact you could save over $200. And the quicker they get to market, the better you're off.

Researcher
Yeah. And yeah. So, what I was going to ask you, what are the main questions that you would ask for researchers that are working on that area? What do you think we should be researching about on those animals?

Participant
I would get, I would try to get well-muscled bull. You might have to use a mature cow for that. Yeah, when I say that, that means you might get a bigger calf. But those big Holstein cows, they can poop out those calves like nothing. It's the heifers that you have to be careful with.

Researcher
Yes. The young ones. Yes.

Participant
Yeah. Yeah.

Researcher
Yeah, so definitely genetics is a big one?

Participant
Oh, huge. I would not use a Simmental bull on a Holstein cow. Simmental bull calves can be as dumb as a turkey.

Researcher
It's not worth investing in them.

Participant
No, no, no, no. But Angus is known, when they hit the ground, they're very alert. I can see why they're using Angus I get that.

Researcher
Yes, yes. And your experience with those animals, so you have raised some dairy beef calves. What are the main differences, if there are any, that you notice between them and a pure beef calf?

Participant
There's still quite a difference. You can recognize it on the animal. You'll see a little white on their leg or underneath their belly button. They're still a little bonier. A true beef is well muscled.

Researcher
I see. So, in their carcass, let's say, their body condition and those senses that you notice more.

Participant

Yes, you will see the Holstein build in that animal. They grow well, though, that the growth is good, but it takes longer. If you have a true beef, and you have a dairy Angus cross, you can see it. You'll probably have a bigger carcass, and it'll take a little longer to get to the market.

Researcher
I see. But is it still worth investing in those animals?

Participant
Absolutely. Especially when the market is as hot as it is right now? Absolutely.

Researcher
I see. Yeah. And have you experienced anything related to their health? On that sense? Do you see any challenges?

Participant
The kind that I'm dealing with right now, the calves are very, very full of life. Obviously, this dairy farmer knows what he's doing there. Their sucking is very strong. All those types of things are very important. A calf, you know, when it's born, it's got to hit the ground running and looking for milk. Nutrition is a huge thing, always.

Researcher
Yeah, so genetics is a big one, but not genetics alone, let's say, is not enough. You need to have that nutrition there.

Participant
That's exactly right. Genetics and nutrition work together.

Researcher
Definitely. Yeah. And yeah, I asked that because there are many things that are different on dairy compared to beef, especially like the cow calf beef operations compared to dairy. So, in the dairy they separate a calf from the cow soon after birth. They house them individually and indoors, while in the beef operation, they would stay with the cow for many months and on pasture. So how do you see those practices having an effect on the calf's health and performance in the future?

Participant
I'll tell you what. The way [wife's name] and I feed these baby calves, I can keep up to pound per day gain as well as a calf that's on a cow. If your nutrition is right. Yes, people say, you know, keep the calf on the cow. If you've got a poor cow, take that calf off. Because sucking a dry tip doesn't do you any good. Just pull that calf away and feed it well. Nowadays we have very, very good feed. Now, you can make a beef dairy cross grow as fast. If you take it away from the cow and feed it separately, you can make it go just about as fast as if you have a beef calf on a beef cow.

Researcher
You just need to do it like in the proper way.

Participant
Oh, absolutely. Nutrition, environment. All that is so important. Keep a calf dry. That's another thing with these calves. Bedding. Bedding is huge. Keep it dry. That type of stuff.

Researcher
Yeah. Yeah, I was going to ask you like, if you had a friend that is a dairy farmer who comes to you saying that they want to sell their calves to the beef supply. They have the dairy beef calves and they want to sell them. What would you tell this farmer that they need to do with these caves in the first days of life that they're under their care?

Participant
Oh, colostrum. They've got to get that first milk. Yeah, that if they're missing that first milk, you're wasting your time.

Researcher
I see. That's the first step?

Participant
That's number one is that they get their Mama's milk. And I'll give you an example, when we buy these calves from this dairy farmer. He always gives us three, roughly about three gallons of colostrum.

Researcher
I see.

Participant
We like to, you know the calves are about a day or two old. I like to keep mixing that and watering it down with normal milk. Colostrum is so important.

Researcher
And then what is the second step then, after colostrum?

Participant
Proper care, just proper care and be very, you know, if it's a small, tender calf, you'll feed it three times a day. They adapt to getting fed twice a day pretty quick.

Researcher
Yeah, I was going to ask you What is the nutrition protocol that you use with those calves that you see that works very well.

Participant
Keep the pen dry. Shavings are crucial. Try to get him on that task. Quick feed as quickly as you can. Usually after about 10 days, they'll start to nibble a little bit on that type of stuff.

Researcher
Yeah. Okay, amazing. Yeah, this early life management is very important, then.

Participant
Oh, huge. You become that calf's mother. When you've got that calf drinking from whatever you have. You become their mom. So you got to... Nutrition is number one. It's like feeding a baby human. You feed them Pavlov. You don't feed them puff weed squirts. Nutrition is number one.

Researcher
Take good care of the calf. Yeah. Yeah. And then the next questions that I have. I'm curious. It's about the consumer perception because we talk a lot about the consumers in the dairy side, because the dairy industry is very concerned about what they think and there are some practices that they may not agree with. One of them being the cow calf separation or housing them indoors. I wonder if those conversations happen in the beef side as well. If you think that consumers perceive beef and dairy differently or not.

Participant
You know what? On TV, sometimes you will hear Angus beef. To me, that is such a joke. It's such a joke. I'll tell you what, we do a fair bit of butchering right off the yard here. And Holstein might be a little bit more bony. But I'll tell you what, you finish off a Holstein, that meat could taste just as good as any beef. But it's not cost effective. So that's why it is so important for these dairy farmers, I agree with that, crossbreed them.

Researcher
Yeah, yeah. And in the end, the consumer is only going to see what shows up on TV or social media, right?

Participant
You know, social media can be the worst enemy. Because you've got to understand, people from the city do not understand what happens on the farm. They don't get that. Why would they? Because they have no experience with it. They go to the store, I think, you know. Go through the dollars. If you got a young couple, they've got a couple of kids. They got a house payment. They've got a car payment. How in the heck can they afford to eat beef now, at the high price that it is? What I don't understand is that four or five people don't get together, go to a farmer, buy a cow and butcher it and divide it amongst them. They could save just about 50% of their money.

Researcher
Yeah. And they already pay a visit to the farm and see how it happens.

Participant
Exactly. Yep.

Researcher

Yeah, definitely. Yeah. So, I can see that. That is not only a concern on the dairy side, or it's something that is maybe for all animal producers.

Participant
Yeah.

Researcher
Yeah. And then we talked about genetics before. And one of the negative or one of the challenges, let's say, with those dairy beef calves is the dairy side of it, regarding their genetics, for the beef production. So there's one practice that people are doing some research on and is not feasible right now to do on farms. But it may be an option in the future, which is to do embryo transfer of a 100% beef embryo into a dairy cow. So, that dairy cow will produce a beef calf, the cow would go to the milking herd to produce milk, the calf would go to the beef supply as a pure beef calf. Have you heard about that before? What do you think about it?

Participant
I think I think that's incredible. Yeah. Oh, absolutely. I think that's better than even crossbreeding.

Researcher
Why is that?

Participant
Because then you've got a heck of a good calf. Oh, absolutely. Now, how expensive is that?

Researcher
Yeah, that's the main part that people trying to figure it out, because this is a very expensive practice, and that's why it's not feasible in the large scale.

Participant
Okay, but can you give me an example of that from a semen from an Angus bull to an embryo transplant? What's the difference on the dollars on that?

Researcher
I'm going to take a note on that, to my list of things to get back to you. So, what's the price of the Holstein bull calves in BC. And the difference in price of the embryo and the beef semen?

Participant
Yeah

Researcher
Because I don't know exactly the numbers in amount of dollars. But that's something that I will definitely have to look up for my research. So, it's great you asked me. The one thing I know is that, in addition to the economic cost, it's also very labor costly. Because you need to have the right personnel to do that. On the cows, you need,

Participant
Then, you need a technician and a vet. And a lot of dairy farmers probably, if they're just going to do a beef dairy cross, might have an Angus bull hanging around. So, they come and eat, or they would AI themselves. Yeah.

Researcher
Exactly. And then that's one thing that the beef semen, it's cheaper than, for example, the sexed semen from dairy. So that's an attractive option for dairy farmers economically, while the embryo transfers definitely going to be more expensive. And that cost may reflect on the price of that calf for the beef producers to buy as well.

Participant
Yeah, yeah. And then if it doesn't catch, then you got to redo it. And you got to make that cow, you got to needle that cow to make it cycle.

Researcher
Exactly.

Participant
There's a lot more work to it. No, I can see that. Yeah. Yeah.

Researcher
So that's one thing that I wonder is how much benefit this embryo transfer would bring in compared to the dairy beef calf. Maybe there is a way, as you said before, with the right management, with the right housing, the right nutrition, that maybe the dairy beef calves will perform well. Maybe there is no need to have 100% genetics. I don't know what do you think about that?

Participant
Yeah. I get that. I get that. Yeah. Everything has to work out business wise. Yeah, the bottom line is, how efficient and how much money does it cost?

Researcher
Yes, yes.

Participant
Most people do not like hired men. I don't. I try as much as I can to do everything myself.

Researcher
Yeah. Yeah. Because there's the cost of labor, and yeah, all those extra layers that come with that.

Participant
Not only that, when you hire people, you hire their problems, and life is complicated enough without running somebody else's problems.

Researcher
Yeah, that makes sense. I can see that too. And then. So, with all the things that we talked so far, at least I see this practice of having this dairy beef calves only increasing in the future. Because what I'm hearing is that it's interesting for the dairy farmers. And it's also interesting for the beef farmers. And now the question is how those conversations should happen? Who do you think maybe should take the lead on the discussions about this practice? Do you think it's something that the dairy industry should be responsible for? Or should the beef industry take responsibility?

Participant
I would put that on the dairy.

Researcher
And why so?

Participant
Well, because really, the beef man doesn't care. He just has his beef herd and why would he worry about the dairy guy. To create more income for him, I think it would be wise that he gets across.

Researcher
Yes, yes. Is there interest, right?

Participant
Yeah.

Researcher
Yeah. Yeah, there's something that we have been talking for a while that the dairy farmers sometimes see these animal as just a surplus. It is a consequence, and sometimes they don't see why invest in those animals. So, that has been a challenge to, kind of, convince them to give the right amount of colostrum, give the right amount of milk. So that's why I made this question that it is important for the dairy industry to see that they also should take some responsibility on those things. And who else should be on the table for those discussions? Do you think? So, dairy is taking the lead, but who they should include on those conversations to guarantee that it's a sustainable practice in the long term?

Participant
I tell you what, dairy men don't need help with that. They know dollars and cents.

Researcher
I see.

Participant
A dairy man is usually a fairly wealthy man. And I know they carry a fair bit of debt too. But dairy men don't need help on that, of decisions like that. The bottom line is a strong point of a dairy farmer.

Researcher
They have the resources, let's say for those things.

Participant
Oh, yeah, no, I don't think they'd need help with that. It's all dollars and cents.

Researcher
Yeah. And then why do you think are the main messages from a beef industry perspective about that. So for now, what I got from our conversation is invest on the right genetics for those animals, and then make sure that they have the right management. Anything else that the dairy side should take into account that they could learn from the beef?

Participant
I think dairy men are ahead of the beef men, as far as nutrition. And all of that goes. Dairy men, dairy farmers, are doggone good farmers. They're very much on top of feed, nutrition, all that type of stuff. Because if they haven't got proper food for that cow, that cow is not going to milk. So, I would say the dairy farmer. How do I word that? His status is probably a little higher than the beef guy. Because of confinement and nutrition. Nutrition is so, so important. Yeah, and they're right on top of that, because that cow's got to milk.

Researcher
Yeah, definitely. And you mentioned confinement?

Participant
Confinement.

Researcher
I see

Participant
Where they're confined in a barn. Cows nowadays are, you know, they walk on slatted floor. So they're very confined. So they're actually kind of a robot. You feed them doggone good food, and you get milk out of it

Researcher
It's an efficient system then?

Participant
Oh, efficiency is number one.

Researcher
Yeah, yeah, definitely. Okay. So, we are reaching into 30 minutes, but actually, we passed 30 minutes. And I have one.

Participant
If we're close to half an hour, let's shut her down for today.

Researcher
Oh, we're. Yeah, I just have one question left. So that's perfect timing.

Participant
Okay.

Researcher
Yeah. So my final question is, let's say we have a time machine and we traveled 30 years in the future. How do you think the beef production is going to be looking like in Canada?

Participant
Oh, boy. That's a loaded question. I don't know.

Researcher
Yeah, I saved it for last.

Participant
If we looked back. 50 years ago, if you had 100 cows, you were huge. Today, there's five, six thousand cows, you know, in a group that calve. It has so changed. Technology is unbelievable.

Researcher
Yeah, we cannot even imagine, right?

Participant
Well, it used to be family farms. You know, mom and dad, and they had the children help them. Oh no, now it's big corporations. That has changed so much. Is it a good thing? I don't know. I don't think so. I think the family farm is still very important. Where dad and mom, you know, work together with their children. And today, yeah, there's good money in beef. But you go back a half a dozen years ago, holy man. It was it was a different story. I would say 30 years from now, if it changes the rate that it is now. I don't know if it's favorable.

Researcher
Yeah. So maybe not such a bright future. Maybe a little bit-

Participant
No, the future, I'm not saying the future is not good, but it's just different. Yeah. People worked for big corporations. You haven't got that personal touch like you used to have.

Researcher
Definitely yeah. Yeah, I think it's very hard. I know it is a hard question. That's why I save it for last.

Participant
It's a loaded question.

Researcher
Yeah, we cannot even imagine, but I just curious on what people think it may look like, but most people have the same answer as you gave me.

Participant
Yeah, yeah.

Researcher
Amazing. Thank you so much for your time. We already covered everything. Anything else that I didn't ask you, but you would like to share about that topic?

Participant
Well, I hope we talk again.

INTERVIEW P81

[Verbal consent, introduction, and demographics were removed from the transcript to guarantee participants' anonymity]

Researcher
So I think my first question for you would be if you had to describe the relationship between the beef and the dairy industry in Canada, how would you describe it in why?

Participant
Well, you know, you kind of said it when we first got started. To be honest, I didn't think there was a necessity for it. I really did not think that dairy and beef would ever mix. But with our cow numbers, the beef numbers are being deplenished, and it's not going to get any better. So the only way that our beef is going to be able to sustain.... I'm trying to figure out how to put it. We need the dairy to sustain the amount of meat capacity for our country. It's a tricky situation. And I had a visit with NAME, the other day when I went up. And that was when I gave you THEIR phone number, and THEY and I talked about it. Without the dairy, it's the only way that Canada will be able to sustain its quota for beef in grocery stores, taking it out, exporting it to other countries. I think it's the only way that we're going to be able to deliver. I don't know if being a cow calf operator is going to keep the beef... Is it going to make my my beef worth more? Because it is beef. It's not dairy beef. It's beef.

Researcher
Yeah.

Participant
Does that make sense?

Researcher
Yes, yes. It makes sense. And I was just wondering, why do you think we are in a situation right now that the beef needs support from dairy to meet its quota?

Participant
Well, because for one thing, our water and the drought and the fires have deplenished the amount of grasslands out there.

Researcher
I see.

Participant
That people are not one able to make enough hay. Cows are too expensive to put expensive hay into. Does that make sense?

Researcher
Yeah, yeah.

Participant
It costs a lot of money to feed these cows. Yes, we're getting high prices for our cows. But does it still justify? When you can sell... It cost $4,000 to buy a red cow now. Okay, yes. I can get maybe $2,500 for my calf, but hay also cost 400 bucks a ton. And grain I have no idea. So is the sustainability there for us to be able to... Okay. Five more cows? Yeah, no. So I can't replenish my herd. There's a lot of variables. And they're wondering, how are we going to improve?

Researcher
Yeah, and economically speaking, one thing that people have mentioned to me before, it's also the supply management of dairy. That doesn't happen for beef. I don't know your thoughts on that. If you have any.

Participant
Yeah, I haven't done my costs on it. For a couple of years, you know, the price of diesel. Everything has gone up in price. And that's why the cost of our hay is so expensive. Now, we're lucky that we can produce enough hay to feed our own cows. But if we had to go out and buy hay, like producers had to, we couldn't do it. We would have had to sell a lot more cows. And we did.

Researcher
Yeah.

Participant
So we're just kind of hanging on. Water is expensive. Hay is expensive. Everything is expensive.

Researcher
Yeah, yeah. And then if you look at the dairy industry, they have some calves that they may not need because their product is the milk. Right? So in the beef side, that's where the animals have value. And maybe that's the opportunity. That's what I'm understanding, that's the opportunity that the beef has, using the animals that the dairy won't need. I see. Yes. And one, one number that I found when I was doing some research on this is that inevitably, animals that are not apt to produce milk anymore, or they're not useful for the dairy and so they will be commercialized as beef in the beef market. And there's around 20% of the meat production in Canada that comes from dairy already. I believe this data is from 2022. So I was just wondering if this 20% is a big number, do you think or you're not surprised as that's what you would expect? And how do you think this number may change in the future?

Participant
Yes. And there are a couple of producers, and we have in the past bought dairy calves from them to put on a cow that's lost the calf. And one year we bought one and he was half Holstein and half Hereford. And that animal grew, like he was amazing. And then we bought one that was straight Holstein. Well, the same thing. He grew and he grew and grew. And it was, it was a good supplementation, because at the time we couldn't find any beef calves to go on the young cows. Well, we didn't want to let them dry out. So that's what we did. But you know what, even those calves are worth. You know, they're almost worth four or $500 to go get a calf from a dairy. So with the beef dairy coming from dairies, they're the

ones that are going to hold the beef dairy supply chain going. That is a question that I can't answer. When you have a visit with NAME, THEY will be able to give you a better idea because THEY take Holsteins from the local dairies. And THEY take them and and I don't know where THEY put them. But THEY will be able to give you that kind of number that you're looking for. Better than me.

Researcher
Yes, yes. And maybe we can talk about how the animals are raised. As you mentioned, when you had dairy calves in your farm, they grew well, no too differently than beef calves. But there are some practices that are very different if we look at how dairy cows are raised and beef calves are raised. I don't know if you have any thoughts on it. On for example, the cows on pasture in beef farms, but indoors in dairy farms. If you think there could be a problem if the dairy is going to supply calves to beef in the future?

Participant
Hmm. I personally, I think if they're going to be raised to go into our supply chain, as long as they're raised in healthy conditions where they can get out and they can run and they can see sunshine and get grass. I don't have a problem with it. But if they're put into a barn, not raised in a veal direction, you know, I think they should be able to be raised outside. I think that's the most important thing.

Researcher
Okay. And why do you think that's the most important thing?

Participant
That's how they grow and you know, their life is not... It's sustainable at that point. It's not just a meat market. What's that mom?

Researcher
Sorry?

Participant 14:10
Happy. My mom just said they're happy.

Researcher
Yes, yes.

Participant
And you know what? So many people, when they come here to buy... We do our own little production. We take some of our old cows and, and stuff. They're pulled out of production. But our cows when people go by our place, and they come in to get a package of hamburger or a roast. They like that they're buying beef that comes from happy cows. Happy cows have a happy life. So why should those calves, those beef dairy calves not be able to have the same thing? They're outside. They're eating grass. They're growing. They enjoy sunshine and fresh water.

Researcher

Yeah.

Participant
There's a certain point of time that they need that for full development. Now, this is just my perspective, because that's how our calves are raised on the cows. They go out onto 800 acres. And they get to move around. They get to go up and down rocks and side hills and drink out of ponds and drink out of water troughs. Why should the beef dairy calves not have that same life? They go through, you know, the last year after they're weaned. They're then put into a feedlot. So I, from my perspective, they still need to learn to live.

Researcher
Yes. And we're probably jumping ahead on my questions. But I'm loving to talk about this that I have two main questions on that aspect. What do you think is the risk for the animal from the farmers perspective for its business if they don't have this opportunity to go outside and be raised as beef calves, let's say? How can it influence on their overall performance over in life? And I think you've already covered a little bit on that. But if you had anything else to mention on that aspect?

Participant
Well, I don't think their immune system is as strong if they don't get outside. If they're inside, whether it's in a barn of a hutch. Is their immune system as strong. So, at that point in time, if they always having to be inoculated for BVD, IBR, P3. You know, the cows always have to be inoculated for that. Ours are. Then, they have got that in their system. But are they truly, are they....? Am I Am I saying this right? Are they... Is their immune system truly as strong? You know, you are the university student.

Researcher
We can try to answer. Yeah. And maybe on that aspect as well. One thing I wanted to ask you is that, I don't know if you're aware of this practice. Can you hear me?

Participant
Yes.

Researcher
Okay. A common practice in the dairy industry is to separate the calf from the cow as soon as they are born. And then the calf is bottle fed or fed by the farmer. And the cow is milked.

Participant
Right then and there. They're not getting... [disconnected] So my question is to you, when the calf pulled directly off of the cow, and they get the colostrum. After that point in time, I don't know what kind of milk are those calves raised on? Are they still [disconnected] Is it a different kind of milk from dairy cows? Dairy cows are fed hay. Beef cows are raised on grass. What is the difference in the milk quality between the two? So our beef calves get [audio glitch]. Do dairy calves do they get [audio glitch]? Do they get lots of vitamin A, D. When we watched our... and we've only done two. A half Holstein and a Holstein calf, they were raised out on the hill, on those beef cows. Well, it was amazing to watch those calves grow and work the hills. Just like the beef calves. So are the dairy calves that are taken off of the

cows and kept in barns and huts or whatever, however they're raised. What are there immune systems like? And those calves are going into our supply chain? I don't know? Because that's where I don't know enough about it. You know, you phone me to get my opinion. Well, I'm asking you questions that I don't know anything about. But if that dairy beef is going to go into our supply chain? And is it going to feed, you know, the consumers. Is it good quality? I'm going to say it's not better than the beef that we put into the supply chain. But is it a good enough quality?

Researcher
Yeah. And even though we may not know the answers, it's already important for me to know that it's something that you believe it's important that we check, let's say. It's something that needs to be considered. Right?

Participant
Mhm.

Researcher
Okay. And you also mentioned about the quality of the product to the consumer. And my second question on what we were talking about how the animals are raised is if you think the public and the consumers perceive dairy production and beef production differently? As you mentioned, your consumers can go and buy meat from your farm and see the animals. Do you think that there's any difference on their opinions about dairy and beef on that sense?

Participant
Well, I mean, I'm talking from my perspective, and like, from my mom's perspective, and our neighbors. When you go down the road, and I'm saying more or less down towards the coast, and you see all those little beef huts, and the calves are in a hut by themselves. That's not healthy. Cows are herd animals. And those calves can't play together. And I know that you guys are doing lots of research on, you know, calves, how they do, whether they're by themselves are in singles or pairs. I know you guys are doing that kind of research. So from a consumer's point of view, when they go by and they see all these little calves in individual huts, how can you say to yourself, oh, that's so cute. It's not. Because from my perspective, I see our calves. When we go out and feed, we see those calves running in groups of 5 and 10. And their tails are up in the air. They have fun. They're a bunch of babies having fun. Those beef calves aren't running and playing and having fun. They are living a solitary life. That's not healthy. Now, a friend of mine that has a dairy up in CITY. NAME is doing it right. You know, yes, the calves come off of the cow. They go into little individual pens for I think five days where they get their colostrum and then they start milk. Then they go into another pen. The heifers are separated from the bulls and the steers. Those animals get to play. They become herd animals. And the calves get to go into this little room where they can drink milk on a free choice basis. I'm impressed.

Researcher
That's a good system.

Participant
The way he is doing that.

Researcher
You're impressed in a positive way.

Participant
Yes. But he's also got a small dairy. I don't know what.. I don't know if he's got 50, 60 cows. I don't know. But to me, that is the right way to do it.

Researcher
Yes. Do they? Do they have access to outdoors? Or pasture?

Participant
As they get older? Yes. But in the springtime, he keeps them confined because he doesn't want them... You know, they're kind of in a low area. You know, they can't, they don't, he doesn't want them to go out where they're gonna get wet or anything.

Researcher
Yeah. Yeah. Maybe on that note, one of the questions that I had was, let's say you have a friend that is a dairy farmer, and maybe could even be that friend. And day one, they decided to start producing dairy beef calves to sell to the beef supply chain. What would you as a beef farmer recommend them to do in their dairy farm? So those animals can be successful supplying to the beef

Participant
They got to be able to get out on grass.

Researcher
Okay, that's number one?

Participant
They have to get out on grass. They have to get out and enjoy the sunshine, fresh water. You know, to me, those are the most important things. Fresh air, grass and fresh water.

Researcher
Yeah. And you mentioned about the nutrition with the milk before as well. Yeah.

Participant
You know, so if this person is going to do beef dairy supply. Now, of course the calves are going to need milk to a certain point. How is he going to, how is he going to give that milk to them? You know, because a calf ideally needs milk up until at least preferably five months old. I mean, yeah, we've had baby calves here that, you know, they get milk replacer up until about until we get sick and tired of feeding them. Because they butt the milk buckets and send the buckets flying and you get mad at them and everything. You can't help but laugh. You know, we try and keep milk in front of them at least to fifth month. So how is your friend going to at least give proper nutrition to those calves to at least five months of age?

Researcher
And that's not what happens on dairy. Where the calves are weaned way earlier that than. Yeah let me go over. We definitely didn't follow my order of questions. But that's great. That's what I want.

Participant
Oh well, it is what it is.

Researcher
But it's a good sign that we're just having a conversation. That's what I hope. Okay, I found one question that I didn't ask before, which is basically with everything that we talked. What do you think that this practice of having those dairy beef calves, so using beef genetics on dairy cows, what do you think it will mean to you as a beef farmer in the future or to your industry? How do you think you can impact on your business?

Participant
Well, I was thinking about that after I had a visit with NAME, and the only thing that I can think of, and I did say this to you. Is it going to make my beef, off of beef cow... Is it going to make that beef worth just that little bit more? Are my calves worth more than dairy beef?

Researcher
I see.

Participant
If dairy beef comes in at the same price as my beef, I won't be happy. But I don't know. I don't know how it's going to impact. At this point in time, it's too early to tell. When you have a visit with feedlots, and you find out their answer, let me know.

Researcher
Yeah. That's what I heard from other cow calf farmers too on that. They are aware that their views may be different from those who run feedlots. Because you have different kinds of interest. Right? My suspicion is that the feedlot people will be more happy or interested on purchasing animals for maybe a lower value. But then, as you mentioned, what is the quality of these animals? Right?

Participant
Exactly. Now, NAME did say to me, you know, a Holstein, when they're fed out, they actually have a different type of fat content than regular beef. And I was like, Oh, really? I did not know that.

Researcher
Yeah, and there's a lot of research now also looking into the best mixes of dairy and beef rates to try to get good quality meat. So yeah, my interpretation is that we don't know yet. If this is going to be an opportunity to value cow-calf calves, or a challenge. Maybe they will risk and compete with you. I don't know, what you think about that?

Participant
I don't know. I mean, we're getting into something that I don't have enough of an opinion to discuss it. Because like I said, I just don't know yet.

Researcher
Yeah.

Participant
And I said, are we looking at 20% of the dairy beef coming into the food value? Or will it end up being more than that? Like I said, our cattle numbers are decreasing rapidly. And it's going to take a long time before it even starts to start increasing. I don't know if it ever will. The US market is deplenished. It's the lowest it's been since, what was the year, 72. Or something like that? They're at 3.8 million cow, beef cows. They've never been that low.

Researcher
Yeah, so that's, it's something that only time will tell. Right?

Participant
Yeah. And I think, you know, with the places that NAME has been, Australia, and Europe, they've got more data than we do. The United States and Canada. I think it's time we really take a hard, hard look at what is going on there.

Researcher
Yeah, we were just now starting to talk about it here. Right. And, I can see that by my own research. I tried to contact beef farmers to talk about it but no one's even sometimes aware of those dairy-beef calves and maybe this dairy beef system that is being established. And it's just a conversation that we may be a little bit late on. But I hope that we can catch up on it.

Participant
How many how many feedlots or how many people like NAME and I have your been talking to?

Researcher
I talked to six people so far. But they are all cow-calf. So I don't have any people from feedlots yet, which I'm hoping to get.

[Participant and Researcher share information about possible prospective participants]

Researcher
Yes, yes. And it's great that, you know, you already talked to me. So you know that it's just a conversation on things that I also don't know the answers to, and we're hoping to figure everything out together. So it's great. The more people I talk to, the better, so I can have more views on on my topic.

Participant
It's coming. Whether we like it or not. It's how do we work with it?

Researcher
Yeah, exactly. Yeah. That's what I'm hoping to do with my research to help both the beef farmers but also helping inform the dairy on what is the best path forward because, as you said, it's coming and there may be multiple ways of doing it. But if we have the information necessary, maybe we can make the right decisions along the way. So yeah.

Participant
Okay, well, did I answer your question? I gave you my two cents worth whether it's worth anything or not, I don't know.

Researcher
It's very, very helpful. I only have to just take a moment to ask you, if we didn't cover anything that you would like to share, I'm happy to hear more on your views. But if you think it's all good, also, if anything comes to mind, you have my contact, you have my number, I'm always a phone call or a text away. And I will send you my final result once I write the paper on it. I'm happy to share. It may take a while, but I'll keep in contact.

INTERVIEW P89

[Verbal consent, introduction, and demographics were removed from the transcript to guarantee participants' anonymity]

Researcher
So that's my first question to you. If you had to describe the relationship between the beef and the dairy industry in Canada, how would you describe it?

Participant
We do a lot of dairy work. We pick up all the Holstein calves, the week-olds off the dairies and go to feedlots in Alberta with them. We pick up all the slaughter cows and a lot of them go to Washington, some we kill locally. And you know, the tougher cows, we just sort them for animal welfare and send them where we can, kind of thing right? We do have a program that we buy for. It's called a P3 program. It's a Limousin-Angus cross with the Holsteins here. You know NAME...

Researcher
Sorry?

Participant
You've heard of NAME? So we do a lot of work for him and he's partners with another gentleman. So they have the program in the US which is a lot of beef cross dairy cows. And we've developed a program here, or they developed it, and we worked with the dairies here to keep it going.

Researcher
So there's a lot of conversation and exchange between the dairy and the beef supply chains.

Participant
Yeah, maybe in the supply chain a little bit. The dairies really don't have a lot of input. They use this special semen, and they get paid a little extra for it. They don't really... I guess obviously with the new sexed semen and stuff, they can breed the heifers they want and then everything else they can just breed to this other semen, and they get paid little better for it, or quite a bit better for it. And don't have to raise the extra heifers. Right?

Researcher
Yeah. So it benefits them in that sense of getting an outcome for those animals?

Participant
Yes.

Researcher
And do you see this as a benefit for the beef side as well to get access to those animals?

Participant

100%, especially now with how the beef herd numbers are in Canada or in North America. If it wasn't for these crossbred calves, there'd be a lot of empty feedlot space. And we'd be feeding more straight Holsteins than... and this is a better product by crossing these in, right?

Researcher
Genetically, those animals are better prepared, let's say to supply to beef than...

Participant
Yes.

Researcher
And, yeah, when I was reading about this supply from dairy to beef, I found data from the Canadian Cattle Association that about 20% of meat production in Canada already comes from the dairy industry. And this data is about two years old now. And considering as we were talking the use of beef genetics in dairy herds, how do you feel about this 20%? Do you think it is number is already what you were expecting? How do you think this will change in the coming years? Will this increase or decrease? How do you think that will change?

Participant
I honestly think it's higher than that. And yeah, I don't think the numbers are there. They haven't figured out what the numbers are, especially with beef numbers going down. Even if dairy numbers go up a little it really increases the percentage. But then there's a lot of cattle that came in from the states. These crossbred cows, they came in at 300 pounds or 400 pounds. I know, lots of trucks that were bringing in. I don't know how many are coming in now. But I bet those are dairy cross cows that will probably be straight in the beef numbers. Right. So. So yeah, I don't think 20% is accurate.

Researcher
So it's like less than what actually happens? There's probably more?

Participant
More than that, for sure. Yeah.

Researcher
And we talked a little about these calves coming from the US. Do you see this increasing within Canada, so dairy farms providing those cross calves to the beef supply in Canada? Do you do still think that those calves must continue coming from the US?

Participant
Well, I think that we're increasing our cross-breed program in Canada. But we still only have a certain number of cows that we milk. So we do still bring a lot of like, I think we're going to slow down on the calves coming in right now. Because our basis between us and the US, we're operating at quite a different price than what the US is. They're getting more and they're paying less for them here, at the moment for finished cattle. So as that number goes up, then like as the basis narrows, and our price gets closer to what theirs is, then we'll start seeing more of them coming again. I think that's kind of

slowed things. But first off, I guess we should go back one step and get you to clarify you're taught that 20% of the beef is dairy or dairy cross. I'd like to know if you're saying that is the Fed market, or just total market that goes into the beef? Like cows manufacturing meats and stuff like that? Like is that just fed market? Or is that everything

Researcher
That will be everything, counting cull cows as well. But as I said, this data is already a few years old. So as you said probably does not represent the reality. And probably, it's bigger now. And maybe in a few years, it may be even bigger, right? Considering the changes in breeding in dairy farms.

Participant
Definitely because of that, and also knowing that like we right now, probably got the highest cull cow prices we've ever had, at this time of year, and it's just gonna get higher. Well, that drags out cows, but there's not enough beef cull cows going forward because they didn't have the feed. They've had some drought years, stuff like that. So like our herd is shrinking. So if we usually have 100 cows come to market, then now work maybe having 40 or 50 cows come to market and your dairy cow numbers haven't changed. So it's now all of a sudden, you're probably 50% cows or dairy or... Like I'm just giving you an example, those numbers aren't true, but that's just kind of what we're seeing.

Researcher
Yes, I totally understand. And considering as we were talking, it's beneficial for the beef farmer to have access to those calves and it's also beneficial for the dairy farmer to have an outcome for those surplus calves. Let's say you have a friend that is a dairy farmer and they are interested in starting to sell those dairy beef crosses. What, you, as a beef farmer, would recommend them to do with those animals in their farm? How can dairy farmers better prepare those animals to supply and succeed in the beef market?

Participant
A few different questions there. Well, first reason that in the States that they became using our crossbred was a lot of times, those Holstein calves are worth nothing. So what they did by starting to use a special semen that these guys were offering, they also signed contracts guaranteeing that the buyer would take all their calves. So now it's in spots in Canada, which is Saskatchewan, Manitoba that's the case. In certain areas, like in BC, there's lots of competition for the cows. So that contract really doesn't matter. Now, the next step is, there's a lot of feedlots. You're talking to beef farmer, I'm gonna call them feedlots. They have capacity in their feedlot pen space for a certain amount of cattle. As our numbers go down, it makes it tougher to buy enough cattle to fill those pens and there's always going to be some empty pens. So by using the dairy cattle or dairy cross, they can fill those pens and keep their operations working more efficiently like keeping the hotel full was always cheaper than half full right? And also by using the crossbred on the ... Right now, what we're using creates a higher grading animal for beef meat grading, lots of triple A's and leaner yields. But it also puts in some efficiencies, makes a better shape. Like it's something that's highly desirable. And I'd actually suggest at one point, if these are bred to the right thing and fed the right way, they're worth as much or even more than some of the beef breeds that are out there.

Researcher
Yeah, and as you mentioned before, it's beneficial for both sides. But how do you see the communication happening? So for example, who is taking maybe the initiative to connect to the dairy and the beef to decide what is the best genetics? What is the best feeding or management of those animals? I don't know if you have any thoughts on what is happening or how this conversation should happen?

Participant
Well, I was in Alberta, as you know, last week, and we did have a sit down with one of the gentlemen that is running one of these programs and we went to him and said look this we're having problems with these programs here. One the birth weight, one the gestation period. Like so he's working with the semen companies that work with the dairies. So they tried to develop the semen that will work best for them genetically. And then the team sales company goes to the dairies and tries to get them to sign contracts and use certain semen. That's how it goes down, basically, at the moment. It's not without issues, like there are some problems, and we're trying to sort them out.

Researcher
So it's like the genetic companies doing this guidance in some way on what is the best breeding management for those calves, for those farms?

Participant
Correct.

Researcher
And then you mentioned some challenges. And this is also related with another question that I had. There are a lot of opportunities that we identify with this, let's say collaboration or this, like communication between both industries. But is there any challenge or some things that we should be aware of moving into this in the future that we could maybe address?

Participant
Well, this is more what each program has to address, but... Well, first off birth weights, they're using some of these Limo genetics in some of it. And some of the birth weights on the Limos are a little heavier and the dairy farmers are having problems with some of the calvings because of the birth weights. Now, this is just one that I've been told. And I'm told it's true that the gestation period on some of the semen is 7 to 11 days longer than some of the more Angus based breeds. So what that does is, it throws out the breeding cycle in the dairy by seven to eleven... Some of the cows that are bred to that could calve, you know, an extra 10 to 12 days late. So they lose 10 to 12 days of their milk period, but in the management, and they bring them into the close up pen, and they hang around, hang around. So they're using the facilities for an extra 10 days that they shouldn't be. So there's all things to get ironed out, right?

Researcher
Some, like details on management and other rearing of those animals, let's say, to make it work. Yes. And then also talking about the management of animals. Some things that we talk in the dairy side,

some practices that we do, basically, they are very different from what is done in the beef farm. So for example, calves are taken off the cows soon after birth, and they are housed individually for a certain time, they're weaned way earlier. How do you see those practices in this merging, not merging, but in this providing of calves to the beef? How do you think this can influence on the health of the animals going into the beef supply in some way?

Participant
Well, first off, the dairies are going to raise them differently than they do their own heifers. Because they don't stay on the dairy they raised there. They get taken and then they'll get picked up within two weeks and sent to a calf ranch, say in Alberta and stuff like that. So obviously, you're going to have a higher death loss than you would if you leave something on a cow forever. I don't know quite how to... It's going to change, obviously, you're probably going to lose between 10 and 20% more calves that way. But then you need the milk cow producing milk. So what are you gonna.. you gonna keep seeing a higher price milk to those if they're in the dairy, so at least they're fed milk replacers in the calf ranches.

Researcher
Yeah.

Participant
Which is cheaper and using different products.

Researcher
And some of the concerns that we have also in the dairy side are the consumer view about those practices. So it's not well received this practice of separating the calf from the cow or some of the individual housing. So we have a lot of talks about it in the dairy side. How do you feel about it for the beef side? Because it doesn't happen in the cow calf operations in the beef farms, for example. Do you see that this can have any influence on how the consumers see the beef production in any way? Can this be a challenge maybe in the future of collaboration between the beef and the dairy?

Participant
Well, it's obviously not an optic of a natural cow running around in the mountains with her calf and that. But it's not... If you want to eat the meat at a reasonable price, there are certain things and we tried to take care of those cattle properly and give them all their needs. And that's as far as I'd go on that. That's if you don't want to eat it because it's taken off. Well, then you're not gonna drink the milk. Because, yeah, you know, then the milk is not in the cow because the cows taking it. So it's all... yeah. There's gonna be those who drink it and eat it and those that don't. So.

Researcher
It's hard to know, right? And then we don't know how much the consumers actually know about the production of animals. We're so far apart. Where people don't sometimes... I've already heard people may not know even the difference between a dairy and a beef cow. It's all cows. So there's also that, but yeah, we wonder what we may see in the future as possible challenges let's say. We talked a little bit about genetics. So yes, we see the use of beef genetics in the dairy herd as a way to improve the quality of the beef produced by those calves. Is there anything else outside of genetics that could be

provided to those calves? For them to be better animals to supply for beef in any way? So I don't know if my question made sense. But in the way of feeding or housing or anything that we could doing the dairy to improve the resilience, let's say if those animals going into beef or on feedlots?

Participant
Well, it's the same thing all the time. Colostrum, and treating that calf right and making sure that calf stays on the farm till two weeks old. You know, not trying to get rid of it at three days old, and, you know, all those kind of things to give it a good start.

Researcher
So they could start being mainly the colostrum, the feeding, and staying on farm for the certain amount of time that it needs to have a good early life development.

Participant
And, yeah, just good animal husbandry. like the same as you'd want to raise your heifers in. Good, clean, dry pens, lots of ventilation, all that stuff, right? Just typical calf raising.

Researcher
Yeah. And I was also wondering about the program that you mentioned the P3. I read a little about it online. But I don't know if you could share your experiences with the program. Like, I was curious to know how it started. And how has it been perceived on the beef industry side, and how farmers are engaging with the program.

Participant
Again, it started with NAME, bringing in from the US. Using similar bowls and stuff like that, that they use down there. There's only a few people here that are that are contracted to be able to buy those calves kind of thing. So they're the ones that are trying to push it forward. It's perceived well, in some areas, and not in others. And guys certainly are using beef cross kind of cattle to increase their viability, increase their bottom line. There's another program called the infocus. It's kind of a competing program to that. It's more of an Angus base than a Limo base. So it's all different things.

Researcher
It's all good for me to know, because I'm still learning about those programs in Canada and how they're being established. So it's great to have these different sources of information to learn more. And I guess we have five minutes left. And I also we cover most of our my questions already, so, thank you so much. And I guess one more question I have is, let's say 30 years from now, how do you think that the beef industry is going to be looking like in Canada? And how this relationship with the dairy industry will be looking like as well? How do you think?

Participant
There's only one thing in the beef industry that stays the same, and that's change. So I really don't know. There's always going to be change. As soon as everybody's on one page, then all of a sudden, there becomes a niche somewhere else to to change things. Right? So it's like we had Angus, and Angus was the only thing to grow. And now we've got so much Angus that now we have to start looking

at a little differently and producing different animals. So I think that the two will work together for a long time because I think as our herd shrink, the dairy is going to be very important part of the beef production. So.

Researcher
Yes, […] this is not the first time that I hear kind of, in my conversations with people about this topic, like how both industries can help each other in this way of resiliency. We never know what is going to happen next. So, yeah, I think it's hard to predict the future but to have the ability to rely on each other in the sense of like sourcing animals, for example. I think it's a good view towards that. Yeah.

Participant
Well, as long as dairies need to have their cows, calve every year to freshen, to be able to produce milk, then there's going to be a byproduct that isn't a byproduct that they can get. Like you can get $700 for a good beef cross bull calf at a week or two old right now. So that's pretty good revenue for these dairies. I know, in the US that they have, over the years, used different hormones and stuff to keep a cow milking for two years or three years or something and not have to calve. But that's not done in Canada, we still have to fresh and so there's always going to be that calf to do something with.

Researcher
Yeah, yeah. And I also don't see that changing in the future for the dairy industry in any way. And I actually see more and more motivation for dairy farmers to find an outcome for those calves. Because the alternative is not looking good for them. So for example, veal consumption is declining, there's not going to be an outcome for veal in the short time. So it's good to have this different path. And it's good to have the beef industry interested on that, too. I was wondering if you and your colleagues, or I don't know, if you feel like there's any negative views about sourcing those animals from dairy for beef farmers in any way? Or do you think is mostly a positive impression about it?

Participant
Well, at a time like this, where herd is shrinking, and everything is short of animals, I think that everybody's going to perceive it as a good thing. If our herd increases, again, which is a very cyclic industry, if we get way too many cattle again, and not enough feedlot space, and not enough demand for the meat and stuff, then they can start looking at it and say, well, the dairies are over supplying or those calves are over supplying the market. But as long as you have a product that's equal to by using these crossbreeds and stuff, then it shouldn't be a problem.

Researcher
Yeah, I think it all comes back to everyone being on the same page, as you mentioned. And yeah, I'm hoping to get with those conversations that I'm having and with my research to start more and more engagement between the two industries. So I'm very thankful for you to taking the time to talk to me. And yeah, I think we're in the end of your 30 minutes. Thank you so much. Is there anything that I didn't ask that you would like to share about this topic or anything related to it?

Participant

No, I think you're fairly thorough on that. But if you do have other questions, you can always send me an email or something, I can clarify things.

INTERVIEW P93

[Verbal consent, introduction, and demographics were removed from the transcript to guarantee participants' anonymity]

Researcher
So I feel like a good question to start this is how would you describe the relationship between the beef and the dairy industry in Canada?

Participant
It's, it's fairly cohesive. I mean, a lot of people don't realize, oh, how much beef that we consume is actually from the dairy sector. And it's just a reality of, of the fact that you know, we're making use of, of those cows and the bulls afterwards, right? There synergy simply with the fact that the industry itself at the far end of it, you have the processors, they really don't care. What those animals, if they're dairy or beef. It's what standard they get, and what they're gonna do with it. So those canner cows and dairy products, that's our that's our McDonald's beef, and there's nothing wrong with it. I enjoy. I enjoy a Big Mac. Yeah, so I think it's pretty good. Although, I think we need to start thinking a little bit more outside the box, as we see, I think society's demands for quality of food. And animal husbandry, for example, I think is critical going forward. Yeah, that's where I see. And that's the positive I see with things is that the dairy sector, in a lot of ways, brought technology into our industry, which I think is good.

Researcher
Yeah, I was going to ask you also like, because the way animals are raised differ between these two, like dairy and beef? And why would you say these two ways of rearing the animals have most in common between them too? And why do they differ the most?

Participant
Well, part of it is with the dairy and their breeding program. You know, now that they're, you know, there, there isn't a need for as many bulls and things like that. And so you know, what, so they have, you know, and they're doing gene, basically implanting now, so they don't end up with any bulls. And so, this is where I see is, you know, down the road, the industry understanding with the market is going to be there. And so, as long as we have dairy cows, we will have, like I said, ground beef. And hopefully, I look at our industry, and I'm an advocate for there's no use racing to the bottom. And the large industrialization of a lot of the dairy industry. And I also see the feedlot industry and and the meat industry, everything you know, you have to be big otherwise you can't stay in business is not a really good model, especially for rural economies and small communities. Having said that, I think that for example, in BC and any place that we have the urban setting, and the urban marketplace that those people they're the ones that are wanting good quality beef and they want to know it's ethically raised and they may want to know that you know, it's been raised out on the range versus in a feedlot you know, all it's life that it's you know, spent a good portion of his life, say, on a ranch versus quite literally a cramped up feedlot someplace. So I think there is a market for that. And a part of that, though, is, of course, we need the economy to be there so that people can afford to give you that extra.

Researcher

Yeah, yeah, that that makes total sense. And I hadn't noted down to ask you, and you already mentioned it, the number of meat that from the beef supply that comes from dairy. So I checked it up, and according to the Canadian cattle association is about 20% of the meat produced in Canada coming from dairy. What do you think about this number? Like, do you think it's a big number? Or were you already expecting to be like this 20%?

Participant
I actually think it's actually came down simply because of the fact that they went to implanting the cows themselves, you know, some of the cows and some of the barns. I mean, the whole you know, the longevity of the cows has changed as well, right.

Researcher
Yeah, yeah.

Participant
So, yeah. But the one thing that I was interested in, seeing was, like I said, I've been working with a number of dairy farmers that have moved up into the interior here. And that's what they're doing is they're raising their heifers in their stock and the one guy, he said, you know, he bought a ranch. So he's maintained 100, head of beef cattle. So everybody says, he's a dairy farmer, he's kind of a rancher as well. And part of that is that that's part of what he sees for a future. He's going to have a feedlot. He's buying land to produce feed. But he also recognizes that he's going to have excess feed that isn't going to meet quality standards. So he realizes that, you know, if he has his feedlot he can be basically doing background and with lower quality feed,

Researcher
I see. Yeah, like there's this clear line between the dairy and beef is getting not so clear, anymore, right?

Participant
And I see an opportunity for so many smaller ranches that instead of trying to maybe run a herd of commercial cow-calf, is that they should contract themselves out to basically how do you put it in race or background, you know, dairy, dairy calves, for the dairy industry down the coast. They don't have they don't have room down there. But if they bought those, those young calves up to the interior, and some of those operations actually raised them up here. These are ways that we can integrate and benefit from each other.

Researcher
I see. Yeah. This is actually answering my next question already. That was about the influence, what kind of influence you think this dairy supply to the beef can have to the beef industry, and like the market, but also the image, and if there are any challenges or opportunities with that.

Participant
Put it this way, the beef, the McDonald's? You know, I can't say for sure that that is dairy or grind that goes into the burgers, but I've been told that but I have no problem with that. So that's where, you

know, when I buy that burger, or I believe the people in Canada, that's one thing that happened when BSE occurred, our beef consumption went up. And that was because we had people realizing that our beef in our industry or the ranching sector, they think about that default on the range and a good healthy, you're an animal. And you know, we refer to it as harvested in the sunshine because your cows are out eating grass and taking the opportunity of grass that's there. And so you know, quite literally we're being really environmentally sensitive because we're not going out and destroying areas we're quite literally just harvesting the grass that's there and if you graze it properly, you're not you know, sustainable and so people were buying and you know, they'd love that and so yeah, they that's when they buy their, their hamburger, that's what they think of is that that range, you know, but not realizing that, you know, a lot of the industry is food production and those cows are standing in the feedlot. But having said that, that's not a negative because it's I believe our feedlot industry it doesn't have the bad players that it used to have simply because there's not room economically to be a bad player anymore.

Researcher
I see

Participant
That's the positive we had that is the same as a dairy farmers, dairy farmers, the old mom-and-pop operations? Well, you know, some of them are very good. I can remember seeing some very bad operations, not only facilities-wise, but just health, you know, herd health. And just you know, how they treated their animals whereas what I see now is a lot of the industry, the big farms that are coming in, you know, they've recognized the how important it is to keep their cows happy, because changes their production.

Researcher
Yeah, it's hard to put like everyone in a bucket, right, all their dairy farmers and all beef ranchers, because they have different systems within each of those. So it's important to recognize that, yeah, okay, yeah. So now I have like, the three main categories of animals that represent this 20% we were talking about. So basically, the animals that come from dairy that go to the beef supply, and then I would like to ask you specifically about each of them. And then I have a little bit of like a text to add a little bit of information to that. So the first I would like to talk about is cull cows. So basically, a definition that we can use for cool cows and, and this is also for the beef, not only for the dairy is an animal that cannot meet or maintain performance and economic criteria. And then in Canada, around like 30% of the dairy herd is replaced each year. So every year 30% of the dairy cows in the country, they are removed from dairy farms, and most of them end up in slaughterhouses. So going to the beef supply chain, and maybe becoming burgers that like you were saying. So any first thoughts about this, like from the beef's perspective?

Participant
No, I think it's, it's necessary. Makes sense. And I kind of chuckle because people get upset, the jello was made of bone and marrow and are going well. But that's how you realize how much of a cow can actually be utilized in the sense of, you know, it's, in fact, that's my one knock on, say somebody's having a rural slaughterhouse because, you know, probably only 35% of the animal actually gets

consumed or utilized. Where the packing industry and stuff I mean, like I said, everything gets gets to go someplace, sort of thing. So, yeah, that's where I really believe in it's been, we have bring about efficiencies as well.

Researcher
Yeah, that totally makes sense. Maybe even like a sustainable aspect of it of using the most you can, right? And, but when we talk with like, dairy farmers about the cull cows, one concern that many people have is like the condition that those animals arrive at the slaughterhouses, because, as we talked, like the definition of cull cow is the one that is not fit to be kept in the dairy herd anymore. So sometimes they do not receive so much care, or they got to this condition because of some like health issue. Do you think that could be a concern for the beef side as well?

Participant
Yeah. And that's where and that's where I've always, you know, well, I get the fact that people want to have real abattoirs and you know, what your backyard butcher shops and things like that. I've always kind of been against it simply because of quality. And I hate to say it, but I can remember, you know, some of the abattoirs around here, I remember taking some of our animals dropped it in there and and first thing in the morning and there's cattle in there, but in the one pen, there's a dead animal already, you know that somebody's you know, had a problem with that animal and so it was wasted over there too. To get butchered and your kind of going, ehh, I don't know. Personally with our ranch, or, you know, when we dealt with animals like that, they got buried try to get rid of bad for yourself. Don't try to sell it or anything else. Yeah. And that's my fear is that, you know, sometimes economics force people to do stupid things or sometimes simple greed too.

Researcher
Yeah, that's unfortunate. And I feel like some people would say that in the beef side didn't goal is the slaughter house. So you're you're raising those animals to reach that point while the dairy is focused on the milk production and what they can get from the cull cows they can get. So it's not an animal that is focused to reach the slaughterhouse house. And that may be why they don't get like they're with such good, like, state.

Participant
Yeah. And that's where I guess that's where I was thinking that it's too bad, more of those cull cows couldn't be humanely put down perhaps at the farm. And then, you know, they wouldn't go into the food are humans consumption, they wouldn't say go into the dog food industry, or, you know, what I, my brother-in-law has a class is a class D or F abattoir license here. So he had to factor abbatoirs that he, you know, he's allowed to process I think it's eight animals or something a year. And so it's kind of an experimental program that he got involved with.

Researcher
Oh, that's very interesting. I feel like that's something my partner would be interested on. Because he really misses the beef farm, we can definitely not have a big beef farm in Canada. So maybe that will be enough.

Participant
Especially for rural communities. It's necessary. Yes. I don't want to see them down in the Lower Mainland, because you know, you need space so that well, just because you have the risk of smell.

Researcher
Yeah, it needs to be in a good area in the correct area for that. Okay. Well, my next category of animals are veal calves. And then many people are not aware of this. But a common practice on almost all dairy farms is that the calf is separated from the cow as soon as it's born or like a few hours after. And then the sort of like this cow goes to the milking herd and then the destination of the calf depends on whether it is a female that needs to be kept in the herd to replace. So it would be a scenario where the calf stays at the farm. But if it is a male dairy calf or a female that is not needed to stay in the herd. In Canada, historically, these animals have been sold immediately into the veal industry. And then these animals breeds are mainly Holsteins. And secondly Jerseys. So the veal calf ended up being another dairy animal like dairy genetics, that is commercialized as beef in the market basically. So any thoughts or what's your opinion about the veal calves in the in the beef market?

Participant
There is feeling sorry for the cows, but again, even with our ranch, we used to always end up put sometimes he follow him, but you know, you have extra twins, for example. Or sometimes Yeah, you just have to see it because sometimes the cow might, something might happen that she can't, she was just stressed. So she will produce milk she ended up have no milk Connor, so you have to pull the calf sort of thing. So I feel for those cows, but if they're socialized, it's not as though they've really missed out on. Sadly, they are somewhat terminal with the fact that unless they go on to milk production, like you say they're pretty well, their lifecycle is probably finished by the time they're less than a year. Or just over a year.

Researcher
Yeah, yeah. And then again, most of the things I bring are from the dairy perspective because that's what we usually talk to, the dairy farmers. And for dairy farmers they see they veal calves as a surplus of their core business, which is producing milk. So we have done some research. And we found that many of those surplus calves, they do not receive the same standard of care as like the female cows that are kept in the herd. So, for example, they receive less amount of colostrum or even less amount of milk while they are kept at dairy farms. So, any any thoughts on that? Do you think this, this could be a source?

Participant
That's where again, it's sad because, yes, it's, it's that standard of care and the ability for the farmers to understand what's humane and what's, you know, it's husbandry of the animal, you know. That's the one thing. Again, I think the positive I've seen with the dairy sector is because of the awareness of, you know, the need to take care of those cows, because of the high investment and, and how it actually is the answer to get higher returns is quite literally taking care of your animals. And that's a good thing. And we're seeing more people, the industry is changing. And I think even in the beef industry, you know, the old, you know, we're seeing it throughout how people manage their animals. People are a little bit wiser, although hate to say it, they're still love. I guess yahoos are unkind people? Yeah.

Researcher
Yeah. Yeah. Do you think it could be any, like reflections to the beef industry over this? Considering that in the end, these animals are so old, like with the beef label?

Participant
No, I don't think you can, you know, if he gets into politics that way. I, it doesn't benefit anybody by trying to, you know, say, well, that's dairy product. So people don't buy it, or it's part of our industry. And while maybe somebody people might want to know. I think it's positive that they're there.

Researcher
Okay. Yeah. And, yeah, so

Participant
I blame them for creating, you know. I don't know if you've ever heard about this. But the reason I'm sure that we had BSE show up was from the fact that the dairy industry because I was talking to a dairy farmer, once I was complaining about the cost of milk supplement. And he was talking about the fact that what they do is they were feeding bone meal is a protein you build up is a cheap source of protein. And that's where BSE or things like that happen is because you're feeding back. That's, that's where I'm sure England and other countries, but the trouble was, because the people were pulling calves off and feeding bone meal to their calves. Yeah,

Researcher
yeah, that's a practice that now at least is illegal in Brazil, because of that disease specifically.

Participant
It's illegal in Canada as well. But like I said, it's funny how some people, you know, this guy was he was only about five years ago. He just happened to be up in a place visit. Why don't you do this and you kind of going like, you serious?

Researcher
Yeah. And that has consequences for everyone. Right? Because like.

Participant
It does, yes. That's one thing I do with my job. Because I deal with trying to help people help themselves. That's the one challenge you're dealing with. Just education. People, I see very much a lot of positives, but I also see the same old kind of, you know, the traditional attitude that you know, this is the way we've done things forever. So why would we change and, you know, it's a challenge sometimes.

Researcher
Yeah, yes, we hear a lot of that not only from you, but in other areas involving farming, basically, meaning those that are very culture or family based. It's harder to change human behavior, basically.

Participant
It's it's totally it's, you know, it's like religion and politics, you know, you get into, to how people manage their animals. It's it's, it's, it's very sensitive. Yeah,

Researcher
I totally understand that. One thing that you mentioned before was like this concern of, let's say the public about how animals are raised and like the ethical side of things. One thing that the dairy industry talks a lot about now, mostly recent, is this cow-calf separation. So, the calf being removed from the cow like soon after birth. So these may be coming a big concern in the dairy side, or like the public not agreeing with that. As a beef farmer, what what are your thoughts on this practice and any specific concerns about it?

Participant
You would think that it should be accommodated, but then again, the challenge with it is, you know, what's, where do we come up with a number that's actually going to change things? I guess, is it a question if they're being mothered? Is that what we're expecting? Or is it something that we want to make sure that their quality of food that they get the proper colostrum and nutrients from the start? We talking kind of nutrient health or psychological health.

Researcher
Is it a hard choice there to balance?

Participant
Yes. And I think there is a balance, you know, can be done. It's I guess, it's, it's like pain management. You know, we never used to use pain management and because of policy, now we have to, but because we've adapted, we kind of often, kind of, may comment to ourselves is why didn't we do this before?

Researcher
Yeah, yeah. Sorry. Oh, I was gonna say it's hard to change, but it's possible.

Participant
And that's, again, how you know, it's all education is too much, but especially with people to want to be educated. But I like to use the word consultant. Part of my job is, you know, trying to well convert people over and so, you know, when you start telling new information that you've told them a year before that you've got that sort of thing, and they have converted? Yeah,

Researcher
It's a long process.

Participant
It can be a very long process, it's very challenging, but I've found with the part of that's my, my mandate is, is to change people's attitude not towards beef consumption, but towards quite literally

environmental management. You know, it's, it's rewarding when you can get people to be this way. You can get old dogs to learn new tricks.

Researcher
Yes, yes. That applies for so many scenarios. Okay, I have one more category, and that's the last one, but it's also the one I'm most curious about, that is the dairy beef calf. So we talked about how the veal industry has historically been the main market for the surplus dairy calves, but the veal consumption in Canada but overall North America is declining with time. And then this resulted in a lower like economic value of the surplus dairy calves in some regions of Canada. So to overcome this, the dairy farmers have started to use beef genetics in part of their dairy cows in so this way, they can include increased the economic value of the animals. So we call this like a dairy-beef system, where the calves they don't go to the veal market, but they are rather slaughtered at the same age as the beef calves that are raised by the farmers at about 18 months of age. So the pathway of these calves would be something like they are born in a dairy farm and then they are separated from the cow soon after birth, then after a few days, they are transported out of the dairy farm to maybe a nursery farm and then potentially a grower in the background or to then go to the feedlot, which is also the destination of the animals that are raised in beef operations. So this change would result in more supply coming from dairy farms. So probably that 20% would increase even more. And, and those animals coming from the dairy in the beef, so either coming from dairy farm or from a cow calf operation that will have a similar, end of the road, basically, and they would reach the market in a similar way. So what what is your opinion about those dairy beef cows? Have you heard about about this before?

Participant
Yeah, I have familiar with, you know, the, the genetics and what they're doing, I think it makes sense. The one thing that the market wants is, is uniformity. And that's the one thing with the dairy industry with the genetics and stuff you are getting uniformity service things. So the way I see it is in dairy industry is going to stick around. It's a market that is the one thing that I know. He mentioned about the amount of colostrum and food that those calves actually get. I know from the perspective of buying milk replacer for like, say calves that we've had to take off your cow or whatever the price of milk replacer used to be pretty cheap, but it was just kind of powdered milk and it was pretty cheap for a bag it was like eight bucks. Well, now it's up to 77 or 80 bucks. And so were used to be able to you could afford to invest in buying a calf or feeding that milk replacer you actually have to think about it now because you're gonna be investing a couple 100 bucks into that calf to get to the stage where it's going to be growing on grass.

Researcher
Yeah. Yeah. And do you see this as being an opportunity or more could be a challenge.

Participant
I see it as an opportunity because the other thing with it is quality control. And well, you know, we can raise a lot of cattle out on the range. The reality of it is is that we're going to have to change the industry because we're running out of land, quite literally take advantage of grass. So, this is where I kind of have promoted through the years about not racing to the bottom but trying to you know, I got asked here I did an interview on a radio CBC here a couple of years ago when they changed the Food

Standards and mentioned that we should be consuming less red beef and of course I had to do an interview after a well known scientist give a talk about why we should be using less beef. I had to follow up, and you know what? I actually agree with them. But my angle on it is I said you know

[Interview was interrupted by phone service issue. The interview continued the next day.]

Researcher
Oh, yeah. I don't know if you have a couple like minutes. I just wanted to finish your conversation. I really have very few questions left. I don't want to use much more of your time today to

Participant
Yeah, no problem. I'm answering phone calls. Today I'm dealing with a number of situations. So okay.

Researcher
Yeah. So I think where we stopped was when I asked you, so we're talking about those dairy-beef calves and like this dairy beef system that may be start being established in Canada now. And I was asking about how do you see the future of those dairy beef operations? Or if there is a future at all for them here?

Participant
And my comment was that, yes, I think it's their critical part of our industry now. One has to realize it's not just about growing those animals, it's about the feedlot industry, it's about who's growing the feed for them. And it's about the abattoirs. And at the end of the day, what those animals are being used for. And so the they've got a niche that they're filling right now. And that's what I think we need to look at is, you know, where our industry is going. I know places like McDonald's and things like that have been working with the Canadian Cattlemen's Association, talking about the industry, where it's going, and part of that is to secure a good healthy source of beef.

Researcher
Yeah. Yeah, that sounds great. And then you mentioned about how those animals basically are treated on those systems. And I wanted to ask more now specifically about the rearing of those calves and then mainly before they actually reached the beef side, because like the way the dairy and the beef calves are raised since birth is very different. We talked a little bit about that. Do you think there are any possible concerns of how these calves are being treated in the dairy side before they reach the beef industry?

Participant
Because I don't know. I haven't been I've been on several farms, but they've been, how do you put it, tours of the farmers there, the farms that are doing it right. And they're very proud of their operations. And you know, in fact, I always use the one tour with was with a bunch of others part of the Fraser Basin Council and they had big tours. We had environmentalists, regional district people, there's a whole cross-section of, of intelligent people I like to say, you know, aware people, but not farmers, and

it was great, because a lot of them didn't understand how a dairy farm actually works and what's required nowadays, right? And so it was great. At the same time while I'm on the road. And just because I know I get around sort of thing, I know there's operations that I certainly wouldn't want to be a calf bull.

Researcher
Yeah. Yeah, and then so let's say what you would describe is the best way of rearing those calves like in an ideal world, how do you think those dairy beef calves should be raised?

Participant
I think it's about the one reality is those those calves are terminal and that's it's like pigs or chickens. Yeah, it's about dopa that quality. SCIENTIST NAME is the psychologists on cows and things like that, whether they would be disappointed, you know whether they need sunlight, or if they, you know, is warmth more important or like you say food and diet requirements, or even contact with other animals? I'm not sure. But that's where I think the industry would do themselves a favor, if they sat down and actually determined some protocols and talking to people like SCIENTIST NAME. So they make sure and get it right versus, you know, like if SCIENTIST NAME said that told me what's the story about a chicken in California now gets to see the sky. But the chickens in California don't really care about the sky. But there was certainly like a nest box.

Researcher
Yeah. Yeah. And I like you mentioned about, like the industry reaching out to, like researchers on are the psychologist of cows, as you said. What about the industries talking to each other, like the dairy and the beef. Do you think there should be any agreement between those?

Participant
I don't think there needs to be agreement. But I think as, as things move forward, they're certainly between industries, there needs to be dialogue. And it's just not between the you know, it's not cow calf producers, because we have the feed industry, we have clearly abattoirs industry, you know, it needs to be continued dialogue on that. And I think the one thing that has been, I think positive with the Canadian Cattlenmen's Association, I mentioned McDonald's and a number of other, wow do you put it fast food companies the stuff they do have, I think, what is it now it's the Canadian Beef Council, and it was part of some of the original meetings that this is what they were talking about. I think, yeah, we need those people. Everybody to understand about our industry, so we can stay in existence. I guess we're not competitors against each other. We're part of we should be working in synergy. Yes.

Researcher
Yeah. Yeah. It's like, it's many people should be involved. Let's say it's not a one industry problem, right?

Participant
Yes. And this is things that I promote within my role. For example, a number of years ago, we had the health, interior health or coastal health, all of sudden became worried about abattoirs. And so that's where they changed all the abattoir licenses and made it so that you really weren't allowed to butcher in

your backyard itself. And so people that you know, all excited about that, and we're making all this noise that we need more abattoirs, and yada, yada, yada. Well, when you talk to the abattoir industry that were up in legal at that time, what they needed was an adjustment from us in the cow calf industry, and the feeder industry to make it so that they had cattle to process year round. Because what I found when I talked to them was the fact that two, three or four months of the year, they had everybody demanding that they take on butchering for them. And then for about seven, eight months of the year, they were running at about 50% capacity. So they were losing money.

Researcher
Yeah. Yeah.

Participant
And so we're, you know, I came back to our own industry and said, you know, if you want Joe Blow to stay in operation, we as an industry or or local community better start figuring out how we're going to help them get the animals. Yeah, yeah. Back to government, because and this is where I explained to them. By opening up three more avatars, it's not going to change the difference without going broke. Which will have so they can be economically viable. So part of that, I'll give you my political rant as an example. But I said what they need is cooler space, because that was the limiting factor. The butchers I found could actually produce or they could process more hours but the limiting factor was the fact they couldn't afford cooler space. So if government was going to help anybody all you need to do is go to the abattoirs become legal and give them better cooler space.

Researcher
And those are things that you only are become aware of when you start talking to many people involved, right, like

Participant
Exactly.

Researcher
Yeah, and that's what I'm aiming to do with my research now like talking to you and in more beef industry people because every time we talk about those dairy beef calves or the dairy beef systems, we are just talking with the dairy side. So my study, this study that we're doing now in this interview is basically the first actually asking the beef side their thoughts on it. So yeah, thank you for being being part of it.

Participant
Well, I've always felt like I've always the person asked the bizarre questions, because I always have a different perspective. But I'm always a winner. I laugh I deal with government consultation the odd time and I always like being one in the room that they're always they'll always be talking about an issue. And they'll always be oh, well, please opinion. And I always give my opinion. And I'm always met with sometimes with a minute of silence. And then I know I give them the answer. They really didn't want to hear, but it's relevant. And that's why I get called in because that's what they need, is somebody from a different perspective to actually get the vision on?

Researcher
Yeah. They know that they need it.

Participant
Well, that's where I look at it is you need do you need somebody to recognize your fault. People's, shortfalls of the industry rather than just praising it always.

Researcher
Yeah, exactly. Well, I have one last question for you. And that's like a different scenario that I don't know if you heard of before. But one practice that many, many, many is something new now in Canada, in dairy farmers are using embryo transfer. So basically, they transfer the embryo of pure beef calf, into a dairy cow, and then they can have basically beef calves from a dairy herd, which is a little bit different than the dairy beef calves. But any, any thoughts? Any opinion on that?

Participant
I could see it coming. I guess, in the sense that, you know, we've been dealing with AI since the 1970s, as a norm, I guess, in the purebred industry. And, you know, and that's it comes down to cost right. But now, with animals being up much more valuable, the economy on that is turning around, so I can see how that's going to be that's going to be a big part of the industry. And that's where, like I mentioned that I can see even, for example, my family's own ranch, we have a commercial cow calf. We're dealing with range issues. So we've often thought well, maybe we should just give up our permitted range and change or you know, go away from being commercial, you know, selling basically, calves. Nice to know, we have a fairly large acreage, maybe we should just fence that off, increase our forage on or grazing ability on that land and, and maybe partner up and not even own cattle but just bring cattle in and not to be a dairy farmer, like you're saying you could background those cows, right, rent them out on us grass, yearlings type of things. So these are the things that as an industry, we need to keep our eyes open and take advantage of opportunities, I guess. So I'm not against it.

Researcher
Yeah. So you believe that will be a good opportunity for the beef side partner with some dairy farmers. Okay. Nice. Yeah. This is off script. But I went to Ireland earlier this year. And that is exactly what many farmers are doing there. My colleague, her family had a full cycle beef operation. And then her I talked to her dad, and he said, Well, I'm not getting any younger, and my kids are getting out of the house. So they partner up with a dairy farm and they buy their calves. And then they just like do the rearing up them. And then his way less work because the calving was the worst for him. He said, So labor decreased and the quality of life for them increased. So there are other countries doing that.

Participant
They took a holiday for what to do.

Researcher
Yeah, they were like, “What do I do with all my free time now?”

Participant
Well, and that's the thing with it is that and the one thing about the dairy industry is they have cash flow. And that's the one thing that our industry doesn't often have. And this is where I look at it as those opportunities is that yeah, it could, it could actually keep some small operations actually afloat.

Researcher
I see. I see. Well, that's all I had. Thank you and feel free to share any thoughts that I didn't ask you about that you think would be important to what everything we were talking.

Participant
Yeah, sure. Feel free to contact me if you have anything. Any other questions, whatever. And, and like I said, but your you mentioned the card, the chance you'll need to go for a cup of coffee.

Researcher
That's great. Thank you so much. Yeah, feel free also to send my contact. I would love to have more people that you think would be good candidates to participate. Now that you've been one you know how it is. So if you know anyone that would like to talk to me about this, I will. I'm definitely looking for more people.

[End of transcript]

INTERVIEW P94

[Verbal consent, introduction, and demographics were removed from the transcript to guarantee participants' anonymity]

Researcher
If you had to describe the relationship between the beef and the dairy industry in Canada, how would you describe it? And you can be both historically or any changes that you've seen recently?

Participant
So for the rest of Canada, I can't speak super clearly to that. I can speak BC specifically, we have a fairly good relationship between beef and dairy. I think that could be said for the rest of the country as well. Like I said I can't speak intimately to those details. But for sure in BC, we're very integrated. Obviously, a lot of dairy cattle end up in the beef supply chain. Whether it's culled dairy cows after they're done their production cycle or obviously with the beef on dairy crosses that are coming up. The industries are very integrated.

Researcher
And what of influence do you think the dairy can have in the beef production and vice versa? How can it make the two industries influence each other?

Participant
Yeah, I think that we do. I don't know if you know, there's something called the beef cattle checkoff that every time an animal is sold, there's money that goes towards the province and different industry organizations within the province as well as there's a portion that goes to the national organizations. So the National organization.... Do you know about the checkoff and how it works?

Researcher
No, no, this is the first time I'm hearing about it. That's great to learn.

Participant
Okay, very, like... Sorry, a little background information on it and then it'll make more sense. So the Beef Checkoff, it's used to run our national organization. So it runs Canada Beef, which is our marketing organization. It runs Beef Research Council, which is our research. Public stakeholder engagement, which takes care of exactly what it sounds like, public perception and that kind of thing. As well as.... I'm missing one. Public stakeholder, engagement, beef research. There's some money that goes towards a marketing group, [name], and I think that there's one more it's not important for the conversation. But any animal that sold in the beef chain goes to that. So dairy does pay a portion of that as well. Right? So every time a dairy cow is marketed for meat, that money is charged as well. Provincially, our Dairy Association has access to those funds. I'm not sure if the other provincial agreements, I won't speak to that. But in British Columbia, they do have access to those funds. So our provincial organizations are the BC Cattle Feeders Association, the BC Dairy Association, BC Cattlemen's Association and The Breeders and Feeders association. So in those meetings, and in that whole context, there's a lot of information sharing back and forth. So there's brainstorming and hey, this

is what we're doing for research on the dairy side. This is what we're doing for research on the beef side, how do they complement each other. So there is quite a bit of information sharing back and forth between the two industries. There's obviously some animosity between the two, especially when it comes to trade. Supply management is in a very different space than... We are in somewhat free market, or free enterprise, how we sell our product. But they definitely do, there is quite a bit of, like I said, I guess that would be the best way to sum it up is quite a bit of information sharing, obviously, with the beef on dairy, like to beef, beef cattle bred to the dairy cows. That's a large influence. And that's kind of changing the game quite a bit. And now there's maybe a little bit more integration.

Researcher
So it's great to hear about that. I think the two industries are more connected than I first thought. It's great that there's this like sharing of knowledge and information. It is great to hear about that. Another thing that I identified when I was researching about this, not this relationship, but also mainly the supply from dairy to beef, is that as you mentioned, about 20% of the meat production in Canada comes from dairy already. And this is a data that is already kind of two years old. How do you feel about this number? Do you think that's expected? Do you think this is going to change?

Participant
It think it is fairly accurate. Yeah. I don't know what they're considering the beef and dairy cross calves. But as far as the cow production, obviously, like a lot of dairy cows go into ground beef, right? So yeah, I think that number, I don't see any change really in it.

Researcher
Yes, yes, this number is supposed to count all animals. So from cull cows to also the calves. So considering that, how do you see that number changing in the future?

Participant
I think it's going to increase. If they're going to consider the beef on dairy crosses as part of that number, then yeah, I think that that's just going to increase. A lot more dairies are obviously using the beef to breed their cows, gets a more desirable product. The straight dairy cattle, you know, to get the calves out those, they don't feed as well, there's some complications with those and the crossing them with the beef definitely takes a lot of that out of it.

Researcher
Yeah, I was just going to cover exactly that. I have a short paragraph explaining, basically, that the dairy industry has a lot of calves that they don't need. So their main product to sell is the milk. And consequently, they have calves that they cannot keep on the dairy farm. So using this beef genetics, you probably already know all of that, is a way of improving the value of those animals and having this outcome to the beef supply. So my question on that is how do you feel about this practice? What are your thoughts on the increased use of beef genetics in dairy herds?

Participant
I have no problem with it. We have low beef cattle numbers, those numbers are still going down. I think that it helps both industries, when there is a good story to come out of it. Right now, a lot of people

would view the calves that come... We have to keep the cows pregnant to keep milk production. So a lot of people view that calf as an unwanted byproduct and a commodity, which I guess I suppose cattle are a commodity, but at least if we are improving genetics, I think people can view that a little bit differently, that they're going into the beef cattle supply. I don't think the general public views it right now when they see a dairy calf go into the feeding sector. It's kind of ugly to them that "oh, that's a baby dairy calf, that an ugly industry". Where I think if there's a bit of a nicer story behind it, or the public perception is a little bit better, when it's "oh no, that's a beef calf going into the beef herd", right? It's the same animal, but the genetics are a little bit improved, we can feed them a little less, or feed them for less amount of time, carcass quality is improved, etc, etc. So yeah, I don't see anything bad about it. The dairy industry needs to continue going and thrive. And if that's a way for us to get more beef cattle, I have no problem with it.

Researcher
Yes, I've heard that before in the other conversations that I had, that it's something that can benefit both sides. Because beef needs those animals and the dairy needs to have an outcome for those extra calves. So it's almost a logical solution. Thinking about that practice, are there any ways that we can guaranteed success in the future? So in this sense, I'm asking not only about genetics, but what can dairy farmers do to guarantee that their calves are going to be good animals to the beef supply in the future?

Participant
Yeah, I think that that's just going to be a natural progression to be honest. Obviously, those calves are going to be worth more money than, you know, a bull calf from a dairy cow previous of straight dairy calf. Like a straight Holstein calf say, their value is very low, where as soon as they're cross beef on dairy, now, it's a black hided animal that we can kind of sell as a beef animal. Its value is going to increase. Its value is going to increase even more if the dairy producer is vaccinating them properly, and their nutrition is proper. And the person that's raising them from a calf into a ruminating animal, that they've done a good job of doing that and transitioning them to where they're going to be on feed. So all of those husbandry practices and good feeding practices and veterinarian practices, that's all going to. I think it's a very natural progression. We know in the US, it's already happening, that they already are making sure that these calves are getting the very best start from the very first days that they're alive and the colostrum that they're getting, and then the calf grower supplement that they're on and the vaccination schedule. So they're already seeing the value in these calves, it's just going to be a natural progression from there, that the dairy farmers, it's not a... Hate to say it, it's not a throwaway byproduct of them raising the milk anymore. It's now a valuable product for them. Yeah, we also talk a lot about that in the dairy side on. Unfortunately, the value of those animals ends up not justifying some of the standards of care. So farmers do not have the infrastructure to keep them the same way as the calves that they're going to be replacement calves. So you mentioned properly rearing them, focusing especially on the amount of time in the early days of their lives, that they are in the dairy farms, what would you consider to be the proper care for those calves? I'm not gonna have the proper timeline. I don't know how many more people you're interviewing or how much more research, but I have a couple of people that would be really useful, I think, for you to maybe chat with that we can talk about afterwards, just remind me. But making sure that they're getting their vaccination, that would be the big one, right? That they're on a good vaccination schedule, that they are being properly fed to set them up

for success later, so that they are getting the appropriate... And I don't, that's where I'm not going to have the correct information. And the gentleman that I can give you a name for... That they're getting to their stage where they're ruminating in a proper nutritional way, that they're not just being fed milk replacer, that they're getting proper health checks that their diets and their rations are created, so that they're going to be a better animal on the outside. I think that's a lot of stuff that definitely, like you said, to make sure that all of those practices are happening, and that there is some money going into those calves. That that's happening. And I don't think that that is, because they were a throwaway product, right? Which is horrible to say, but you know, you know the industry?

Researcher
Yes, yes.

Participant
Yeah, making sure their nutrition is optimal and the health side of it are the kind of the biggest ones. I know that there's farms, there's more and more firms that are starting to raise calves, I guess. So getting them from where they're a milk drinking animal into a ruminating animal, that's kind of that tough transition there. Not tough, but it just needs to be done to benefit the calf. So, more of those types of barns where they do have the facilities and the infrastructure that they can raise those calves.

Researcher
Yes, yes. And then definitely having these increased the value of the animal is a big motivator that will allow farmers to provide more for those animals. And we definitely talk about using beef on dairy as a way to even improve animal welfare. Because then these animals can receive better care because of their increased value. And this will consequently help them thrive in the future into the supply chain. Right? Some things that we also talk a lot about on the dairy side are some practices that are not well perceived by the public. So for example, cow calf separation, so for dairy, they need to milk the cows, so the calf is removed as soon after birth from the cow. They're usually housed individually and they don't have pasture access or outdoor access. Those are all practices that usually don't happen in the cow calf operations in beef. So where usually the calves for beef would be sourced from. What are your thoughts on these practices and how that could influence this future dairy beef system that may occur in both industries?

Participant
I don't have concerns about it. I think that there's enough technology now and enough information and knowledge that they're able to still make sure that that calf is receiving optimal nutrition. That they're, you know, them being housed separately. That's a... While it might not seem like an animal welfare aspect, it is. Because that's protecting them from disease, they're very susceptible to disease when they're younger. So that's keeping them from commingling until they have a bit of an immune system, then they can commingle with their friends. So yeah, I think that I don't have a concern over it. I think that where we are in technology and the amount of research that's been done on how to make sure that these calves are receiving everything that they need nutritionally, there's not a concern that they're not subpar to their beef, traditional beef and, you know, cow calf counterparts.

Researcher

Yeah. And then we talked about how to make this dairy beef system a success, or what is it that the animals needs, what dairy farmers can do. On the other side of the coin? Are there any challenges or roadblocks that we can predict? And maybe prepare? Do you see any challenges basically, in the future of those systems in any way,

Participant
Probably a bit of capacity. As far as having either the facility or the people that are able to, so you know, the calf lifecycle, they can't, they have to be individual for so many days and they are on milk. And then once they start transitioning to where they're introduced to food, you know, that's a couple of weeks or 10 days, or whatever it is, they're offered hay or silage, or grain or whatever their ration is. Having the people in place like having the capacity or the different farms that kind of have that expertise. I think that may be a bit of a challenge. But it might not, the dairy farmers may just have to, that might just be a consideration for them going forward, right? Is that that's something that they're going to have to allow for. But that would probably be the biggest one that I can think of to be honest.

Researcher
Yeah, something that we could prepare for in advance. But it's also hard to do predict what may happen, right? Yeah. And you mentioned before about some an animosity, I'm sorry, this word is hard for me to pronounce, that maybe some of the farmers may have towards dairy, based on the supply managed system and trade discussions. Do you think that that could be also a challenge in the future? And maybe how can we address that?

Participant
Yeah, I think that maybe there's some very, maybe more experienced generations may have more of that than the ones coming up. I think, you know, we compete with dairy in our industry. For a lot of years, we competed for land. So you know, dairy farms would buy up land at a higher value because they can afford it. And that was maybe a source of animosity at one point. But I think a lot of that is going away as the generations go on. The biggest issue right now, there's some trade stuff that's happening nationally that people are upset about, or that might be more so the government is kind of pitting the two industries against each other a little. But I think that that animosity is going away. I don't think that that's going to be a future. There'll always be a little bit of jealousy, I think. I'm slightly jealous of supply management. But I've chosen to be a beef firmer and not a dairy farmer, so that's my problem. But yeah, I think that there will maybe be a little bit of it. But I don't think going forward for the two industries to work together. I think there's too much to benefit for that to be an issue.

Researcher
Yeah, I do like that view, a more optimistic approach that that may be a problem, but it's not as big as the benefits that can come out of this.

Participant
Yeah.

Researcher

And we do a lot of conversations about communication and responsibility. So for example, we've have done some research in Australia about the same topic on who is responsible for leading this discussion, because there are many things that need to be talked about between both industries, but not only those two, but also the value chain and other organizations involved. So in Canada, thinking about this supply, from dairy to beef and all the things that are involved. Who do you think should be on the table? Have those discussions and maybe who should be the one with the responsibility to lead those conversations?

Participant
Oh, that's a good question. So I would say it probably, at this point with the shortage of beef cattle we have, initially my response would be dairy should be leading those because this is their byproduct that they need to figure out. However, we are in such a cattle shortage and there is still a benefit to beef producers, especially the feedlot, more so beef feedlot. I mean, it's not a benefit to cow calf in reality. It's a benefit to the beef feedlot producers, that they should share it equally to be honest between dairy and feedlots. That being said, I think that there is also a very large space for seed stock producers to be involved. They're starting to select bulls with genetics that will benefit the dairy crosses. So I think that there's also a spot for that. Do you know, have you ever heard of NAME?

Researcher
Yes, I heard about them in another interview, actually.

Participant
Like you should really try. I can get you their, I don't know, if you have their contact information, I can get it for you.

Researcher
Oh, yes, that would be great.

Participant
They would be fantastic. For you to chat with. They are one of the biggest, if not the largest. They are Canada's largest cattle feeder, for sure. But they are also a very large influence in the beef on dairy world. So they are one of the biggest beef and dairy cross feeders in North America. So they would be very valuable. And they know the marketing side of it. They are a veterinarian, so he knows the health side of it. And very intimately knows all of these answers. But yeah, so I think, to answer the question from my side, definitely the feedlots and dairy, as well as the seed stock producers, I think have a piece of that that they can share.

Researcher
And should also maybe genetic companies being involved in the conversation, what do you see is their role?

Participant
I think, I'm probably missing them in that stock piece. That's probably where their connection is, definitely that the genetics company should be involved.

Researcher
And thinking about the research or the information that we have to make decisions about the rearing, the genetics, and all of that, this question may be more to benefit me personally. What do you think are the big next questions that researchers can address to help farmers on moving forward and in the dairy beef system basically, in the future?

Participant
I don't know that they're... How do I word this delicately? I don't think there's going to be option to not accept it going forward. I think that this is the way that it's going to be going forward. I think this is a very efficient solution to beef cattle shortages, because we have large shortages right now in our our cow herd is getting smaller and smaller. It's a solution for dairy producers to add value to their product with a product that was a byproduct that was near, not completely but as low value. It's a public perception conversation that looks a lot better. And in a world where efficiencies and sustainability and all that other stuff is is very prominent. I think this is a very acceptable solution to a lot of that. So I don't know that there is a lot of maybe the sustainability questions. You know, there could maybe be some conversation around that is, how does this fit in with the sustainability platforms? How does this fit in with creating a more efficient animal? Obviously, genetics are a huge piece of this. So we're taking a dairy cow that has the genetics to produce milk and have high volumes of milk? How do we complement that with genetics that are you know, the marbling genetics and the carcass quality, other carcass quality genetics, the production or the what's the word? I'm trying to say? I guess the production genetics coming from the beef side as well. So, you know, there's an opportunity to create a very manufactured animal that... What does this actually look like? You know, we know that we can raise it shorter period of time than we can, you know, it takes it's a shorter period of time to get them to slaughter than it is on a traditional dairy cow. How can we increase that? What other technologies can we implement, where does implant technologies or other feed additive type technologies fit into this and, and how do we Yeah, I think that that's probably a really great question to go forward with. If you haven't already thought of that one is maybe that whole, where does this fit? And how does this look? How does it benefit or increase the sustainability conversation? Obviously, that's such a big thing in the world right now.

Researcher
Yeah, it's definitely of interest for everyone to investigate how it influences sustainability. And also, maybe many of those things we are only going to answer with time, because the more people start raising those animals and adopting those systems, that's how we're actually going to see it working in practice. But definitely there are questions that will be helpful to address in the long term. And talking about technologies, as you mentioned, another thing that people have been talking about, is embryo transfer. I don't know if you heard about it before on that sense. So basically, they would get fully 100% genetics of beef embryo into a dairy cow, which would generate the gestation to produce the milk for dairy, and then this animal would be 100% beef calf. How do you feel about that practice? How do you see that being implemented, maybe...?

Participant

I just had this conversation with our nutritionist who's another person who might be good for you to interview as far as the, if you were looking for information more on the raising of the calves and what that needs to look like, and how that needs to be better. But anyways, they, we just had this conversation, and it's too costly right now. And there's not enough people that are trained, that are skilled, to be able to do the embryo transfer to really supply that. But yeah, essentially, you're just going to be using that cow as a house to produce a calf. And to get a pure beef calf out of it would be absolutely spectacular. I think it's going to be there someday. It's just too costly. Not enough known about it right now, more and more and more people are getting into it. And you know, you're seeing a lot of people. It used to be very elite to do embryo transfer, where now, most of the purebred breeders are doing some kind of embryo work. So I think that that's just going to grow and progress into that whole side of it. That will be the way that it goes eventually. I don't know how soon that's going to be but.

Researcher
Yeah. Would you have more interest in those animals compared to the dairy beef crosses?

Participant
Absolutely.

Researcher
Yeah. Because of the genetics influences a lot right? On the quality of the of the meat in the end?

Participant
Well, yeah, what we can do is we can literally, we're just taking a completely manufactured animal, right? We're saying I want this dam, and this sire. I know the dam's traits. I know the sire's traits. This is a calf we want because this is going to be the most, you know, optimal animal to feed. And you stick it in the cows. And then you're going to pull, you know what's going to come out, be born, and you're going to feed it nutritionally, it's going to be set up, you know, very strategically, so that that animal is just going to be created for efficiency, right?

Researcher
Yeah, yeah. And benefiting both sides at the same time.

Participant
A hundred percent.

Researcher
And I have another question that now I don't know if we covered already or not. But you mentioned about the public perception. And definitely, we all see this practice as helping the public perception, especially for dairy in this sense, because the alternative of selling those calves to beef would be way worse for animal welfare perspectives and radical perspectives. But at the same time, we wonder if the public perception of beef could be influenced because for now, the beef is perceived more in a pasture base, while the dairy is more confined. Do you have any thoughts on that on maybe having these dairy animals coming to beef, possibly having an influence in public perception about beef production?

Participant
I think as long as there's still ranchers that are viable, and there is still that story to be told, I don't think it'll be an issue. I think it maybe could even be a benefit further, because now that might be its own niche market eventually, right? I think they're both have space for a niche market. I think that with a lot of what we have right now for you know, you can you can eat natural beef or you can eat grass fed or you can eat organic and some of its very unattainable for people. But, you know, having more options in that. I never think that food options are a bad thing. I think the more people you know, there's people that are going to only buy conventional beef because that's what they can afford or because that's how they choose to spend their money. And then there's people that are going to really like the idea of going to the rancher to purchase the ranch raised, pasture raised, grass fed option that's going to cost more, but that's what they want to do. I think that it's actually maybe a benefit to public perception, in that it's just creating more options for people to choose from. And I don't think any of those options are ever bad. I don't like it when the different, you know, grass fed versus grain fed when they're pitted against each other. I think they're there to benefit each other, because it's just giving people a choice. Yeah, it just having more options. As we see in Europe, they have so many labels nowadays, so many options. So it's good to have a few more options for our markets as well. Yeah. And then you mentioned about still having the ranchers, and there may be a premium value for those animals. Absolutely.

Researcher
Yeah. How would you see that as for your feedlot? Let's say, would you still have interested to purchase those animals from the ranch compared to the dairy? Or how would that work? Do you think?

Participant
Yeah, I think that there's, I think it's going to be equal opportunity. We obviously purchase calves based off of, a lot of the time, is reputation. So, you know, if a rancher is doing a really good job of raising calves, and we know that they're animals that we want to feed, we're gonna buy those ones. If a dairy is creating calves that we want to feed, we're gonna buy those too. So yeah, I think that as it progresses, it's just gonna naturally evolve, where people are going to have their choice of how they market and where they market and where they're purchasing from. For us, it's not going to make a difference. We'll still be looking for the same things. There's going to be people that are good producers and people that are poor producers, and we're going to seek out the ones that we think are doing a good job.

Researcher
Yeah. And then also having the traceability of those animals and going back to where they came from. Yeah. So again, the product, yeah, that's a system that can happen, for sure. Right, in the future. And then talking about the future, I only have one question left. We went through these very smoothly, I didn't follow my order, usually.

Participant
That's okay

Researcher

That's great, because it means that we were just having a conversation. That's what I wanted. And I have my last question is, let's say 30 years from now, you will enter a time machine travel time. How do you think the beef industry is going to be looking like in Canada? And I know, it's a hard question. But how do you imagine is going to be looking like? It's going to be in the future for sure.

Participant
I think it's gonna be a lot smaller. Dairy is going to be predominantly where we get our beef from, I think these cross bred animals or maybe not even cross bred, whether they might be embryos, I think that that's going to be 30 years from now, probably by then that's going to be the majority of it. But yeah, I think that, we have a need for cow calf. It is required. There's a very large environmental portion to that, and a management of a lot of our land that is not accessible for anything else, but raising cows. So I think that there's always going to be a need for it. And until we figure out something different, but yeah, I think that this dairy thing is going to expand greatly. Yeah.

Researcher
Yeah. Okay. Yes. Those are all the questions that I had planned. And I don't know if there's anything else that related to this topic that I didn't ask you about, but you would like to share?

Participant 33:42
I don't think so. You're pretty thorough on it.

Researcher
Okay, great. That's a good sign. Yes, so I will just stop the recording now.

INTERVIEW P95

[Verbal consent, introduction, and demographics were removed from the transcript to guarantee participants' anonymity]

Researcher
So my first question that I have for you is if you had to describe the relationship between the dairy and the beef industries in Canada, how would you describe it?

Participant #1
Maybe I'll let my husband take that one. We don't have a lot of dairy in our area. Central and Northern Alberta, I would say more central, I guess. So but he could probably talk on this more, my husband, NAME.

Participant #2
Hello, can you hear me good?

Researcher
Yes, I can hear you.

Participant #2
What do you mean by the relationship? Like?

Researcher
I mean, what those two industries have maybe more in common, but also what did they differ the most? How would you describe them?

Participant #2
I mean, for the most part, the dairy has cattle they can't use for milking, right? So the steers and some heifers for breeding that are left over for breeding, that will be culled. And so those work out well on the feedlots. The relationship is good. You know, we're always looking for something to feed so that we can buy some from the dairy side of it. But other than that though... I think, you know, the relationship was good, but it's just a relationship in necessity, maybe more than anything. They need to find a place for the calves and we have that ability to do so.

Researcher
I heard the word "symbiotic" used before to describe it, in a way that the dairy has some animals that the beef can use. So that feeds into what you explained as well.

Participant #2
Yeah, exactly. Yeah.

Researcher

And I was doing some research on the dairy supply to beef. I found some data from 2020 that about 20% of the meat production in Canada comes from dairy already. I was wondering if this 20% is a big number for you, or do you think that's expected? And how do you think maybe this number may change in the future?

Participant #2
Yeah, I'm not sure on the number. I honestly have no idea. I've never followed up on that. It could very well be so. I really don't know, to be honest.

Participant #1
We just heard it. We just started feeding dairy and dairy crosses in the last five years, so fairly new for us as well.

Participant #2
But I don't know how many dairy calves make up the total feedlot, like I wouldn't know that. Maybe like 20% seems high? But it very well could be. There's more dairy in the east, obviously, than there is in the West. In the West, on the feedlot side, I don't think it's 20% dairy. But again, I really don't know, I would just be guessing.

Researcher
Yeah, but it's interesting to hear that you recently started to raise more of those animals coming from dairy. So maybe that could also be a sign of a possible increase in the supply coming to beef from dairy?

Participant #2
Not really in Canada a whole lot. With dairy being supply-managed, there's very little growth in that industry. You know, just as the supply needs to be, as we have more consumers and the demand for milk goes up. And then there is some growth, they're also becoming more efficient with milk production per cow. So you know, it's not like they're expanding very quickly. So a lot of it, we see a lot more dairy, like straight Holstein and Holstein crosses coming out of the US. Mainly because in the US, and at least in the northwest, the packing plants quit harvesting those cattle because they wanted a more consistent meat. So they were really sticking to straight beef. And so then a lot of those dairy cows ended up coming north at a price point that made our feedlots up north here, excited to buy them. And maybe we feed them differently than they did in the US. Maybe we're more consistent with meat up the other end and what they're grading. But we haven't seen at least from our packing plants, we haven't seen that they turn away from them. And which has caused a lot of US dairy producers to take on beef genetics, and they're really, really getting better at that I think there's something like 3 million or 4 million dairy beef calves in the US now. And majority is just because they're more efficient animal. The grading is more consistent. And it brings more value back to the dairy.

Researcher
Yeah, and then so you mentioned many of those animals are coming from the US. And is that the case for your animals? So the ones that you have those dairy beef calves are they coming from the US or some from Canada?

Participant #2
Majority are from the US. Maybe a percent of them are Canada but the cross cattle have all been out of the US. The street Holsteins, we get some Canadian ones, but the majority out of the US.

Researcher
Okay. And I was just wondering why and you mentioned that a little bit but maybe if you could explain more, why you have those animals coming from the US and not from Canada. So you mentioned the supply management system reduces the amount of animals that you have available.

Participant #2
I think it reduces the amount of animals available in Canada but also in a way it reduces the amount of beef-Holstein crosses because they're just the smaller cow calf or the dairies haven't been really breeding to the beef. Part of the could be from what I understand is because then AI and a lot of Hutterite colonies have dairies and AI was outlawed on a lot of colonies. I think that's starting to change. For religious purposes, they can start to AI so we might start seeing more cross breeding but the US dairy industry like to give you an example, I don't know how many dairy farms in Canada milk more than two or three hundred head at a time,. Whereas in the US you're going to see 15, 20, 30 thousand, even 40 thousand head dairies. But in Canada, we will never see that under supply management because nobody can afford to expand like that. And so the expansion is drastically larger in the US on the dairy side. So we're seeing more and more calves there. And they're fairly attractive for us to bring across for the most part. Cost wise, it's all about cost. If they fit the bill, and we buy them. If they don't, we don't.

Researcher
I see. And then yeah, you mentioned that probably some more Canadian dairy farmers will start using beef genetics on their herds. And this is something that we are noticing as well. If that happens, what can make this dairy beef Canadian calf attractive to you? If you had to compare it with the US and then you mentioned cost? Would that be the biggest one or is there anything else that could come to mind?

Participant #2
I mean, genetics plays a huge part of it. In the US, a lot of the dairies they're still use. Oh my gosh, no, I can't think of the breed. Not Holstein, but Jersey, And the Jersey cows don't perform as well. They don't get as big in the feedlot, even if they're crossed with beef. And so when we buy US calves, there's quite a bit of Jersey influence in there. We don't see that a lot in Canada and Canada's majority Holsteins and the genetics is pretty good. So if they breed with some really good beef genetics into those. Those cattle would be more efficient, which would make it more attractive for us to bid on them. So.

Researcher
Yeah. And then you have some experience with those animals. And I believe you also have pure beef calves. Do you notice any difference between them in your farm or anything that it's beneficial or maybe not as good as your beef calves?

Participant #1

Yeah, we noticed quite a few differences. So with the dairy crosser, the dairy calves, they come to us at about 350, 370 pounds. And so they're on full feed right away, and they stay for a whole year. So it's a lot easier having them on the single ration coming in and not having to step it up compared to the beef cattle, they come in, you know, generally you want them in a little bit heavier, about five, six hundred pounds. They come in in the fall, off the moms or different times of the year, depending on calving. And then we only have them for about the 220 days. But they start off on a background in ration. And we have to step them up. So it's about depending on what side they are, when they come in, if they're you know, four or five hundred pounds or 500 pounds, then we have to start on on the first backgrounding. If they've been backgrounded somewhere else, then we can step them up. So it's about five different steps in the ration going from background and all the way to finishing. So for that age and not weight. If it's custom feeding. So we do some custom and some cattle we own. And so the custom, the straight Holsteins and the Holstein crosses are definitely more beneficial for us in our feedlot because they are with us for a full year versus the 220 days. And they're already on that one ration so we don't have to worry about any problems with feed that way. I do find there is a higher death loss with the Holstein. Straight Holsteins, they're a little bit higher, and then the crosses are a little bit lower than the straight Holsteins but still higher than all beef cattle.

Researcher
And do you have any information on the cause of this mortality or do you have any suspicions on the reason why?

Participant #2
A lot of it has to do with just chronic pneumonias. Those little calves get sick along the way. Their immunity isn't quite as strong because they're not getting mother's milk all the way along. So a lot of it's just chronic pneumonias that festers over time.

Researcher
So I see yes. And maybe this is related with my next question, because one of the things that we think of when you think about dairy beef calves is that those calves, they are raised as dairy. So they go under dairy farming conditions, and they go through some dairy farming practices that are different from what happens in tall calf operations in the beef side. For example, the dairy calves, they are housed indoors, they are also housed individually, and all of those practices that only happen in dairy. So how you thinking that different calf rearing practices between the dairy and the beef farming can influence the life of those calves. So how much they are successful in the beef supply chain in the future?

Participant #1
Well, I think ultimately, I think it kind of goes back to the genetics as well. If you do have good, like with the cross, if you do have a good beef genetics, they'll definitely be a more hardy animal for all weather conditions. But yes, in the barn, and the dairy industry has been doing a lot better job with us. But it used to be so strict that the biosecurity too and you know, kind of like what we're seeing with the pigs and the birds and that with all these different flus that's wiping out these whole herds. So dairy has definitely done a really good job over the last, I would say 20 years, selecting better genetics, even just in straight Holsteins. So for the hardiness and for more disease resistant. But again, I think the biggest thing is when they come off their mum that early, because they come off what day five, isn't it or even

sooner. So it's been a while since I've worked on dairy farms, practices are different now. But coming off their mum like that, I mean, that's a lot of stress at any time. But I think the younger, you know, not having that colostrum and not having that milk longer from the mum is harder on the animal to the effect, but they're not as hardy in the... Yeah. Because we have all of our calves. Yeah, all of our cows are calved outside. They do get moved from kind of paddock to paddock to prevent disease. And then once they're, you know, about five days or even a week old, they'll go up to a bigger pasture.

Researcher
I see. Yeah. And I actually had a question exactly about the cow calf separation that happens on dairy. So the common practice is to remove the calf from the cow soon after birth. And this is definitely something that doesn't happen in the beef industry. So maybe you can comment a little bit more on what are your views about this practice? And maybe as a beef producer, what would be your recommendations for dairy farmers moving forward? If it's interesting for them to review this practice or not in the future

Participant #2
Excuse me. I mean, they have to do that in order to get the milk production right? So if you left the calf on too long, you're missing out on a lot of milk so it's definitely their goal. Their businesses to produce milk and the best way to produce milk is to feed an animal for it and not let the calf have it so then they you know, feed the calf. Basically the stuff that the calf gets is a byproduct of milk. So it's a powdered milk that is made from the milk protein after it's filtered and that. So again, the calves are still getting milk but it's just in a different form. But I mean, it's I think they've done a pretty good job over the years of keeping those animals healthy and anytime you do raise animals in a confined area, there is more chance of sickness. But no, I think they're doing good and they've got to develop on timing and everything else. And the cattle while we get from them are pretty resilient. Unfortunately, some of them just come with more issues, maybe than they would have if they were out, roaming around. But you know what's ideal isn't always practical.

Researcher
Yeah, definitely. Yeah, so we talked about genetics. But now I wanted to also focus on some practices that maybe we could do on the dairy side. So let's imagine a good friend of yours is a dairy farmer. And then he decided that he wants to start selling his dairy beef cows to you maybe or to another beef farmer, what would be your recommendation to your friend on what are the must does to guarantee that his calves are going to succeed in the beef supply chain?

Participant #2
I think the first thing is to work closely with consultants on the beef nutrition and the veterinarian side to make sure that the calves are getting the proper vaccines and looked after and then on the nutrition side, for sure that they're not doing any damage to that rumen. And because we're gonna have those calves for a long time. So making sure that the feed that they get is adequate, and all things that they need from protein to energy, and the right amount of starch and fat, and things are processed in a way that is going to be very beneficial to the rumen versus something that maybe will do some long term damage. So.

Researcher
I see, getting them ready, nutritionally, to move to the beef supply, I see.

Participant #2
Yeah, to do everything possible to bring us a healthy, ready to go animal. And that's where the consultants like veterinarians and nutritionists are so important.

Researcher
Yeah, and then maybe going back a little, because I'm not definitely not following my order of questions. But this is great. It means we were having a conversation. We talked about the increased use of this beef genetics on dairy. But I'm curious on your general views about this practice? Do you think it is a good thing for the beef industry? Do you think it can come with some challenges? What is your opinion overall?

Participant #2
It's definitely a positive thing from both the consumer and from feedlot side of it. The beef dairy crosses cattle typically grade higher than our straight beef or the dairy cattle. You get more yield than the dairy cattle, a little less than the beef cattle. So a lot of the advantages of the beef cattle really come out on on these cross breed cattle. You know, overall, I think it's a good deal. The genetics is there for them to be efficient, and for them to be also great in yield into a product that the consumer wants and likes.

Researcher
Yeah, and you mentioned it's good for the consumer as well. I will I'm curious on what what are the reasons why you think it's good for the consumer too?

Participant #2
Consumers like a juicy tender well marbled steak. And you get a better consistency out of the dairy crosses than you do in the straight dairy.

Researcher
Yes, definitely. And one thing that we also talk about is that the alternative for dairy farmers of what to do with those surplus calves is definitely challenging. So historically, those extra calves they don't have a reason to keep they were so to veal. And veal consumption is decreasing over the years in Canada. So the the value of those animals is not good, not worth it anymore. So having the option to have the dairy beef crosses is definitely interesting. So we increase the value of those animals, and then we're able to provide more for them on the dairy farm as well. So from what I'm hearing to the beef industry, it's interesting to the dairy industry is definitely industry interesting to keep this practice. How do you think the conversations should happen then in the future about it? Is this something that the dairy industry should be taking the lead on the conversation or the beef industry? I'm curious on how you think those may be collaborations will work in the future?

Participant #1
I definitely think it should be both industries. But ultimately, if it's the dairy industry itself, the individual farms need to make their own decision on where they want to market their calves. And what kind of

calves that they want. The straight Holsteins or the Holstein cross. But ultimately, I think, I mean, if both are involved, it should be both. Both parties, both industries should be together talking on this, because that's, that's why they are separate industries. Everyone has their specialty. Right? So whereas you get some dairy farmers and beef producers and then talks can be had to make the best genetics possible for that dairy beef cross? From all the way from birth to consumers.

Researcher
Yeah, so is it important to have this good relationship, like at the farm level? Between the individual farms?

Participant #2
Yeah, for sure. I mean, the dairy industry has the most to gain from it. Because they're selling a product. In the feedlot, we're just margin takers. So if I pay, you know, $1,000 for this calf, and I know what my feet cost, and I know what I'm gonna get at the other end. That kind of determines what I pay for it. So if the calf is more efficient, yields better, and it's gonna make more money at the other end, I pay more for it. So you know, there could be times on a 300 pound animal if it was sold to me as a dairy calf or as a crossbreed. I might be willing to pay $200 to $400 more for the crossbreed than straight dairy. Well, that's a lot of money for that dairy, to put in their pocket, so they have the most to gain. And the better job they do with genetics, the more we're going to want to buy those calves, because they're going to perform it on our end. So it's good for them to know how those cattle perform all the way through so that they can change the genetics or modify or stay on course, to do what is best, because the better they do on my end, the more I pay for them. So. And since they're the producer, it's in their best interest to follow that up.

Researcher
Yeah, so maybe also having the genetic companies on the table with those discussions as well, in bringing on the research on what are the best genetics for those animals?

Participant #1
For sure.

Researcher
Okay, and then I have a question about a practice that is not common, but it's starting to be researched about, which is embryo transfer. I don't know if you heard about it before, but one of the main discussions on this dairy beef calves is that they are not a pure beef animal. So with the embryo transfer, what would happen is dairy farmers would buy a pure beef embryo and transfer it into their dairy cows. So this cow would produce milk and also give birth to a purebred beef calf. What is your opinion about this practice? Do you think it's sustainable in the long term? Or do you have any concerns about it?

Participant #1
I guess I would be surprised if it was sustainable. But just because the supply isn't there for the dairy industry, especially in Canada at this time. Maybe in the US would be. But also that would be quite costly, you know that. And not every embryo takes, I did a little bit of embryo transferring back quite a

few years ago. But so it's quite finicky. Process. I'm sure it's changed a little bit here. But it just I don't know how the cost benefits would work out. I don't know if there was there would be much.

Researcher
Yeah, it is a costly procedure for sure. And the main reason for people to to do it is to have this genetic benefit of having a pure beef animal instead of a dairy beef cross. But do you think it would be worth it? I guess you need to see the numbers, right?

Participant #1
Well, just thinking of the amount of days on feed and just the different rations that a beef versus a dairy beef cross would need. I think just from thinking of that point, I think that the dairy cross is more sustainable, and more cost effective. But I'll let my husband talk because he's the one who knows the numbers inside and out, I think. And with death rate and all that too, like, yeah. I just think that the dairy cross seems more reliable and you're saying that the carcasses, in the end, they're more consistent.

Participant #2
They can be. If the genetics are right. But...

Participant #1
Yeah, I think it's all about genetics is what it comes down to getting a good dairy beef cross.

Participant #2
Personally, I like the idea of embryo transplants. I don't know the sustainability of it long term. But I think here's an opportunity for the dairy industry, since they're already thinking those calves to kind of do what the sheep did and breed more twins to get two cows instead of one. And the dairies already watching their cows calves very closely anyways. So, as long as that, you know, there isn't a lot of complications on the calving side, to get two cows and instead of one, I think would be very beneficial for the dairy industry, and bring more calves about. So now they would get twice the amount of income from each cow on the calf side, right? So. And so that's where I think an embryo transplant would be very beneficial for them. So, but I don't know enough about it just enough to be dangerous.

Researcher
Yeah, yeah. And then I was also thinking about your motivation, then to buy those animals, let's say if you had the option to buy, hypothetically, pure beef calf that came from an embryo transfer from a dairy farm, or a pure beef calf from a cow-calf beef operation. What are the things that you would take into account to make the decision between those two animals.

Participant #2
Feeding would be the main thing. So the dairy cattle or the straight dairy, or crosses, their rumens are very strong, and they seem to be able to handle a lot of high energy feed for a lot longer than a straight beef animal. So if the dairies are used to feeding the crosses in the straight dairy cows, say maybe a higher energy ration, those calves are small, that might be the exact opposite of what they want to do with the beef calves. And because they could damage the rumen or something like that, that would be a concern I would have, versus raising them on a cow where they're getting the right amount of nutrition.

So that would definitely be one thing. That would be a concern, because at the end of the day, on our end, we're pushing that rumen as hard as we can for the last 180 days of the animal's life. And so that room has already been damaged. And that really affects our efficiency. And there's definitely a greater chance of damage in...

Participant #1
the straight beef...

Participant #2
...and the dairy side of it, right, than raising it off the cow.

Participant #1
They restrict […].

Researcher
Yeah. Yeah, that makes total sense. And I have one more question. But you we already covered a lot of ground. Thank you so much. So my final question is, let's imagine we have a time travel machine. 30 years from now, how do you think the beef production will look like in Canada?

Participant #1
That’s a long time.

Participant #2
I imagine with, obviously with AI and computer systems, we're constantly changing things are learning more. But I don't know, it's a it's an interesting question. For sure, because the cost of grass and everything's getting to be so expensive. And so in my head, I've already been, you know, thinking, is it cost prohibitive to take beef cows, and bring them into a feedlot situation and get twins off of them, you know, select genetic bulls that, and cows that would throw twins, so and then raise up there somewhere to the dairy side of it, versus grazing them just because the grass is getting to be so expensive and so hard to find. So and so maybe the dairy would do more of that as well. But I think over time, whether it's 30 years or 50 years, we are going to see some changes to how beef production from until the feedlot side of it anyways, is done. And I don't know what the cow calf industry is gonna look like over the next 30 years? I definitely, see there's gonna be some changes there. But what all that is gonna look like I don't know, you know, I think there's gonna continue to be protein demand, especially in developing countries that are maybe not used to eating as much protein and they're excited to have beef and higher quality of beef. So I just feel like the demand is going to continue to be there. And you know, there's been lots of talk on plant based foods and that. But they're really not taken off. Even with the consumer, they seem to be a little bit of a fad, they like them for a little bit. And then after that they seem to get away from them. We've seen that with Impossible Burger and, and all the stuff that. They're just not selling.

Participant #1
And it hasn't really decreased the demand for like real beef, for all beef. Even versus other proteins like lentils, and chickpeas. So it's just the demand for, you know, real good beef. And that's something the

lab meat has not been able to produce yet, the taste or the texture. So it's definitely there's a high demand and it's hasn't decreased, it's always increasing. Sometimes the cuts have changed and supply, like in demand, such as some time.... And depending on where you are demographically. You know, not everybody can afford a ribeye steak, whereas hamburgers more.

Researcher
Definitely. And then I like to comment about how the different impact for the feedlots and also the cow calf operations. And I'm curious if you allow me one extra question is how do you think this, maybe focusing on the dairy supply to beef but in general, the future will influence the cow calf operations differently than the feedlots?

Participant #1
I think with the cow calf, you know, they might want to add some more. Go back to some of the tri-breeds or the dairy cross-breeds. You know, as long as the frame and the production is there, I think there's a few tri-breeds that we don't see as much anymore. I'm a big fan of one, but Shorthorn is one of my favorites because the tri-breed and we used to see a lot of that and 80s and 90s. And then Angus got really big and Charolais. So I think those are that's kind of the top two breeds and in our verse. So, but maybe going back to more of a tri-breed or breeding some, even if it's not a third dairy but having some dairy genetics in the beef, more of a blend.

Researcher
I see I see.

Participant #2
What was the question again? Sorry.

Researcher
Oh, yes. My question was how the different operations on beef so the cow calf operation and the feedlots are going to be influenced in the future, considering the this dairy supply to beef?

Participant #2
Concerning to the dairy supply to beef, okay. I'm not sure if it'll influence the cow calf, or the feedlot side a whole lot, just because the feedlots are always going to try to buy anything that's going to be profitable. And when people ask me my favorite animal to feed, I always say it's a [could not hear] The business is to make money, doesn't matter to me a whole lot with those animals are. I know certain animals perform better. But if I gotta pay a lot more for them, and the money isn't there, at the end, it doesn't matter. Whereas on the cow calf side, I think it's the same thing. We're seeing the older, smaller producers retire and we're seeing a lot of consolidation in that industry. And I think we're going to see a lot of genetic improvement in that industry. Because of that, it's a lot easier to run 1000 or 2000 cows and improve the genetics than it is to sometimes do it on a small scale. And because they just get people are focusing on multiple things. So if you're by yourself, and you got 150 cows, there's only so much you can focus on and spend time in. But if you have a couple of 1000 head and also, you know, you have somebody that's solely focused on buying bulls, and genetics and return on investment, and you know, all those business type things start to come out. So I think we see, I think the cow calf side is

still going to be working harder to be relevant in the industry by being profitable. A lot about possibilities gonna come from better genetics.

Researcher
It all goes back to the genetics.

Participant #2
It all goes back to, I mean, it's like this, you know, if I buy an animal at 600 pounds, and I feed it to 1500 in the feedlot, that's my 100 pounds, right? If it cost me $1.50 A pound to put gain on that animal, because of the feed conversion, everything else. But 1500. No, I can't do math, it's $1400 of money, right? And so maybe I buy like calf or for $2,000. And I sell it for 3400. And I put 1400 into it. My breakeven genetics are really good on feed conversion, which we're starting to see more and more getting better. And let's say it's really good, and this is probably over the top. Say it only cost me $1 a pound to on feed now because they're eating that much less, and converting more into beef, and less into heat, energy and maintenance. And now it's just so now listen, that's $900 less than it cost me so instead of paying the guy 2000 or four, sorry, it would cost me $500 less to $450 less to feed that animal, I'm actually going to the next time I go to that farmer, I'll probably pay him $400 more for his calf, if that makes sense. So he's better genetics make a big difference on my end, so I'm gonna pay him more but it also makes a big difference on his end. Because if he's grazing a cow calf pair, a bull on grass, the more efficient those cattle are, the more cattle he can put on the same grass. The more cow he can raise, the more cows he sells, the more money he gets from his cow. So pretty quickly, with genetics, you can almost double your value of your animals compared to your neighbor, just by increased herd and getting more money for your calves. If you work really hard to get better genetics and he doesn't. And maybe doubling is shooting for the moon type thing. But there's definitely a lot in between. There's a lot of room. And that's why genetics are so important for the cow calf side.

Participant #1
And with genetics, they are constantly monitoring. And so geneticists are very important in the beef industry.

Researcher
Yes, definitely. And you also touched a little bit on this relationship. So if you know that the animals that you're buying from these farmer are going to be good in the future is motivation to invest more. So it's also interesting to keep the important aspect of having this relationship with the farmers as well. And this is all I had. Thank you very much. It's been, I think, 45 minutes or more. I really appreciate your time. If you have any more thoughts on everything that we cover, but I didn't ask you about. I'm happy to hear it if you have anything else to share?

Participant #1
Anything? Covered a lot?

Participant #2
Any other questions or want to come for a tour? Let us know.

Participant #1
Yeah.

Researcher
Yes, I will. If you invited me I will go!
