# Supplementary material for: It’s not black and white: Perspectives of Western Canadian beef farmers on dairy-beef production
Source: PLoS One. 2025 Sep 10;20(9):e0330697. doi: 10.1371/journal.pone.0330697 (PMC12422455; doi:10.1371/journal.pone.0330697)
Supplement: S4 Table — Summary table showing the distribution of study participants across different types of beef production operations (e.g., cow-calf, backgrounding, feedlot). (PDF) [file pone.0330697.s004.pdf]

It’s not black and white: Perspectives of Western Canadian beef farmers on dairy-beef production

Bianca Vandresen[1], Daniel M. Weary[1], Marina A. G. von Keyserlingk[1]*

[1]Animal Welfare Program, Faculty of Land and Food Systems, The University of British Columbia, Vancouver, BC V6T 1Z6 Canada

*Corresponding author

Email: nina@mail.ubc.ca (MvK)

Supplementary Material 4. Number of Canadian beef farmers who participated in the study (n=20) by province and by type of beef operation.

**Beef phase**	**AB**	**BC**	**SK**	**Total**
Backgrounding	1	-	-	1
Cow-calf	2	6	1	9
Cow-calf and backgrounder	3	-	-	3
Cow-calf and feedlot	3	-	-	3
Feedlot	-	2	-	2
Self-production	2	-	-	2
**Total**	**11**	**8**	**1**	**20**
