# Supplementary material for: It’s not black and white: Perspectives of Western Canadian beef farmers on dairy-beef production
Source: PLoS One. 2025 Sep 10;20(9):e0330697. doi: 10.1371/journal.pone.0330697 (PMC12422455; doi:10.1371/journal.pone.0330697)
Supplement: Attachment — Submitted filename: Response to Reviewers_Vandresen et al 2025_FinalV2a.docx [file pone.0330697.s006.docx]

**It’s not black and white: Perspectives of Western Canadian beef farmers on dairy-beef production.**

Bianca Vandresen^1^, Daniel M. Weary^1^, Marina A. G. von Keyserlingk^1^*

^1^Animal Welfare Program, Faculty of Land and Food Systems, The University of British Columbia, Vancouver, BC, Canada

--

Please submit your revised manuscript by Aug 30 2025 11:59PM. If you will need more time than this to complete your revisions, please reply to this message or contact the journal office at [plosone@plos.org](mailto:plosone@plos.org). When you're ready to submit your revision, log on to <https://www.editorialmanager.com/pone/> and select the 'Submissions Needing Revision' folder to locate your manuscript file.

Please include the following items when submitting your revised manuscript:

- A rebuttal letter that responds to each point raised by the academic editor and reviewer(s). You should upload this letter as a separate file labeled 'Response to Reviewers'.
- A marked-up copy of your manuscript that highlights changes made to the original version. You should upload this as a separate file labeled 'Revised Manuscript with Track Changes'.
- An unmarked version of your revised paper without tracked changes. You should upload this as a separate file labeled 'Manuscript'.

If you would like to make changes to your financial disclosure, please include your updated statement in your cover letter. Guidelines for resubmitting your figure files are available below the reviewer comments at the end of this letter.

Journal Requirements:

When submitting your revision, we need you to address these additional requirements.

1. Please ensure that your manuscript meets PLOS ONE's style requirements, including those for file naming. The PLOS ONE style templates can be found at <https://journals.plos.org/plosone/s/file?id=wjVg/PLOSOne_formatting_sample_main_body.pdf> and <https://journals.plos.org/plosone/s/file?id=ba62/PLOSOne_formatting_sample_title_authors_affiliations.pdf>

 The manuscript and additional files have been reviewed to follow the formatting style requested.

2. Please provide a complete Data Availability Statement in the submission form, ensuring you include all necessary access information or a reason for why you are unable to make your data freely accessible. If your research concerns only data provided within your submission, please write "All data are in the manuscript and/or supporting information files" as your Data Availability Statement.

 All data used in the analysis are available as supplementary material at:

Vandresen, Bianca; Weary, Daniel M.; von Keyserlingk, Marina A.G., 2025, "It’s not black and white: Perspectives of Western Canadian beef farmers on dairy-beef production.", https://doi.org/10.5683/SP3/SD4SSJ, Borealis, V1

3. Thank you for stating the following financial disclosure:
Social Sciences and Humanities Research Council of Canada

Please state what role the funders took in the study. If the funders had no role, please state: "The funders had no role in study design, data collection and analysis, decision to publish, or preparation of the manuscript."
If this statement is not correct you must amend it as needed.
Please include this amended Role of Funder statement in your cover letter; we will change the online submission form on your behalf. We have added this to the cover letter and also included it in the acknowledgments – please move as you see fit.

4. If the reviewer comments include a recommendation to cite specific previously published works, please review and evaluate these publications to determine whether they are relevant and should be cited. There is no requirement to cite these works unless the editor has indicated otherwise.

Lines highlighted in yellow to double-check lines before submitting

**Response to Reviewers**

**Reviewer #1: 1 – Please consider adding a map of Canada showing the regions where the research took place. This would help readers better understand the study's location and context.**

AU: Thank you for this helpful suggestion. We agree that a visual representation would support readers’ understanding of the study’s geographic context. In response, we have included a map of Canada showing the distribution of beef cattle heads by province and have highlighted the provinces where participants were located. This is now presented as Figure 1 in the revised manuscript.

**2 – The practical reason for choosing this target population needs to be explained more clearly. Why were these participants selected, and why are they important for this topic?**

AU: We appreciate the reviewer’s comment and have clarified the rationale for participant selection in the revised manuscript. Specifically, we recruited individuals who earn an income from beef production and reside in Canada, referred to throughout the paper as “beef farmers.” This population was chosen for two key reasons. First, we sought to investigate the views of beef farmers on beef-on-dairy production, which necessitates engaging those directly involved in beef production, as explained in the introduction. Second, participants were required to reside in Canada because views on animal production systems are shaped by local cultural and market contexts. Given the unique dynamics of the Canadian beef and dairy sectors, a Canadian sample was essential to ensure relevance and contextual accuracy. We have clarified this in the revised manuscript (line 132), which now reads: “Since local context can influence peoples’ views toward animal production systems [25], we recruited only from within Canada to reflect the culture and market dynamics specific to this region.” The participation of beef farmers from the western provinces of Canada reflects the outcome of recruitment efforts, which were employed to recruit participants from across Canada, but resulted in participants being drawn primarily from these regions. This is not unexpected, as the majority of beef cattle production in Canada is concentrated in the western provinces. As clarified in line 135: “Despite the efforts to include farmers from across Canada, including offering interviews in French to accommodate participants from francophone regions, the final sample consisted of participants from three western provinces: Alberta, British Columbia, and Saskatchewan, which are the provinces with the highest concentration of beef production in Canada (Fig 1) [26].

**3 – What was the specific criterion for reaching data saturation? In snowball sampling, recruitment usually continues until no new information appears. Was the number of 20 participants truly enough, or could it reflect that participants were very similar in their views?**

AU: Thank you for raising this important point. We acknowledge the ongoing debate in qualitative research regarding the use of data saturation as a criterion for determining sample size. As discussed in the manuscript (lines 111–118), saturation is increasingly critiqued as a *post hoc* rationale that may not align with the interpretative and context-dependent nature of qualitative inquiry. Therefore, our sample size was not determined based on a formal assessment of data saturation.

Instead, as noted in the manuscript (lines 127–140), sample size was guided by pragmatic considerations, including the exhaustion of recruitment strategies. Recruitment began on September 1, 2023, and continued until May 10, 2024, when no further prospective participants could be identified through snowball sampling.

We have added further clarification in the revised manuscript at line 127: “Given these limitations, our study used a snowball method to recruit a convenience sample of participants for phone interviews; recruitment ceased once all known referral paths had been exhausted.”

And at line 134: “Recruitment began on September 1, 2023, and ended on May 10, 2024. Despite the efforts to include farmers from across Canada, including offering interviews in French to accommodate participants from francophone regions, the final sample consisted of participants from three western provinces: Alberta, British Columbia, and Saskatchewan, which are the provinces with the highest concentration of beef production in Canada (Fig 1) [26].”

Additionally, the final sample of 20 participants aligns with sample sizes reported in other studies undertaken by other research groups employing similar qualitative methods (e.g., Shortall, 2022; Wemette et al., 2020), and while some shared views emerged, we also observed meaningful diversity in participants’ perspectives, as described in the results section.

Shortall, O.K., 2022. A Qualitative Study of Irish Dairy Farmer Values Relating to Sustainable Grass-Based Production Practices Using the Concept of ‘Good Farming.’ Sustainability 14, 6604. <https://doi.org/10.3390/su14116604>

Wemette, M., Safi, A.G., Beauvais, W., Ceres, K., Shapiro, M., Moroni, P., Welcome, F.L., Ivanek, R., 2020. New York State dairy farmers’ perceptions of antibiotic use and resistance: A qualitative interview study. PLOS ONE 15, e0232937. <https://doi.org/10.1371/journal.pone.0232937>

**4 – The discussion could go deeper. Even though the study is based on opinions, some statements from participants appear to be incorrect or misleading. The discussion is a good place to address or correct this kind of information.**

AU: We agree that the discussion offers an opportunity to critically engage with participant statements that may appear inaccurate or inconsistent with current scientific or industry knowledge. As such, we have expanded the discussion in the following lines:

Line 561: “However, the extent to which these concerns are valid is uncertain, as it remains unclear how many of these animals will ultimately enter the beef supply or how they will compete in the market alongside conventionally raised beef cattle. For example, while some participants expressed concern about an increase in the number of dairy-origin calves entering the beef sector, others believed the overall number of calves would remain stable, with the primary change being the use of crossbred dairy-beef genetics. Nevertheless, our study indicates that Canadian beef farmers share concerns that dairy-beef calves could create competition, potentially lowering calf prices for cow–calf producers.”

Line 608: “The extent to which the public becomes aware of the dairy origin of meat remains unknown, as does the potential impact of such awareness on the public image of beef farmers. Nevertheless, beef farmers expressed concerns about the risks this practice may pose to their industry, given the substantial differences between beef and dairy production systems, farmers’ lifestyles, and the role of tradition as a core value among beef producers [46].”

However, we would like to emphasize that, in qualitative research, the accuracy of participants’ claims does not diminish their significance. These views, whether factually correct or not, are meaningful in shaping attitudes, decisions, and behaviours, and thus have real-world implications for the adoption and social dynamics of beef-on-dairy production. In fact, the presence of misunderstandings or knowledge gaps highlights the importance of enhancing communication and knowledge exchange among stakeholders. We have made this point more explicit in the revised manuscript (line 660): “These findings underscore the importance of enhanced communication and knowledge exchange between the dairy and beef sectors to ensure that producers are equipped with accurate information about implications of beef-on-dairy production.”

**5 – Even if the study is mostly qualitative, some numbers, percentages, charts or tables would make the results easier to follow and more engaging (especially for a broad audience interested in the topic such as dairy, beef, environment, market)**

AU: Thank you for this thoughtful suggestion. While we recognize the potential value of incorporating quantitative summaries to enhance accessibility, we respectfully disagree that this approach would benefit the current study. As this research was designed and conducted using only qualitative methodology, specifically reflexive thematic analysis, introducing numerical representations of codes or sub-themes would contradict the epistemological foundations of our analysis.

As discussed by proponents of thematic analysis (e.g., Braun and Clarke, 2006), quantifying qualitative data can inadvertently imply that frequency equates to importance, which risks oversimplifying the nuanced and context-dependent nature of participants’ values and perspectives. For example, a theme expressed less frequently may still hold significant conceptual weight in understanding participants’ positions on dairy beef production.

That said, we have carefully structured the results using clear subheadings, illustrative quotes, and thematic summaries to aid comprehension and engagement for a broad audience. We believe this approach maintains methodological integrity while also supporting accessibility and clarity.

Braun, V., Clarke, V., 2006. Using thematic analysis in psychology. Qual. Res. Psychol. 3, 77–101. <https://doi.org/10.1191/1478088706qp063oa>

**6 – Include more details about the study’s limitations, such as selection bias or limitations of the qualitative method itself.**

AU: Thank you for this valuable suggestion. In response, we have added a new subsection titled “Limitations and future research” in the discussion, where we expand on the limitations of our study and outline recommendations for future work.

We acknowledge the potential for selection bias, as participants were recruited through convenience and snowball sampling. Despite efforts to contact provincial producer organizations to broaden recruitment, we were unsuccessful in securing their support, which may have limited the diversity of participants.

We also recognize that conducting interviews by phone, while practical for reaching participants across Canada, limited opportunities for observing non-verbal cues and may have felt less personal. Additionally, while individual interviews allowed participants to speak freely, they do not offer the interactive benefits of group discussions where ideas can be exchanged and co-constructed. Future studies could complement interviews with focus groups to explore shared reasoning and social dynamics, or with surveys to enhance the representativeness and generalizability of findings.

These additions are now included in lines 682–712 of the revised manuscript.

**7 – How can we be sure the participants gave their honest opinions and were not influenced by the way questions were asked? Is the interviewer experienced in interviewing?**

AU: Thank you for this important observation. We acknowledge that the potential influence of the interviewer and the authenticity of participants' responses are inherent limitations of all qualitative research. To mitigate these concerns, the interviewer, who is experienced in conducting qualitative interviews and facilitating focus groups, took several steps to build rapport and create a comfortable environment that encouraged open and honest sharing. While portions of the rapport-building conversation were excluded from the transcript excerpts and supplementary materials to protect participant anonymity, they played an important role in fostering trust.

We have addressed this explicitly in the manuscript at line 187, which now reads: “All interviews were conducted by the first author (BV), an experienced interviewer who employed efforts to build rapport with participants before interviews began."

**8 – Conclusion (lines 652–653) The first lines of the conclusion say the method was “effective,” but the study does not seem to evaluate the method itself. I suggest rephrasing this part.**

AU: We agree that the original phrasing may have implied an evaluation of the method, which was not the study’s aim. We have revised the sentence to better reflect the intent and focus of the research. The revised sentence now reads (line 714): “This study employed semi-structured interviews to explore beef farmers' views on dairy-beef production, providing a deeper understanding of their perspectives on this practice.”
